# Iridium-catalysed hydroamination of internal homoallylic amines†

**DOI:** 10.1039/d3cc05594a

**Published:** 2024-01-04

**Authors:** An T. Ho, Evan P. Vanable, Chelsea San Miguel, Kami L. Hull

**Affiliations:** a Department of Chemistry, University of Texas at Austin 100 East 24th Street Austin TX 78741 USA kamihull@utexas.edu; b Department of Chemistry, University of Illinois at Urbana-Champaign 600 South Mathews Avenue Urbana Illinois 61801 USA

## Abstract

An Ir-catalysed regioselective hydroamination of internal homoallylic amines is reported. Both cyclic and acyclic internal olefins undergo directed hydroamination reactions with both aromatic and cyclic aliphatic amines to afford a variety of 1,4-diamines in fair to excellent yields. Diastereoselectivity and mechanistic investigations support that for cyclic substrates the reactions are proceeding *via trans*-aminoiridation to form a 5-membered metalacyclic intermediate.

## Abstract 2

The Ir-catalyzed amine-directed hydroamination of internal alkenes is developed. Mechanistic investigations support that a *trans*-aminoiridation is occurring.

## Graphical abstract

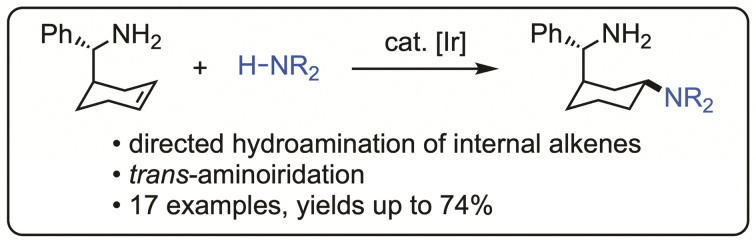

Putrescine derivatives are an important class of polyamines due to their bioactivities as well as their utility as synthons for other nitrogenous moieties (Scheme 1a).^1^ Hydroamination, the addition of a N–H bond across an unsaturated C=C or C≡C bond, is an attractive approach to form these valuable products as it couples two readily accessible starting materials in an atom-economical fashion.^2^ Hydroamination methods commonly suffer from challenges with respect to reactivity, low binding affinity of the alkene to the transition metal catalyst relative to the amine, chemoselectivity as both hydro- and oxidative amination can occur, and regioselectivity as it is generally possible to form two regioisomers. These challenges are exacerbated in the hydroamination of internal olefins due to increased steric hindrance, additional β-hydrides to undergo elimination, and the lack of significant electronic and steric bias to promote regioselectivity.

**Scheme 1:**
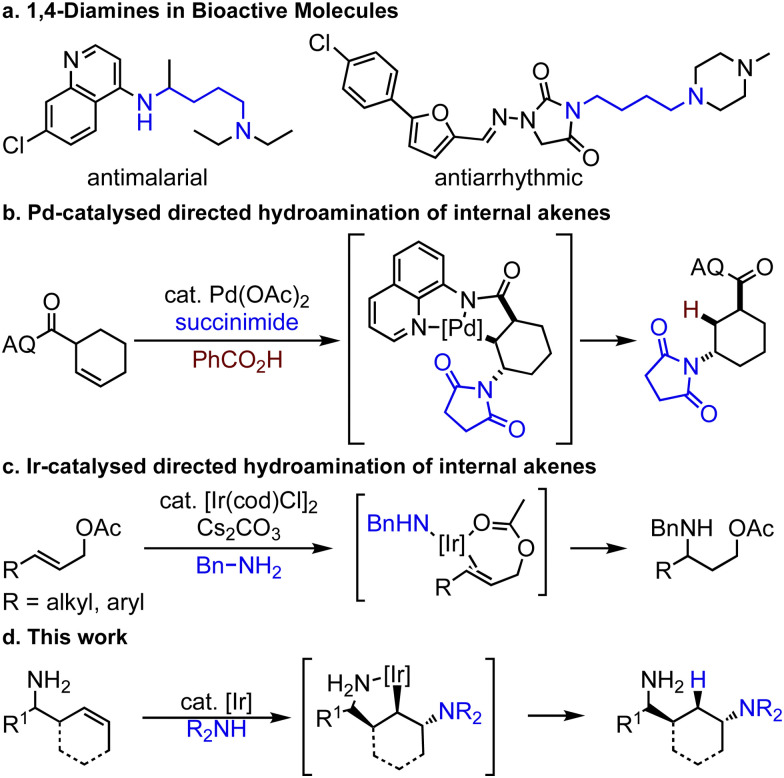
Hydroamination of internal olefins.

Several approaches are known for the hydroamination of strained internal olefins. Milstein reported the Ir-catalysed hydroamination of norbornene with aniline.^3,4^ In this reaction, *cis*-aminometalation occurs selectively across the norbornene and the lack of accessible *cis* β-hydrogens result in no oxidative amination. Reports of intermolecular hydroaminations of unactivated internal alkenes are limited and generally afford mixtures of regioisomers.^5^ Hydroamination of unactivated internal olefins is known to be promoted by a Cu catalyst. These reactions couple a nucleophilic hydride, generated by transmetalation with a silane, the olefin, and an electrophilic amine.^5*a*–*d*^ Ir has also been shown to catalyse the hydroamination of unactivated internal alkenes with 6-amino-2-picoline.^5*e*^

The Hull group pioneered the utilization of directing groups to promote regioselective hydroamination of terminal alkenes.^6^ With homoallylic amines, primary amine directing groups prove excellent at promoting selective alkene hydroamination. This directing group promotes the aminometalation of the proximal alkene by increasing the affinity of the substrate to the catalyst. Further, these reactions have excellent chemoselectivity: upon aminometalation, a metallacycle is generated, which slows β-hydride elimination and promotes selective hydroamination over oxidative amination of terminal alkenes. We have showed that with Ir and cationic Rh, 5-membered metalacyclic intermediates are typically favoured: the Markovnikov product forms with allylic amines, while homoallylic amines afford anti-Markovnikov regioisomer.^6*e*^ In a similar approach, 8-aminoquinoline (AQ) is used to control the regio- and chemo-selectivity in the Pd-catalysed hydroamination of terminal and internal alkenes with acidic amine nucleophiles^7^ and acetoxy groups promote the Ir-catalyzed regioselective hydroamination with primary amines (Scheme 1b and c).^8^ Given the complementarity of the nucleophiles which participate in our Rh/Ir-catalysed hydroamination, both aryl and aliphatic amines,^2*a*^ we sought to develop the hydroamination of internal homoallylic amines (Scheme 1d).

Our initial efforts towards the development of this hydroamination focused on homoallylic amine 1a and aniline 2a (Table 1). Promisingly, employing an iridium catalyst under previously optimized conditions results in 34% yield of 3 with excellent regioselectivity for the 1,4-diamine (Table 1, entry 1). Changing from LiI to Mg(NO_3_)_2_ significantly improves in the yields of 3. A variety of phosphine ligands were screened (Table 1, entries 2–4 and Table S1, ESI†) and all showed similar reactivity with 1a, however *tol*-BINAP was moderately superior as it affords 3 in 80% yield (Table 1, entry 4) in the presence of Mg(NO_3_)_2_. Other Lewis acidic additives were explored and proved inferior (Table 1, entry 4–8, Table S2 and S3, ESI†). The additive is not essential, as in its absence we observe a 34% yield of 3. Notably, the addition of MgCl_2_ inhibits the reaction as only 9% yield of 3 is observed. The optimized condition are as follows: 1.5 mol% [Ir(cod)Cl]_2_, 3.3 mol% (±)-*tol*-BINAP, 25 mol% Mg(NO_3_)_2_, in neat aniline (2a, 7.5 equiv.), at 100 °C for 16 hours. Under these conditions, 1a undergoes hydroamination to afford 3 in 60% isolated yield (2.0 : 1.0 d.r.) as a single regioisomer. When the *trans*-isomer (*E*-1a) (6.9 : 1 *E*:*Z*) is subjected to the hydroamination conditions 3 is observed in decreased yield (35%, 2 : 1 d.r.), relative to the *Z*-1a, along with the remaining 1a (3.3 : 1 d.r.). We hypothesize that the reduced yield with (*E*)-1a is due to it being an inferior ligand for the catalyst compared to (*Z*)-1a. The epimerization of (*E*)-1a is consistent with olefin epimerization under the reaction conditions.

**Table 1:** Reaction optimization^a^

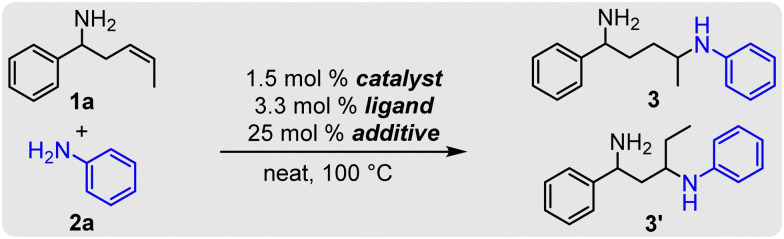					
	Catalyst	Ligand^b^	Additive	Yield^c^	3 : 3′^d^
1	[Ir(cod)Cl]_2_	± BINAP	LiI	34%	>20 : 1
2	[Ir(cod)Cl]_2_	± BINAP	Mg(NO_3_)_2_	78%	>20 : 1
3	[Ir(cod)Cl]_2_	SEGPHOS	Mg(NO_3_)_2_	77%	>20 : 1
4	[Ir(cod)Cl]_2_	± *tol*-BINAP	Mg(NO_3_)_2_	80%	>20 : 1
5	[Ir(cod)Cl]_2_	± *tol*-BINAP	LiNO_3_	74%	>20 : 1
6	[Ir(cod)Cl]_2_	± *tol*-BINAP	MgCl_2_	9%	>20 : 1
7	[Ir(cod)Cl]_2_	± *tol*-BINAP	AgNO_3_	47%	>20 : 1
8	[Ir(cod)Cl]_2_	± *tol*-BINAP	—	34%	>20 : 1

a General conditions: 0.20 mmol of 1a, 1.5 mol% catalyst, 3.3 mol% ligand, and 25 mol% additive, neat in aniline at 100 °C for 16 hours.

b Structures are shown in SI.

c Calibrated *in situ* yields as determined by GC using 1-methylnaphthalene as the internal standard.

d As determined by ^1^H NMR and/or GC chromatography.

Next, we examined the scope of the reaction. Notably, all homoallylic amines which participate in the hydroamination reaction afford the 1,4-diamine as a single regioisomer (> 20 : 1). The conditions are compatible with both electron-rich (6) and electron-deficient aryl amines (5, 11, 12), forming products in 41–51% yields. While the reactions are typically run neat when the nucleophile is a liquid, hexanes is an effective cosolvent. A variety of functional groups are tolerated in the reaction, including halogens (5, 12), esters (11), and ethers (6). Steric hindrance of the nucleophile has an adverse effect as *o*-toluidine affords 7 in a modest 29% yield and mesityl amine does not participate (16). Steric hindrance proximal to the directing group has a significant impact on the reaction: phenyl (3) to phenethyl (4), results in an increase in yield,60% and 74% respectively. More hindered directing groups, *i.e.* α,α-dimethyl, do not react (14). Likewise, increasing the size of R^2^ to a decrease in yield; when R^2^ is ethyl 9 is isolated in 25% yield and only trace 13 (R^2^ = phenethyl) is observed. On the other hand, cyclic olefins show good reactivity: α-(2-cycloalkyl)-amines readily undergo hydroamination as 10–12 are formed in 41–51% yield with excellent regio (> 20 : 1) and diastereoselectivities (12 : 1 to 20 : 1).

The cyclic alkene substrates offer mechanistic insight into the C–N bond formation. The crystal structure of Armstrong's acid salt of 10 shows that the aniline group is *trans* to the directing group (Scheme 2). This suggests either that *trans*-aminometalation occurs or that the catalyst selectively binds opposite of the directing group and that the C–N bond forms *via* migratory insertion into the [Ir]–NHAr bond. Both cyclohexene and sterically hindered homoallylic amines do not undergo the Ir-catalysed hydroamination (neither 14 nor 15 are observed), indicating that the directing group is required to promote the reaction and that *trans*-aminoiridation is occurring. Notably, other iridium-catalysed hydroaminations with anilines all proceed *via cis*-aminometalation – oxidative addition into the ArHN–H bond then migratory insertion. *Trans*-aminoiridation has been previously proposed with nucleophilic benzyl amines in the Ir-catalysed hydroamination of allyl acetates.^8^

**Scheme 2:**
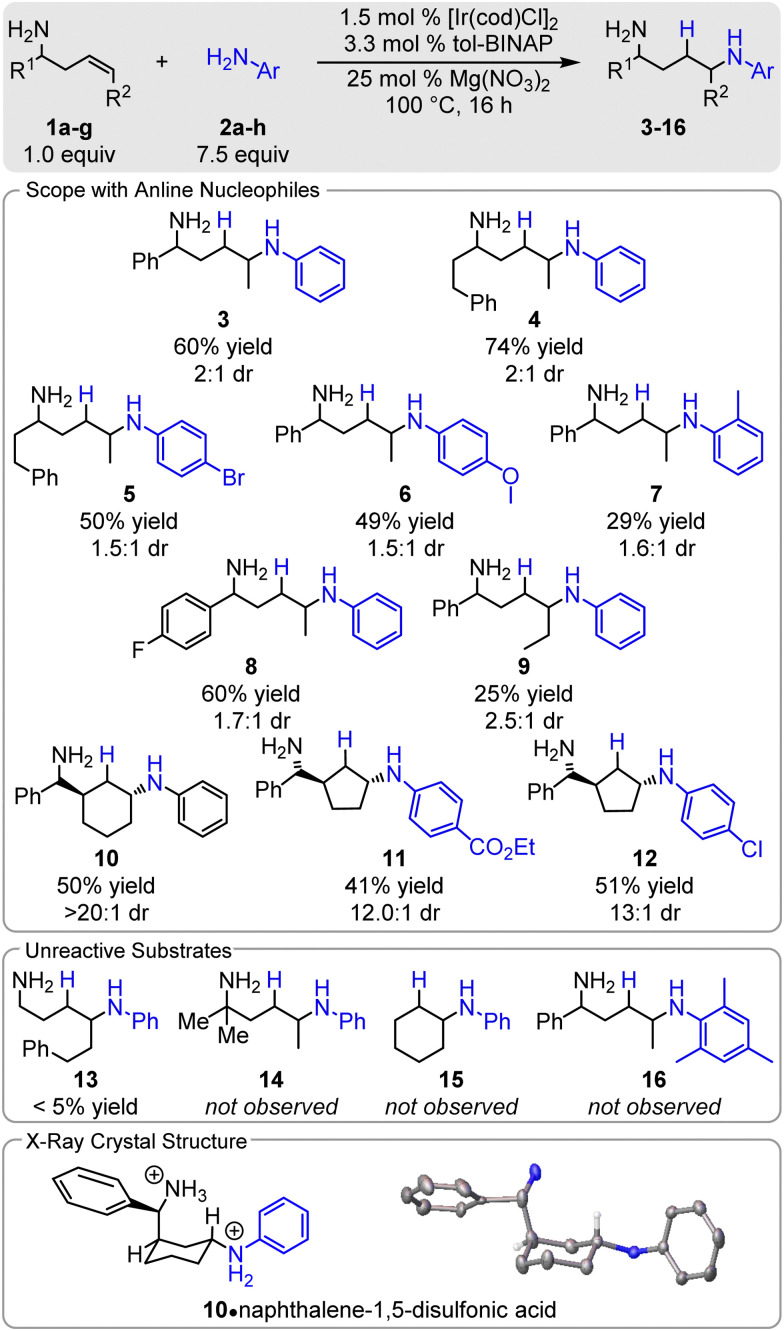
Scope of hydroamination with anilines.

The observation of the *trans*-relationship between the directing group and anilines prompted us to test the reaction conditions with aliphatic amines (Scheme 3). When morpholine is used, both cyclic and acyclic alkenes result in hydroamination products (17, 23). The acyclic alkene affords the hydroamination 17 in a 65% yield (1.1 : 1 d.r.). The low d.r. may be attributed to olefin isomerization and/or poor facial control in the aminometalation step. Alternatively, cyclohexene-derived substrate affords 23 with higher diastereoselectivity (29% yield, >20 : 1 d.r.). The low yield is likely due to the sterically hindered homoallylic amine, while excellent d.r. can be attributed to the inability of the alkene to undergo *cis/trans* isomerization and highly organized aminometalation step. Again, a *trans* relationship between the directing group and the nucleophile in 23•2TsOH is observed. The reaction occurs with a variety of cyclic amines, including piperidine derivatives (18–21) and *N*-substituted piperazines (22). The conditions tolerate ethers (19), tertiary amines (22), and acetals (21). Unfortunately, acyclic primary and secondary amines do not participate (24, 25). Overall, this hydroamination reaction has promising synthetic utility due to its modularity; both coupling partners are easily modified, useful synthetic handles are tolerated, allowing for further functionalization at a specific position. This also allows rapid access to *N*,*N*′-differentially substituted diamines, which are challenging to synthesize using conventional methods. Moreover, diamine products can be transformed into other moieties of equal importance such as amide, cyclic urea, cyclic amide, and diazepane.^9^

**Scheme 3:**
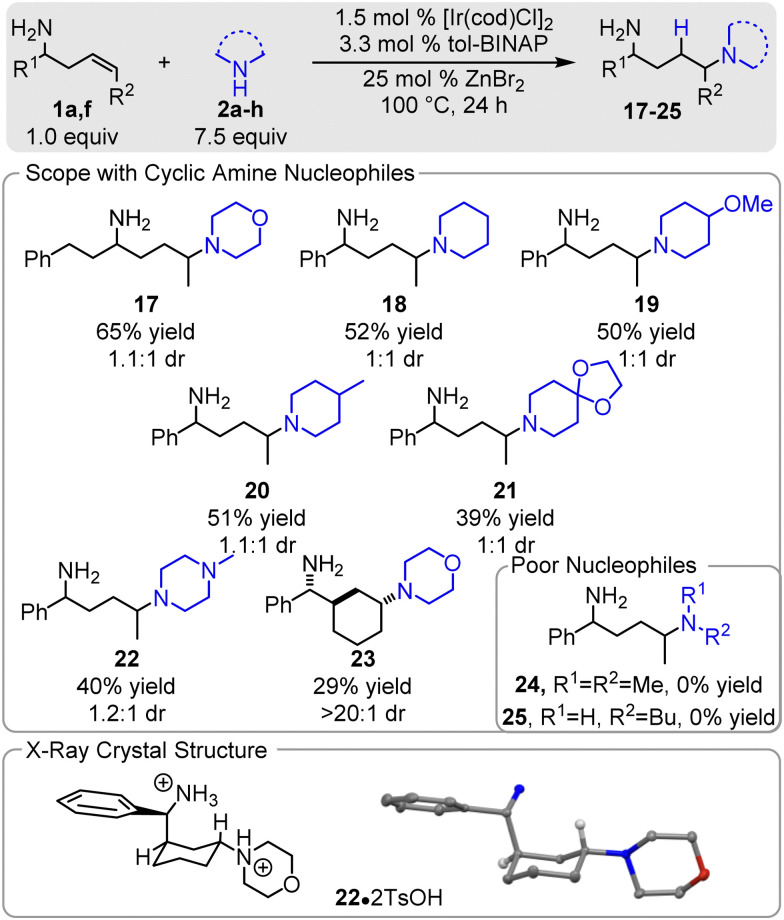
Scope of hydroamination with aliphatic amines.

The *trans*-diastereoisomers formed with cyclic substrates (10–12 and 23) suggest that the C–N bond is formed *via trans*-aminometalation. This is further supported by a competition Hammett investigation^10^ between aniline and *para*-substituted anilines (Scheme 4a): a linear relationship is observed with an *ρ* of −1.5 with cyclic olefin 1d. This moderately negative *ρ* is consistent with the more nucleophilic amine reacting selectively in the product-determining step and *trans*-aminometalation of the olefin. Due to the observed olefin isomerization with acyclic alkenes, we are unable to determine definitively if a *cis*- or *trans*-aminoiridation is occurring under these conditions. An analogous competition Hammett investigation was conducted with 1a; similar *ρ* values with the cyclic and acyclic substrates would suggest that they may be going through analogous mechanisms. Indeed, a negative *ρ* of −1.1 is observed (Scheme 4b). This is consistent with a product-determining *trans*-aminoiridation, however we are unable to exclude *cis*-functionalization, which may have a similar *ρ* value.

**Scheme 4:**
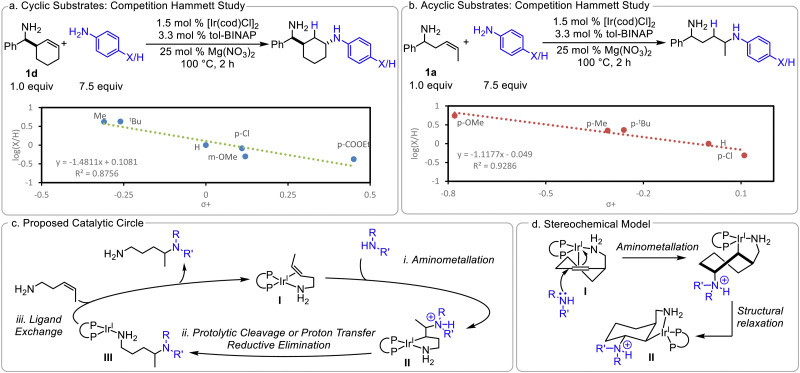
Mechanistic investigation.

Finally, we sought to determine the role of the Mg(NO_3_)_2_. While the hydroamination of 1a does occur without any additive to afford 3 in low yields, added Mg salts significantly increase the reaction efficiency. We hypothesized that these additives may abstract the chloride from the catalyst. To investigate this possibility, 10 mol% Bu_4_NCl was added to a hydroamination reaction to inhibit the proposed abstraction. Indeed, the addition of the Cl^−^ impedes the reaction; only 10% *in situ* yield of 3 is observed after 16 hours (*vs.* 80% yield in the absence of added chloride, see ESI†). AgNO_3_, commonly used for halide abstraction, promotes hydroamination, albeit in a lower 47% yield. This suggests that Mg also coordinates to the diamine product to prevent product inhibition. Taking our observations into account, a catalytic cycle is proposed (Scheme 4c). The Mg(NO_3_)_2_ abstracts the Cl^−^ from the catalyst and 1a binds to afford κ-2 amino olefin complex I. (i) aminometalation occurs to generate the 5-membered metalacyclic intermediate II, (ii) proteolytic cleavage of Ir–C bond affords the product bound complex III and, (iii) ligand exchange regenerates I. The *trans* relationship between the nucleophile and directing group is the result of the nucleophilic attack of κ-2 complex (Scheme 4d). While the proposed catalytic cycle, shown in Scheme 4c, invokes a Ir(i) catalyst and is overall redox neutral, an alternative mechanism would involve oxidative addition into an ArNH–H bond, to afford Ir(iii), then proceeding through an analogous catalytic cycle.

In summary, we herein report an intermolecular iridium-catalysed hydroamination of internal homoallylic amines. The reaction is compatible with both aryl and aliphatic amines, affording 1,4-diamine products in fair to very good yields. Mechanistic evidence supports that an unusual *trans*-aminometalation with an Ir-catalyst and aniline is occurring.

The authors thank the NIH (R35 GM125029), the Welch Foundation (F-1994-20220331), Eli Lilly, and Novartis for their generous support. C. S. M. thanks the NSF (CHE-1945401 and CHE-2003735) for funding of the CREATE program.

## Conflicts of interest

There are no conflicts to declare.

## Supplementary Material

CC-060-D3CC05594A-s001

CC-060-D3CC05594A-s002

CC-060-D3CC05594A-s003
