# Supplementary material for: Iridium-catalysed hydroamination of internal homoallylic amines
Source: Chem Commun (Camb). 2024 Jan 4;60(12):1615–8. doi: 10.1039/d3cc05594a (PMC10846566; doi:10.1039/d3cc05594a)

Electronic Supplementary Material (ESI) for ChemComm.
This journal is © The Royal Society of Chemistry 2024

Supporting Information for:

Iridium-Catalyzed Hydroamination of Internal Homoallylic Amines

An T. Ho[1], Evan P. Vanable[2], Chelsea San Miguel[1], and Kami L. Hull[1,2*]

[1]Department of Chemistry, University of Illinois at Urbana-Champaign, Urbana, IL 61801
[2]Department of Chemistry, University of Texas at Austin, 100 E. 24th Street, Austin, TX 78712.

kamihull@utexas.edu

TABLE OF CONTENTS

A. General Information 2
B. Select Optimization 4
C. Substrate Synthesis 7
D. General Hydroamination Procedure and Product Characterization 12
E. Control Experiments 22
F. Mechanistic Investigations 24
G. X-Ray Crystal Structure Data for 10 and 22 30
H. References 35
I. HRMS Spectra 36
J. NMR Spectra 98

A. General Information

General Experimental Procedures:
Unless otherwise specified, all reactions to synthesize homoallylic amines with air sensitive reagents (Grignards, etc.) were carried out in flame-dried glassware under an atmosphere of nitrogen; mildly air sensitive reactions (such as those involving $Ti(OEt)_4$ were not setup in flame-dried glassware. All hydroamination reactions were, and should be, setup under inert atmosphere; while the precatalysts, ligands, amines, and alkenes are all relatively air stable, the active catalyst is not. Nitrogen was dried by passing through drying tube equipped with Drierite. Air- and moisture-sensitive reagents were handled in a nitrogen-filled glovebox (working oxygen levle ~ 1.0 ppm) or using standard Schlenk technique. Column chromatography was performed with silica gel from Grace Division Discovery Sciences (35-75 µm mesh); all columns were slurry packed. Analytical thin-layer chromatography (TLC) was performed on precoated glass silica gel plates purchased from EMD Chemicals Inc. and visualized with either short wave (254 nm) ultraviolet light or by staining with Ninhydrin stain or $KMnO_4$ and briefly heating. Distillations were performed using a 3 cm short-path column under reduced pressure.

Instrumentation:
^{1}H, ^{13}C, and ^{19}F NMR were recorded using a Varian Unity 400 or 500 MHz (100 or 125 MHz respectively for ^{13}C) or a VXR-500 MHz, Bruker Avance NEO 400 MHz (101 MHz for 13C, 376 MHz for 19F), Agilent MR 400 MHz (101 MHz for ^{13}C, 376 MHz for ^{19}F), Bruker Avance III 500 MHz (126 MHz for ^{13}C, 471 MHz for ^{19}F), or Bruker AVIII HD 500 MHz (126 MHz for ^{13}C, 471 MHz for ^{19}F) spectrometer(s). Spectra were referenced to residual solvent using either $CDCl_3$ (^{1}H NMR: δ7.26 ppm, ^{13}C NMR: δ 77.36 ppm) or C_6D_6 (^{1}H NMR: δ7.15 ppm and ^{13}C NMR: δ 128.60 ppm), ^{19}F NMR spectra were referenced to added C_6F_6 (^{19}F NMT: δ -164.9 ppm). Chemical shifts are reported in part per million and the multiplicity is as indicated: s (singlet), d (doublet), t (triplet), q (quartet), p (pentet), m (multiplet), and br (broad). Coupling constant values are designated by *J* and are reported in Hertz. Integration of the products is provided. Analysis of products and starting materials by Gas Chromatography (GC) where indicated is performed using a Shimadzu GC-2010 Plus Gas Chromatograph or Shimadzu Nexis GC-2030 gas chromatograph with hydrogen as carrier gas with a flame ionization detector. The analysis by Gas Chromatography Mass Spectrometry (GC-MS) was performed on a Shimadzu GC-2010 Plus coupled with GC-MS QP2010SE or a a Shimadzu GC-2010 Plus Gas chromatograph equipped with Shimadzu GCMS-QP2010 SE mass spectrometer. The analytes is separated by way of a SHRXI-5MS- 30 m x 0.25 mm x 0.25 µm column using helium carrier gas; identification of the analyte is assisted by electron impact ionization. High Resolution-Mass Spectrometry (HR-MS) was performed in the Department of Chemistry at the University of Illinois at Urbana-Champaign and at the Mass Spec Facility at the University of Texas at Austin. All air-sensitive reactions involving the hydroamination reaction, unless otherwise indicated, were set-up with the aid of a glove box maintained under a nitrogen atmosphere.

Materials:
Solvents used for extraction and column chromatography were reagent grade and used as received. Reaction solvents tetrahydrofuran (Fisher, unstabilized HPLC ACS grade), diethyl ether (Fisher, BHT stabilized ACS grade), methylene chloride (Fisher, unstabilized HPLC grade), dimethoxyethane (Fisher, certified ACS), toluene (Fisher, optima ACS grade), 1,4-dioxane (Fisher, certified ACS), acetonitrile (Fisher, HPLC grade), and hexanes (Fisher, ACS HPLC grade) were dried on a Pure Process Technology Glass Contour Solvent Purification System using activated Stainless Steel columns while following manufacture's recommendations for solvent preparation and dispensation unless otherwise noted. were distilled and

degassed by the freeze-pump-thaw method before use. All liquid aldehydes and amines were freshly distilled prior to use.

B. Select Optimization

Table S1. Ligand screen

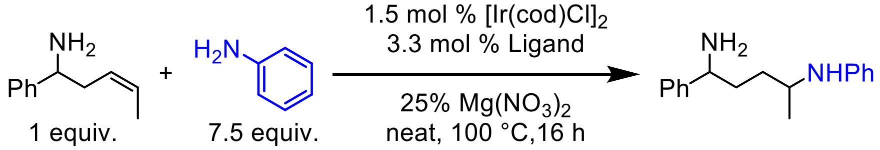

Ligand	GC yield (%)
Dppb	0
DPEphos	0
Xantphos	0
BINAP	78
Tol-BINAP	80
SEGPHOS	77
DTBM-SEGPHOS	17

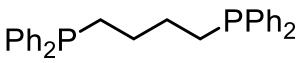

dppb

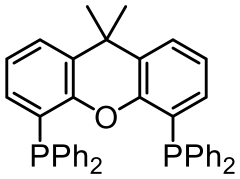

Xantphos

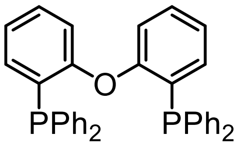

DPEphos

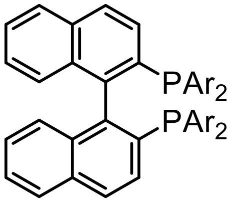

BINAP Ar = C_6H_5

***tol*-BINAP** Ar = 4-Me-C_6H_4

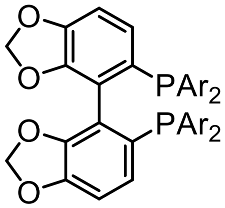

SEGPHOS Ar = C_6H_5

DTBM-SEGPHOS Ar = 3,5-tBu-4-MeO-C_6H_2

Table S2. Additive Screen

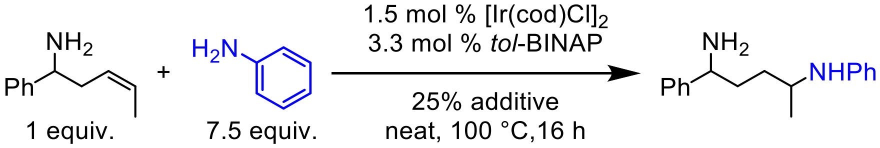

**Additive**	**GC yield (%)**
None	34
$LiNO_3$	74
$Mg(NO_3)_2$	80
$Mg(OTf)_2$	79
$MgCl_2$	8
$AgNO_3$	47
NaBArF	0

Table S3. Additive Screen for Cyclic Olefin

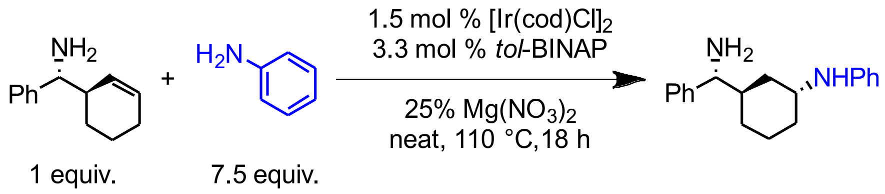

Additive	In-situ Yield	d.r.
KBr	20	0.46
CaI_2	27	1.22
$LiNO_3$	41	9.71
$MgSO_4$	21	1.39
$Ca(NO_3)_2$	27	2.99
KI	34	3.09
NaI	40	6.38
$CaBr_2$	18	1.10
**$Mg(NO_3)_2$**	47	**28.17**
CsI	34	4.59
Li_2SO_4	20	1.34
$MgBr_2$	21	1.60
**$ZnBr_2$**	50	**10.87**
K_2SO_4	20	1.44
ZnI_2	47	4.24
LiI	46	3.54

C. Substrate Synthesis

General Procedure A: Synthesis of linear internal olefins

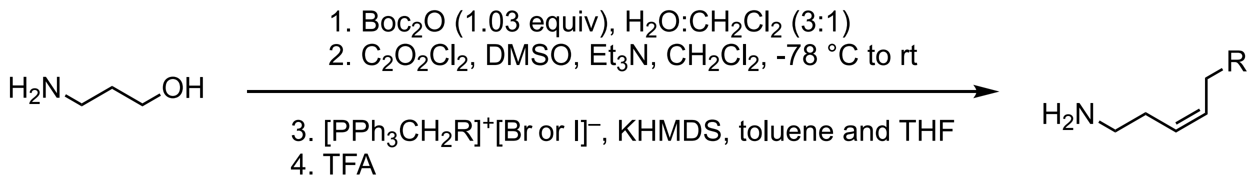

Step 1. 3-amino-1-propanol derivatives (1 equiv) were protected by adding di-*tert*-butyl-dicarbonate (1.03 equiv) to a biphasic mixture of deionized water (3 mL/mmol sm) and DCM (1 mL/mmol sm). This was stirred for 1-24 h, extracted twice with 30 mL dichloromethane, dried over brine and magnesium sulfate, and the solvent was removed *in vacuo*. These products were taken to the next step without further purification.

Step 2. The protected material was oxidized using a typical Swern oxidation condition. An oven-dried flask was placed under N_2 and 2 mL dichloromethane/mmol starting material was added to it. This was chilled to –78 °C and had 1.5 equiv oxalyl chloride added to it. 2.0 equiv dimethylsulfoxide (mixed 1:1 with dichloromethane to prevent freezing in the syringe) was added to this dropwise over five minutes. The solution was stirred for fifteen minutes at this temperature, and then a dichloromethane solution (2 mL/mmol) of the Boc amine was added to the solution over ten minutes. This was stirred for a further 30 minutes and 5 equiv trimethylamine was added to the reaction. The reaction was allowed to warm to room temperature overnight and quenched with 2 mL/mmol of saturated ammonium chloride solution and 1 mL/mmol of deionized water. The mixture was extracted twice with 40 mL dichloromethane, dried with sodium sulfate, and the solvent removed *in vacuo*. The aldehydes produced were mostly stable to column chromatography, however, generally, they were used without further purification. Note: purified yields ranged from 60-80% over 2 steps; purification was not found to improve yields for further reactions.

Step 3. The resulting aldehydes were then subjected to a *Z*-selective Wittig olefination. An oven-dried flask containing 2.2 equiv of the corresponding triphenylphosphonium bromide or iodide salt was cycled to a nitrogen atmosphere and chilled to 0 °C. To this was added 3 mL dry THF/mmol aldehyde, and 2.1 equiv KHMDS in toluene (0.7 M solution) slowly, producing bright red or orange suspensions. This was allowed to stir for 1-2 hours at this temperature. The aldehyde was dissolved in THF (2 mL/mmol) and added dropwise to the ylide solution. The reaction was allowed to warm up to rt overnight and quenched (allowing it to stir longer resulted in no further product formation, and in some cases degradation) using 2 mL/mmol starting material saturated ammonium chloride solution. The resulting suspension was extracted twice with 50 mL diethyl ether, loaded onto Celite, and columned using 25% ethyl acetate in hexanes. These columns generally did not remove all of the impurities, however, the goal was to remove most of the phosphine oxide and phosphine byproducts. These reactions consistently produced >20:1 *Z*-olefin, although with modest yields (30-50%).

Step 4. The Boc group was then removed to reveal the free internal Z-homoallylamine. This was accomplished by dissolving the starting olefin in 1.5 mL/mmol dichloromethane and chilling it to 0 °C. TFA was added (5 equiv) dropwise, usually inducing a stark color change. This was stirred at rt for 1.5 hours, and then chilled, 1.5 mL/mmol starting material deionized water added, 1.2 mL/mmol starting material 5 M NaOH added slowly, and a basic pH confirmed using pH test strips. This was extracted with 3 x 10 mL

dichloromethane, dried using sodium sulfate, and the solvent removed *in vacuo.* These products could be purified using distillationunder reduced pressure (0.2-1 torr) to afford the pure *Z*-homoallylamines in 25-70% yields.

General Procedure B: Synthesis of Cyclic Internal Olefins

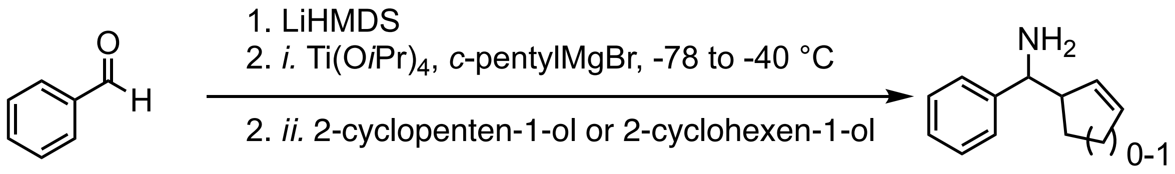

1.0 equiv of benzaldehyde was combined with 1.05 equiv LiHMDS in THF at 0 °C. This was warmed to room temperature overnight, concentrated by rotovap, and distilled (55 °C, 0.4 torr) to afford benzaldehyde trimethylsilyl imine in 61% yield.

The trimethylsilyl imine was put under a nitrogen atmosphere and dissolved in 8 mL/mmol diethyl ether. Then, 1.5 equiv titanium isopropoxide (neat) was added in a slow dropwise fashion. The reaction was stirred for 5 minutes, and chilled to -78 °C. To this was added slowly 3 equiv cyclopentylmagnesium chloride in THF. The mixture was stirred for 10 minutes and then warmed to a -40 °C where it stirred for an additional 1 hour. This solution was chilled to -78 °C, and then 1.3 equiv of the corresponding lithium alkoxide of the allylic alcohol (2-cyclopenten-1-ol or 2-cyclohexen-1-ol) was added (in 5 mL/mmol alkoxide of THF). This was allowed to warm to rt and stir over for two days. The reaction was quenched with 5 mL/mmol saturated sodium bicarbonate solution, stirred vigorously for 1 h, and filtered through a pad of Celite using sodium bicarbonate and diethyl ether to rinse. This was extracted with 3 x 50 mL portions of diethyl ether, dried over sodium sulfate, and concentrated *in vacuo*. The free amine was produced in > 20:1 d.r. and distilled under reduced pressure to afford the pure product. Yields were 50-65%.

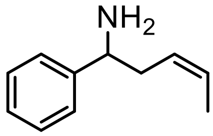

1a

General Procedure A

E:*Z* ratio 1:5

^{1}H NMR (500 MHz, $CDCl_3$): δ 7.34 (qd, *J* = 8.3, 4.0 Hz, 4H), 7.26 – 7.21 (m, 1H), 5.58 (dddd, *J* = 10.9, 8.4, 6.8, 5.2 Hz, 1H), 5.38 (dddt, *J* = 11.4, 8.3, 6.6, 1.8 Hz, 1H), 3.97 (dd, *J* = 7.7, 5.9 Hz, 1H), 2.49 – 2.35 (m, 2H), 1.60 (dd, *J* = 6.8, 1.8 Hz, 3H) ppm.

^{13}C NMR (126 MHz, $CDCl_3$): δ 146.46, 128.70, 127.25, 127.23, 127.00, 126.65jj, 56.34, 37.51, 13.34 ppm.

HR-MS (ESI-TOF) *m/z*: $[M+H]^+$ calculated for $C_{11}H_{16}N^+$, 162.1283; found, 162.1385.

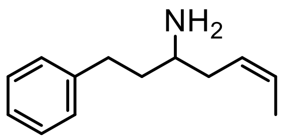

1b

General Procedure A

E:*Z* < 1:10

^{1}H NMR (500 MHz, $CDCl_3$): δ 7.28 (t, *J* = 7.5 Hz, 2H), 7.22 – 7.14 (m, 3H), 5.61 (dtdd, *J* = 9.4, 6.8, 6.0, 1.5 Hz, 1H), 5.41 (dddt, *J* = 11.4, 8.5, 5.0, 1.8 Hz, 1H), 2.82 (ddd, *J* = 10.0, 8.0, 5.1 Hz, 1H), 2.80 – 2.72 (m, 1H), 2.65 (ddd, *J* = 13.7, 10.2, 6.1 Hz, 1H), 2.20 (dt, *J* = 13.4, 6.2 Hz, 1H), 2.11 (dt, *J* = 14.7, 7.9 Hz, 1H), 1.78 (dddd, *J* = 15.1, 7.2, 6.1, 4.9 Hz, 1H), 1.82-1.75 (m, 1H), 1.64 (dq, *J* = 7.6, 1.1 Hz, 3H), 1.41 (s, 2H) ppm.

^{13}C NMR (126 MHz, $CDCl_3$): δ 142.66, 128.71, 128.70, 127.49, 126.82, 126.10, 51.38, 39.72, 35.82, 33.09, 13.44.

HR-MS (ESI-TOF) *m/z*: $[M+H]^+$ jcalculated for $C_{13}H_{20}N^+$, 190.1596; found, 190.1597.

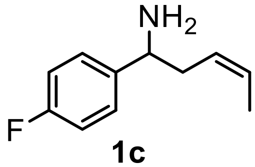

1c

General Procedure A

E:*Z* = 6:1

^{1}H NMR (400 MHz, $CDCl_3$): δ 7.31 (dd, *J* = 8.7, 5.5 Hz, 2H), 7.00 (t, *J* = 8.8 Hz, 2H), 5.64 – 5.46 (m, 1H), 5.40 – 5.24 (m, 1H), 3.96 (dd, *J* = 6.0, 5.8 Hz, 1H), 2.37 (qt, *J* = 13.4, 6.4 Hz, 2H), 1.64 (s, 2H), 1.61 – 1.51 (m, 3H) ppm.

^{13}C NMR (101 MHz, $CDCl_3$): δ 161.92 (d, J^{CF} = 244.5 Hz), 141.73 (d, J^{CF} = 3.3 Hz), 127.97 (d, J^{CF} = 8.0 Hz), 127.01, 126.71, 115.18 (d, J^{CF} = 21.1 Hz), 55.45, 37.37, 13.10 ppm.

^{19}F NMR (376 MHz, $CDCl_3$): δ -119.38 (tt, *J* = 8.2, 5.1 Hz) ppm.

HR-MS (ESI-TOF) *m/z*: $[M+H]^+$ calculated for $C_{11}H_{15}FN^+$, 180.2459; found, 180.1190.

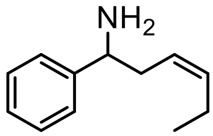

1d

General Procedure A

E:*Z* = 1:5

^{1}H NMR (500 MHz, $CDCl_3$): δ 7.36 – 7.31 (m, 4H), 7.26 – 7.21 (m, 1H), 5.53 – 5.44 (m, 1H), 5.35 – 5.28 (m, 1H), 3.96 (dd, *J* = 7.6, 6.0 Hz, 1H), 2.45 – 2.36 (m, 2H), 2.03 (dddd, *J* = 14.6, 7.3, 5.5, 3.7 Hz, 2H), 1.52 (s, 2H), 0.92 (t, *J* = 7.5 Hz, 3H) ppm.

^{13}C NMR (126 MHz, $CDCl_3$): δ 146.26, 134.57, 128.48, 127.03, 126.46, 125.45, 56.22, 37.61, 20.86, 14.35.

HR-MS (ESI-TOF) *m/z*: $[M+H]^+$ calculated for $C_{12}H_{18}N$, 176.1439; found,z 176.1440.

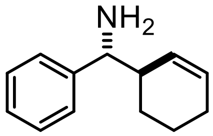

1e

General Procedure B

d.r. > 20:1

^{1}H NMR (500 MHz, $CDCl_3$): δ 7.38 – 7.30 (m, 4H), 7.29 – 7.23 (m, 1H), 5.85 (bs, 2H), 3.77 (d, *J* = 7.3 Hz, 1H), 2.38 (dddd, *J* = 10.8, 8.4, 5.7, 2.2 Hz, 1H), 2.00 (ddt, *J* = 8.6, 4.7, 2.1 Hz, 2H), 1.72 (dtd, *J* = 13.9, 6.7, 5.6, 3.9 Hz, 1H), 1.51 (tdd, *J* = 10.1, 7.0, 3.1 Hz, 4H), 1.35 – 1.25 (m, 1H) ppm.

^{13}C NMR (126 MHz, $CDCl_3$): δ 145.82, 129.82, 128.58, 128.10, 127.13, 127.09, 60.70, 43.35, 27.23, 25.67, 21.83 ppm.

HR-MS (ESI-TOF) *m/z*: $[M+H]^+$ calculated for $C_{13}H_{18}N^+$, 188.1439; found, 188.1445.

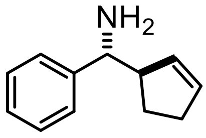

1f

General Procedure B

d.r. ~ 14:1

^{1}H NMR (500 MHz, $CDCl_3$): δ 7.35 – 7.30 (m, 4H), 7.24 (dtd, *J* = 8.5, 4.4, 2.2 Hz, 1H), 5.88 (dq, *J* = 5.5, 2.2 Hz, 1H), 5.82 – 5.78 (m, 1H), 3.75 (dd, *J* = 7.3, 1.1 Hz, 1H), 3.05 – 2.97 (m, 1H), 2.38 – 2.19 (m, 2H), 1.87 – 1.75 (m, 1H), 1.55 (dddd, *J* = 13.2, 9.1, 6.5, 5.3 Hz, 1H), 1.50 (bs, 2H) ppm.

^{13}C NMR (126 MHz, $CDCl_3$): δ 146.15, 133.51, 131.63, 128.59, 127.12, 127.08, 60.89, 54.11, 32.52, 27.55 ppm.

HR-MS (ESI-TOF) *m/z*: $[M+H]^+$ calculated for $C_{12}H_{16}N$, 174.1283; found, 174.1277.

D. General Hydroamination Procedure and Product Characterization

1,4- Selective General Procedure C for internal olefins

To a 4 mL vial equipped with a stirbar was added $[Ir(cod)Cl]_2$ (2.01 mg, 1.5 mol %), *tol*-BINAP (4.48 mg, 3.3 mol %), $Mg(NO_3)_2$ (7.4 mg, 0.05 mmol, 0.25 equiv), homoallylic amine (0.2 mmol, 1.0 equiv) and aryl amine (1.5 mmol, 7.5 equiv). The 4 mL vial was sealed with a Teflon cap, removed from nitrogen-filled glove box, and heated to 100 °C for 16 h while stirring. The crude reaction was purified directly by column chromatography (eluent: ramp from 97% DCM:3% NH_4OH: 0% MeOH to 92% DCM:3% NH_4OH:5% MeOH).

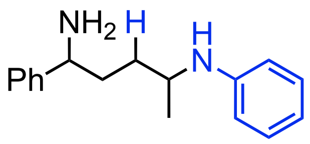

3
60% yield
2.0:1 dr

***N*4,1-diphenylpentane-1,4-diamine (3)**
The product was synthesized according to General Procedure C. This product (60%, 0.12 mmol) was isolated as a 2:1 mixture of diastereomers, with > 20:1 selectivity over the 1,3 and 1,5 diamine product as a 2:1 mixture of diastereoisomers.

^{1}H NMR (500 MHz, $CDCl_3$): δ 7.40 – 7.30 (m, 4H), 7.29 – 7.23 (m, 1H), 7.16 (t, *J* = 7.8 Hz, 2H), 6.68 (td, *J* = 7.4, 1.4 Hz, 1H), 6.55 (d, *J* = 8.1 Hz, 2H), 3.90 (t, *J* = 6.9 Hz, 1H), 3.51 – 3.42 (m, 1H), 1.91 – 1.73 (m, 2H), 1.71 – 1.61 (m, 1H, *major*), 1.59 – 1.52 (m, 0.5 H, *minor*), 1.51 – 1.43 (m, 0.5 H, *minor*), 1.42-1.31 (m, 1H, *major*) 1.17 (d, *J* = 6.4, 2.2 Hz, 3H) ppm.

^{13}C NMR (126 MHz, $CDCl_3$): δ 147.86, 146.62, 129.59, 128.86, 127.35, 126.62, 117.18, 113.40, 56.71, 48.83, 36.33, 34.33, 21.16 ppm.

HR-MS (ESI-TOF) *m/z*: $[M+H]^+$ calculated for $C_{17}H_{23}N_2$, 255.1861; found, 255.1864.

The diastereoisomeric ratio was determined by ^{1}H NMR; one of the CH_2 groups separate.

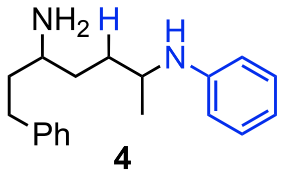

4
74% yield
2:1 dr

N2,7-diphenylheptane-2,5-diamine (4)

The product was synthesized according to General Procedure C. This product (74%, 0.15 mmol) was isolated at 90 °C as a 2:1 mixture of diastereomers (approximated by ^{13}C NMR, no peaks resolved in the ^{1}H NMR), with > 20:1 selectivity over the 1,3 and 1,5 diamine product.

^{1}H NMR (500 MHz, $CDCl_3$): δ 7.33 – 7.29 (m, 2H), 7.24 – 7.15 (m, 5H), 6.69 (t, *J* = 7.2 Hz, 1H), 6.59 (d, *J* = 8.0 Hz, 2H), 3.48 (h, *J* = 6.1 Hz, 1H), 2.86 – 2.72 (m, 3H), 2.66 (dddd, *J* = 13.5, 9.6, 6.3, 2.7 Hz, 2H), 1.87 – 1.75 (m, 2H), 1.73 – 1.41 (m, 6H), 1.21 (d, *J* = 6.4 Hz, 3H) ppm.

^{13}C NMR (126 MHz, $CDCl_3$): δ 147.93, 147.91 (minor), 142.48, 129.65, 128.74, 128.68, 126.15, 117.27 (minor), 117.24, 113.46 (minor), 113.44, 51.30, 51.28 (minor),49.03, 48.95 (minor), 40.08, 34.61, 33.89, 32.89, 32.85 (minor), 21.31, 21.24 (minor) ppm.

HR-MS (ESI-TOF) *m/z*: $[M+H]^+$ calculated for $C_{19}H_{27}N_2$, 283.2174; found mass, 283.2163.

The only two signals that showed baseline separation are shown below and support a d.r. of approximately 2:1.

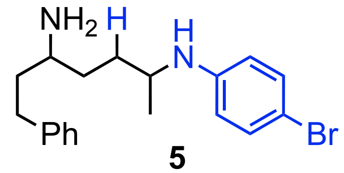

5
50% yield
1.5:1 dr

N2-(4-bromophenyl)-7-phenylheptane-2,5-diamine (5)

The product was synthesized according to General Procedure C. This product (50%, 0.10 mmol) was isolated as a 1.5:1 mixture of diastereomers (approximated by ^{13}C NMR; there were no separate peaks in the ^{1}H NMR), with > 20:1 selectivity over the 1,3 and 1,5 diamine product.

^{1}H NMR (500 MHz, $CDCl_3$): δ 7.31 – 7.25 (m, 2H), 7.24 – 7.15 (m, 5H), 6.43 (d, *J* = 8.8 Hz, 1H), 3.40 (hept, *J* = 7.2, 6.7 Hz, 1H), 2.73 (dtd, *J* = 16.5, 8.7, 7.6, 3.4 Hz, 2H), 2.62 (ddd, *J* = 13.6, 10.0, 6.3 Hz, 1H), 1.80 – 1.70 (m, 1H), 1.67 – 1.31 (m, 5H), 1.17 (dd, *J* = 6.3, 1.2 Hz, 3H) ppm.

^{13}C NMR (126 MHz, $CDCl_3$): δ 146.70, 146.68 (minor), 142.24, 132.07, 128.53, 128.45, 125.94, 114.73 (minor), 114.71, 108.37 (minor), 108.35 (major), 51.02 (major), 50.99 (minor), 48.93 (minor), 48.84 (major), 40.07 (minor), 40.02 (major), 34.46 (minor), 34.40 (major), 33.52 (major), 33.45 (minor), 32.69 (minor), 32.64 (major), 20.90 (major), 20.84 (minor) ppm.

HR-MS (ESI-TOF) *m/z*: $[M+H]^+$ calculated for $C_{19}H_{26}N_2Br$, 361.1279; found, 361.1274.

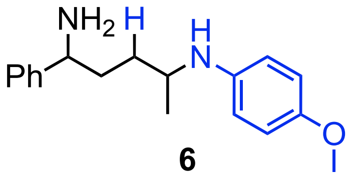

6
49% yield
1.5:1 dr

N4-(4-methoxyphenyl)-1-phenylpentane-1,4-diamine (6)
The product was synthesized according to General Procedure C. This product (49%, 0.098 mmol) was isolated as a 1.5:1 mixture of diastereomers, with > 20:1 selectivity over the 1,3 and 1,5 diamine product.

^{1}H NMR (500 MHz, $CDCl_3$): δ 7.31 (m, 4H), 7.26 – 7.20 (m, 1H), 6.75 (d, *J* = 8.9 Hz, 2), 6.50 (d, *J* = 8.9 Hz, 2H), 3.88 (t, *J* = 6.9 Hz, 1H), 3.74 (s, 3H), 3.39 – 3.30 (m, 1H), 2.27 (br s, 2H), 1.60 (ddd, *J* = 17.2, 11.1, 5.6 Hz, 0.6H, 1H major), 1.55 – 1.37 (m, 0.9H, 2H minor), 1.36 – 1.27 (m, 1H, major), 1.2 (overlapping d, *J* = 6.4 Hz, 3H, major and minor) ppm.

δ ^{13}C NMR (126 MHz, $CDCl_3$): δ 151.96, 146.27 (minor), 146.19 (major), 141.88, 128.65, 127.17, 126.44, 115.07, 114.82 (major), 114.79 (minor), 56.50 (major), 56.46 (minor) 55.96, 49.70 (minor), 49.68 (major), 36.00 (major), 35.98 (minor) 34.11 (major), 34.04 (minor), 20.99 (minor), 20.97 (major) ppm.

HR-MS (ESI-TOF) *m/z*: $[M+H]^+$ calculated for $C_{18}H_{25}N_2O$, 285.1967; found, 285.1965.

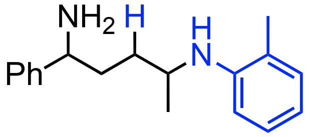

7
29% yield
1.6:1 dr

1-phenyl-N4-(o-tolyl)pentane-1,4-diamine (7)
The product was synthesized according to General Procedure C. This product (29%, 0.058 mmol) was isolated as a 1.6:1 mixture of diastereomers, with > 20:1 selectivity over the 1,3 and 1,5 diamine product.

^{1}H NMR (500 MHz, $CDCl_3$): δ 7.40 – 7.30 (m, 4H), 7.28 – 7.25 (m, 1H), 7.10 (t, *J* = 7.7 Hz, 1H), 7.05 (d, *J* = 7.3 Hz, 1H), 6.63 (t, *J* = 7.5 Hz, 1H), 6.55 (d, *J* = 8.2 Hz, 1H), 3.97 – 3.89 (m, 1H), 3.57 – 3.47 (m, 1H), 2.73 (bs, 3H), 2.11 (s, 3H), 1.92 – 1.75 (m, 2H), 1.73 – 1.63 (m, 1H, major), 1.63 – 1.44 (m, 1H, minor 2H), 1.41 – 1.33 (m, 1H, major), 1.19 (d, *J* = 6.3, Hz, 3H) ppm.

^{13}C NMR (126 MHz, $CDCl_3$): δ 145.47, 130.37, 128.76, 127.38, 127.23, 126.52, 121.83, 121.82, 116.47, 110.19 (major), 110.13 (minor), 56.45 (major), 56.42 (minor), 48.43 (minor), 48.41 (major), 35.66 (major), 35.61 (minor), 34.09 (major), 34.00 (minor), 21.17 (minor), 21.12 (major), 17.75 (major), 17.73 (minor) ppm.

HR-MS (ESI-TOF) *m/z*: $[M+H]^+$ calculated for $C_{18}H_{25}N_2$, 269.2018; found, 269.2017

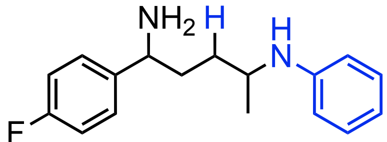

8
60% yield
1.7:1 dr

1-(4-fluorophenyl)-N4-phenylpentane-1,4-diamine (8)
The product was synthesized according to General Procedure C. This product (60%, 0.12 mmol) was isolated as a 1.7:1 mixture of diastereomers, with > 20:1 selectivity over the 1,3 and 1,5 diamine product.

^{1}H NMR (400 MHz, $CDCl_3$): δ 7.33 – 7.20 (m, 2H), 7.14 (dd, *J* = 8.5, 7.2 Hz, 2H), 7.00 (td, *J* = 8.6, 6.2 Hz, 2H), 6.66 (td, *J* = 7.3, 1.0 Hz, 1H), 6.53 (d, *J* = 7.4 Hz, 2H), 3.89 (t, *J* = 6.8 Hz, 1H), 3.43 (h, *J* = 6.3 Hz, 1H), 2.67 (bs, 2H), 1.91 – 1.64 (m, 2H), 1.64 – 1.53 (m, 1H, major), 1.52-1.36 (m, 2H, minor)), 1.36-1.22 (m, 1H, major) 1.14 (d, *J* = 6.3 Hz, 3H, minor) 1.13 (d, *J* = 6.3 Hz, 3H, major) ppm.

^{13}C NMR (101 MHz, $CDCl_3$): δ 162.01 (d, *J* = 245.0 Hz) (overlapping peaks), 147.56 (overlapping peaks), 141.17 (overlapping peaks), 129.40 (overlapping peaks), 128.07 (d, *J* = 7.9 Hz) (overlapping peaks), 117.06 (overlapping peaks), 115.43 (d, *J* = 21.3 Hz) (overlapping peaks), 113.21 (major), 113.20 (minor), 55.77 (major), 55.74 (minor), 48.57 (minor), 48.50 (major), 35.78 (overlapping peaks), 33.88 (major), 33.73 (minor), 20.94 (minor), 20.93 (major).

^{19}F NMR (376 MHz, $CDCl_3$): δ -118.43 (overlapping peaks) ppm.

HR-MS (ESI-TOF) *m/z*: $[M+H]^+$ calculated for $C_{17}H_{21}FN_2$, 273.1762; found, 273.1759.

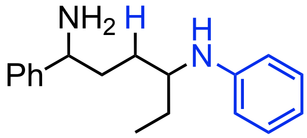

9
25% yield
2.5:1 dr

***N*4,1-diphenylhexane-1,4-diamine (9)**
The product was synthesized according to General Procedure C. This product (25%, 0.050 mmol) was isolated as a 2.2:1 mixture of diastereomers, with > 20:1 selectivity over the 1,3 and 1,5 diamine product.

^{1}H NMR (500 MHz, $CDCl_3$): δ 7.38 – 7.24 (m, 5H), 7.15 (t, *J* = 7.8 Hz, 2H), 6.65 (t, *J* = 7.3 Hz, 1H), 6.54 (d, *J* = 8.0 Hz, 2H), 3.90 (t, *J* = 7.1 Hz, 1H), 3.28 (p, *J* = 6.1 Hz, 1H), 2.84 (br s, 3H), 1.90 – 1.69 (m, 2H), 1.68 – 1.29 (m, 4H), 0.89 (t, *J* = 7.5 Hz, 3H, major), 0.88 (t, *J* = 7.5 Hz, 3H, minor).

^{13}C NMR (126 MHz, $CDCl_3$): δ 148.14 (major), 148.13 (minor), 145.50 (overlapping peaks), 129.40 (overlapping peaks), 128.72(overlapping peaks), 127.36 (minor), 127.33 (major), 126.54 (minor), 126.48 (major), 116.78 (minor), 116.76 (major), 113.05 (major), 113.03 (minor), 56.46 (minor), 54.21 (minor),

54.18 (major), 35.51 (major), 35.34 (minor), 31.32 (major), 31.04 (minor, 27.49 (minor), 27.47 (major), 10.22 (minor), 10.13 (major).

HR-MS (ESI-TOF) *m/z*: $[M+H]^+$ calculated for $C_{18}H_{25}N_2^+$, 269.2012; found mass, 269.2019

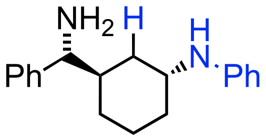

10
50% yield
>20:1 dr

(+/-) *N*-((trans)-3-(famino(phenyl)methyl)cyclohexyl)aniline (10)
This product (50%, 0.10 mmol) was isolated as a mixture of diastereomers with predominantly the shown diastereomer (>20:1), with > 20:1 selectivity over the 1,3 and 1,5 diamine product.

^{1}H NMR (500 MHz, $CDCl_3$): δ 7.40 – 7.32 (m, 2H), 7.32 – 7.24 (m, 3H), 7.19 (t, *J* = 7.4 Hz, 2H), 6.69 (t, *J* = 7.3 Hz, 1H), 6.64 (d, *J* = 7.3 Hz, 2H), 3.82 – 3.76 (m, 1H), 3.69 (d, *J* = 7.9 Hz, 1H), 2.10 (d, *J* = 13.3 Hz, 1H), 1.85 (tdt, *J* = 11.3, 7.6, 3.6 Hz, 1H), 1.81 – 1.74 (m, 1H), 1.73 – 1.40 (m, 7H), 1.01 – 0.89 (m, 1H) ppm.

^{13}C NMR (126 MHz, $CDCl_3$): δ 147.35, 145.02, 129.30, 128.31, 127.10, 127.04, 116.83, 113.03, 60.81, 47.50, 39.78, 33.99, 30.19, 28.89, 20.43 ppm.

HR-MS (ESI-TOF) *m/z*: $[M+H]^+$ calculated for $C_{19}H_{25}N_2^+$, 281.2012; found mass, 281.2018

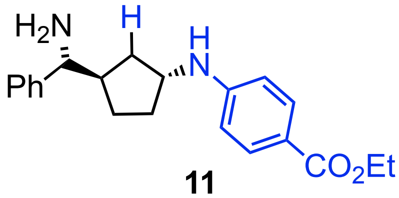

11
41% yield
12.0:1 dr

ethyl 4-(((1R,3R)-3-((R)-amino(phenyl)methyl)cyclopentyl)amino)benzoate (11)
This product (41%, 0.082 mmol) was isolated as a mixture of diastereomers with predominantly the shown diastereomer (12:1 selectivity), with > 20:1 selectivity over the 1,3 and 1,5 diamine product. (note: initial d.r. of substrate is 14:1)

^{1}H NMR (500 MHz, $CDCl_3$): δ 7.87 (d, *J* = 8.7 Hz, 2H), 7.39 – 7.30 (m, 4H), 7.30 – 7.23 (m, 1H), 6.54 (d, *J* = 8.7 Hz, 2H), 4.33 (q, *J* = 7.1 Hz, 2H), 3.94 (m, 1H), 3.71 (d, *J* = 8.9 Hz, 1H), 2.38 (h, *J* = 9.5 Hz, 1H), 2.21 – 1.86 (m, 5H), 1.53 (dtd, *J* = 10.6, 7.2, 3.1, 1H), 1.44 (ddt, *J* = 13.4, 8.0, 4.2 Hz, 1H), 1.37 (t, *J* = 7.2 Hz, 3H), 1.31 – 1.23 (m, 1H).

^{13}C NMR (126 MHz, $CDCl_3$): δ 167.22, 151.61, 145.56, 131.78, 128.80, 127.55, 127.08, 118.72, 112.11, 61.73, 60.47, 54.09, 46.61, 37.92, 33.75, 29.10, 14.80.

HR-MS (ESI-TOF) *m/z*: $[M+H]^+$ calculated for $C_{21}H_{27}N_2O_2$, 339.2073; found mass, 339.2071

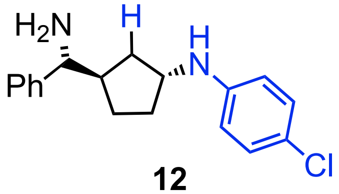

12
51% yield
13:1 dr

N-(3-(amino(phenyl)methyl)cyclopentyl)-4-chloroaniline (12)
This product (51%, 0.10 mmol) was isolated as a mixture of diastereomers with predominantly the shown diastereomer (13:1 selectivity), with > 20:1 selectivity over the 1,3 and 1,5 diamine product (note: initial d.r. of substrate is 14:1).

^{1}H NMR (500 MHz, $CDCl_3$): δ 7.40 – 7.31 (m, 3H), 7.30 – 7.23 (m, 2H), 7.12 (d, *J* = 8.8 Hz, 2H), 6.51 (d, *J* = 8.8 Hz, 2H), 3.84 (qd, *J* = 6.7, 3.7 Hz, 1H), 3.69 (d, *J* = 8.9 Hz, 1H), 2.72 – 2.29 (bs, 3H), 2.37 (h, *J* = 9.5 Hz, 1H), 2.13 (dddd, *J* = 12.7, 8.8, 6.7, 3.6 Hz, 1H), 1.95 – 1.80 (m, 2H), 1.51 (dtd, *J* = 11.1, 7.4, 3.4 Hz, 1H), 1.41 (dtd, *J* = 13.2, 8.2, 5.5 Hz, 1H), 1.31 – 1.18 (m, 1H).

^{13}C NMR (126 MHz, $CDCl_3$): δ 146.59, 145.64, 129.31, 128.78, 127.51, 127.09, 121.90, 114.51, 61.80, 54.58, 46.60, 37.91, 33.78, 29.17.

HR-MS (ESI-TOF) *m/z*: $[M+H]^+$ calculated for $C_{18}H_{22}N_2Cl^+$, 301.1466; found mass, 301.1465

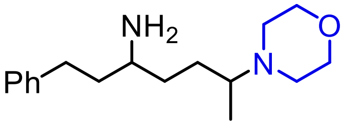

17
65% yield
1.1:1 dr

6-morpholino-1-phenylheptan-3-amine (17)
The product was synthesized according to General Procedure C. This product (65%, 0.13 mmol) was isolated as a 1.1:1 mixture of diastereomers, with > 20:1 selectivity over the 1,3 and 1,5 diamine product.

^{1}H NMR (500 MHz, $CDCl_3$): δ 7.31 – 7.26 (m, 2H), 7.19 (d, *J* = 7.8 Hz, 3H), 3.77 – 3.64 (m, 4H), 2.78-2.71 (m, 2H), 2.69 – 2.58 (m, 1H), 2.57-2.51 (m, 2H), 2.51 – 2.40 (m, 3H), 2.15 – 2.01 (bs, 2H), 1.76 (m, 1H), 1.69 –1.48 (m, 2H), 1.45 – 1.25 (m, 3H), 0.98 (dd, *J* = 6.6, 1.7 Hz, 3H) ppm. *Note. This was isolated as a 1.1:1 mixture of diastereoisomers; the overlapping signals complicated analysis.*

^{13}C NMR (126 MHz, $CDCl_3$): δ 142.35 (overlapping peaks), 128.53 (overlapping peaks), 128.48 (overlapping peaks), 125.94 (overlapping peaks), 67.54, 67.52, 59.64, 59.61, 51.24, 51.18, 49.00, 48.95, 39.87, 39.80, 35.09, 35.03, 32.75, 32.72, 30.04, 30.00, 14.30 (overlapping peaks) ppm. *Note. This was isolated as a 1.1:1*

mixture of diastereoisomers; the carbon signals typically come in pairs. We were unable to definitively assign major/minor isomers.

HR-MS (ESI-TOF) *m/z*: $[M+H]^+$ calculated for $C_{17}H_{29}N_2O^+$, 277.2274; found mass, 277.2267.

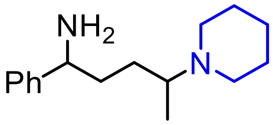

18
52% yield
1:1 dr

1-phenyl-4-(piperidin-1-yl)pentan-1-amine (18)
The product was synthesized according to General Procedure C. This product (52%, 0.13 mmol) was isolated as a 5:1 mixture of diastereomers, with > 20:1 selectivity over the 1,3 and 1,5 diamine product.

^{1}H NMR (400 MHz, $CDCl_3$): δ 7.34 – 7.30 (m, 4H), 7.25 – 7.21 (m, 1H), 3.85 (t, *J* = 6.7 Hz, 1H), 2.56 (m, 1H), 2.43 (m, 4H), 2.01 (s, 2H), 1.77 – 1.52 (m, 7H), 1.41 (q, *J* = 5.9 Hz, 2H), 1.22 – 1.10 (m, 1H), 0.93 (d, *J* = 6.6 Hz, 3H).

^{13}C NMR (101 MHz, $CDCl_3$): δ 146.81, 128.59, 127.02, 126.45, 59.93, 56.73, 54.74, 49.45, 37.18, 30.52, 29.83, 26.36, 25.99, 24.93, 14.06, 13.97. *Note. This was isolated as a 5:1 mixture of diastereoisomers, the signals for the major isomer are provided.*

HR-MS (ESI-TOF) *m/z*: $[M+H]^+$ calculated for $C_{16}H_{27}N_2^+$, 247.2169; found mass, 247.2176.

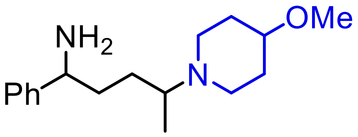

19
50% yield
1:1 dr

4-(4-methoxypiperidin-1-yl)-1-phenylpentan-1-amine (19)
The product was synthesized according to General Procedure C. This product (50%, 0.1 mmol) was isolated as a 1.4:1 mixture of diastereomers, with > 20:1 selectivity over the 1,3 and 1,5 diamine product.

^{1}H NMR (500 MHz, $CDCl_3$): δ 7.36 – 7.26 (m, 4H), 7.26 – 7.20 (m, 1H), 3.90 – 3.82 (m, 1H), 3.31 (s, 3H), 3.24 – 3.16 (m, 1H), 2.74 – 2.59 (m, 3H), 2.38 (t, *J* = 9.1 Hz, 1H), 2.32 – 2.12 (m, 3H), 1.94 (bs, 2H), 1.71 (m, 2H), 1.58 (m, 2H), 1.39 – 1.33 (m, 1H (0.47H, minor)), 1.22 – 1.11 (m, 1H (0.75H, major)), 0.96 (m, 3H).

^{13}C NMR (101 MHz, $CDCl_3$): δ 146.65 (minor), 146.55 (major), 128.60 (overlapling peaks), 127.07 (major), 127.03 (minor), 126.42 (major), 126.38 (minor), 59.38 (overlapping peaks), 56.62 (major), 56.43 (minor),

55.63 (overlapping peaks), 44.81 (major), 44.74 (minor), 36.93 (major), 36.84 (minor), 31.04 (broad, overlapping peaks), 30.95 (broad, overlapping peaks), 30.62 (major), 30.43 (minor), 29.81 (minor), 14.11 (major), 14.03 (minor).

HR-MS (ESI-TOF) *m/z*: $[M+H]^+$ calculated for $C_{17}H_{28}N_2O^+$, 277.2274; found mass, 277.2277

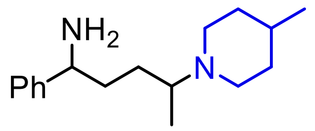

20
51% yield
1.1:1 dr

4-(4-methylpiperidin-1-yl)-1-phenylpentan-1-amine (20)
The product was synthesized according to General Procedure C. This product (51%, 0.11 mmol) was isolated as a 1.1:1 mixture of diastereomers, with > 20:1 selectivity over the 1,3 and 1,5 diamine product.

^1H NMR (500 MHz, $CDCl_3$): δ 7.34 – 7.27 (m, 4H), 7.21 (d, *J* = 17.1 Hz, 1H), 3.84 (m, 1H), 2.72 – 2.61 (m, 2H), 2.54 (m, 1H), 2.23 (m, 1H), 2.11-1.90 (m, 2H), 1.74 – 1.54 (m, 5H), 1.52 – 1.06 (m, 5H), 0.92 (t, *J* = 6.7 Hz, 3H), 0.88 (d, *J* = 6.3 Hz, 3H). *Note. This was isolated as a 1.1:1 mixture of diastereoisomers; the overlapping signals complicated analysis.*

^{13}C NMR (126 MHz, $CDCl_3$): δ 146.76, 146.67, 128.51 (overlapping signals), 126.95, 126.92, 126.39, 126.35, 59.48, 59.43, 56.67, 56.49, 50.60, 50.51, 46.92, 46.82, 37.11, 37.06, 34.80, 34.74, 34.62, 34.57, 31.27, 31.24, 30.60, 30.59, 22.04 (overlapping signals), 14.09, 14.00. *Note. This was isolated as a 1.1:1 mixture of diastereoisomers; the carbon signals typically come in pairs. We were unable to definitively assign major/minor isomers.*

HR-MS (ESI-TOF) *m/z*: $[M+H]^+$ calculated for $C_{17}H_{29}N_2^+$, 261.2325; found mass, 261.2326

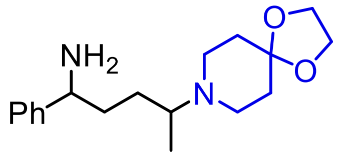

21
39% yield
1:1 dr

1-phenyl-4-(1,4-dioxa-8-azaspiro[4.5]decan-8-yl)pentan-1-amine (21)
The product was synthesized according to General Procedure C. This product (39%, 0.079 mmol) was isolated as a 1.1:1 mixture of diastereomers, with > 20:1 selectivity over the 1,3 and 1,5 diamine product.

^1H NMR (400 MHz, $CDCl_3$): δ 7.33 – 7.28 (m, 4H), 7.22 (m, 1H), 3.92 (m, 4H), 3.84 (m, 1H), 2.93 (m, 1H), 2.66 – 2.50 (m, 4H), 2.50 – 2.39 (m, 2H), 1.90 (bs, 2H), 1.80 – 1.63 (m, 4H), 1.62-1.51 (m, 1H(0.7H)), 1.46 – 1.29 (m, 2H (1.1H)), 1.22 – 1.09 (m, 1H (0.6H)), 0.92 (d, J = 6.6 Hz, 3H (1.5H), 0.91 (d, J = 6.6 Hz, 3H (1.5H)). *Note. This was isolated as a 1.1:1 mixture of diastereoisomers; the overlapping signals complicated analysis.*

^{13}C NMR (101 MHz, $CDCl_3$): δ 146.66, 146.57, 128.61 (overlapping signals), 127.07, 127.05, 126.43, 126.40, 107.66, 107.61, 64.29 (overlapping signals), 59.09, 59.05, 56.65, 56.49, 46.14 (overlapping signals), 37.00, 36.90, 35.34, 35.30, 30.83, 30.80, 14.26, 14.12. *Note. This was isolated as a 1.1:1 mixture of diastereoisomers; the carbon signals typically come in pairs. We were unable to definitively assign major/minor isomers.*

HR-MS (ESI-TOF) *m/z*: $[M+H]^+$ calculated for $C_{18}H_{29}N_2O_2^+$, 305.2224; found mass, 305.2230.

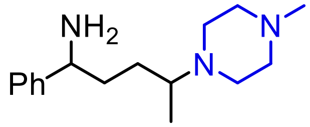

22
40% yield
1.2:1 dr

4-(4-methylpiperazin-1-yl)-1-phenylpentan-1-amine (22)
The product was synthesized according to General Procedure C. This product (40%, 0.8 mmol) was isolated at 100 °C as a 1.2:1 mixture of diastereomers, with > 20:1 selectivity over the 1,3 and 1,5 diamine products.

^1H NMR (500 MHz, $CDCl_3$): δ 7.30 (m, 5H), 7.23 (m, 1H), 3.84 (m, 1H), 2.55-2.35 (m, 9zH), 2.25 (s, 3H), 1.71 (m, 2H), 1.62-1.52 (m, 1H, major (0.55H)), 1.45 – 1.36 (m, 1H, minor (0.45H)), 1.31 (ddt, J = 13.3, 8.9, 7.0 Hz, 1H, minor), 1.23 (s, 1H (NH)), 1.20-1.10 (m, 1H, major), 0.95-0,91 (m, 3H, apparent triplet, overlapping doublets for major and minor diastereoisomers).

^{13}C NMR (126 MHz, $CDCl_3$): δ 146.50, 146.36, 128.57 (overlapping major and minor), 127.08, 127.04, 126.44, 126.41, 58.99, 58.96, 56.65, 56.55, 55.53, 55.48, 47.97 (broad peak), 46.11, 46.10, 36.74, 36.70, 30.52, 30.48, 29.80, 14.30, 14.26. *Note. This was isolated as a 1.2:1 mixture of diastereoisomers; the carbon signals typically come in pairs. We were unable to definitively assign major/minor isomers.*

HR-MS (ESI-TOF) *m/z*: $[M+H]^+$ calculated for $C_{16}H_{28}N_3^+$, 262.2278; found mass, 262.2287.

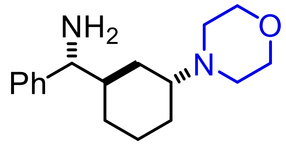

23
29% yield
>20:1 dr

(3-morpholinocyclohexyl)(phenyl)methanamine (23)
This product (29%, 0.058 mmol) was isolated as a mixture of diastereomers with predominantly the shown diastereomer (**>**20:1 selectivity), with > 20:1 selectivity over the 1,3 and 1,5 diamine products.

^{1}H NMR (500 MHz, $CDCl_3$): δ 7.42 – 7.26 (m, 4H), 7.23 (t, *J* = 6.9 Hz, 1H), 3.74 – 3.67 (m, 4H), 2.43 (m, 4H), 2.34 (m, 1H), 2.05 – 1.89 (m, 2H), 1.77 – 1.65 (m, 1H), 1.64 – 1.49 (m, 4H), 1.44 (ddt, *J* = 13.2, 6.6, 3.1 Hz, 2H), 1.38 (dtd, *J* = 12.0, 6.7, 6.2, 3.3 Hz, 2H), 1.08 – 0.96 (m, 1H).

^{13}C NMR (126 MHz, $CDCl_3$): δ 145.98, 128.59, 127.21, 127.18, 67.71, 60.03, 59.20, 50.69, 39.75, 30.97, 29.83, 28.65, 20.78.

HR-MS (ESI-TOF) *m/z*: $[M+H]^+$ calculated for $C_{17}H_{27}N_2O^+$, 275.2118; found mass = 275.2117

E. Control Experiments

a. Reactivity Comparison of *cis* and *trans* olefin isomers

Hydroamination Experiment with (*E*)-**1a**

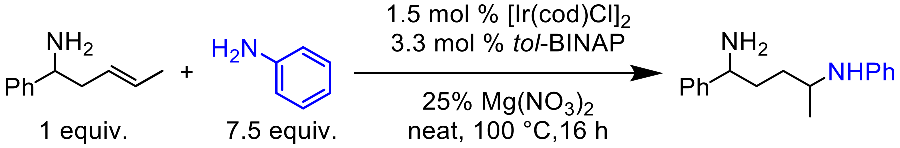

Figure S1. GC spectrum of starting material of (*E*)-**1a** with *E*:*Z* ratio of 6.9:1

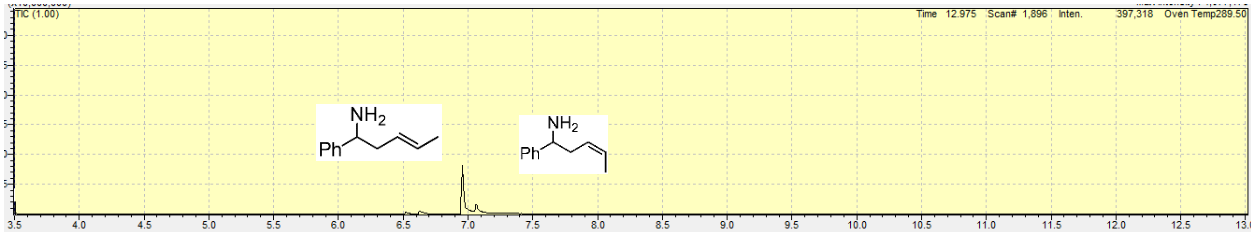

Figure S2. GC spectrum of the reaction mixture after 16 h with remaining (*E*)-**1a** with *E*:*Z* ratio of 3.3:1

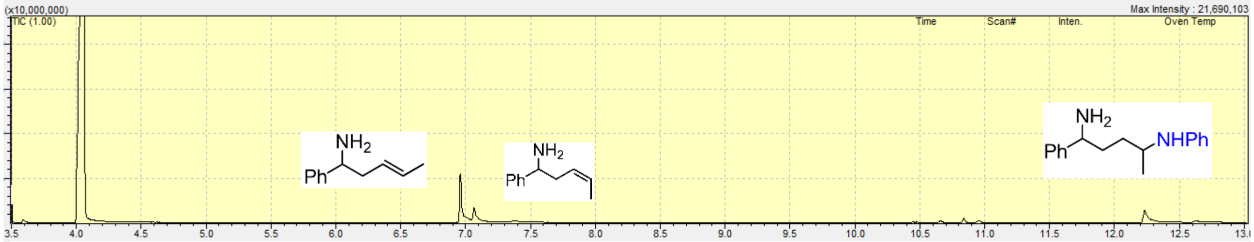

When (*E*)-**1a** is used in the reaction, rather than (*Z*)-**1a**, we see a significant reduction in yield, 35% and 80% yield, respectively. Further, the *E:Z* ratio of **1a** erodes over the course of the reaction, as the intial ratio was 6.9:1 and the final ratio, after 16 hours, is 3.3:1. This suggests that the alkene is isomerizing under our reaction conditions. The d.r. for the reactions with the *cis* and *trans* alkenes are identical, as depicted by the ^{1}H NMR signal corresponding to the CH_3 shown (top spectra, in green, is the *cis* alkene; bottom spectra is the *trans)*.

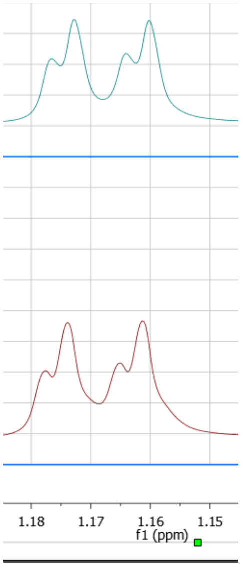

b. Hydroamination Control Experiments with Cyclohexene

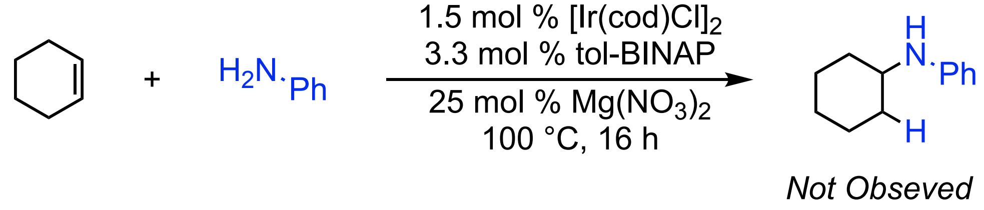

Figure S3. GC trace of hydroamination with cyclohexene and aniline.

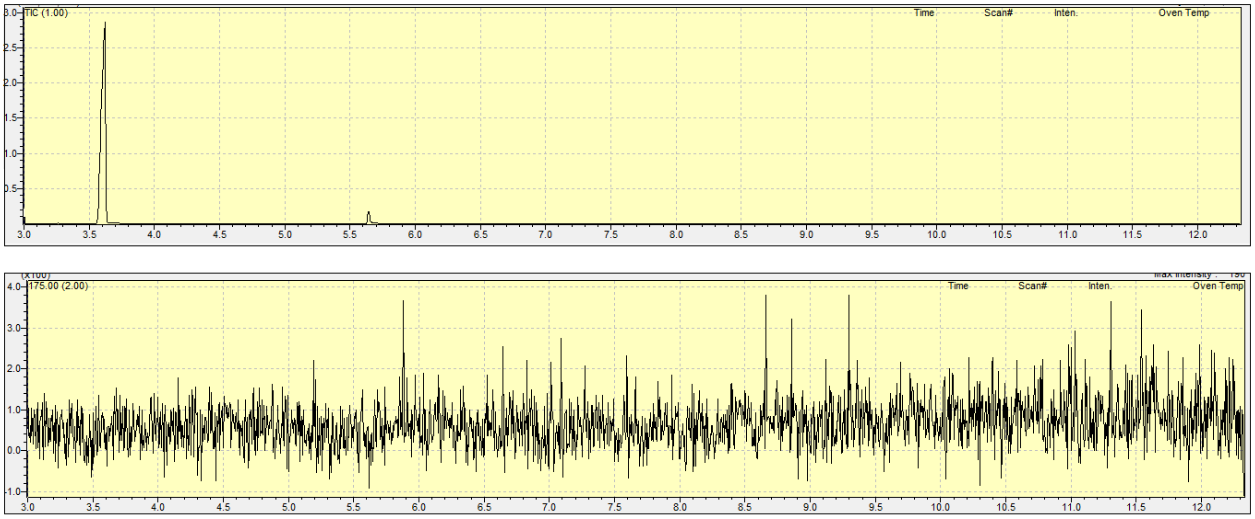

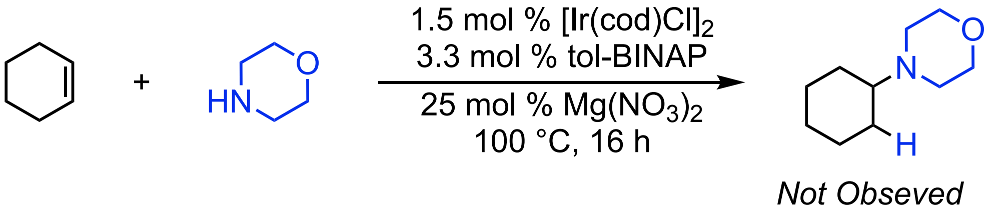

Figure S4. . GC trace of hydroamination with cyclohexene and morpholine.

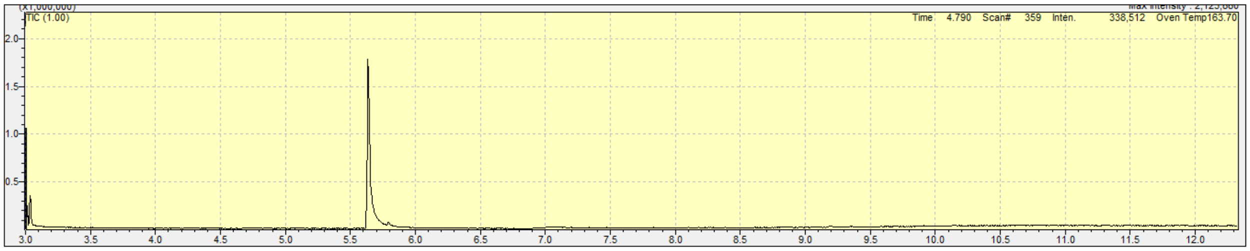

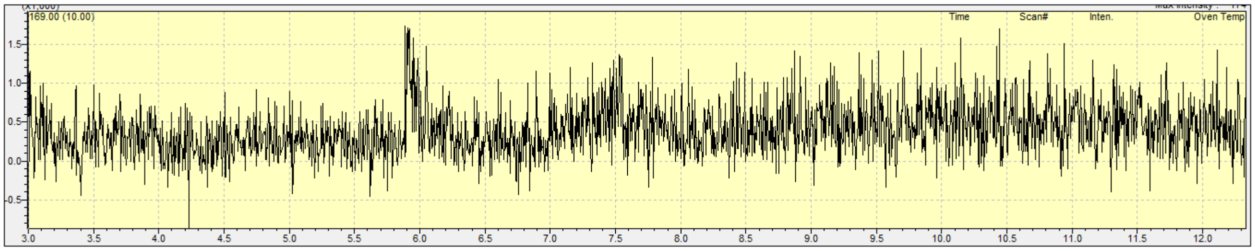

No hydroamination products are observed in the absence of the amine-directing group, suggesting that it is necessary for reactivity.

F. Mechanistic Investigations

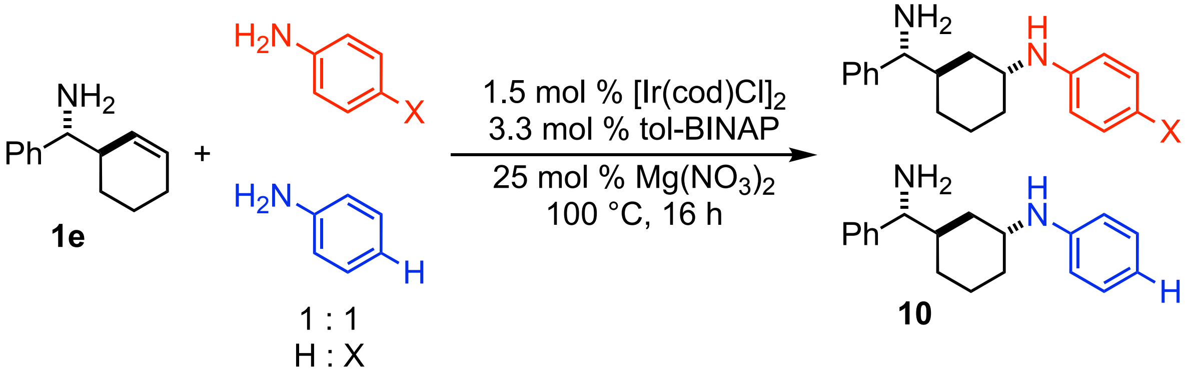

a. Competition Hammett plot with cyclic internal alkene 1e

To a 4 mL vial equipped with stir bar was added $[Ir(cod)Cl]_2$ (2.01 mg, 1.5 mol %), *tol*-BINAP (4.48 mg, 3.3 mol %), $MgNO_3$ (7.4 mg, 0.05 mmol, .25 equiv), **1e** (0.2 mmol, 1 equiv). A mixture of aniline (1.5 mmol, 7.5 equiv) and substituted aniline (1.5 mmol, 7.5 equiv.) was made and heated at 30 °C until the solid was fully dissolved; the aniline mixture was added to the 4 mL vial, sealed with Teflon cap, removed from the nitrogen-filled glove box, and heated to 100 °C for 2 h while stirring. The resulting slurry was then cooled to room temperature, the 4 mL vial was uncapped, 1-methylnaphthalene (5 µL, 35.2 µmol) was added as an internal standard, and *ca* 1 mL each of half-saturated K_2CO_3 (aq) and CH_2Cl_2 was added to the slurry. The 4 mL vial was capped, the biphasic mixture shaken several times. The crude organic layer was analyzed by GC and calibrated *in situ* yields of **X** vs. **H** were determined by comparison to an internal standard.

Table S4. Initial rate constants for the hydroamination of **1e**

Aniline	Products ratio (X/H)	$Log_{10}([X]/[H])$
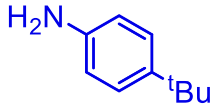 26	1.465	0.630
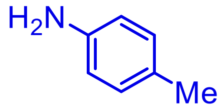 27	1.384	0.618
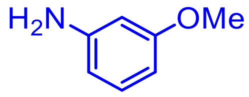 28	0.509	-0.085
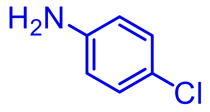 29	0.681	-0.303
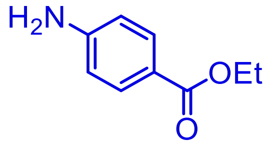 30	0.170	-0.375

^{1}H NMR and HRMS Data for 25-29:

26. ^{1}H NMR (400 MHz, $CDCl_3$) δ 7.22 – 7.13 (m, 7H), 6.61 (d, *J* = 8.2 Hz, 2H), 3.69 (m, 1H), 3.63 (d, *J* = 9.1 Hz, 1H), 2.25 – 2.20 (m, 2H), 2.10-2.00 (m, 1H), 1.83 – 1.67 (m, 2H), 1.52 – 1.33 (m, 4H), 1.27 (s, 9H), 0.97 – 0.77 (m, 1H), 0.77-0.64 (m, 1H).
HR-MS (ESI-TOF) *m/z*: $[M+Na]^+$ calculated for $C_{23}H_{32}N_2Na^+$, 359.2458; found mass, 359.2461

27. ^{1}H NMR (400 MHz, $CDCl_3$) δ 7.39 – 7.22 (m, 5H), 6.95 (d, *J* = 8.2 Hz, 2H), 6.55 (d, *J* = 8.4 Hz, 2H), 3.76 – 3.69 (m, 1H), 3.65 (d, *J* = 8.5 Hz, 1H), 2.21 (s, 3H), 2.19 – 1.98 (m, 2H), 1.84 – 1.71 (m, 1H), 1.50 – 1.35 (m, 3H), 1.34 – 1.20 (m, 2H), 0.92 – 0.78 (m, 1H).
HR-MS (ESI-TOF) *m/z*: $[M+Na]^+$ calculated for $C_{20}H_{26}N_2Na^+$, 317.1988; found mass, 317.1984

28. ^{1}H NMR (500 MHz, $CDCl_3$) δ 7.38 – 7.29 (m, 2H), 7.29 – 7.16 (m, 3H), 7.06 (t, *J* = 8.1 Hz, 1H), 6.24 (d, *J* = 8.1 Hz, 1H), 6.23 (d, *J* = 8.1 Hz, 1H), 6.18 (d, *J* = 2.2 Hz, 1H), 3.77 (s, 3H), 3.76 – 3.71 (m, 1H), 3.66 (d, *J* = 8.1 Hz, 1H), 2.07 (d, *J* = 13.4 Hz, 1H), 1.88 – 1.80 (m, 1H), 1.80 – 1.70 (m, 1H), 1.58 – 1.45 (m, 3H), 1.44 –

1.34 (m, 2H), 0.95 – 0.84 (m, 1H).
HR-MS (ESI-TOF) *m/z*: $[M+Na]^+$ calculated for $C_{20}H_{26}N_2ONa^+$, 333.1937; found mass, 333.1941

29. ^{1}H NMR (400 MHz, $CDCl_3$) δ 7.37 – 7.28 (m, 2H), 7.26 – 7.17 (m, 3H), 7.09 (d, *J* = 8.8 Hz, 2H), 6.54 (d, *J* = 8.9 Hz, 2H), 3.74 – 3.66 (m, 1H), 3.64 (d, J = 8.3 Hz, 1H), 2.67 – 2.17 (bs, 3H), 2.09 (d, *J* = 13.5 Hz, 1H), 2.00 – 1.88 (m, 1H), 1.78 – 1.68 (m, 1H), 1.55 – 1.21 (m, 5H), 0.86 (q, *J* = 10.9 Hz, 1H).
HR-MS (ESI-TOF) *m/z*: $[M+H]^+$ calculated for $C_{19}H_{24}ClN_2^+$, 315.1623; found mass, 315.1617

30. ^{1}H NMR (500 MHz, $CDCl_3$) δ 7.87 (d, *J* = 8.8 Hz, 2H), 7.39 – 7.22 (m, 5H), 6.57 (d, *J* = 8.7 Hz, 2H), 4.32 (q, *J* = 7.1 Hz, 2H), 3.84 (s, 1H), 3.67 (d, *J* = 8.2 Hz, 1H), 2.10 (d, *J* = 12.8 Hz, 1H), 1.90 – 1.68 (m, 6H), 1.62 – 1.48 (m, 3H), 1.47 – 1.39 (m, 1H), 1.37 (t, *J* = 7.1 Hz, 3H), 0.92 (q, *J* = 12.1 Hz, 1H).
HR-MS (ESI-TOF) *m/z*: $[M+H]^+$ calculated for C22H28N2O2$^+$, 353.2224; found mass, 353.2230

Figure S5. Hammett plot for the hydroamination of **1e** with sigma value.

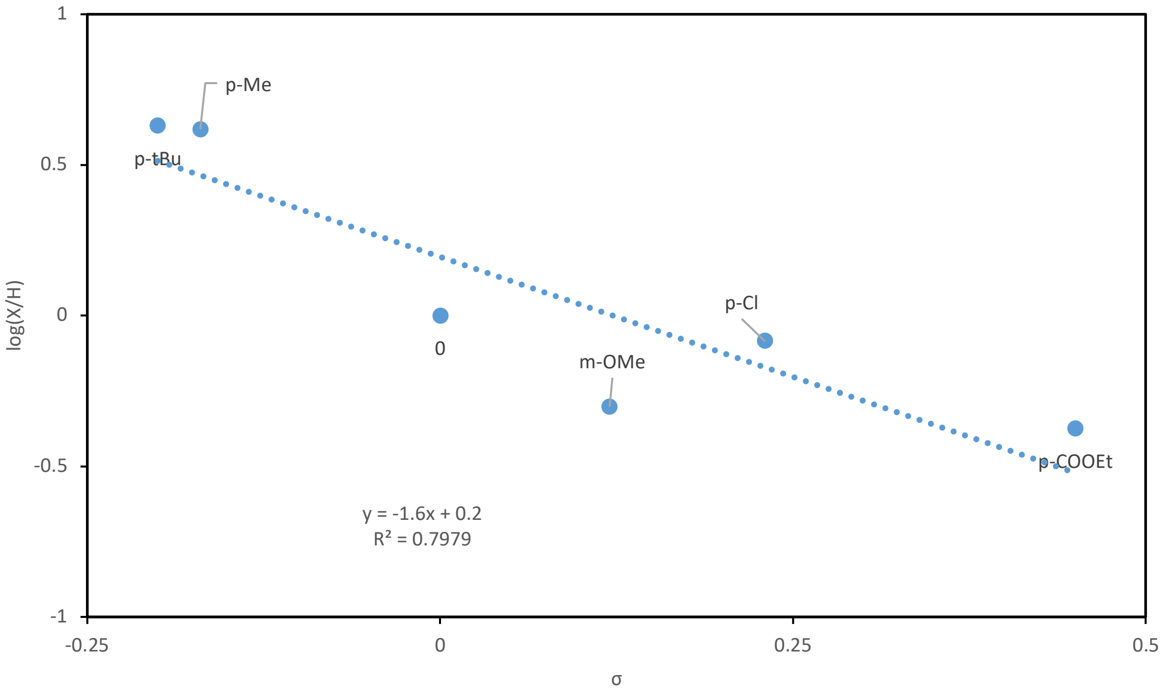

Figure S6. Hammett plot for the hydroamination of **1e** with sigma plus value

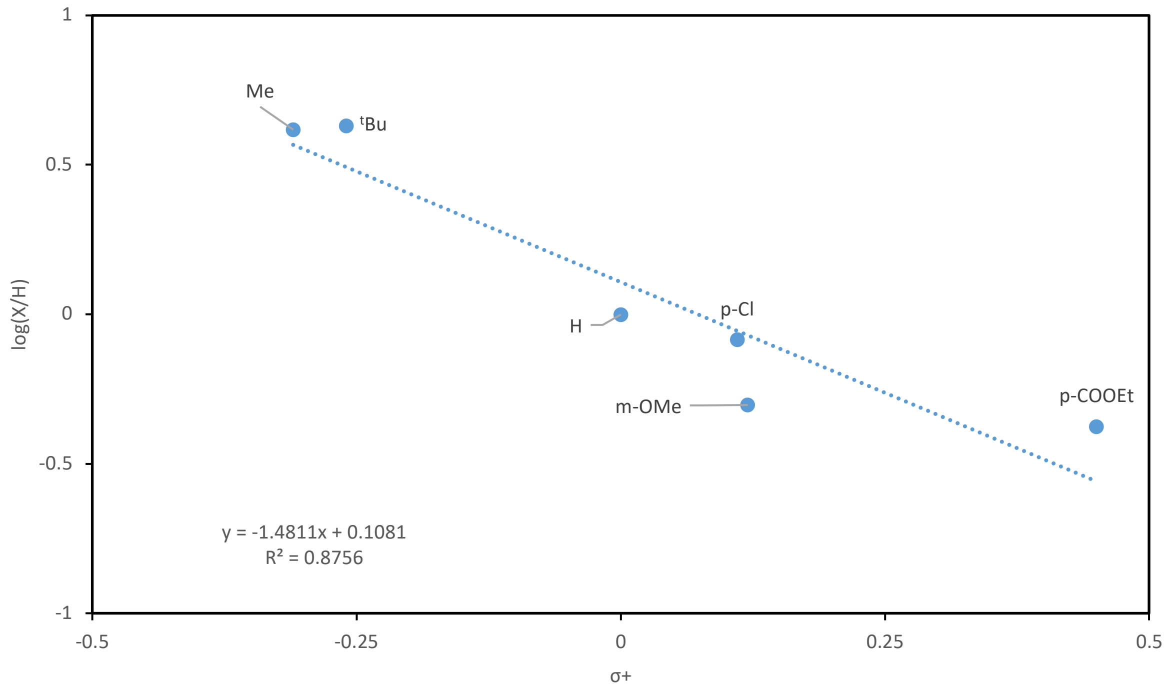

b. Competition Hammett Study with acyclic *cis*-alkene 1a

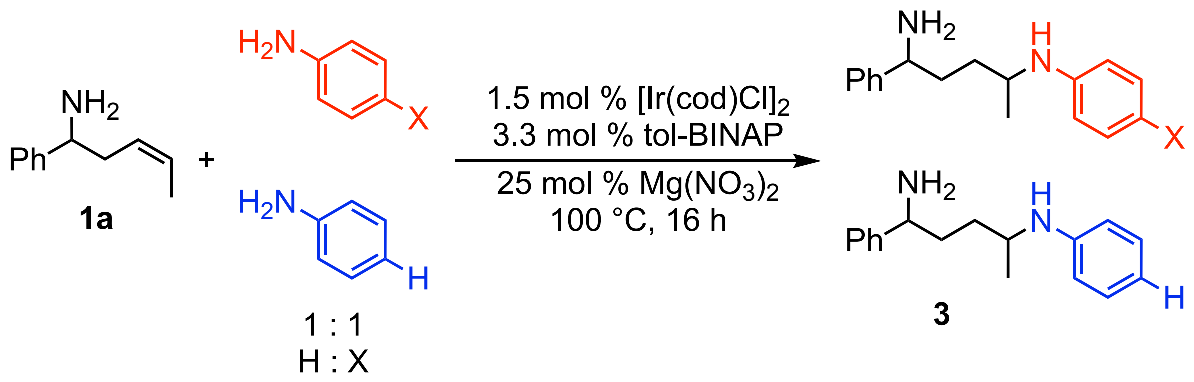

To a 4 mL vial equipped with stir bar was added $[Ir(cod)Cl]_2$ (2.01 mg, 1.5 mol %), *tol*-BINAP (4.48 mg, 3.3 mol %), $MgNO_3$ (7.4 mg, 0.05 mmol, .25 equiv), **1a** (0.2 mmol, 1 equiv). A mixture of aniline (1.5 mmol, 7.5 equiv) and substituted aniline (1.5 mmol, 7.5 equiv.) was made and heated at 30 °C until solid was dissolved and the mixture was homogenous. The anilines mixture was added to the 4 mL vial, the vial was sealed with Teflon cap, removed from nitrogen filled glove box, and heated to 100 °C for 2 h while stirring. The resulting slurry was then cooled to room temperature, the 4 mL vial was uncapped, 1-methylnaphthalene (5 µL, 35.2 µmol) was added as an internal standard, and *ca* 1 mL each of half-saturated K_2CO_3 (aq) and CH_2Cl_2 was added to the slurry. The 4 mL vial was capped, the biphasic mixture shaken several times. The crude organic layer was analyzed by GC and calibrated *in situ* yields of **X** vs. **H** were determined by comparison to an internal standard.

Figure S7. Hammett plot for the hydroamination of **1a** with sigma value.

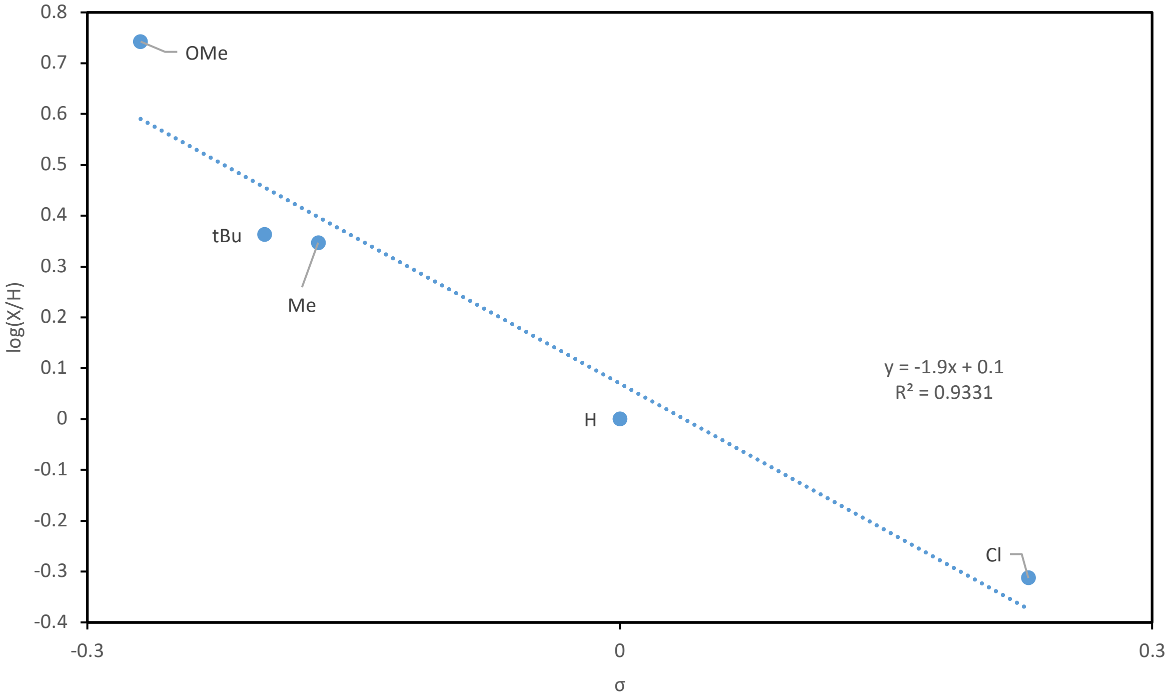

Figure S8. Hammett plot for the hydroamination of **1a** with sigma plus value

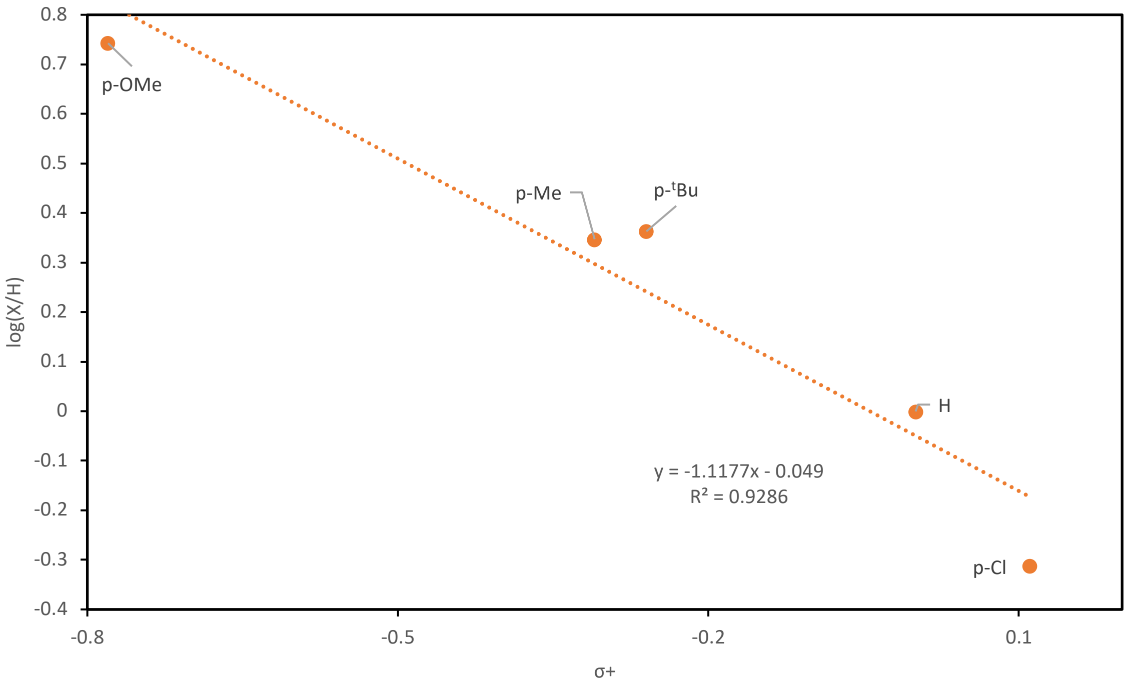

Table S5. Initial rate constants for the hydroamination of **1a**

Aniline	Products ratio (X/H)				$Log_{10}([X]/[H])$
	Run 1	Run 2	Run 3	Average	
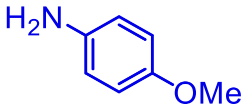 6	7.278	4.658	4.651	6±1	0.7426
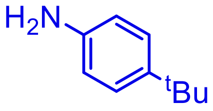 31	2.663	1.976	2.284	2.3±0.3	0.3632
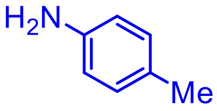 32	2.575	2.291	1.807	2.2±0.4	0.3472
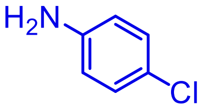 33	0.484	0.490	0.488	0.487±0.002	-0.3123

^{1}H NMR Data for 30-32:

31. ^{1}H NMR (500 MHz, $CDCl_3$) δ 7.36 – 7.21 (m, 7H), 7.16 (d, *J* = 8.6 Hz, 2H), 6.48 (d, *J* = 8.4 Hz, 2H), 3.88 (t, *J* = 6.9 Hz, 1H), 3.47 – 3.37 (m, 1H), 2.13 (s, 3H), 1.90 – 1.71 (m, 2H), 1.60 (dddd, *J* = 13.0, 11.1, 6.3, 4.9 Hz, 1H (0.65H, major)), 1.51 (ddt, *J* = 13.1, 8.9, 6.4 Hz, 1H (0.35H, minot)), 1.47 – 1.38 (m, 1H (0.35H, minor)), 1.38 – 1.29 (m, 1H (0.65H, major)), 1.27 (s, 9H), 1.13 (d, *J* = 6.3 Hz, 3H (minor)), 1.13 (d, *J* = 6.3 Hz, 3H (major)).
HR-MS (ESI-TOF) *m/z*: $[M+H]^+$ calculated for $C_{21}H_{30}N_2^+$, 311.2475; found mass, 311.2482

32. ^{1}H NMR (500 MHz, $CDCl_3$) δ 7.38 – 7.21 (m, 5H), 6.95 (d, *J* = 7.9 Hz, 2H), 6.46 (d, *J* = 7.9 Hz, 2H), 3.88 (t, *J* = 6.9 Hz, 1H), 3.40 (d, *J* = 6.3 Hz, 1H), 2.22 (s, 3H), 2.09 (s, 3H), 1.89 – 1.70 (m, 2H), 1.60 (tt, *J* = 11.3, 5.5 Hz, 1H (0.7H, major)), 1.51 (dq, *J* = 14.9, 7.0 Hz, 1H (0.3H minor)), 1.47 – 1.37 (m, 1H (0.3H, minor)), 1.32 (tt, *J* = 12.0, 5.8 Hz, 1H (0.7H, major)), 1.12 (d, *J* = 6.4 Hz, 3H).
HR-MS (ESI-TOF) *m/z*: $[M+H]^+$ calculated for $C_{21}H_{31}N_2^+$, 311.2482; found mass, 311.2475

33. ^{1}H NMR (500 MHz, $CDCl_3$) δ 7.32 – 7.23 (m, 5H), 7.05 (d, *J* = 8.8 Hz, 2H), 6.40 (d, *J* = 8.8 Hz, 2H), 3.90 (t, *J* = 7.0 Hz, 1H), 3.35 (q, *J* = 6.4 Hz, 1H), 2.43 (s, 3H), 1.94 – 1.72 (m, 2H), 1.57 (tt, *J* = 11.5, 5.8 Hz, 1H (0.7H major)f), 1.52 – 1.37 (m, 2H (0.6H, minor)), 1.32 (td, *J* = 12.1, 5.7 Hz, 1H (0.7H, major)), 1.10 (d, *J* = 6.3 Hz, 3H (minor)), 1.09 (d, *J* = 6.3 Hz, 3H (major)).
HR-MS (ESI-TOF) *m/z*: $[M+H]^+$ calculated for $C_{17}H_{22}ClN_2^+$, 289.1466; found mass, 289.1462

Table S6. Chloride Loading Screen

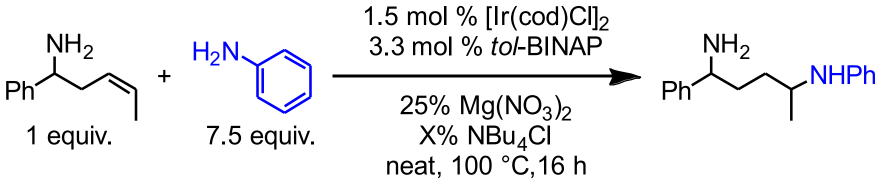

NBu_4Cl loading	GC yield (%)
none	80
10 mol%	10

G. X-Ray Crystal Structure Data for 10 and 22

Preparation of 10•napthalene-1,5-disulfonic acid

X-ray quality crystals were obtained for **10**•naphthalene-1,5-disulfonic acid by mixing 0.1 mmol **10** and 0.1 mmol Armstrong's Acid (naphthalene-1,5-disulfonic acid)in 1 mL ethanol for 30 minutes at room temperature, then taking a small portion of this and layering it with fresh ethanol and EtOAc as an antisolvent, and waiting for 2 weeks for suitable crystals to form.

X-ray crystallography parameters and select bond lengths and angles for 10•Armstrong's Acid:

X-Ray Diffraction Techniques: The structure was collected on a Bruker three-circle platform goniometer equipped with an Apex II CCD and an Oxford cyrostream cooling device. Radiation was from a graphite fine focus sealed tube Mo Kα (0.71073 Å) source. A suitable crystal was mounted on a cryoloop using paratone N oil. The structure was collected at 100 K. Data was collected as a series of ϕ and/ or ω scans. Data was integrated using SAINT[1] and scaled with either numerical or multi-scan absorption correcting using SADABS.[1] Using Olex2,[2] the structure was solved with the XS[3] structure solution program using the Patterson method and refined with the XL[4] refinement package using Least Squares minimization.

Table S7. X-ray diffraction experimental details for **10•** napthalene-1,5-disulfonic acid

Formula	$C_{160}H_{128}N_8O_{32}S_8$
W	2931.26
Crystal system	Monoclinic
Space group (Z)	$P2_1/C$
a (Å)	15.6273(6)
b (Å)	11.1454(4)
c (Å)	21.3411(8)
α (°)	90°
β (°)	104.896(2)°
γ (°)	90°
Volume (Å^3)	3592.1(2)
Calc. ρ (g/cm^3)	1.355
μ (mm^{-1})	0.21
Crystal Size (mm)	0.104x0.119x0.322
Reflections	74455
Completeness (to 2θ)	1.0 (50.79)
GOF on F^2	1.122
R1, wR2[c] [I>2σ(I)]	0.0529, 0.1797

[a] λ = 0.71073 Å; [b] T=100 K; [c] $R1 = \Sigma||F_o|-|F_c||/\Sigma|F_o|$, $wR2 = \{\Sigma[w(F_o^2-F_c^2)^2]/\Sigma[w(F_o^2)^2]\}^{1/2}$

Figure S9. Crystal structure of **10**•napthalene-1,5-disulfonic acid with select atoms labeled. Hydrogen atoms have been omitted, one thing to note is that both amines are protonated. Thermal ellipsoids are drawn at 50% probability.

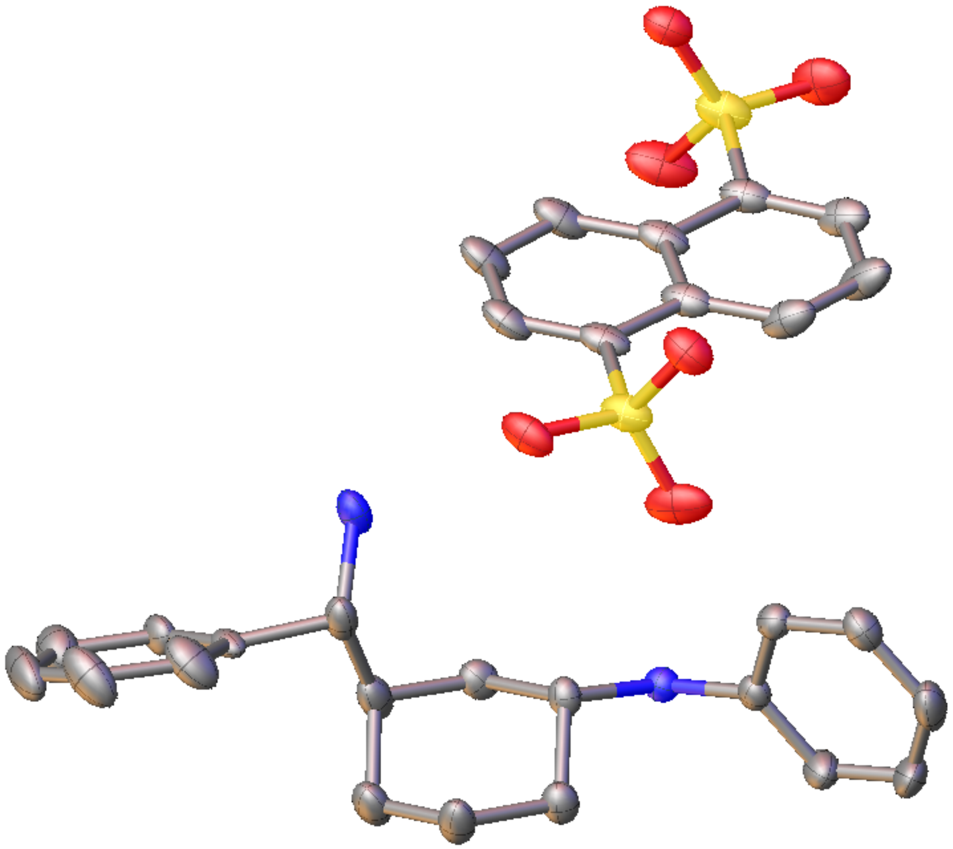

X-ray quality crystals were obtained for **22**•2TsOH through mixing 0.2 mmol **22** and 0.42 mmol *p*-toluene sulfonic acid monohydrate (TsOH•H_2O) in 1 mL ethanol for 30 minutes at room temperature, then taking a small portion of this and layering it with fresh ethanol and diethyl ether as an antisolvent, and waiting for 2 weeks for suitable crystals to form.

X-ray crystallography parameters and select bond lengths and angles for 22•2TsOH:

X-Ray Diffraction Techniques: The structure was collected on a Bruker three-circle platform goniometer equipped with an Apex II CCD and an Oxford cyrostream cooling device. Radiation was from a graphite fine focus sealed tube Mo Kα (0.71073 Å) source. A suitable crystal was mounted on a cryoloop using paratone N oil. The structure was collected at 100 K. Data was collected as a series of ϕ and/ or ω scans. Data was integrated using SAINT and scaled with either numerical or multi-scan absorption correcting using SADABS. Using Olex2, the structure was solved with the XS structure solution program using the Patterson method and refined with the XL refinement package using Least Squares minimization.

Table S8. X-ray diffraction experimental details for **22**•2TsOH

Formula	$C_{31}H_{42}N_2O_7S_2$
W	2475.13
Crystal system	Cubic
Space group (Z)	$P2_12_12_1$
a (Å)	11.1123(4)
b (Å)	11.2096(4)
c (Å)	24.9678(9)
α (°)	90°
β (°)	90°
γ (°)	90°
Volume (Å^3)	3110.1(0)
Calc. ρ (g/cm^3)	1.322
μ (mm^{-1})	0.22
Crystal Size (mm)	0.15x0.475x0.529
Reflections	77911
Completeness (to 2θ)	1.0 (56.61)
GOF on F^2	1.035
R1, wR2^c [I>2σ(I)]	0.0275, 0.0737

^{a} λ = 0.71073 Å; ^{b} T=100 K; ^{c} $R1 = \Sigma||F_o|-|F_c||/\Sigma|F_o|$, $wR2 = \{\Sigma[w(F_o^2-F_c^2)^2]/\Sigma[w(F_o^2)^2]\}^{1/2}$

Figure S10. Crystal structure of **22**•2TsOH with select atoms labeled. Hydrogen atoms have been omitted, one thing to note is that both amines are protonated. Thermal ellipsoids are drawn at 50% probability.

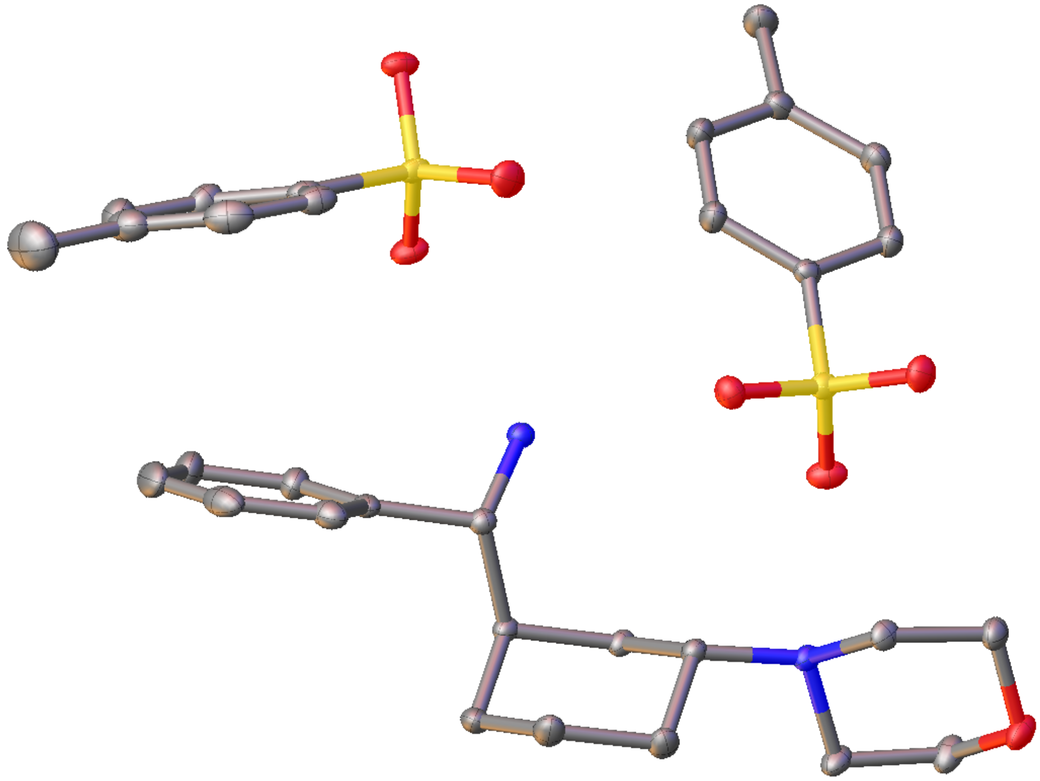

H. References

(1) Bruker AXS. **2009**, Apexx II. Bruker AXS, Madison, Wisconsin.

(2) O. V. Dolomanov, L. J. Bourhis, R. J. Gildea,J. A. K. Howard, H. Puschmann, *Journal of Applied Crystallography* 2009, **42**, 339-341.

(3) L. J. Bourhis, O. V. Dolomanov, R. J. Gildea, J. A. K. Howard, H Puschmann, *Acta Crystallographica Section A Foundations and Advances* 2015, **71**, 59-75.

(4) G. M. Sheldrick, *Acta Crystallographica Section A Foundations of Crystallography* 2008, **64**, 112-122.

I. HRMS Spectra

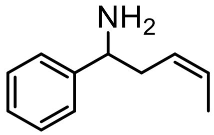

1a

HR-MS (ESI-TOF) *m/z*: [M+H]$^+$ calculated for $C_{11}H_{16}N^+$, 162.1283; found, 162.1285.

Simulated

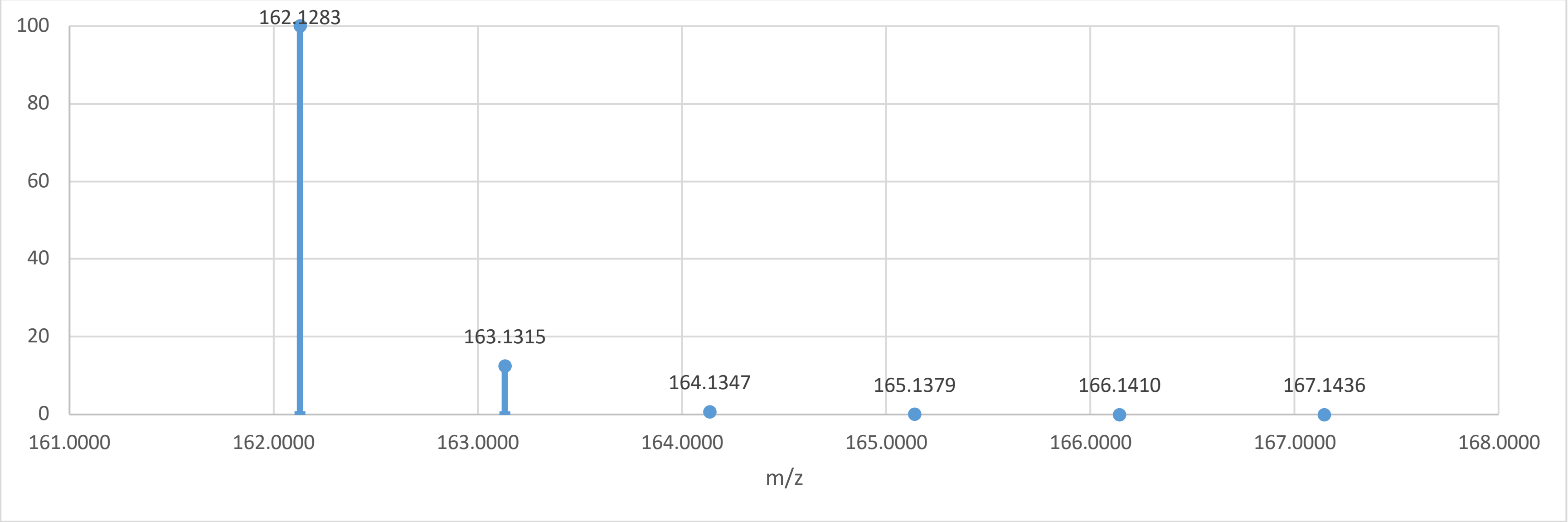

Measured

Monoisotopic Mass, Even Electron Ions
51 formula(e) evaluated with 1 results within limits (up to 50 closest results for each mass)
Elements Used:
C: 0-200 H: 0-200 N: 0-6 Na: 0-1 Br: 0-1

ev-e-z-me
Qtof_69271 57 (2.183) AM (Cen,5, 80.00, Ar,14000.0,558.36,0.70,LS 5); Sm (SG, 2x5.00); Cm (57:61) 1: TOF MS ES+
1.57e+003

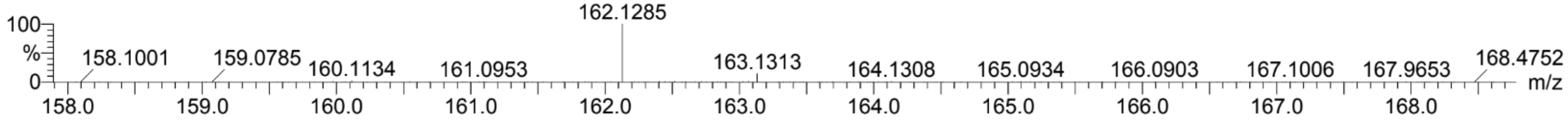

Minimum:				-1.5		
Maximum:		5.0	5.0	100.0		
Mass	Calc. Mass	mDa	PPM	DBE	i-FIT	Formula
162.1285	162.1283	0.2	1.2	4.5	0.9	C11 H16 N

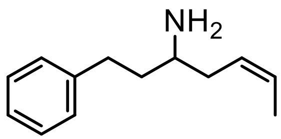

1b

HR-MS (ESI-TOF) *m/z*: $[M+H]^+$ jcalculated for $C_{13}H_{20}N^+$, 190.1596; found, 190.1597.

Simulated

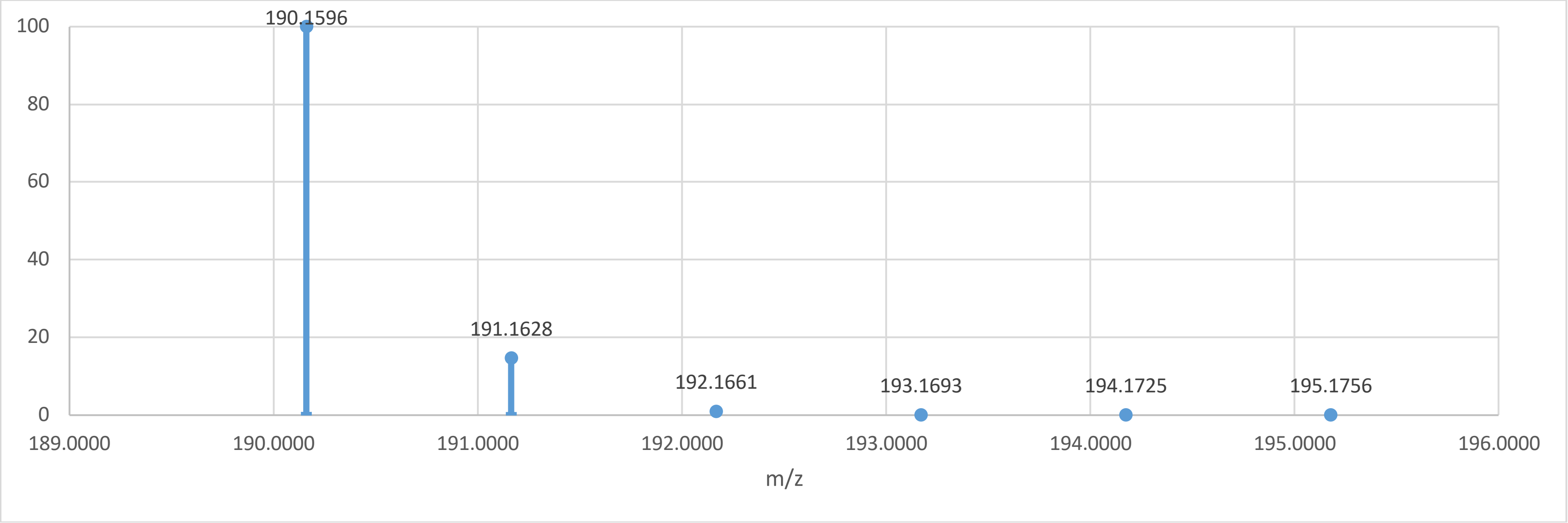

Measured

Monoisotopic Mass, Even Electron Ions
40 formula(e) evaluated with 1 results within limits (up to 50 closest results for each mass)
Elements Used:
C: 0-200 H: 0-200 N: 0-6 Na: 0-1

ev-e-hcinn
Qtof_69269 68 (2.591) AM (Cen,5, 80.00, Ar,14000.0,558.36,0.70,LS 5); Sm (SG, 2x5.00); Cm (68:72)

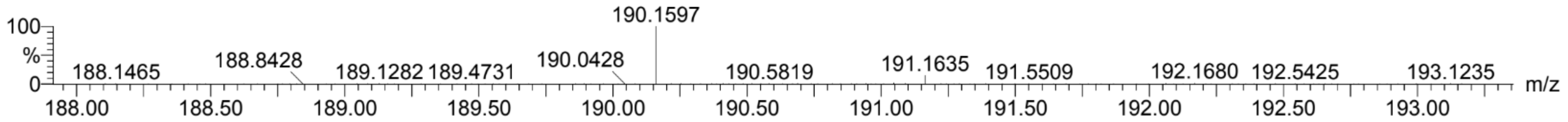

Minimum:				-1.5		
Maximum:		5.0	5.0	100.0		
Mass	Calc. Mass	mDa	PPM	DBE	i-FIT	Formula
190.1597	190.1596	0.1	0.5	4.5	0.4	C13 H20 N

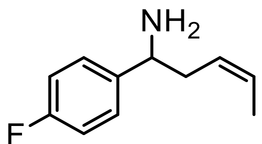

1c

HR-MS (ESI-TOF) *m/z*: $[M+H]^+$ calculated for $C_{11}H_{15}FN^+$, 180.1189; found, 180.1190.

Simulated

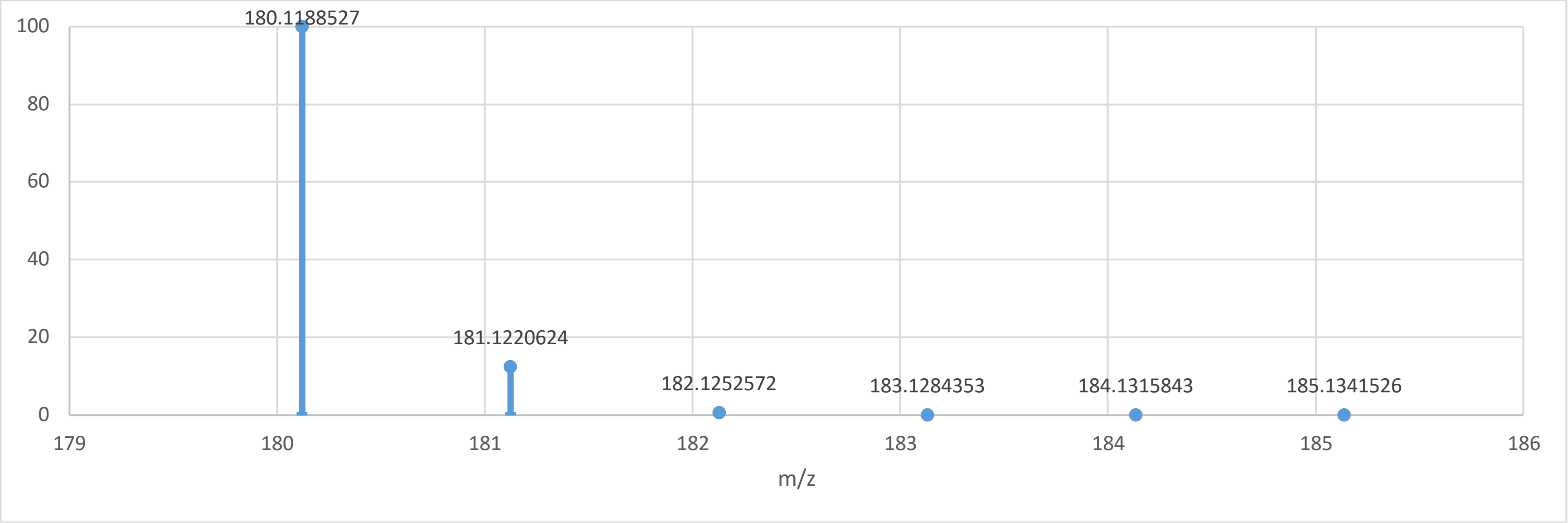

Measured

MS Zoomed Spectrum

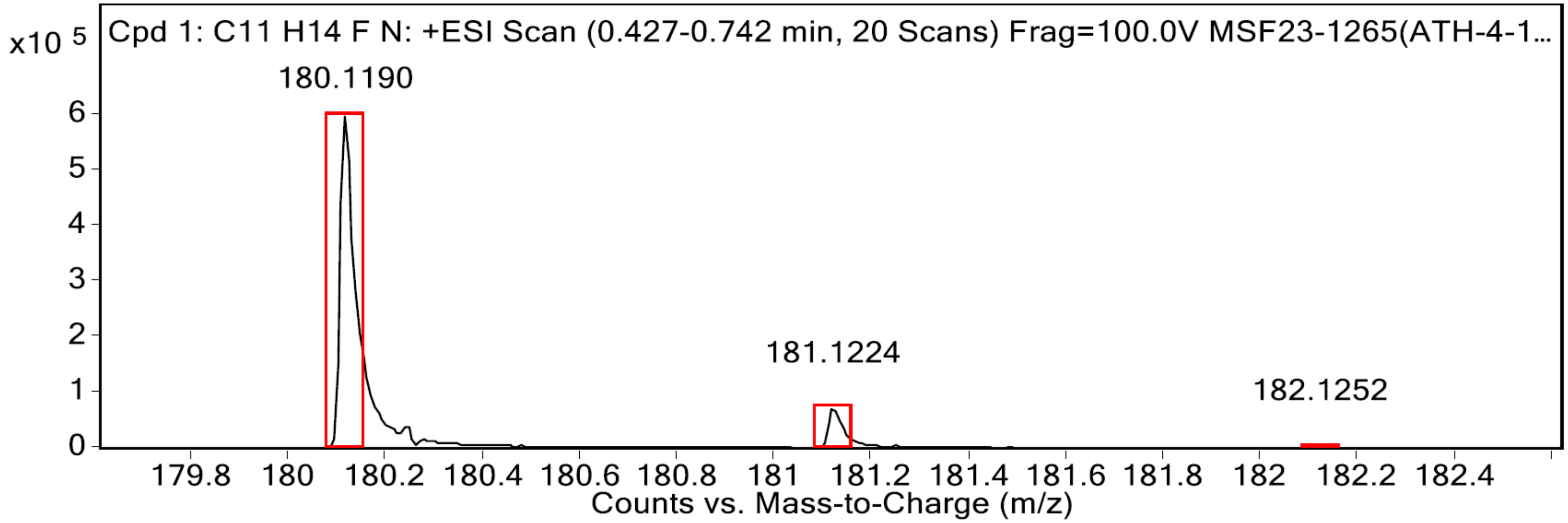

MS Spectrum Peak List

Obs. m/z	Calc. m/z	Charge	Abundance	Formula	Ion Species	Tgt Mass Error (ppm)
163.0927			11743325			
180.1190	180.1183	1	601519	C11H14FN	(M+H)+	-3.84
181.1224	181.1215	1	73559	C11H14FN	(M+H)+	-5.07
182.1252	182.1247	1	5551	C11H14FN	(M+H)+	-2.85

--- End Of Report ---

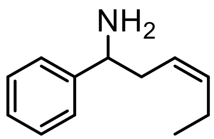

1d

HR-MS (ESI-TOF) *m/z*: $[M+H]^+$ calculated for $C_{12}H_{18}N+$, 176.1439; found,z 176.1440.

Simulated

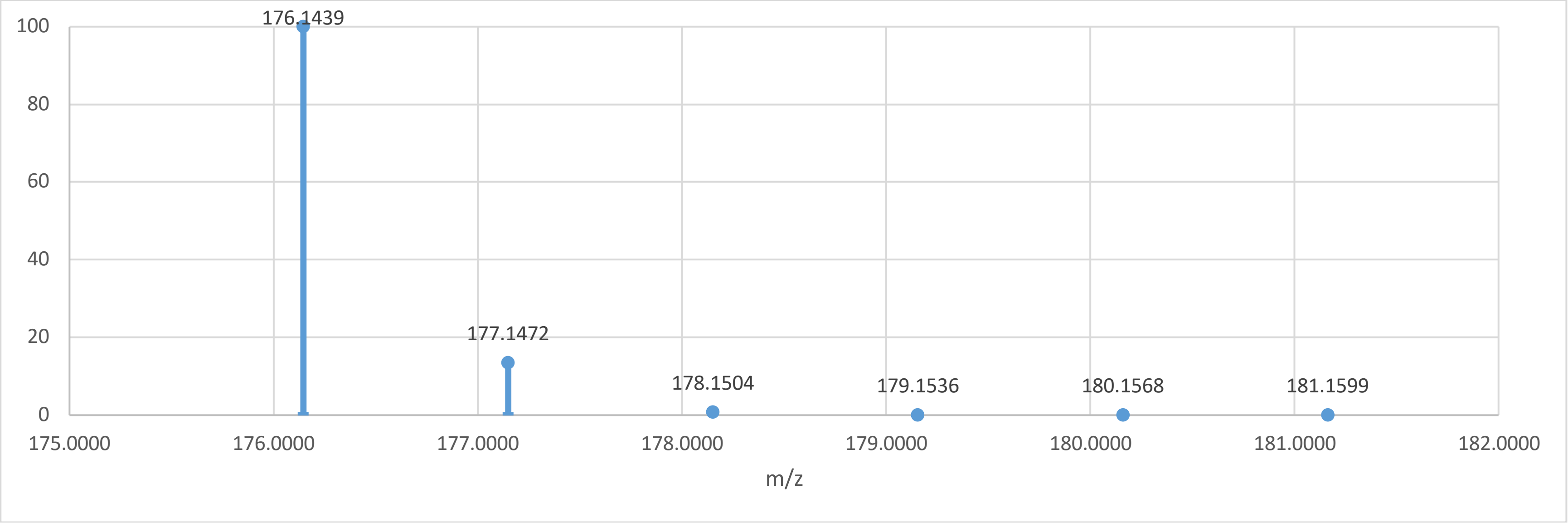

Measured

Monoisotopic Mass, Even Electron Ions
58 formula(e) evaluated with 1 results within limits (up to 50 closest results for each mass)
Elements Used:
C: 0-200 H: 0-200 N: 0-6 Na: 0-1 Br: 0-1

c12h17n
Qtof_69275 59 (2.235) AM (Cen,5, 80.00, Ar,14000.0,558.36,0.70,LS 5); Sm (SG, 2x5.00); Cm (56:60)

1: TOF MS ES+
3.41e+003

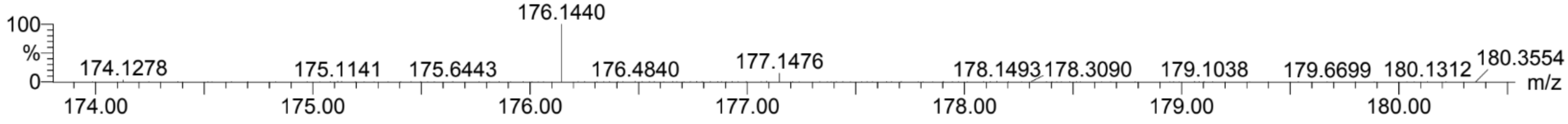

Minimum:				-1.5		
Maximum:		5.0	5.0	100.0		
Mass	Calc. Mass	mDa	PPM	DBE	i-FIT	Formula
176.1440	176.1439	0.1	0.6	4.5	1.8	C12 H18 N

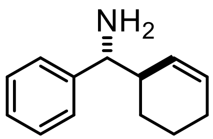

1e

HR-MS (ESI-TOF) *m/z*: $[M+H]^+$ calculated for $C_{13}H_{18}N^+$, 188.1439; found, 188.1445.

Simulated

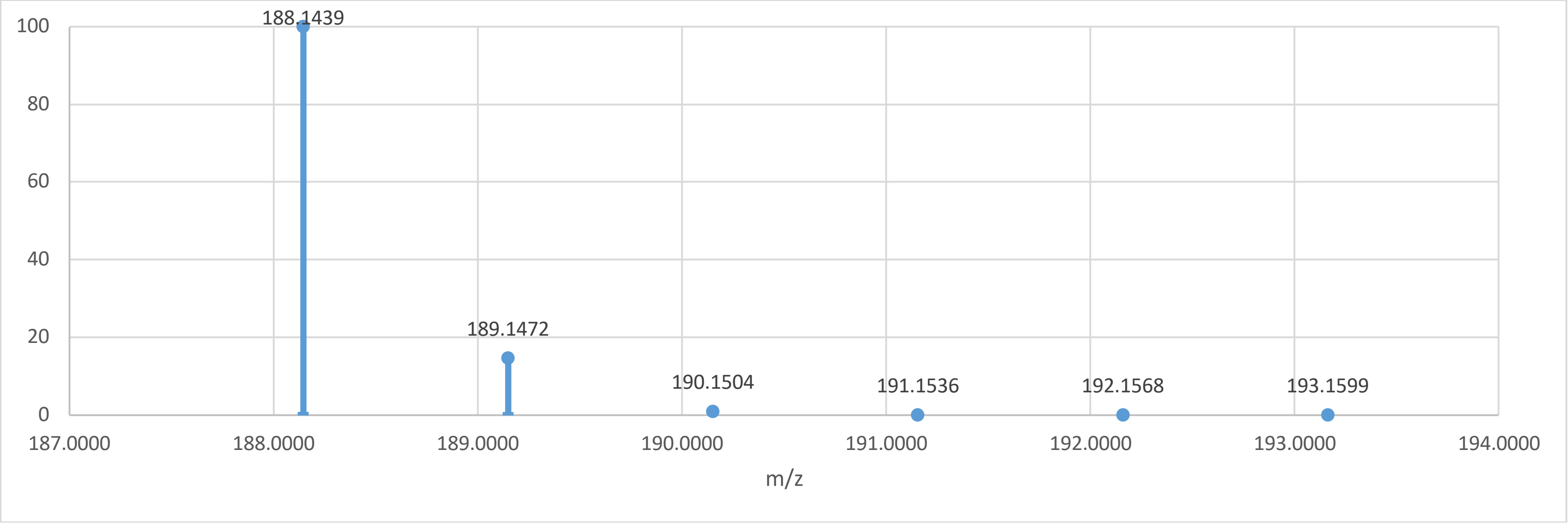

Measured

Monoisotopic Mass, Even Electron Ions
39 formula(e) evaluated with 1 results within limits (up to 50 closest results for each mass)
Elements Used:
C: 0-200   H: 0-200   N: 0-6   Na: 0-1

ev-e-cyen
Qtof_69267 73 (2.796) AM (Cen,5, 80.00, Ar,14000.0,558.36,0.70,LS 5); Sm (SG, 2x5.00); Cm (73:78)   1: TOF MS ES+ 3.50e+003

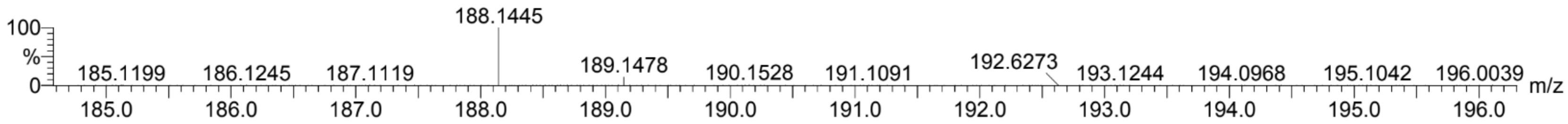

Mass	Calc. Mass	mDa	PPM	DBE	i-FIT	Formula
Minimum:				-1.5		
Maximum:		5.0	5.0	100.0		
188.1445	188.1439	0.6	3.2	5.5	1.3	C13 H18 N

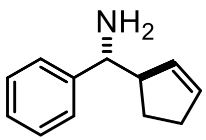

1f

HR-MS (ESI-TOF) *m/z*: $[M+H]^+$ calculated for $C_{12}H_{16}N+$, 174.1277; found, 174.1277

Simulated

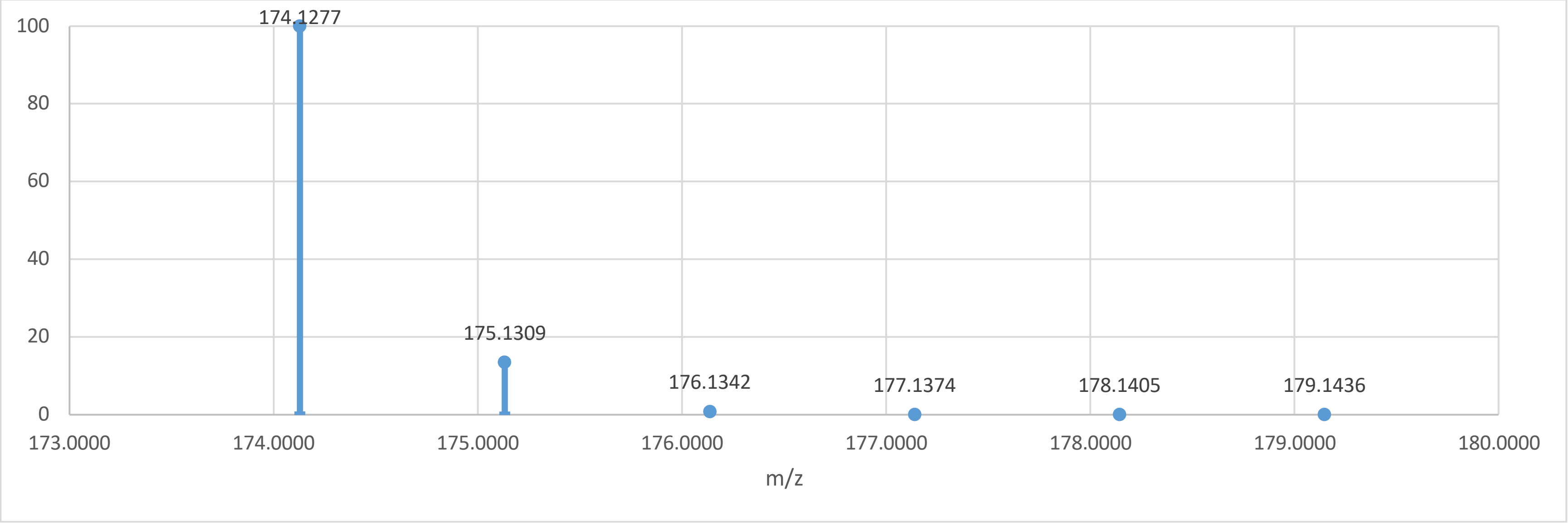

Measured

Monoisotopic Mass, Even Electron Ions
37 formula(e) evaluated with 1 results within limits (up to 50 closest results for each mass)
Elements Used:
C: 0-200 H: 0-200 N: 0-6 Na: 0-1

ev-e-cyp
Qtof_69268 58 (2.217) AM (Cen,5, 80.00, Ar,14000.0,558.36,0.70,LS 5); Sm (SG, 2x5.00); Cm (55:58)

1: TOF MS ES+
6.02e+002

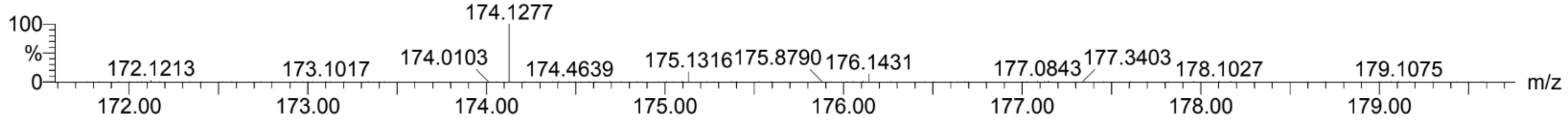

Mass	Calc. Mass	mDa	PPM	DBE	i-FIT	Formula
Minimum:				-1.5		
Maximum:		5.0	5.0	100.0		
174.1277	174.1283	-0.6	-3.4	5.5	36.6	C12 H16 N

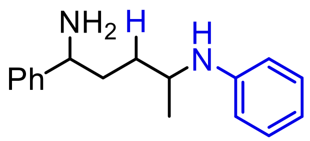

3

HR-MS (ESI-TOF) *m/z*: $[M+H]^+$ calculated for $C_{17}H_{23}N_2$+, 255.1856; found, 255.1864.

Simulated

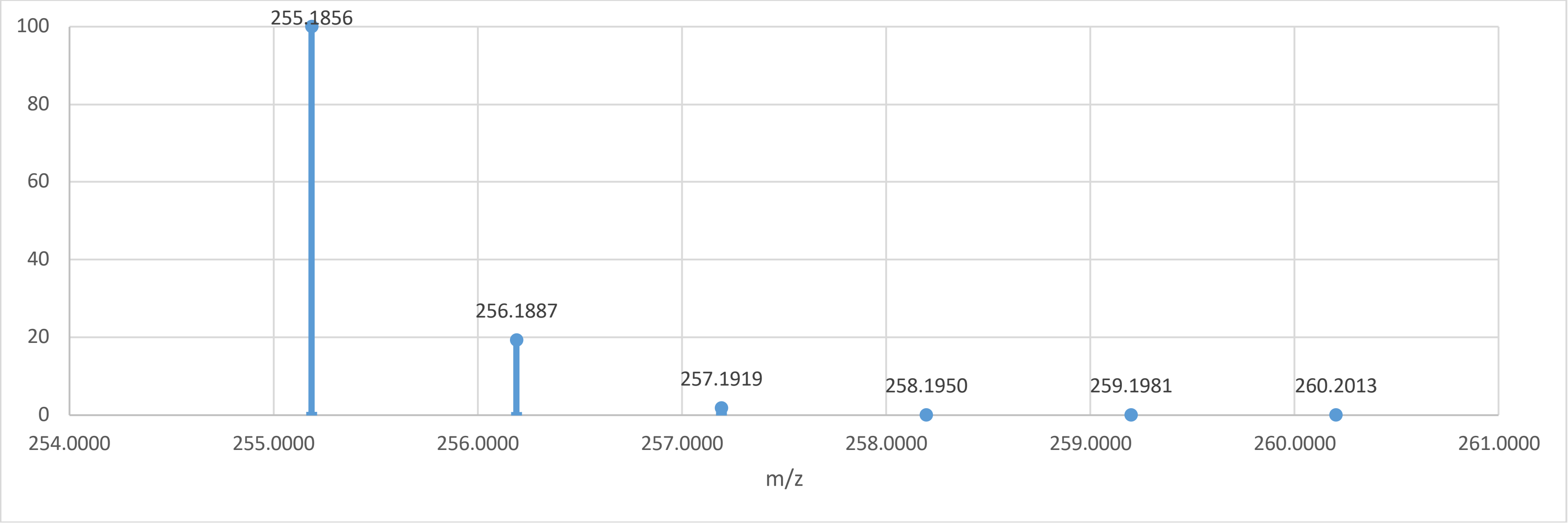

Measured

Monoisotopic Mass, Even Electron Ions
225 formula(e) evaluated with 1 results within limits (up to 50 closest results for each mass)
Elements Used:
C: 0-200 H: 0-200 N: 0-5 O: 0-5 Na: 0-1

1370-6-z-me-an
Qtof_68319 74 (2.830) AM (Cen,5, 80.00, Ar,14000.0,558.36,0.70,LS 5); Sm (SG, 2x5.00); Cm (74:79)

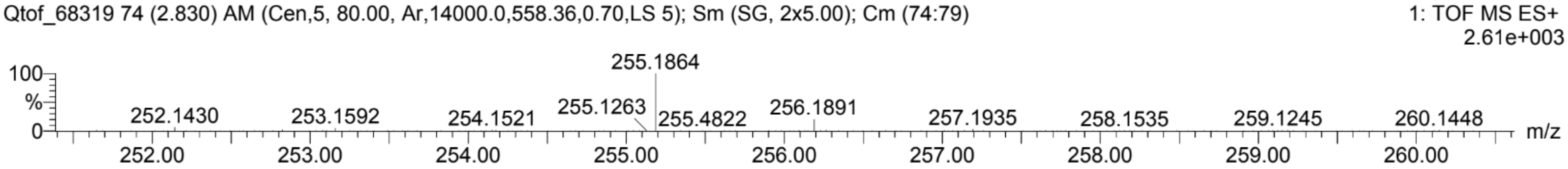

	Mass	Calc. Mass	mDa	PPM	DBE	i-FIT	Formula
Minimum:					-1.5		
Maximum:			5.0	5.0	100.0		
	255.1864	255.1861	0.3	1.2	7.5	1.7	C17 H23 N2

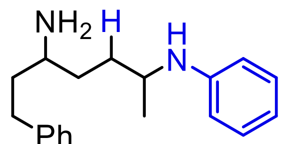

4

HR-MS (ESI-TOF) *m/z*: $[M+H]^+$ calculated for $C_{19}H_{27}N_2$+, 283.2169; found mass, 283.2163.

Simulated

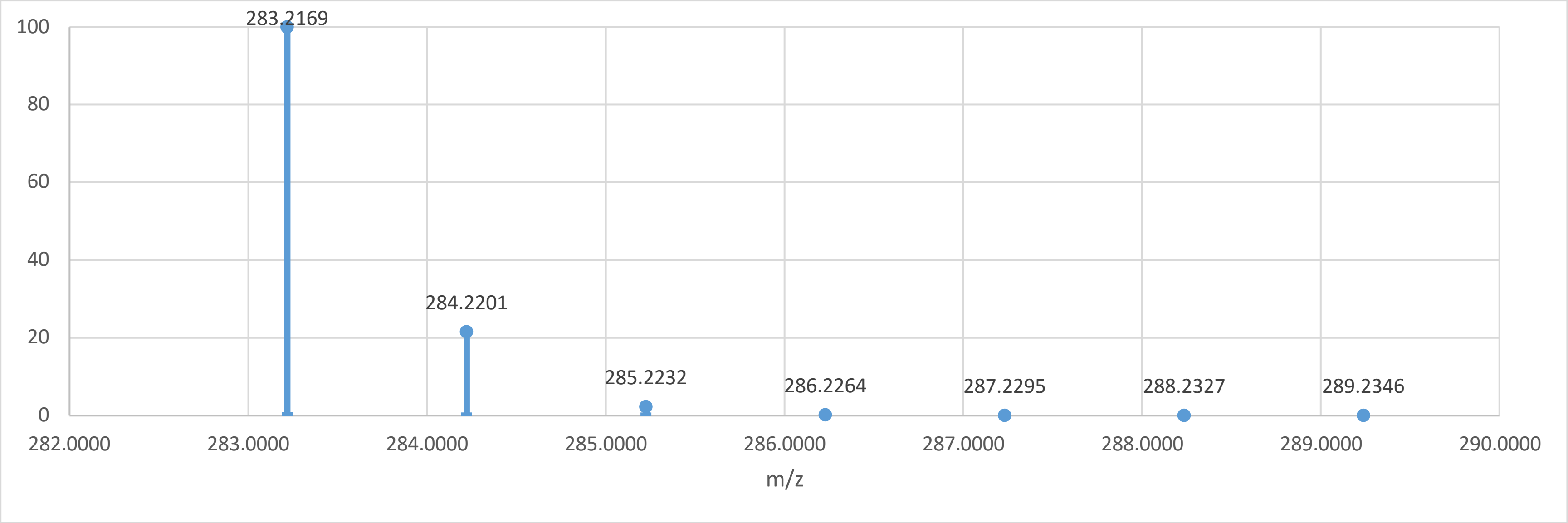

Measured

Monoisotopic Mass, Even Electron Ions
249 formula(e) evaluated with 2 results within limits (up to 50 closest results for each mass)
Elements Used:
C: 0-200 H: 0-200 N: 0-5 O: 0-5 Na: 0-1
1392-an-hcinn
Qtof_68321 46 (1.775) AM (Cen,5, 80.00, Ar,14000.0,558.36,0.70,LS 5); Sm (SG, 2x5.00); Cm (45:46) 1: TOF MS ES+ 4.05e+003

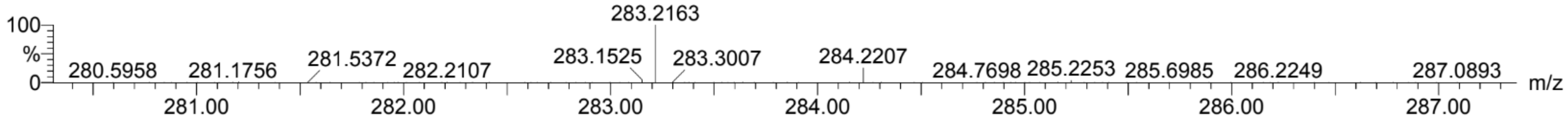

Minimum:				-1.5		
Maximum:		5.0	5.0	100.0		
Mass	Calc. Mass	mDa	PPM	DBE	i-FIT	Formula
283.2163	283.2174	-1.1	-3.9	7.5	10.1	C19 H27 N2
	283.2150	1.3	4.6	4.5	28.5	C17 H28 N2 Na

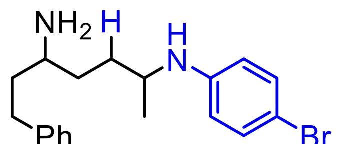

5

HR-MS (ESI-TOF) *m/z*: [M+H]$^+$ calculated for $C_{19}H_{26}N_2Br+$, 361.1279; found, 361.1274.

Simulated

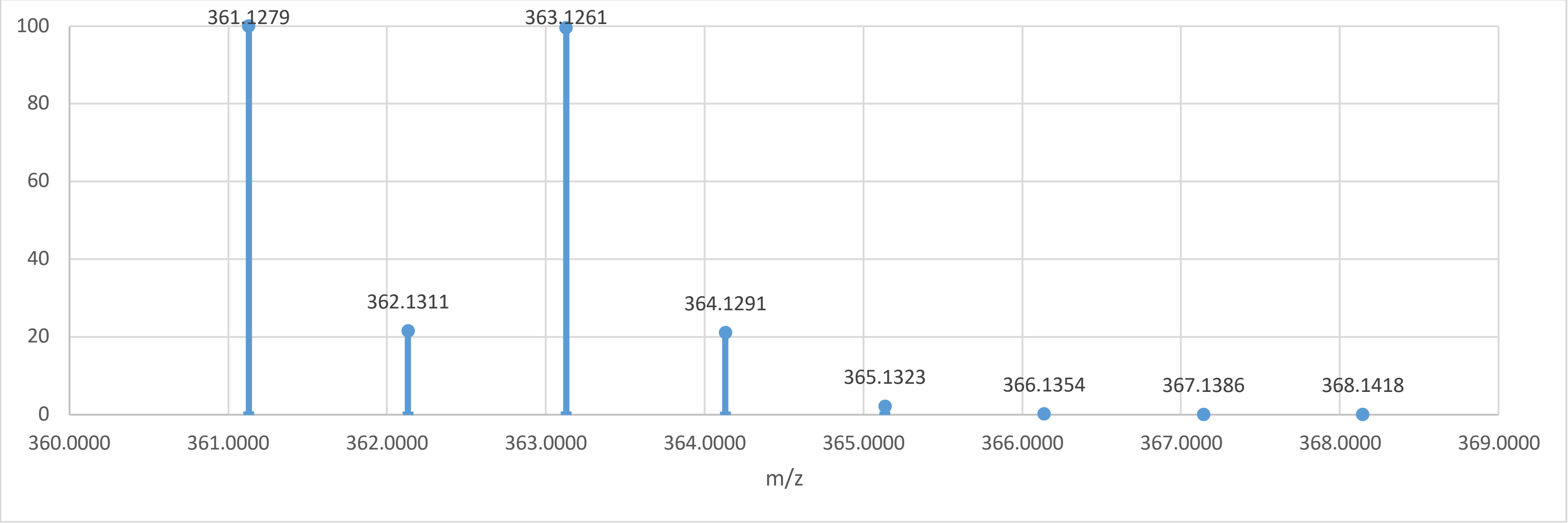

Measured

Monoisotopic Mass, Even Electron Ions
122 formula(e) evaluated with 1 results within limits (up to 50 closest results for each mass)
Elements Used:
C: 0-200 H: 0-200 N: 0-6 Na: 0-1 Br: 0-1

ev-e-hcinn-4br
Qtof_69270 71 (2.694) AM (Cen,5, 80.00, Ar,14000.0,558.36,0.70,LS 5); Sm (SG, 2x5.00); Cm (71:72) 1: TOF MS ES+ 1.49e+003

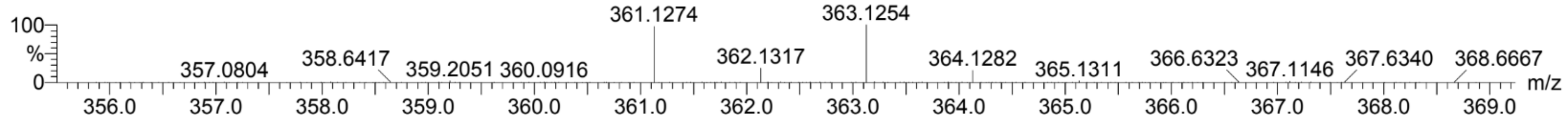

Minimum:				-1.5		
Maximum:		5.0	5.0	100.0		
Mass	Calc. Mass	mDa	PPM	DBE	i-FIT	Formula
361.1274	361.1279	-0.5	-1.4	7.5	2.0	C19 H26 N2 Br

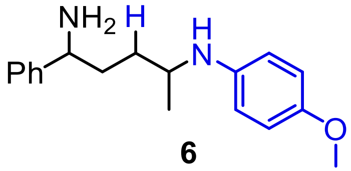

6

HR-MS (ESI-TOF) *m/z*: $[M+H]^+$ calculated for $C_{18}H_{25}N_2O+$, 285.1967; found, 285.1965.

Simulated

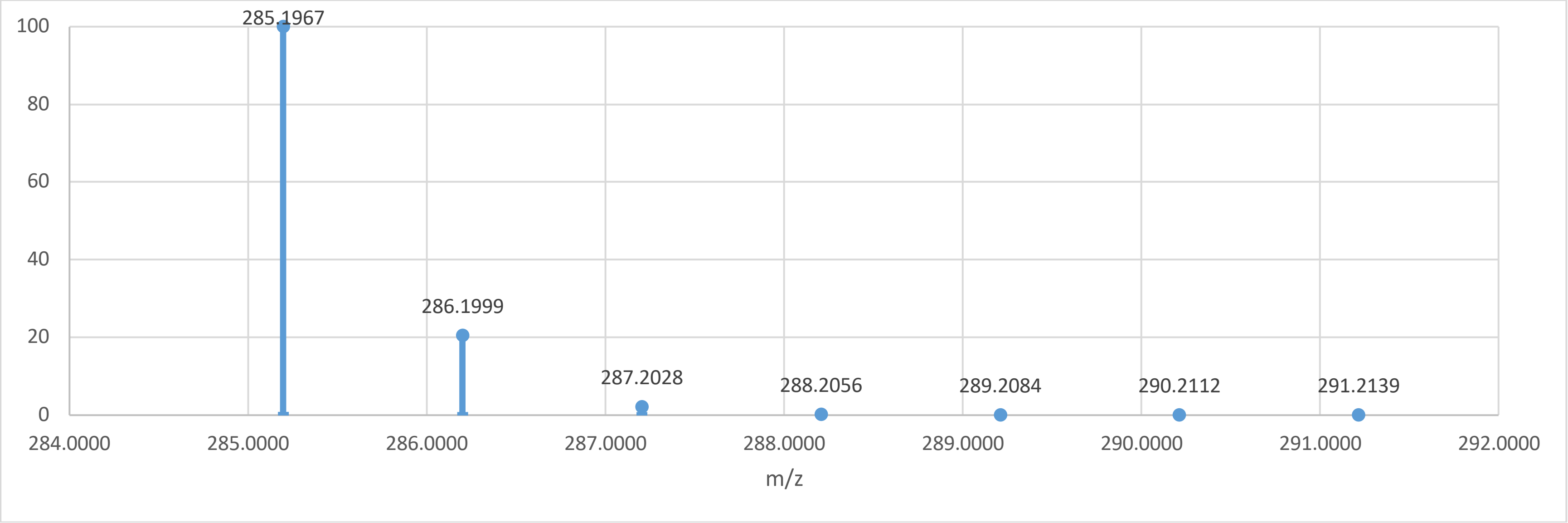

Measured

Monoisotopic Mass, Even Electron Ions
251 formula(e) evaluated with 1 results within limits (up to 50 closest results for each mass)
Elements Used:
C: 0-200 H: 0-200 N: 0-5 O: 0-5 Na: 0-1

1394-z-me-meo
Qtof_68324 57 (2.183) AM (Cen,5, 80.00, Ar,14000.0,558.36,0.70,LS 5); Sm (SG, 2x5.00); Cm (55:57) 1: TOF MS ES+ 5.62e+003

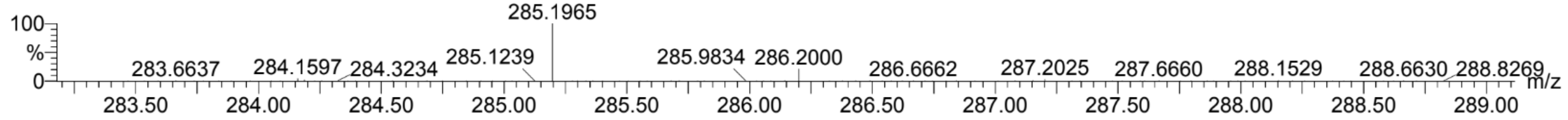

Minimum:				-1.5		
Maximum:		5.0	5.0	100.0		
Mass	Calc. Mass	mDa	PPM	DBE	i-FIT	Formula
285.1965	285.1967	-0.2	-0.7	7.5	2.0	C18 H25 N2 O

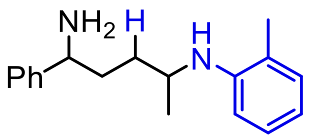

7

HR-MS (ESI-TOF) *m/z*: [M+H]$^+$ calculated for $C_{18}H_{25}N_2$+, 269.2012; found, 269.2017

Simulated

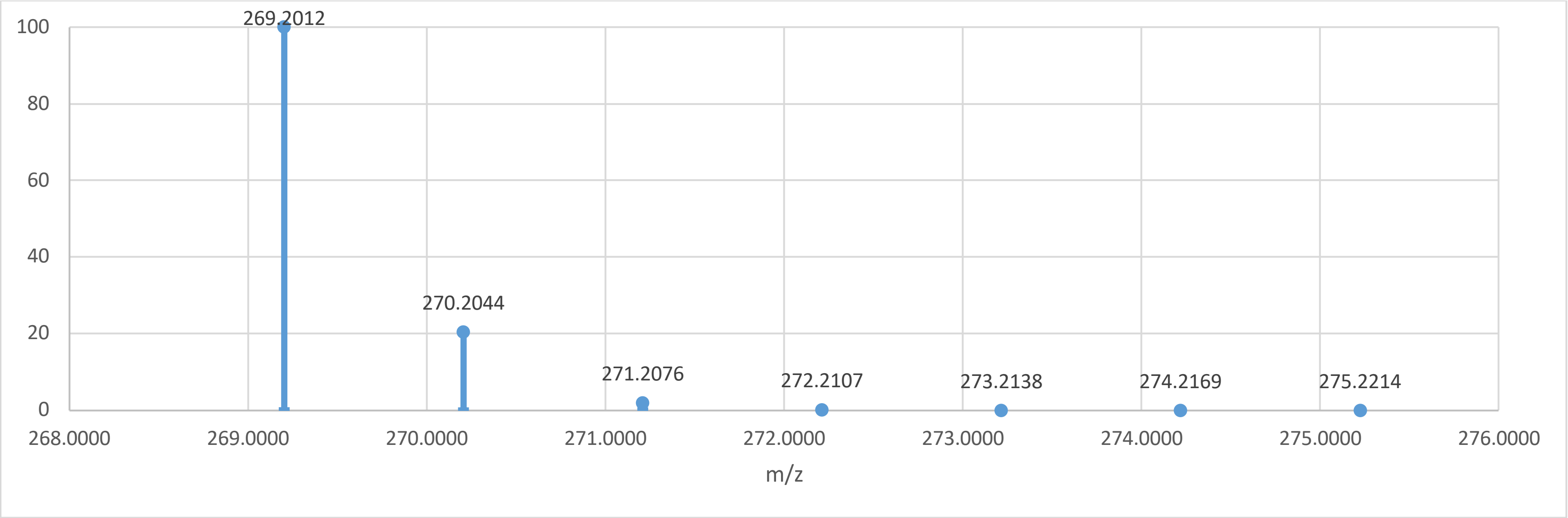

Measured

Monoisotopic Mass, Even Electron Ions
269 formula(e) evaluated with 1 results within limits (up to 50 closest results for each mass)
Elements Used:
C: 0-200 H: 0-200 N: 0-5 O: 0-6 Na: 0-1

Z-Me-oTol2
Qtof_68337 57 (2.183) AM (Cen,5, 80.00, Ar,14000.0,558.36,0.70,LS 5); Sm (SG, 2x5.00); Cm (54:60)

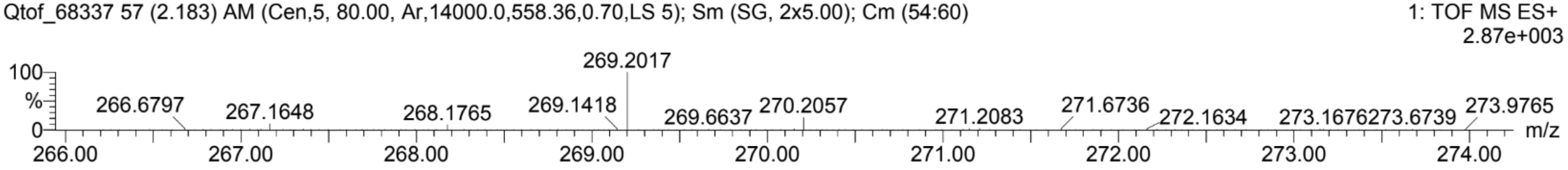

Minimum:				-1.5		
Maximum:		5.0	5.0	100.0		
Mass	Calc. Mass	mDa	PPM	DBE	i-FIT	Formula
269.2017	269.2018	-0.1	-0.4	7.5	1.2	C18 H25 N2

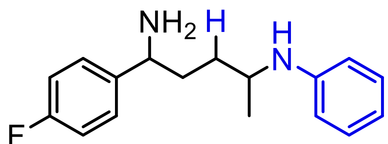

8

HR-MS (ESI-TOF) *m/z*: [M+H]$^+$ calculated for $C_{17}H_{22}FN_2$+, 273.1762; found, 273.1759.

Simulated

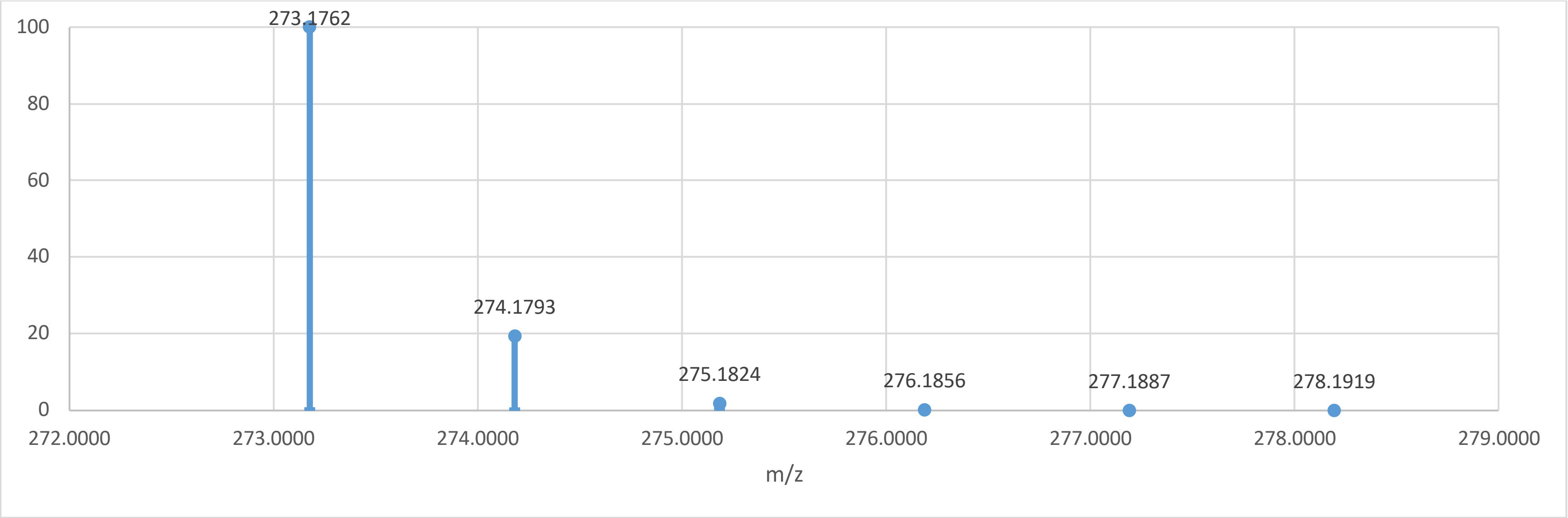

Measured

MS Zoomed Spectrum

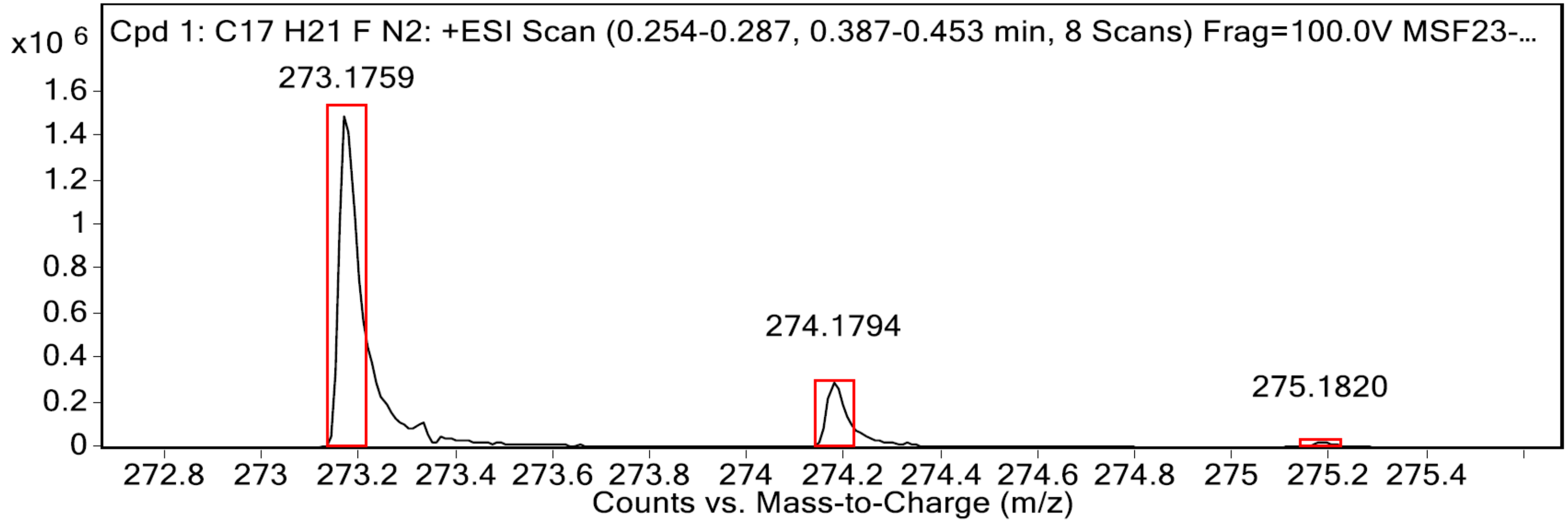

MS Spectrum Peak List

Obs. m/z	Calc. m/z	Charge	Abundance	Formula	Ion Species	Tgt Mass Error (ppm)
273.1759	273.1762	1	1530428	C17H21FN2	(M+H)+	1.07
274.1794	274.1793	1	300359	C17H21FN2	(M+H)+	-0.37
275.1820	275.1824	1	26914	C17H21FN2	(M+H)+	1.61
276.1970	276.1856	1	2327	C17H21FN2	(M+H)+	-41.53

--- End Of Report ---

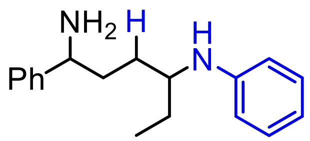

9

HR-MS (ESI-TOF) *m/z*: $[M+H]^+$ calculated for $C_{18}H_{25}N_2^+$, 269.2018; found mass, 269.2019

Simulated

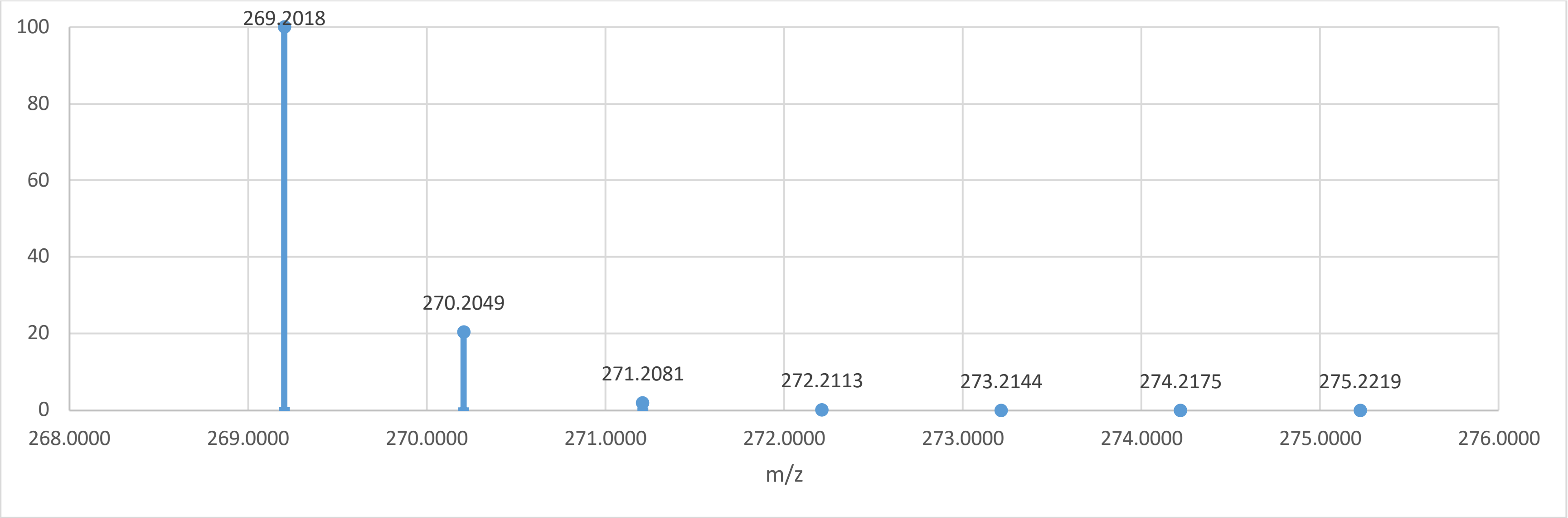

Measured

Monoisotopic Mass, Even Electron Ions
237 formula(e) evaluated with 1 results within limits (up to 50 closest results for each mass)
Elements Used:
C: 0-200   H: 0-200   N: 0-5   O: 0-5   Na: 0-1

1380-z-et-an
Qtof_68320 61 (2.319) AM (Cen,5, 80.00, Ar,14000.0,558.36,0.70,LS 5); Sm (SG, 2x5.00); Cm (61:66)

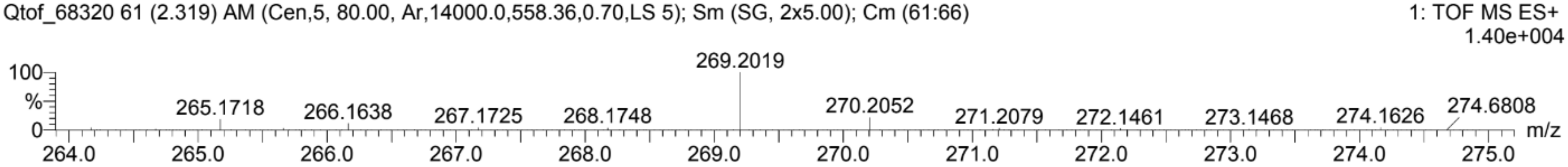

Minimum:				-1.5		
Maximum:		5.0	5.0	100.0		

Mass	Calc. Mass	mDa	PPM	DBE	i-FIT	Formula
269.2019	269.2018	0.1	0.4	7.5	2.0	C18 H25 N2

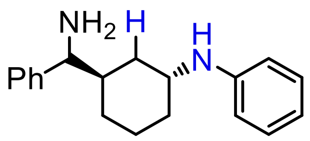

10

HR-MS (ESI-TOF) *m/z*: $[M+H]^+$ calculated for $C_{19}H_{25}N_2^+$, 281.2012; found mass, 281.2013

Simulated

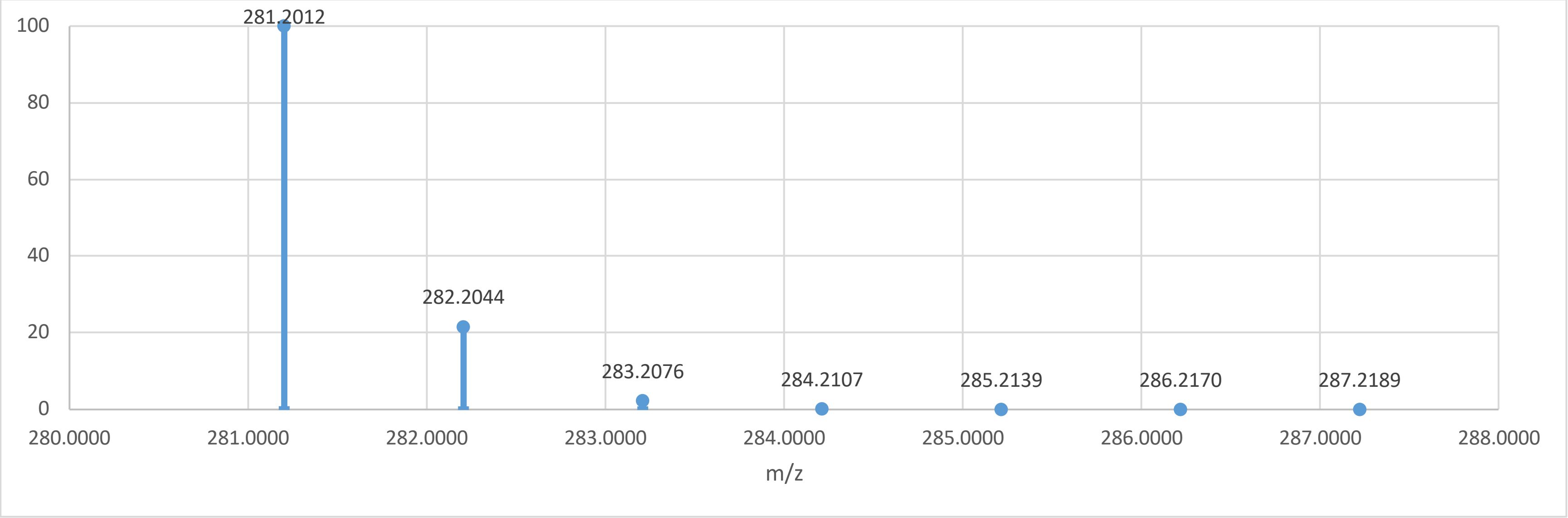

Measured

Monoisotopic Mass, Even Electron Ions
251 formula(e) evaluated with 1 results within limits (up to 50 closest results for each mass)
Elements Used:
C: 0-200 H: 0-200 N: 0-5 O: 0-5 Na: 0-1

ev-e-cyen-an
Qtof_70102 61 (2.319) AM (Cen,5, 80.00, Ar,14000.0,734.47,0.70,LS 5); Sm (SG, 2x5.00); Cm (58:61)

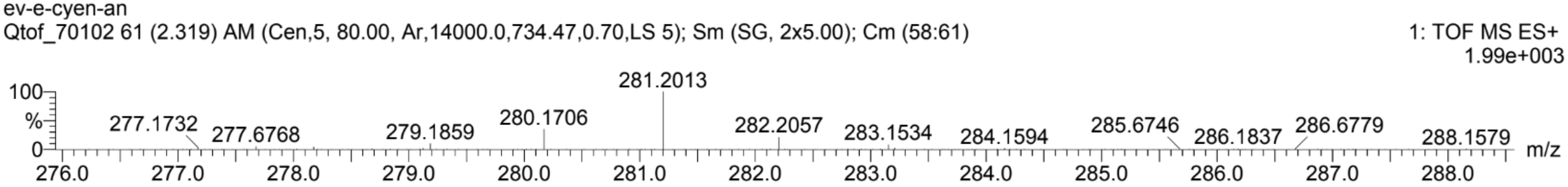

Minimum:				-1.5		
Maximum:		5.0	5.0	100.0		
Mass	Calc. Mass	mDa	PPM	DBE	i-FIT	Formula
281.2013	281.2018	-0.5	-1.8	8.5	2.4	C19 H25 N2

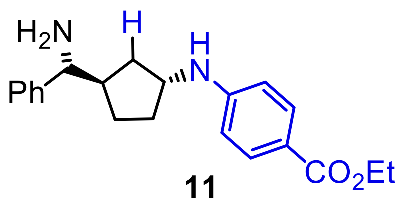

11

HR-MS (ESI-TOF) *m/z*: $[M+H]^+$ calculated for $C_{21}H_{27}N_2O_2^+$, 339.2073; found mass, 339.2071

Simulated

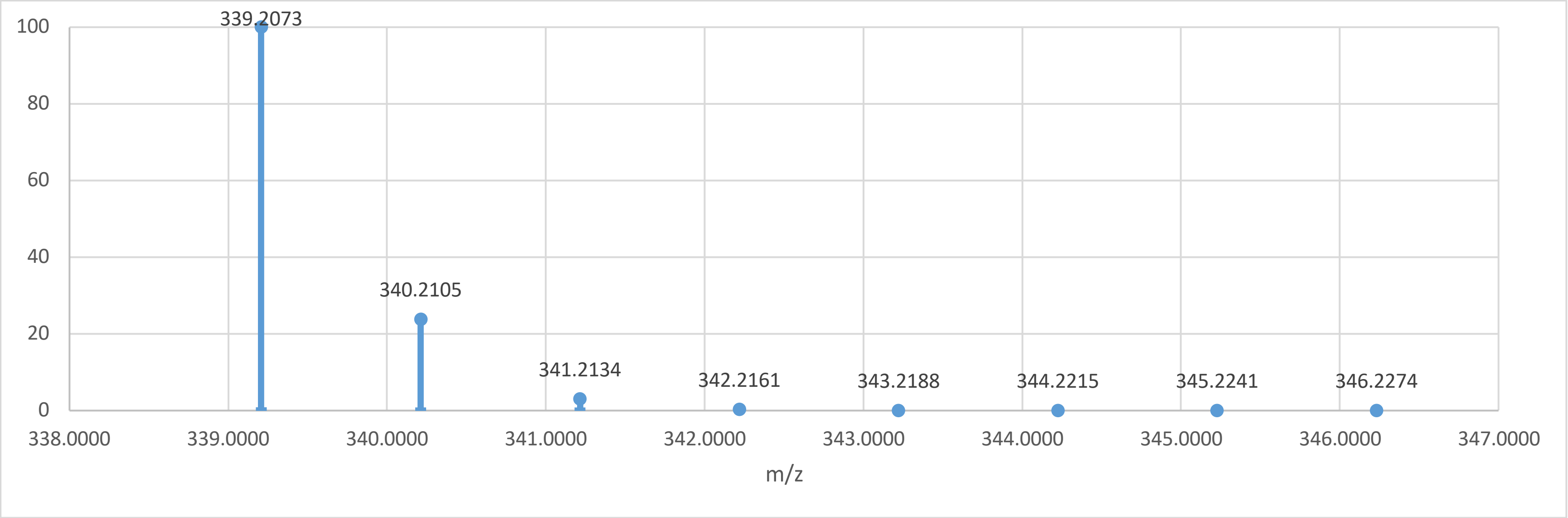

Measured

Monoisotopic Mass, Even Electron Ions
300 formula(e) evaluated with 1 results within limits (up to 50 closest results for each mass)
Elements Used:
C: 0-200 H: 0-200 N: 0-5 O: 0-5 Na: 0-1

cyp-ester
Qtof_68332 50 (1.911) AM (Cen,5, 80.00, Ar,14000.0,558.36,0.70,LS 5); Sm (SG, 2x5.00); Cm (48:50)   1: TOF MS ES+
5.51e+003

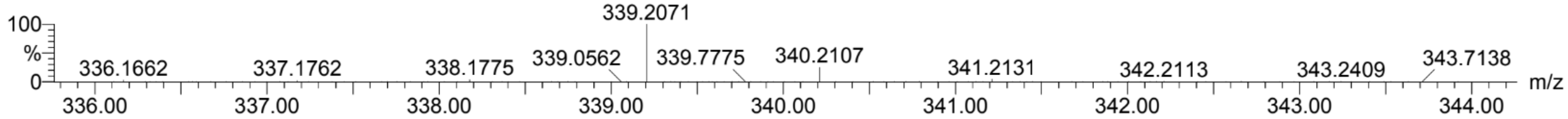

Mass	Calc. Mass	mDa	PPM	DBE	i-FIT	Formula
Minimum:				-1.5		
Maximum:		5.0	5.0	100.0		
339.2071	339.2073	-0.2	-0.6	9.5	6.1	C21 H27 N2 O2

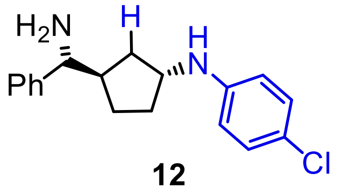

12

HR-MS (ESI-TOF) *m/z*: $[M+H]^+$ calculated for $C_{18}H_{22}N_2Cl^+$, 301.1466; found mass, 301.1465

Simulated

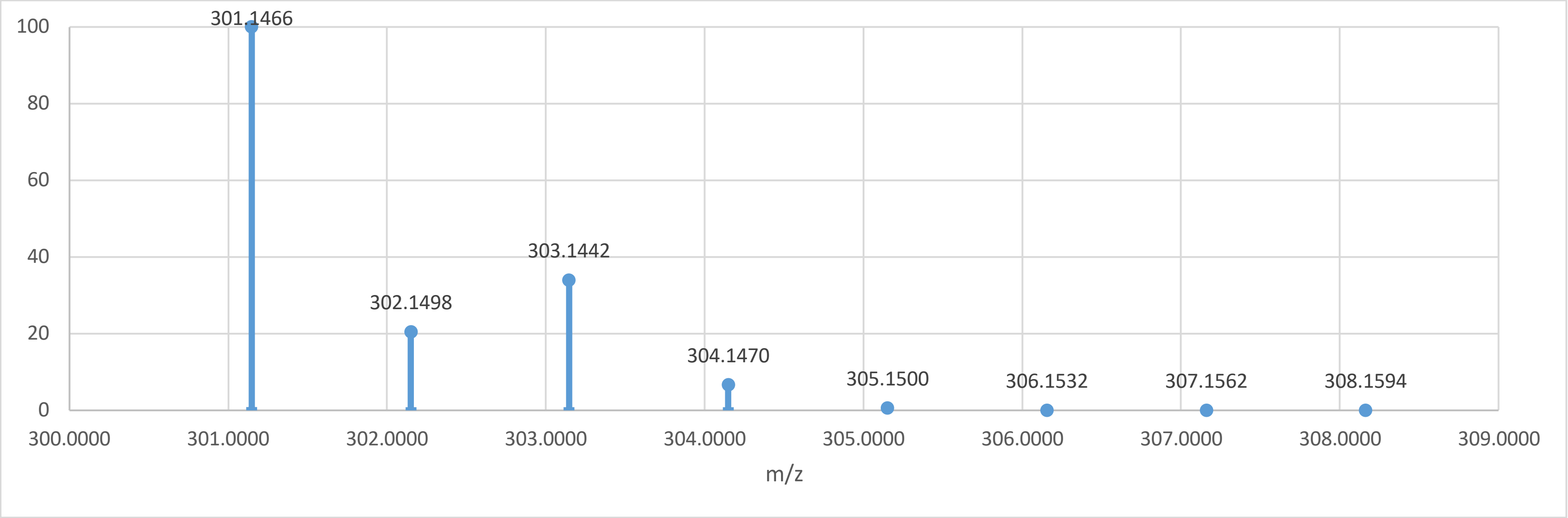

Measured

Monoisotopic Mass, Even Electron Ions
500 formula(e) evaluated with 2 results within limits (up to 50 closest results for each mass)
Elements Used:
C: 0-200 H: 0-200 N: 0-5 O: 0-5 Na: 0-1 Cl: 0-1

cyp-4cl
Qtof_68330 59 (2.251) AM (Cen,5, 80.00, Ar,14000.0,558.36,0.70,LS 5); Sm (SG, 2x5.00); Cm (55:63)

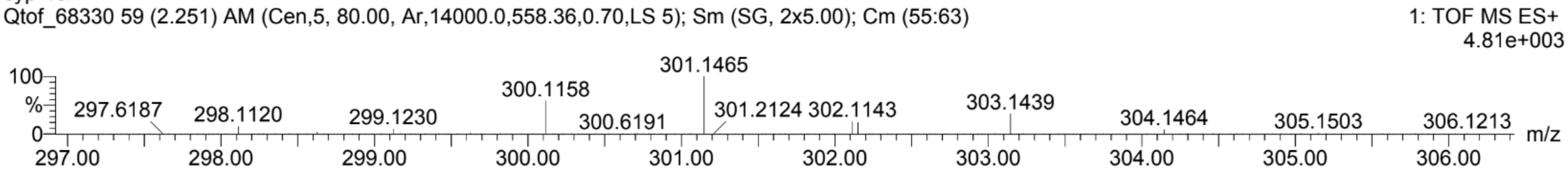

Minimum:				-1.5		
Maximum:		5.0	5.0	100.0		

Mass	Calc. Mass	mDa	PPM	DBE	i-FIT	Formula
301.1465	301.1472	-0.7	-2.3	8.5	3.9	C18 H22 N2 Cl
	301.1453	1.2	4.0	13.5	741.6	C19 H17 N4

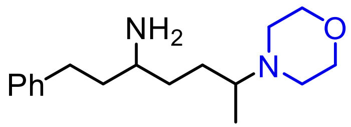

17

HR-MS (ESI-TOF) *m/z*: [M+H]$^+$ calculated for $C_{17}H_{29}N_2O^+$, 277.2274; found mass, 277.2267.

Simulated

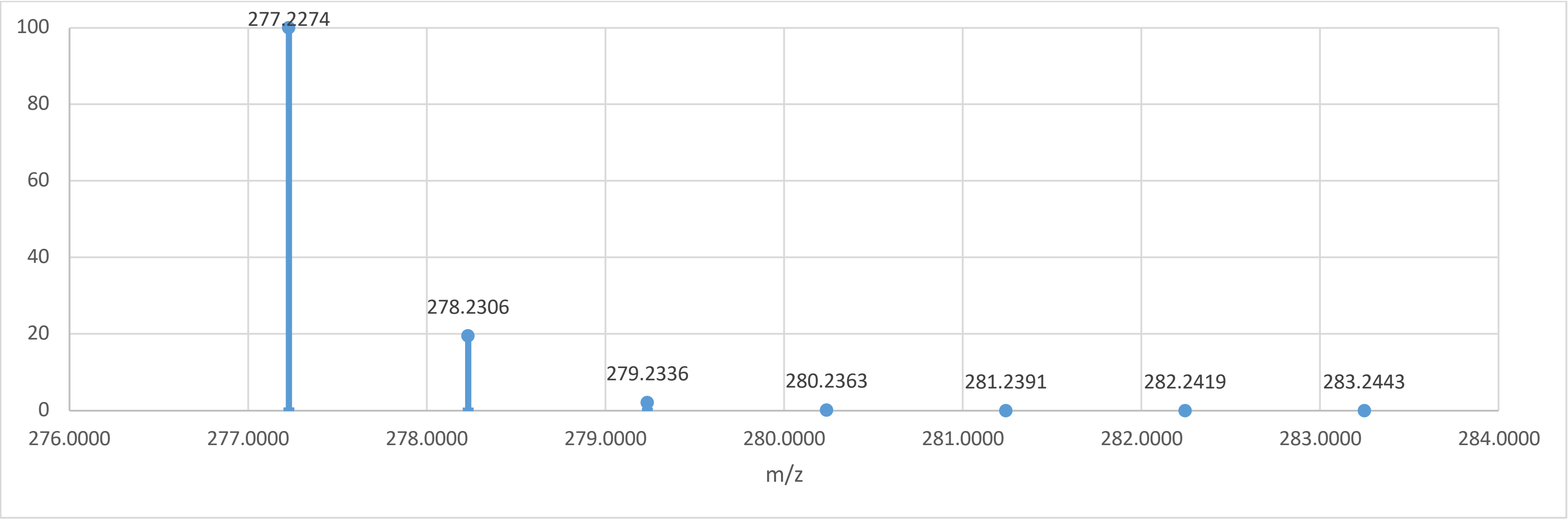

Measured

Monoisotopic Mass, Even Electron Ions
245 formula(e) evaluated with 2 results within limits (up to 50 closest results for each mass)
Elements Used:
C: 0-200 H: 0-200 N: 0-5 O: 0-5 Na: 0-1
1392-morph-hcinn
Qtof_68322 71 (2.693) AM (Cen,5, 80.00, Ar,14000.0,558.36,0.70,LS 5); Sm (SG, 2x5.00); Cm (71:72)

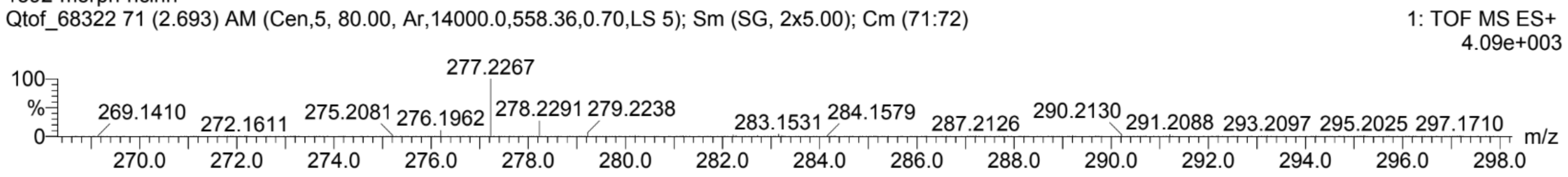

Mass	Calc. Mass	mDa	PPM	DBE	i-FIT	Formula
Minimum:				-1.5		
Maximum:		5.0	5.0	100.0		
277.2267	277.2256	1.1	4.0	1.5	114.8	C15 H30 N2 O Na
	277.2280	-1.3	-4.7	4.5	80.2	C17 H29 N2 O

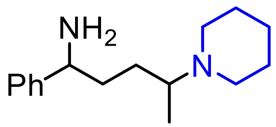

18

HR-MS (ESI-TOF) *m/z*: $[M+H]^+$ calculated for $C_{16}H_{27}N_2^+$, 247.2174; found mass, 247.2176.

Simulated

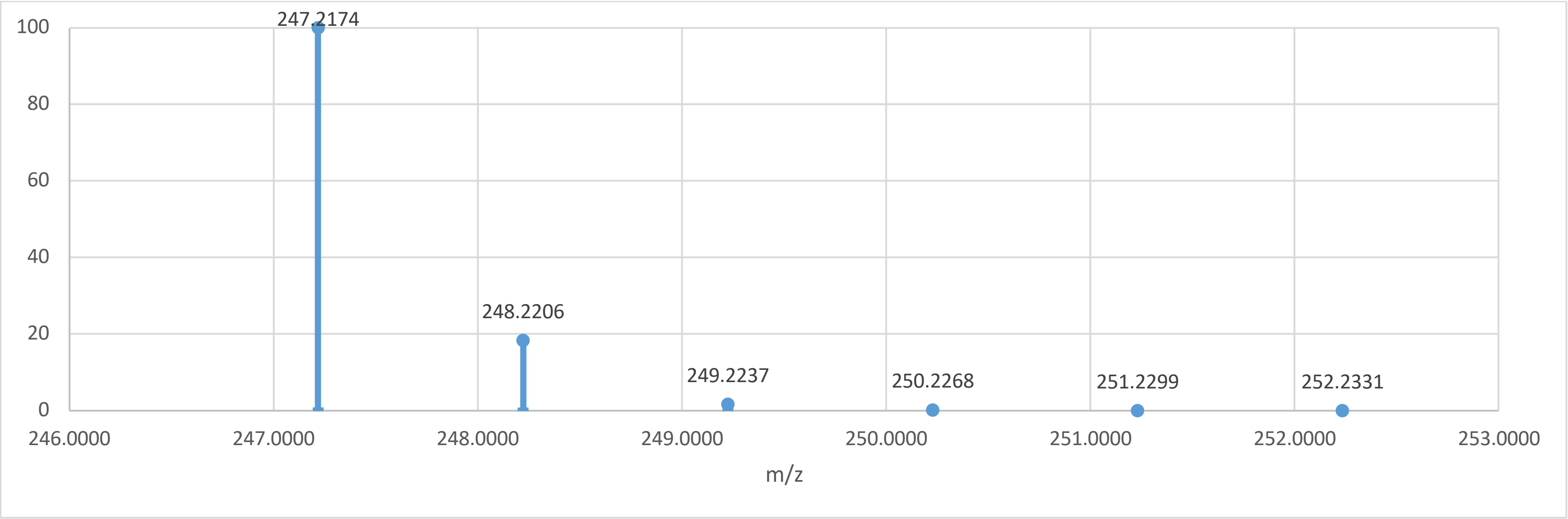

Measured

MS Zoomed Spectrum

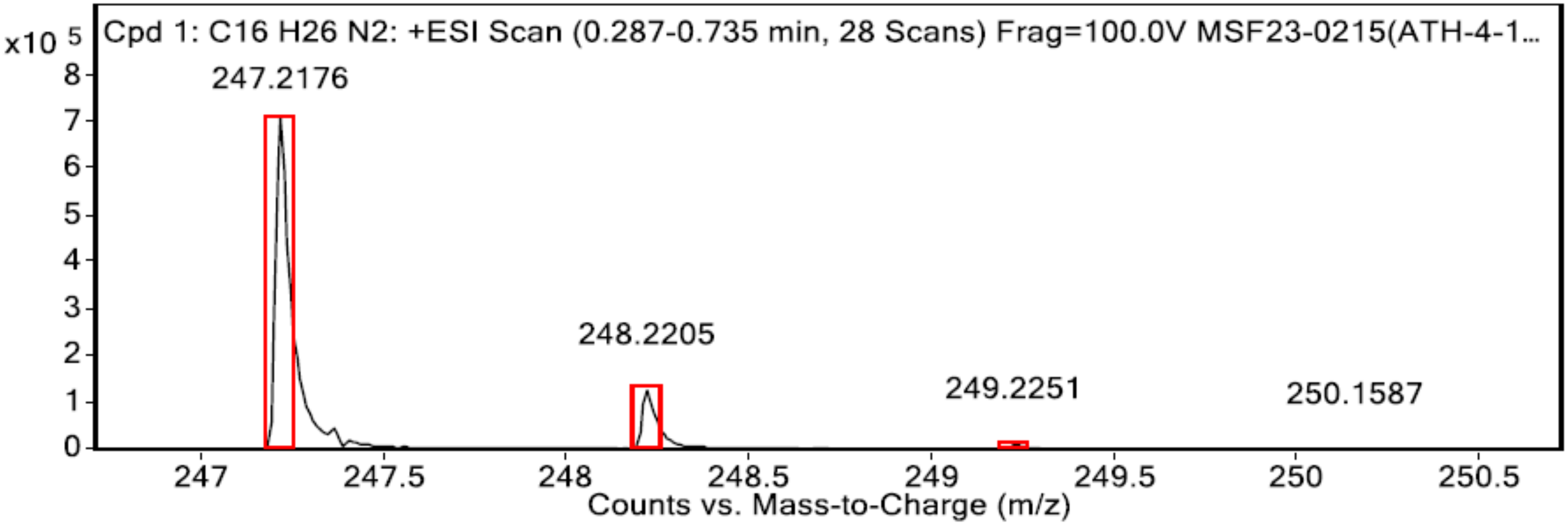

MS Spectrum Peak List

Obs. m/z	Calc. m/z	Charge	Abundance	Formula	Ion Species	Tgt Mass Error (ppm)
247.2176	247.2169	1	713338	C16H26N2	(M+H)+	-2.85
248.2205	248.2200	1	129202	C16H26N2	(M+H)+	-1.79
249.2251	249.2232	1	13058	C16H26N2	(M+H)+	-7.69
250.1587	250.2263	1	1486	C16H26N2	(M+H)+	270.12

--- End Of Report ---

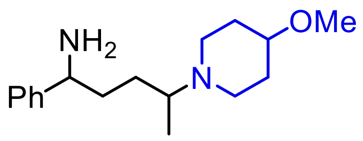

19

HR-MS (ESI-TOF) *m/z*: $[M+H]^+$ calculated for $C_{17}H_{29}N_2O^+$, 277.2274; found mass, 277.2277

Simulated

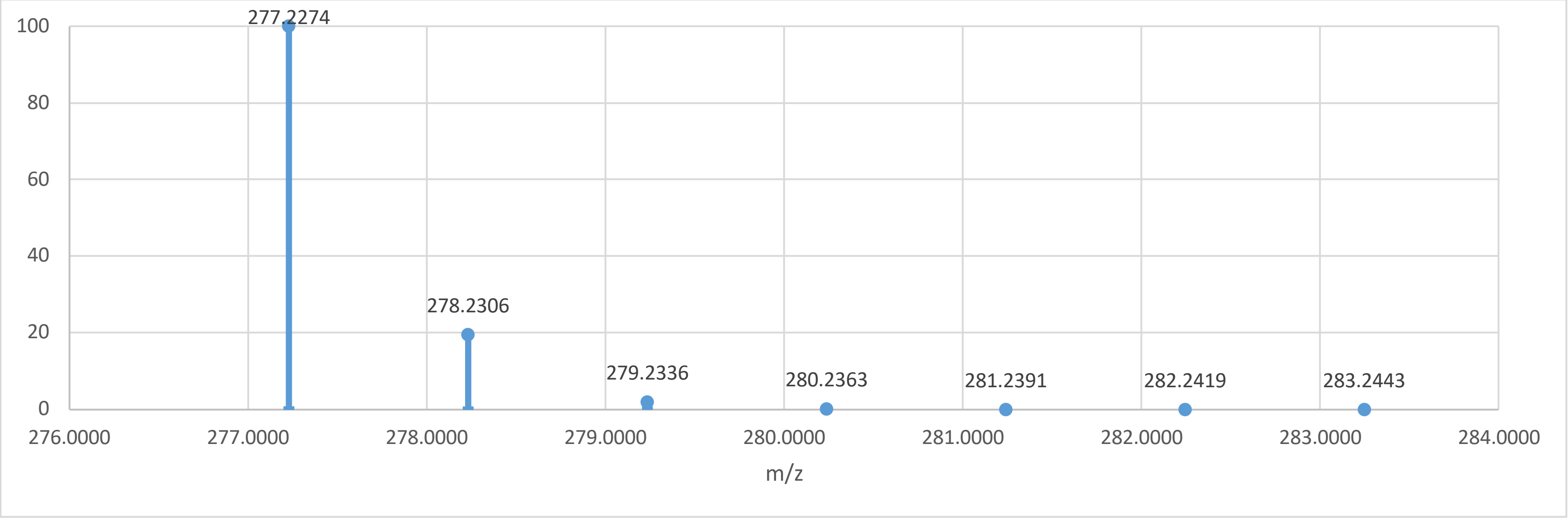

Measured

MS Zoomed Spectrum

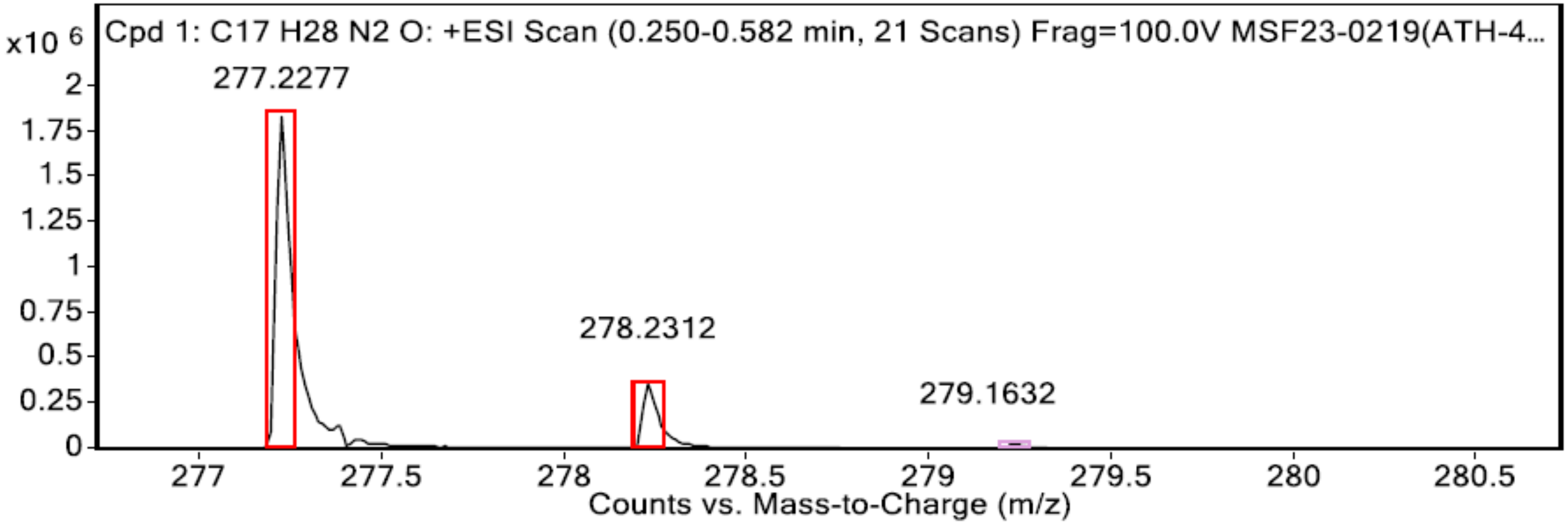

MS Spectrum Peak List

Obs. m/z	Calc. m/z	Charge	Abundance	Formula	Ion Species	Tgt Mass Error (ppm)
277.2277	277.2274	1	1854404	C17H28N2O	(M+H)+	-0.95
278.2312	278.2306	1	362767	C17H28N2O	(M+H)+	-2.01
279.1632	279.2336	1	1161	C17H28N2O	(M+H)+	252.03

--- End Of Report ---

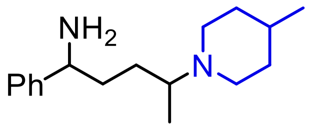

20

HR-MS (ESI-TOF) *m/z*: $[M+H]^+$ calculated for $C_{17}H_{29}N_2^+$, 261.2325; found mass, 261.2326

Simulated

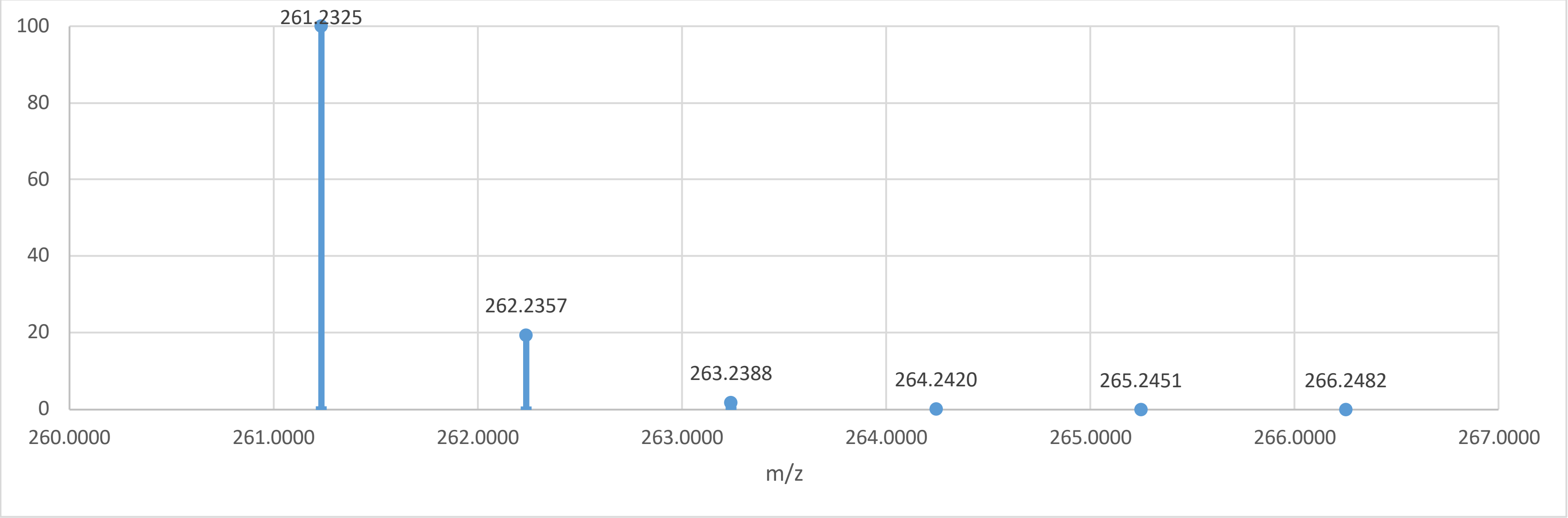

Measured

MS Zoomed Spectrum

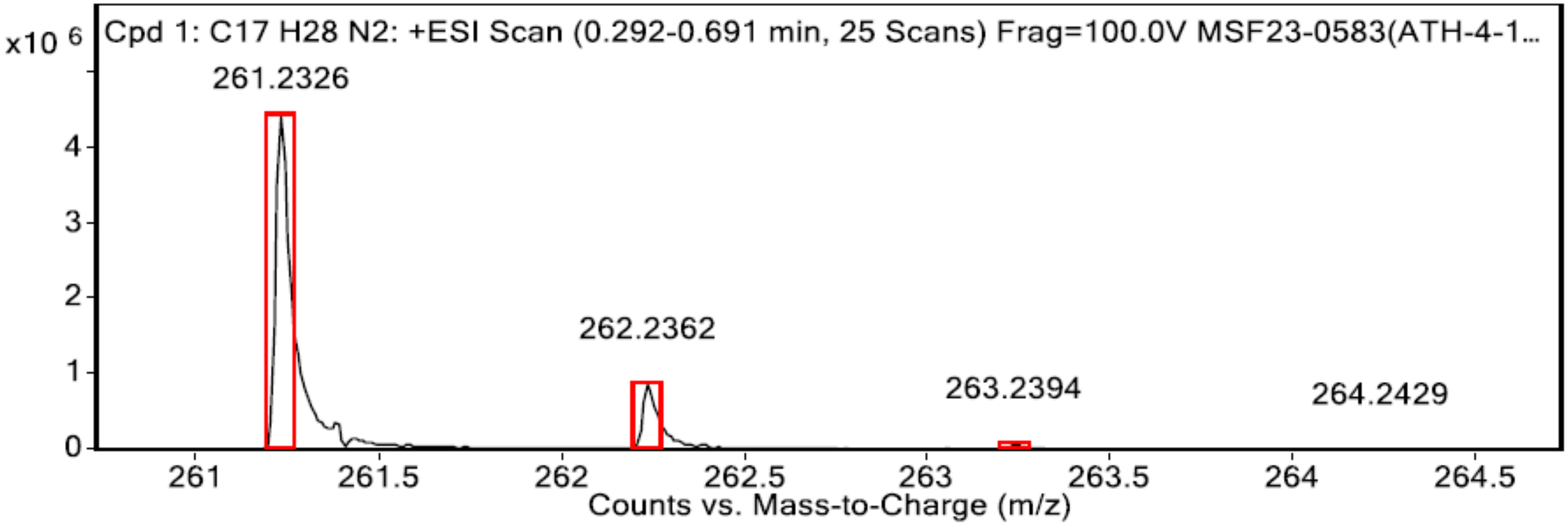

MS Spectrum Peak List

Obs. m/z	Calc. m/z	Charge	Abundance	Formula	Ion Species	Tgt Mass Error (ppm)
261.2326	261.2325	1	4427268	C17H28N2	(M+H)+	-0.35
262.2362	262.2357	1	877679	C17H28N2	(M+H)+	-2.03
263.2394	263.2388	1	83968	C17H28N2	(M+H)+	-2.04
264.2429	264.2420	1	4643	C17H28N2	(M+H)+	-3.32

--- End Of Report ---

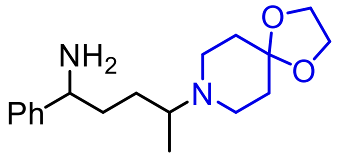

21

HR-MS (ESI-TOF) *m/z*: $[M+H]^+$ calculated for $C_{18}H_{29}N_2O_2^+$, 305.2224; found mass, 305.2230.

Simulated

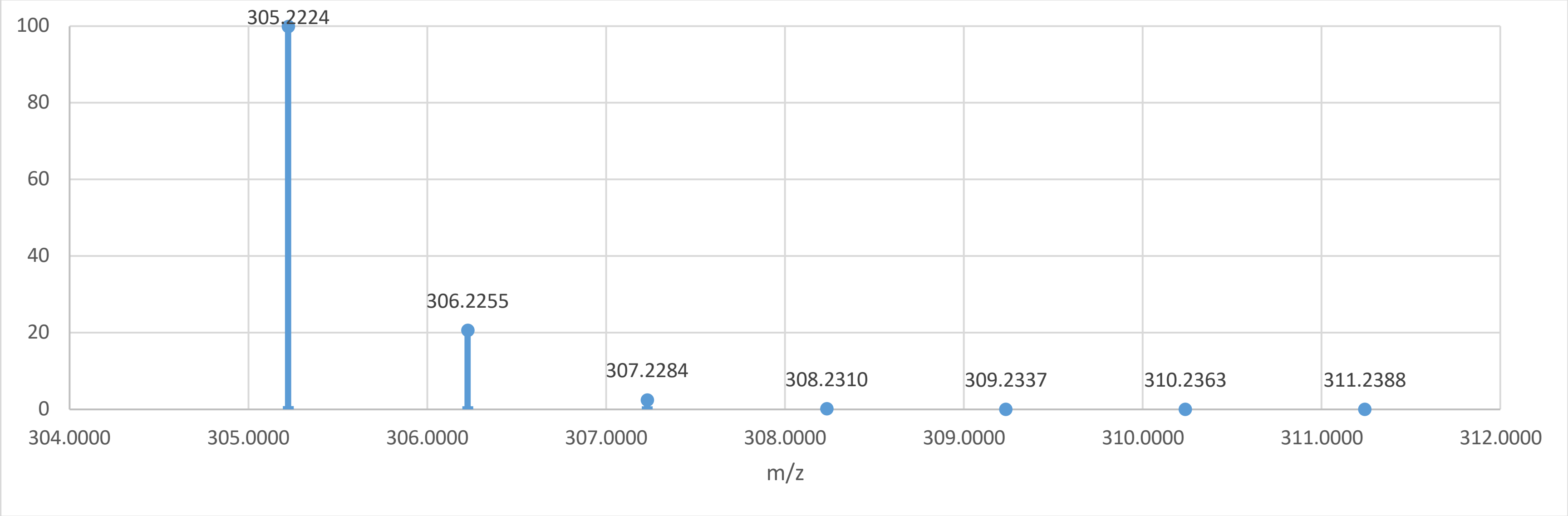

Measured

MS Zoomed Spectrum

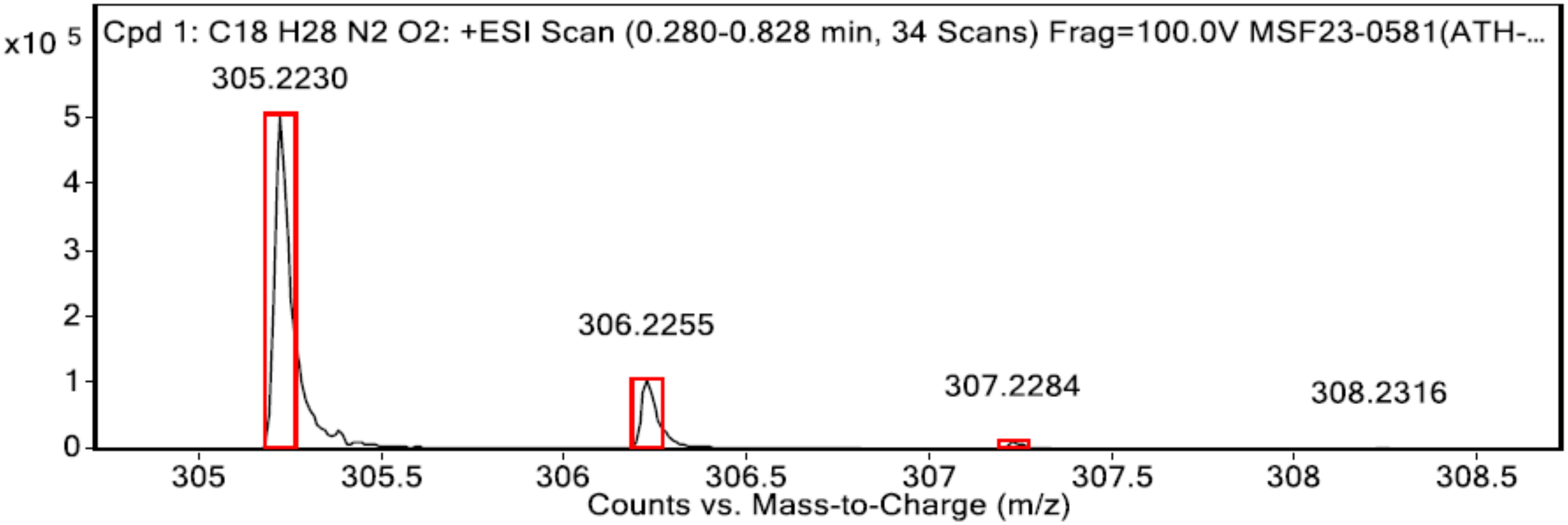

MS Spectrum Peak List

Obs. m/z	Calc. m/z	Charge	Abundance	Formula	Ion Species	Tgt Mass Error (ppm)
144.1020			1712258			
305.2230	305.2224	1	504905	C18H28N2O2	(M+H)+	-2.02
306.2255	306.2255	1	105557	C18H28N2O2	(M+H)+	0.04
307.2284	307.2284	1	12823	C18H28N2O2	(M+H)+	-0.14
308.2316	308.2310	1	2389	C18H28N2O2	(M+H)+	-1.75

--- End Of Report ---

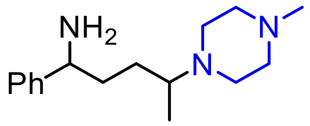

22

HR-MS (ESI-TOF) *m/z*: [M+H]$^+$ calculated for $C_{16}H_{28}N_3^+$, 262.2278; found mass, 262.2287.

Simulated

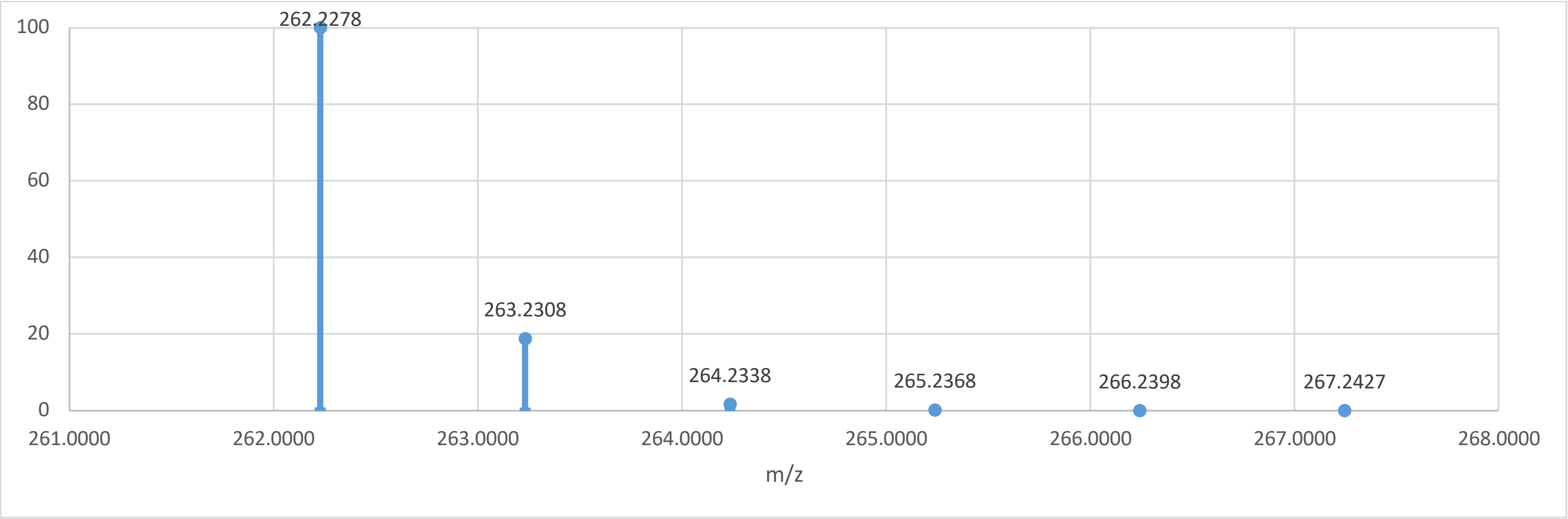

Measured

MS Zoomed Spectrum

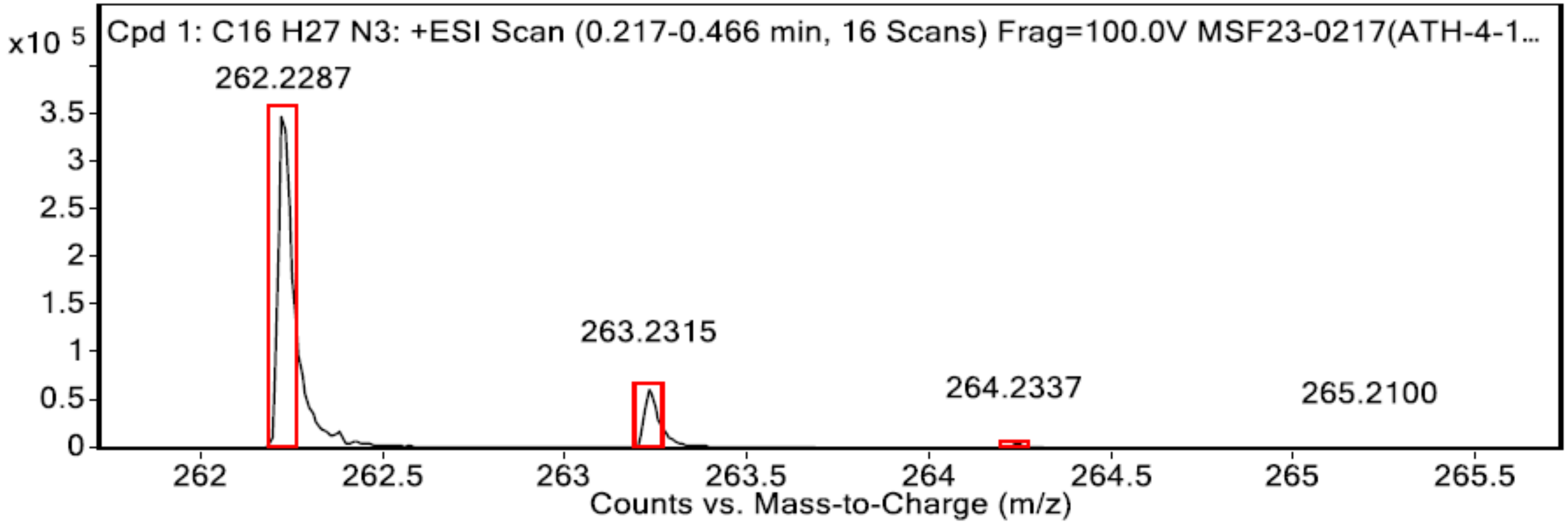

MS Spectrum Peak List

Obs. m/z	Calc. m/z	Charge	Abundance	Formula	Ion Species	Tgt Mass Error (ppm)
262.2287	262.2278	1	359567	C16H27N3	(M+H)+	-3.69
263.2315	263.2308	1	64990	C16H27N3	(M+H)+	-2.55
264.2337	264.2338	1	6170	C16H27N3	(M+H)+	0.32
265.2100	265.2368	1	459	C16H27N3	(M+H)+	101.12

--- End Of Report ---

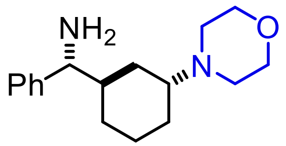

23

29% yield

>20:1 dr

HR-MS (ESI-TOF) *m/z*: $[M+H]^+$ calculated for $C_{17}H_{27}N_2O^+$, 275.2118; found mass = 275.2117

Simulated

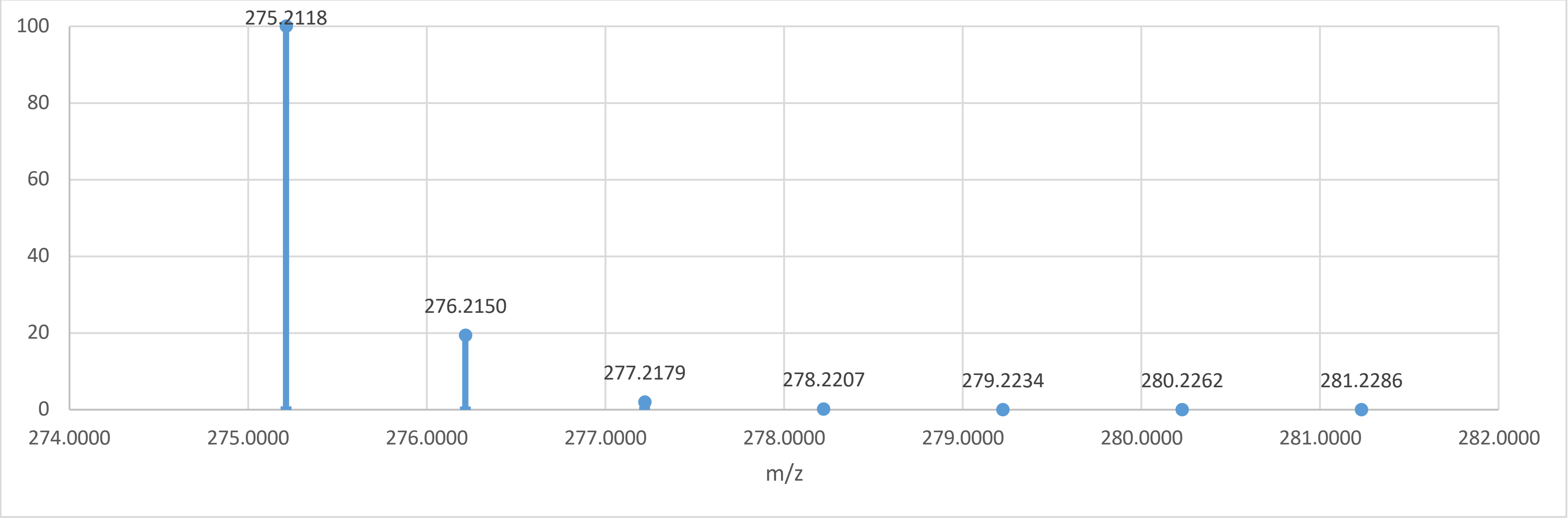

Measured

Monoisotopic Mass, Even Electron Ions
245 formula(e) evaluated with 1 results within limits (up to 50 closest results for each mass)
Elements Used:
C: 0-200 H: 0-200 N: 0-5 O: 0-5 Na: 0-1

cyen-morph
Qtof_68328 57 (2.183) AM (Cen,5, 80.00, Ar,14000.0,558.36,0.70,LS 5); Sm (SG, 2x5.00); Cm (55:57)

1: TOF MS ES+
2.99e+003

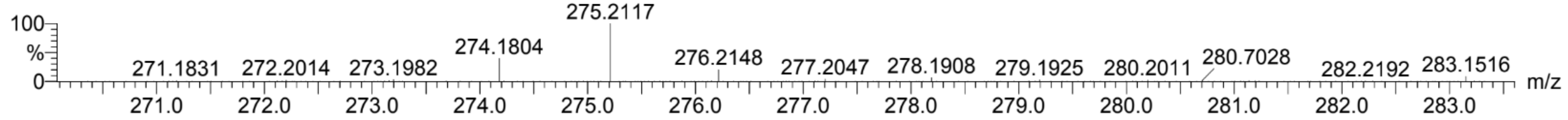

Minimum:				-1.5		
Maximum:		5.0	5.0	100.0		

Mass	Calc. Mass	mDa	PPM	DBE	i-FIT	Formula
275.2117	275.2123	-0.6	-2.2	5.5	9.7	C17 H27 N2 O

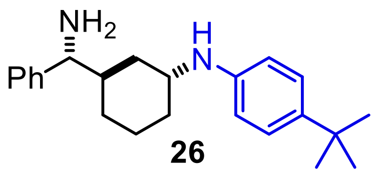

26

HR-MS (ESI-TOF) *m/z*: $[M+Na]^+$ calculated for $C_{23}H_{32}N_2Na^+$, 359.2458; found mass, 359.2461

Simulated

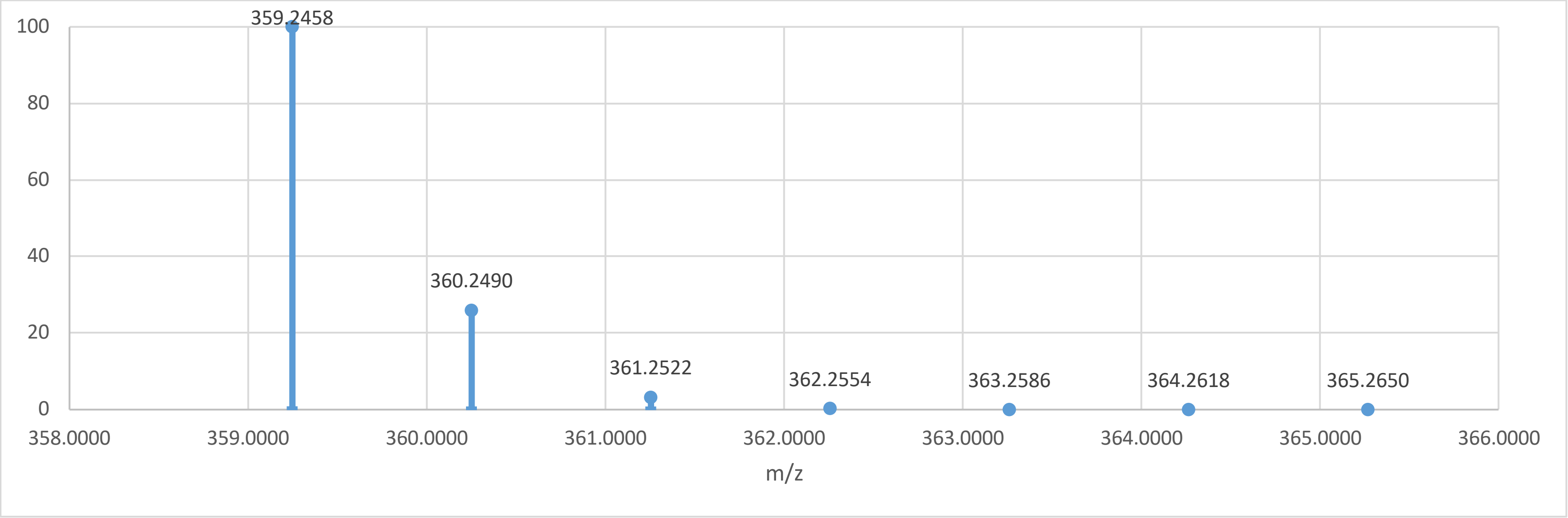

Measured

MS Zoomed Spectrum

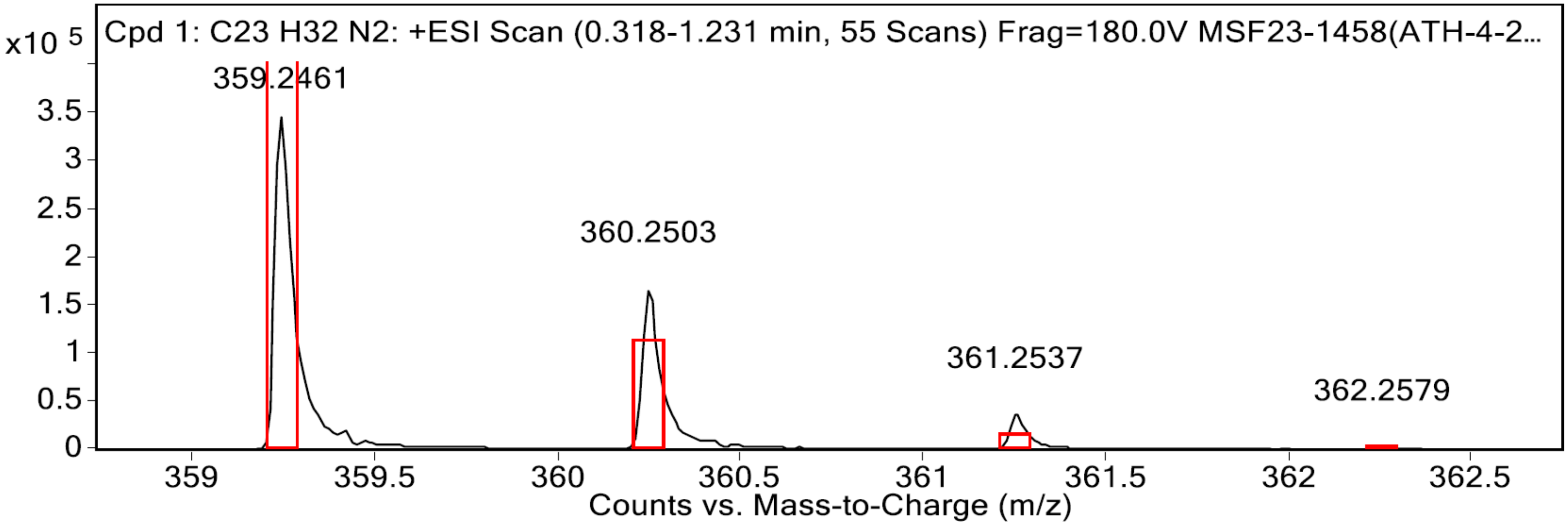

MS Spectrum Peak List

Obs. m/z	Calc. m/z	Charge	Abundance	Formula	Ion Species	Tgt Mass Error (ppm)
320.2368			2726235			
359.2461	359.2458	1	346872	C23H32N2	(M+Na)+	-0.85
360.2503	360.2490	1	169485	C23H32N2	(M+Na)+	-3.53
361.2537	361.2522	1	38309	C23H32N2	(M+Na)+	-4.11
362.2579	362.2554	1	4415	C23H32N2	(M+Na)+	-7.01

--- End Of Report ---

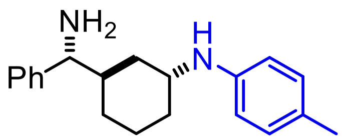

27

HR-MS (ESI-TOF) *m/z*: [M+Na]$^+$ calculated for $C_{20}H_{26}N_2Na^+$, 317.1988; found mass, 317.1984

Simulated

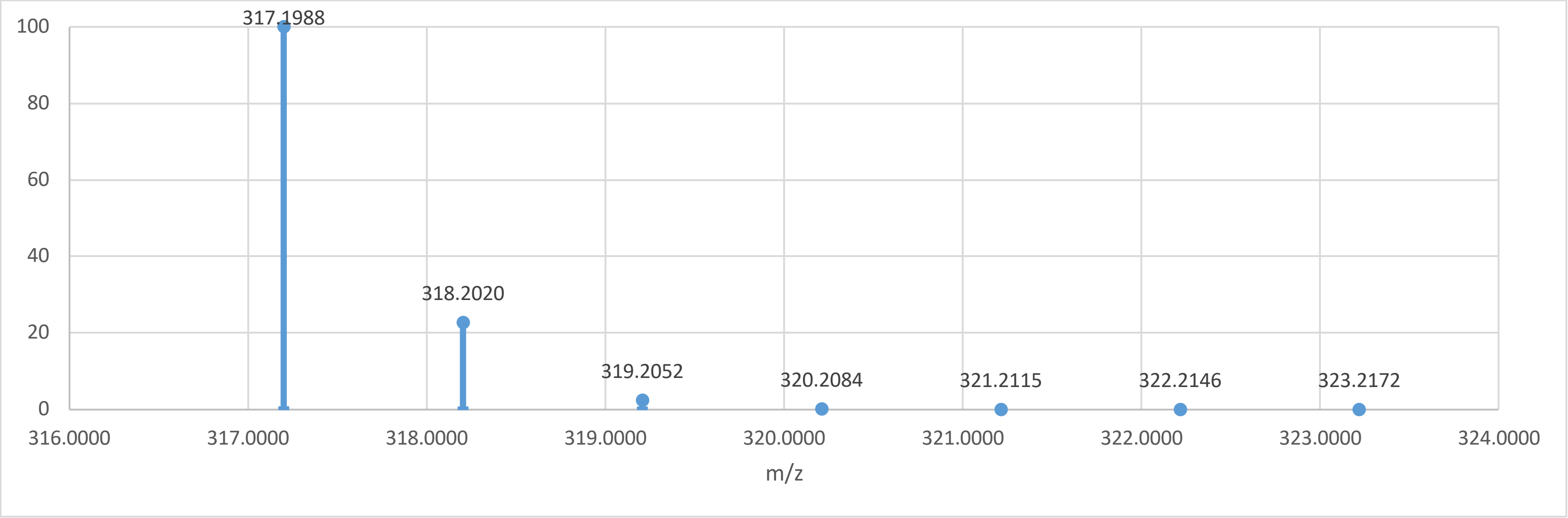

Measured

MS Zoomed Spectrum

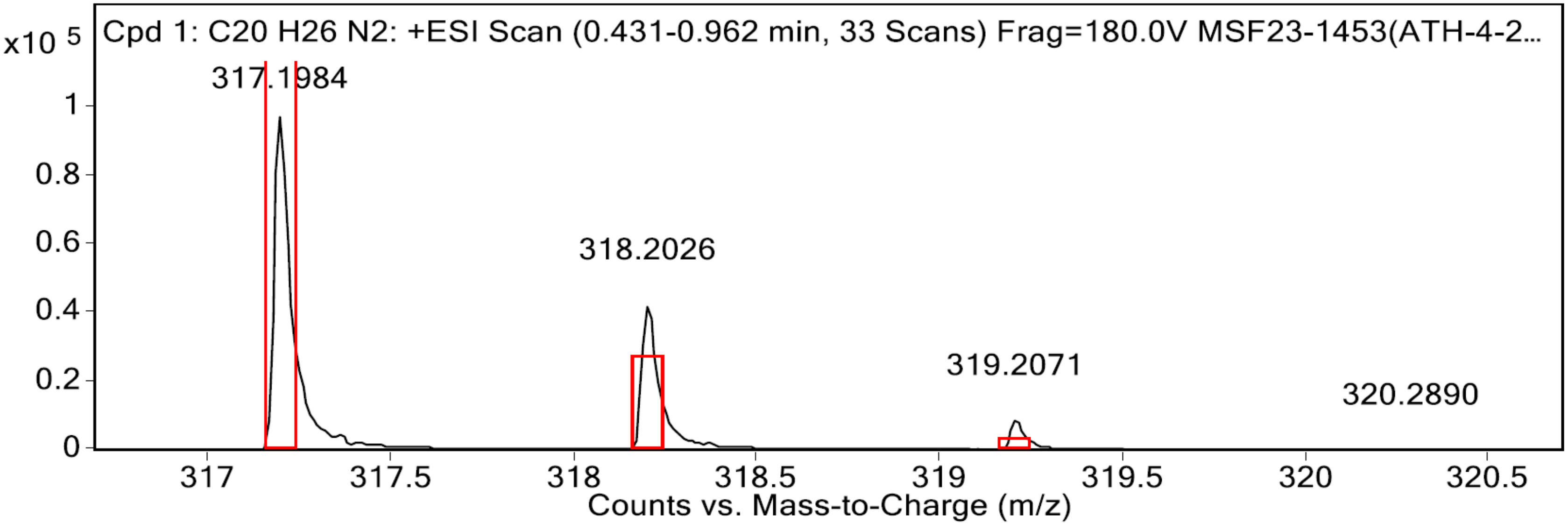

MS Spectrum Peak List

Obs. m/z	Calc. m/z	Charge	Abundance	Formula	Ion Species	Tgt Mass Error (ppm)
278.1898			1259193			
317.1984	317.1988	1	97266	C20H26N2	(M+Na)+	1.38
318.2026	318.2020	1	42477	C20H26N2	(M+Na)+	-1.88
319.2071	319.2052	1	8702	C20H26N2	(M+Na)+	-6.1
320.2890	320.2084	1	69	C20H26N2	(M+Na)+	-251.79

--- End Of Report ---

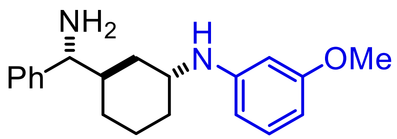

28

HR-MS (ESI-TOF) *m/z*: [M+Na]$^+$ calculated for $C_{20}H_{26}N_2ONa^+$, 333.1937; found mass, 333.1941

Simulated

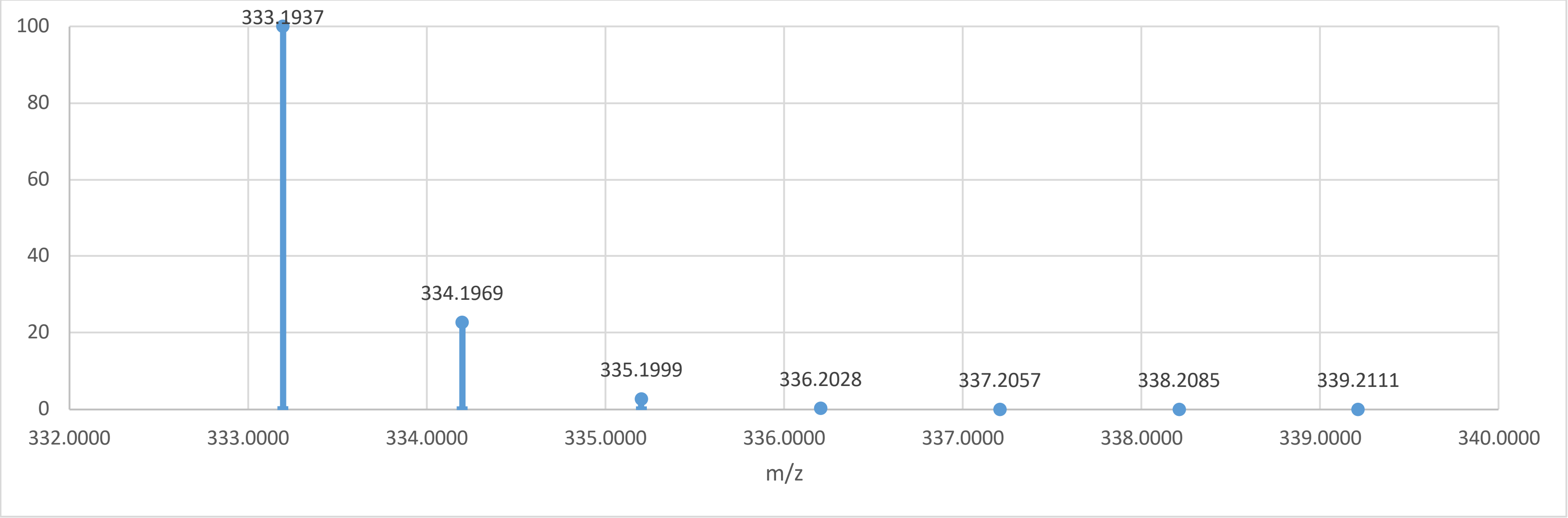

Measured

MS Zoomed Spectrum

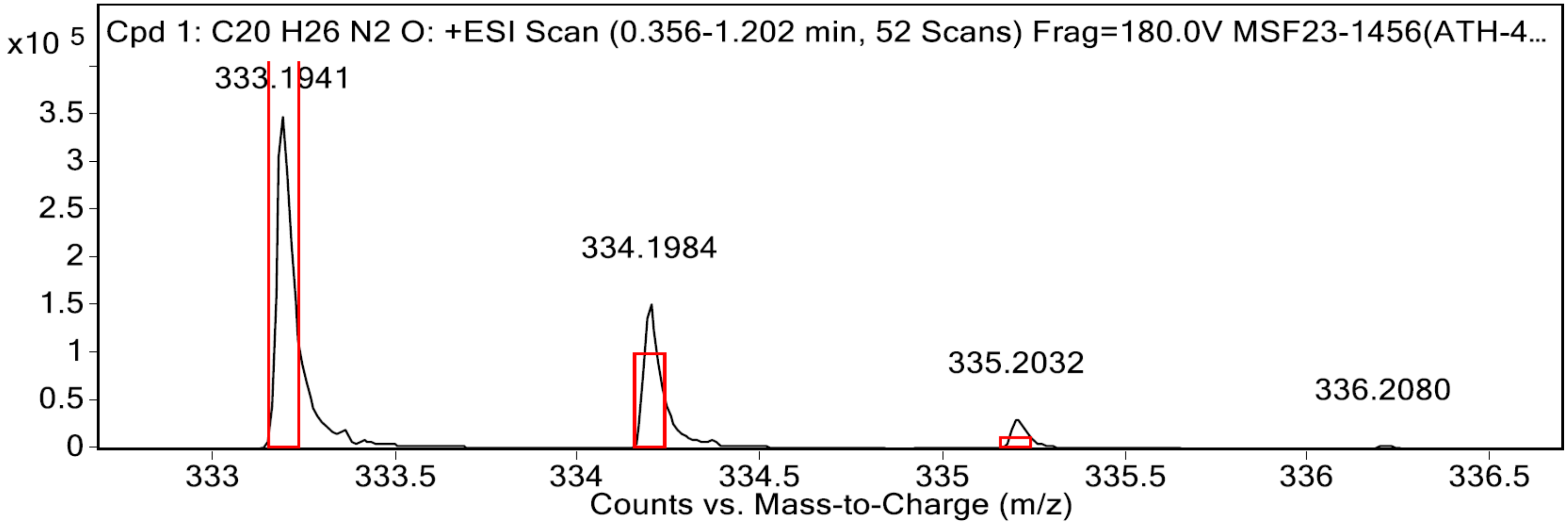

MS Spectrum Peak List

Obs. m/z	Calc. m/z	Charge	Abundance	Formula	Ion Species	Tgt Mass Error (ppm)
294.1847			3007993			
333.1941	333.1937	1	347719	C20H26N2O	(M+Na)+	-1.25
334.1984	334.1969	1	152979	C20H26N2O	(M+Na)+	-4.33
335.2032	335.1999	1	32556	C20H26N2O	(M+Na)+	-9.68
336.2080	336.2028	1	4433	C20H26N2O	(M+Na)+	-15.48

--- End Of Report ---

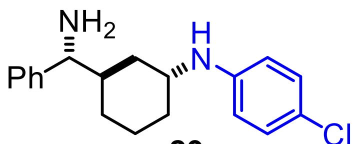

29

HR-MS (ESI-TOF) *m/z*: $[M+H]^+$ calculated for $C_{19}H_{24}ClN_2^+$, 315.1623; found mass, 315.1617

Simulated

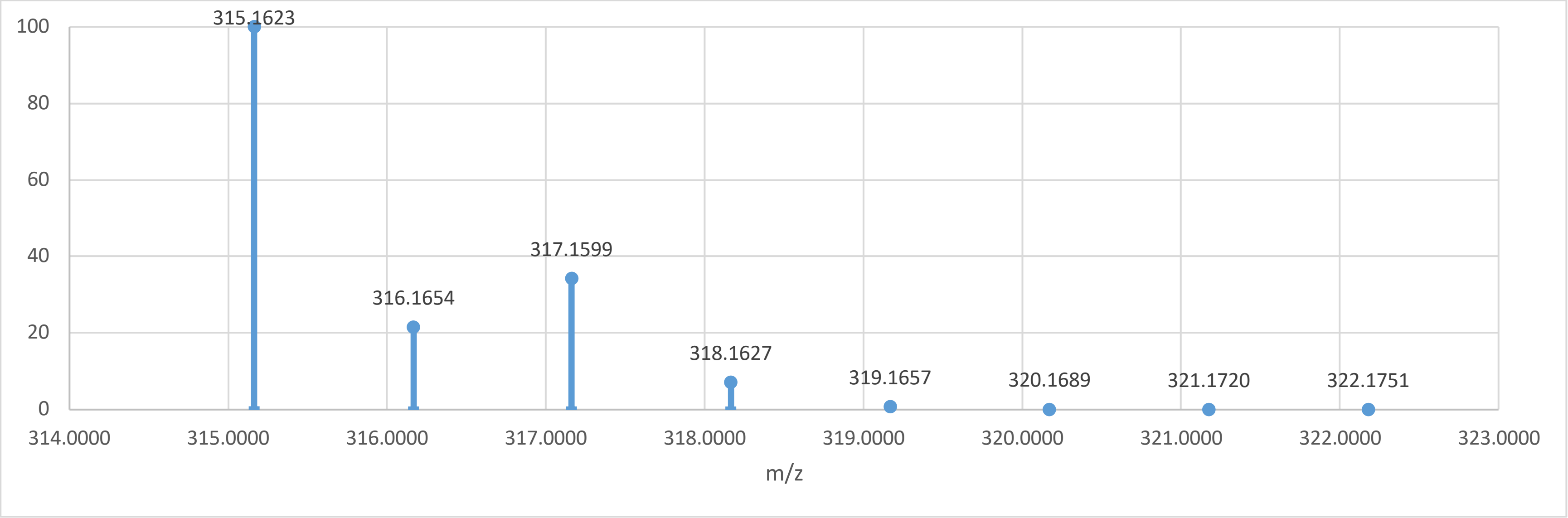

Measured

MS Zoomed Spectrum

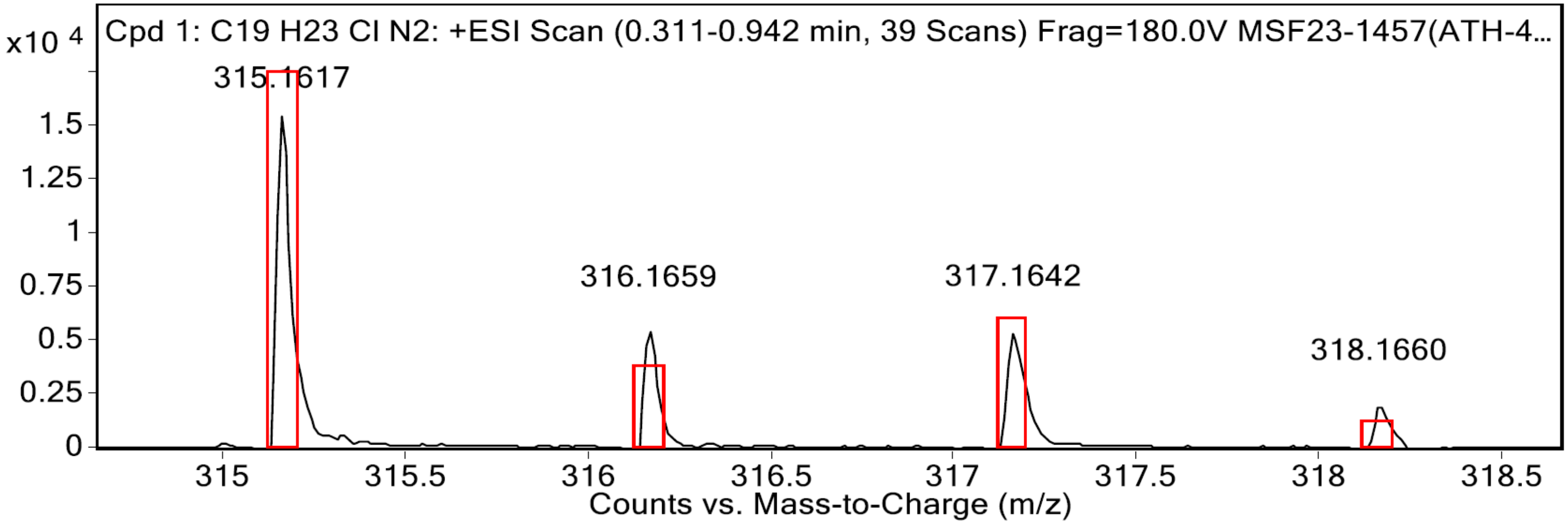

MS Spectrum Peak List

Obs. m/z	Calc. m/z	Charge	Abundance	Formula	Ion Species	Tgt Mass Error (ppm)
171.1159			1251253			
315.1617	315.1623	1	15687	C19H23ClN2	(M+H)+	1.63
316.1659	316.1654	1	5465	C19H23ClN2	(M+H)+	-1.36
317.1642	317.1599	1	5498	C19H23ClN2	(M+H)+	-13.56
318.1660	318.1627	1	2041	C19H23ClN2	(M+H)+	-10.52

--- End Of Report ---

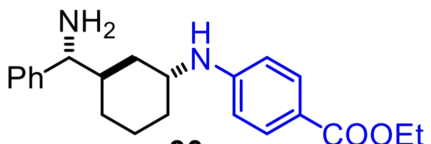

30

HR-MS (ESI-TOF) *m/z*: [M+H]$^+$ calculated for $C_{22}H_{29}N_2O_2^+$, 353.2224; found mass, 353.2230

Simulated

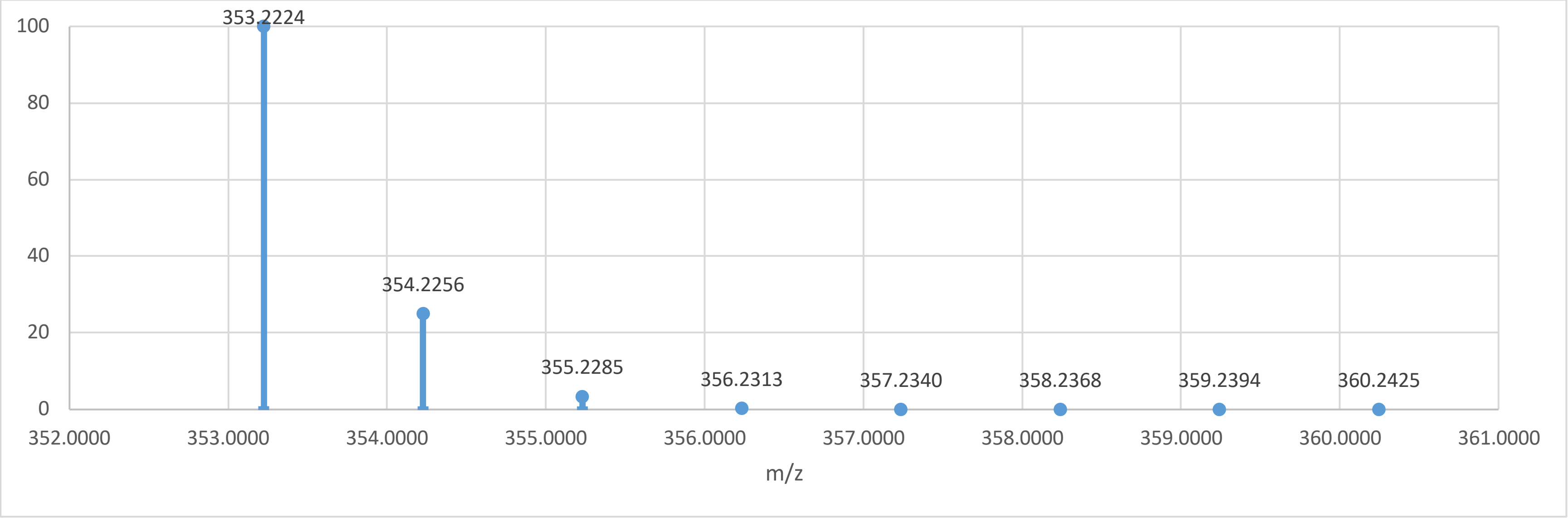

Measured

MS Zoomed Spectrum

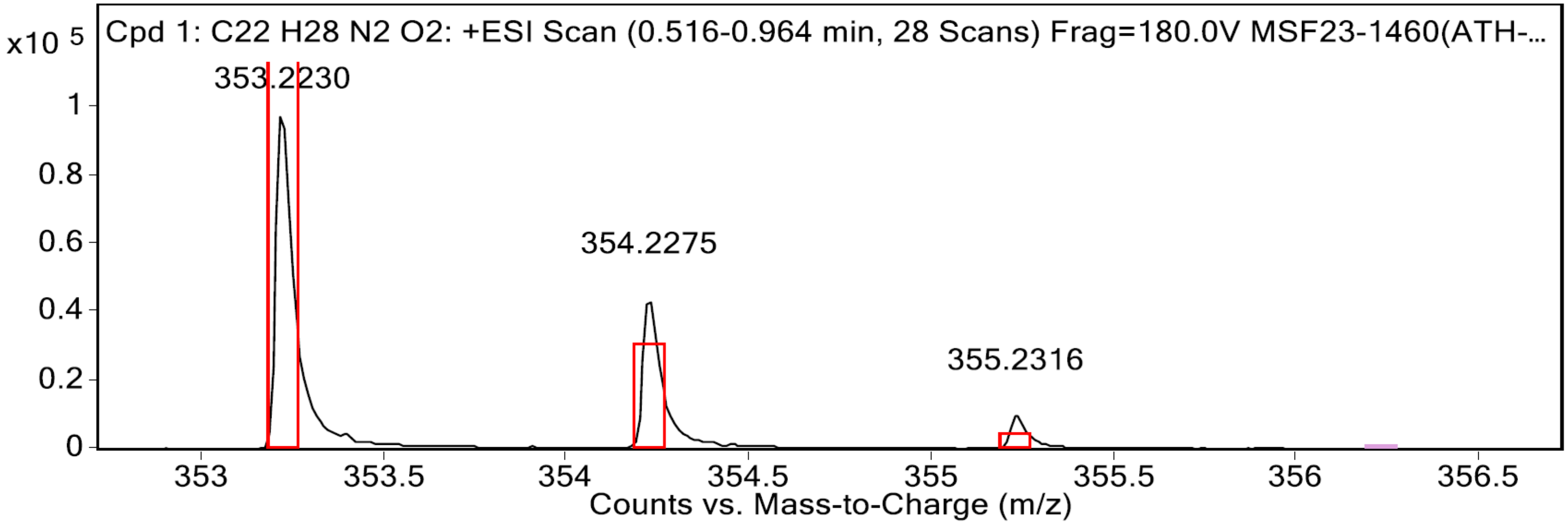

MS Spectrum Peak List

Obs. m/z	Calc. m/z	Charge	Abundance	Formula	Ion Species	Tgt Mass Error (ppm)
336.1963			1492790			
353.2230	353.2224	1	100199	C22H28N2O2	(M+H)+	-1.83
354.2275	354.2256	1	44184	C22H28N2O2	(M+H)+	-5.5
355.2316	355.2285	1	10049	C22H28N2O2	(M+H)+	-8.72

--- End Of Report ---

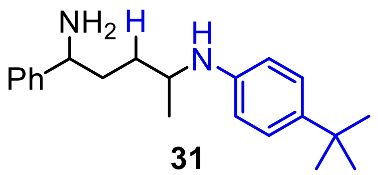

31

HR-MS (ESI-TOF) *m/z*: $[M+H]^+$ calculated for $C_{21}H_{31}N_2^+$, 311.2487; found mass, 311.2475

Simulated

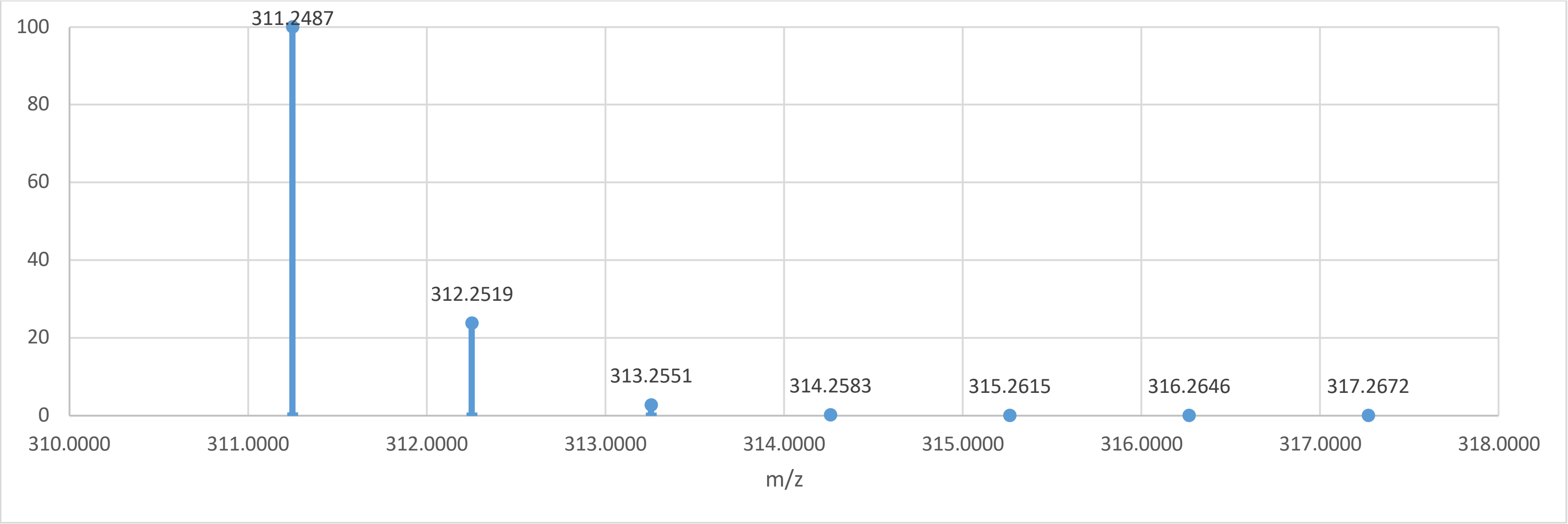

Measured

MS Zoomed Spectrum

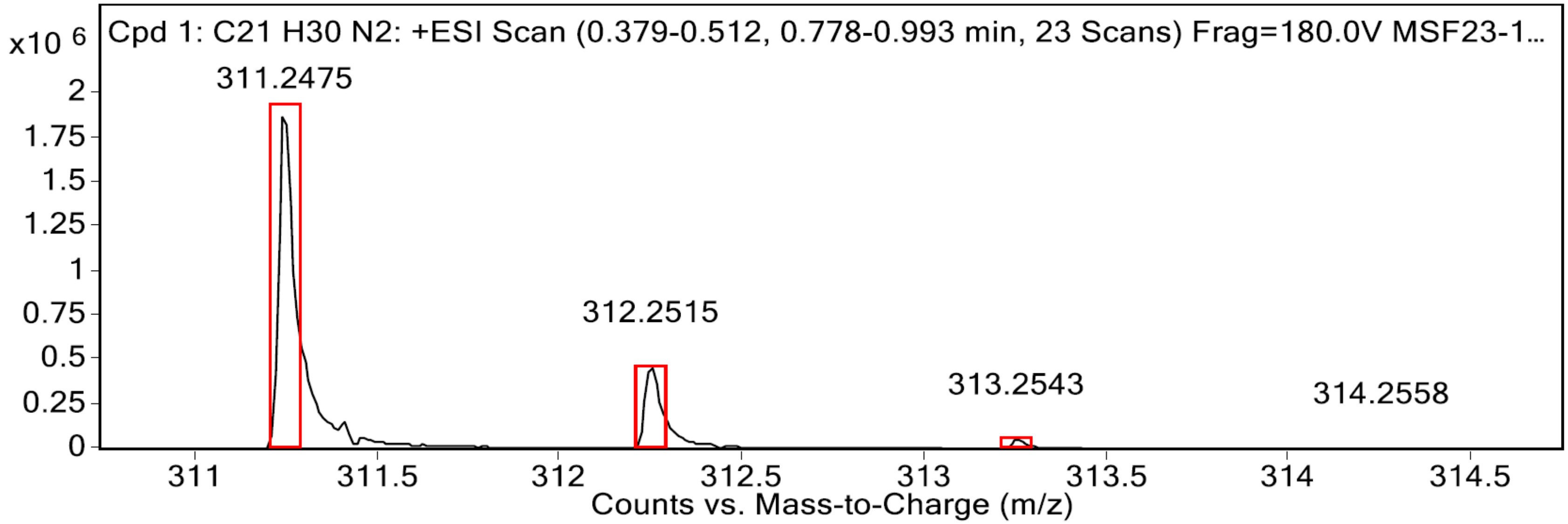

MS Spectrum Peak List

Obs. m/z	Calc. m/z	Charge	Abundance	Formula	Ion Species	Tgt Mass Error (ppm)
294.2209			2958008			
311.2475	311.2482	1	1934663	C21H30N2	(M+H)+	2.13
312.2515	312.2514	1	462614	C21H30N2	(M+H)+	-0.24
313.2543	313.2546	1	54890	C21H30N2	(M+H)+	1.01
314.2558	314.2578	1	3433	C21H30N2	(M+H)+	6.25

--- End Of Report ---

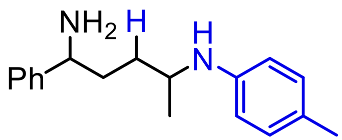

32

HR-MS (ESI-TOF) *m/z*: $[M+H]^+$ calculated for $C_{18}H_{25}N_2^+$, 269.2018; found mass, 269.2008

Simulated

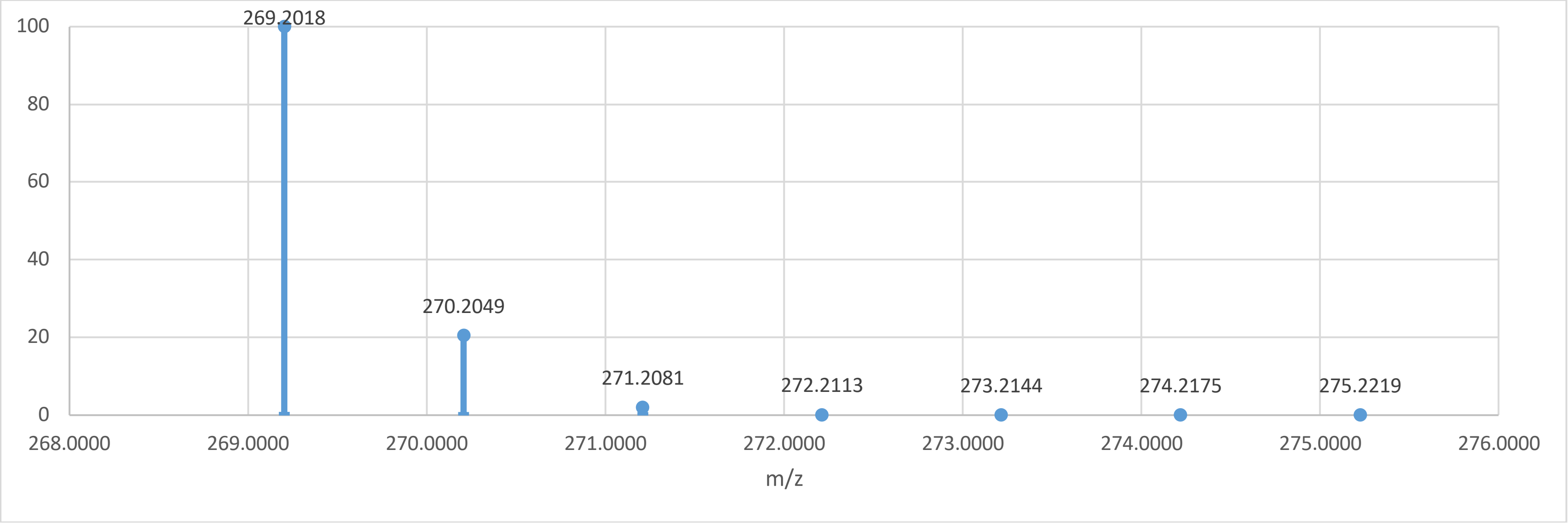

Measured

MS Zoomed Spectrum

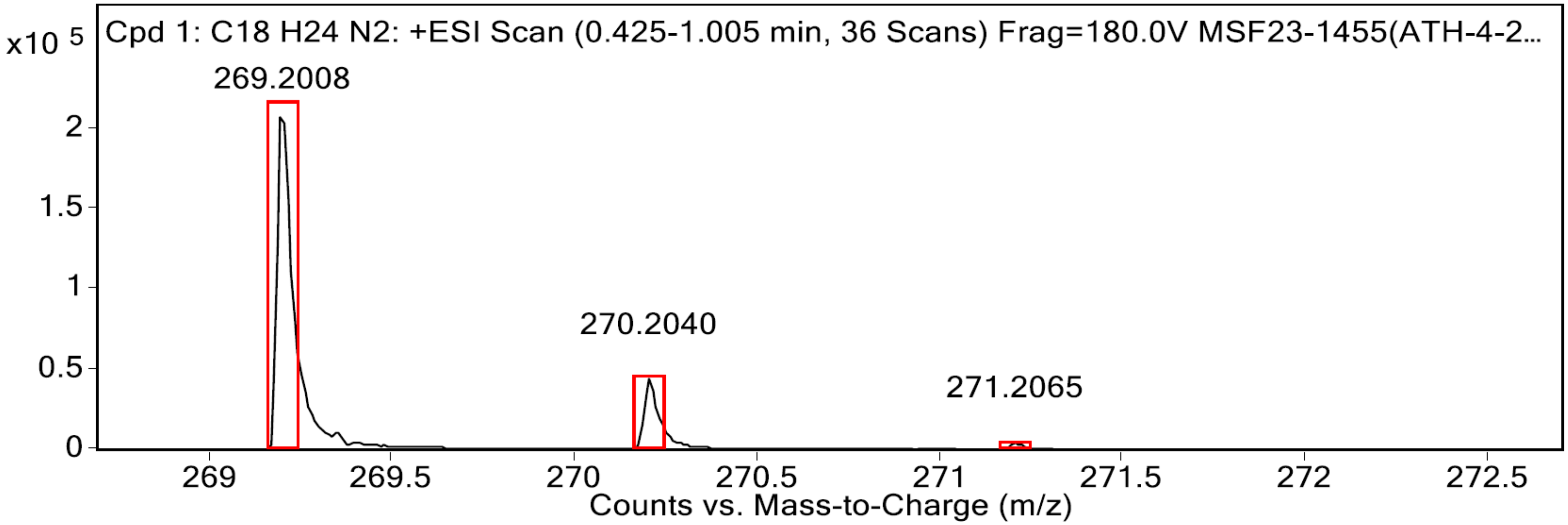

MS Spectrum Peak List

Obs. m/z	Calc. m/z	Charge	Abundance	Formula	Ion Species	Tgt Mass Error (ppm)
252.1738			2727513			
269.2008	269.2012	1	215531	C18H24N2	(M+H)+	1.49
270.2040	270.2044	1	44052	C18H24N2	(M+H)+	1.59
271.2065	271.2076	1	4694	C18H24N2	(M+H)+	3.94

--- End Of Report ---

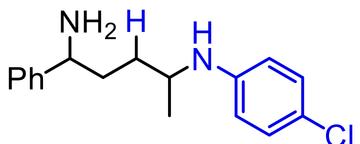

33

HR-MS (ESI-TOF) *m/z*: $[M+H]^+$ calculated for $C_{17}H_{22}ClN_2^+$, 289.1472; found mass, 289.1462

Simulated

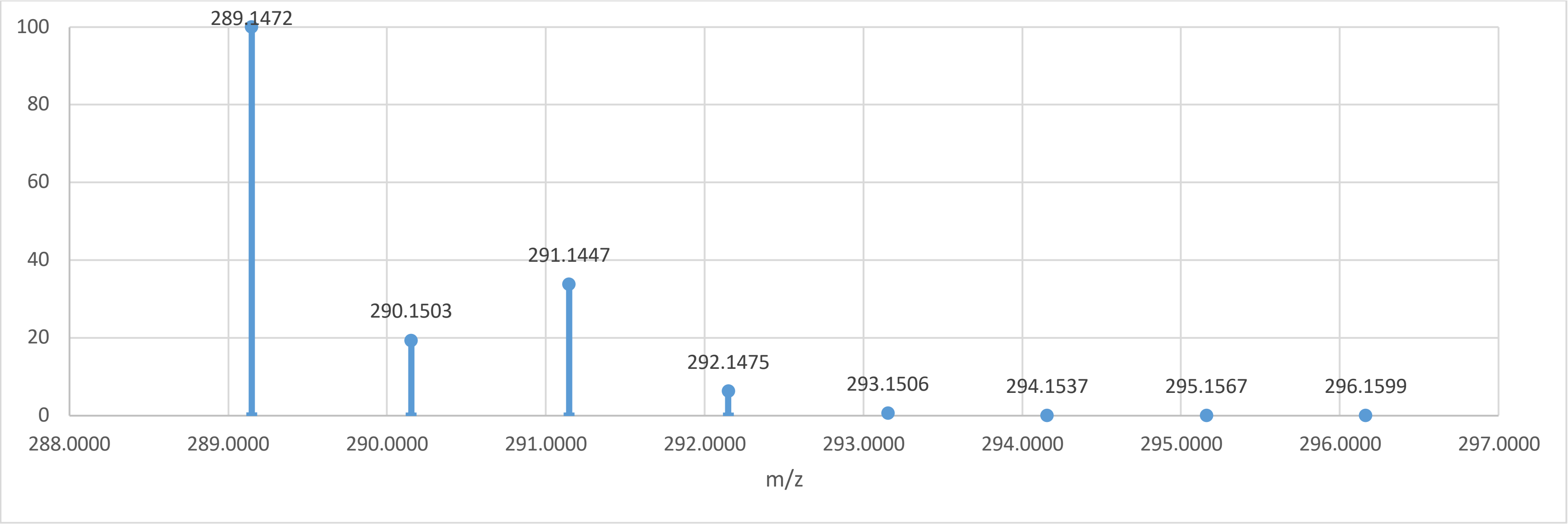

Measured

MS Zoomed Spectrum

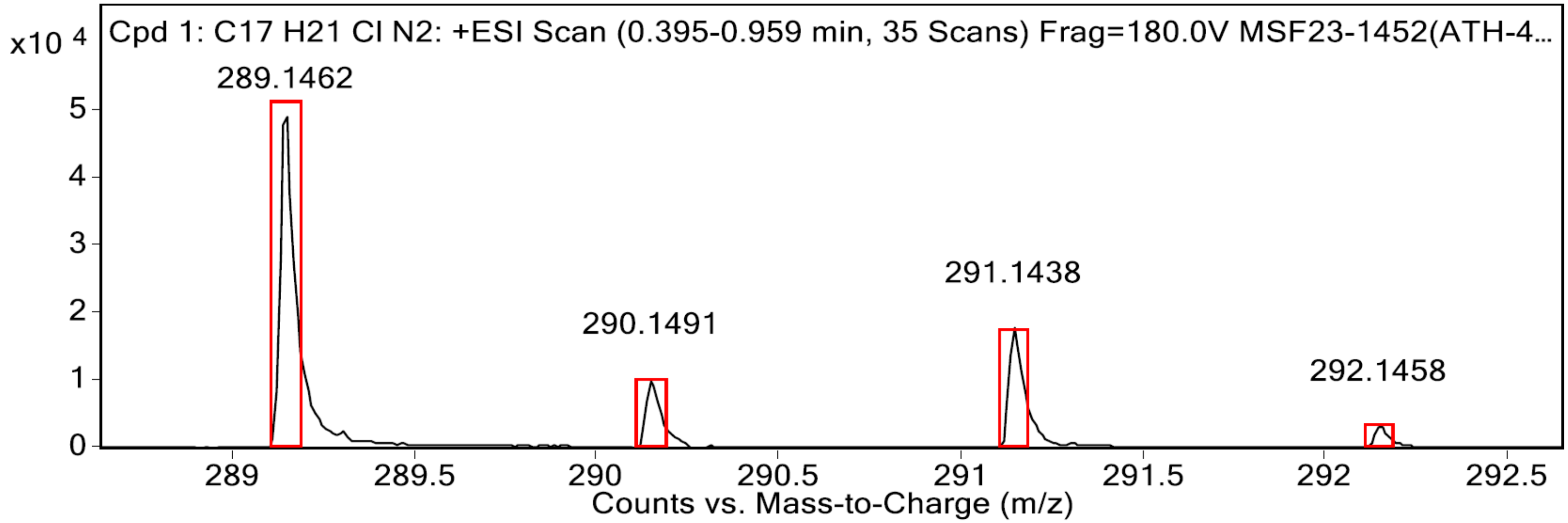

MS Spectrum Peak List

Obs. m/z	Calc. m/z	Charge	Abundance	Formula	Ion Species	Tgt Mass Error (ppm)
272.1194			1794913			
289.1462	289.1466	1	50341	C17H21ClN2	(M+H)+	1.4
290.1491	290.1498	1	10365	C17H21ClN2	(M+H)+	2.27
291.1438	291.1441	1	17968	C17H21ClN2	(M+H)+	1.08
292.1458	292.1470	1	3362	C17H21ClN2	(M+H)+	3.94

--- End Of Report ---

J. NMR Spectra

1H, 500.35 Hz, CDCl3

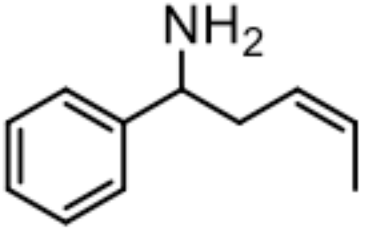

1a

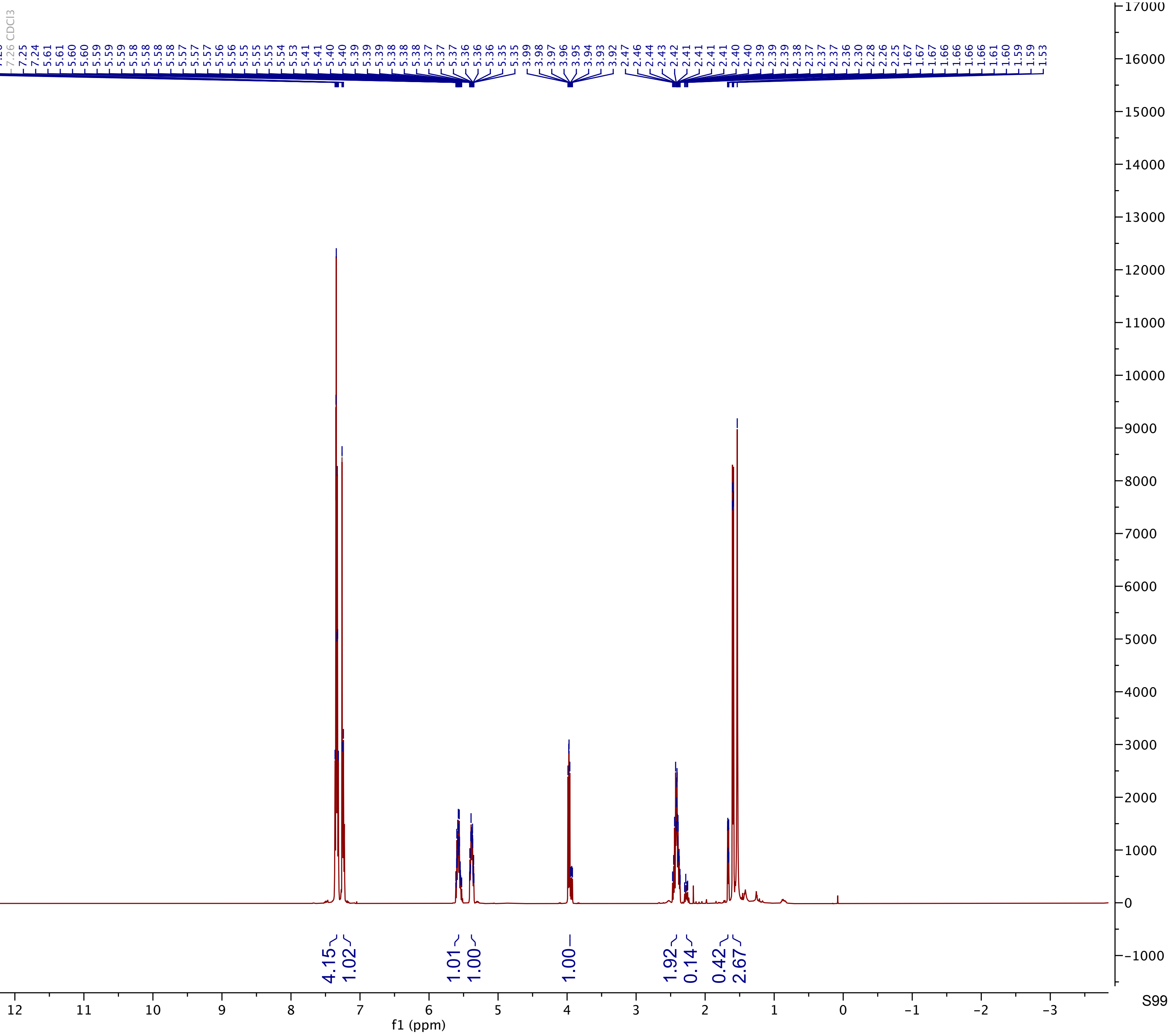

13C, 125.83 Hz, CDCl3

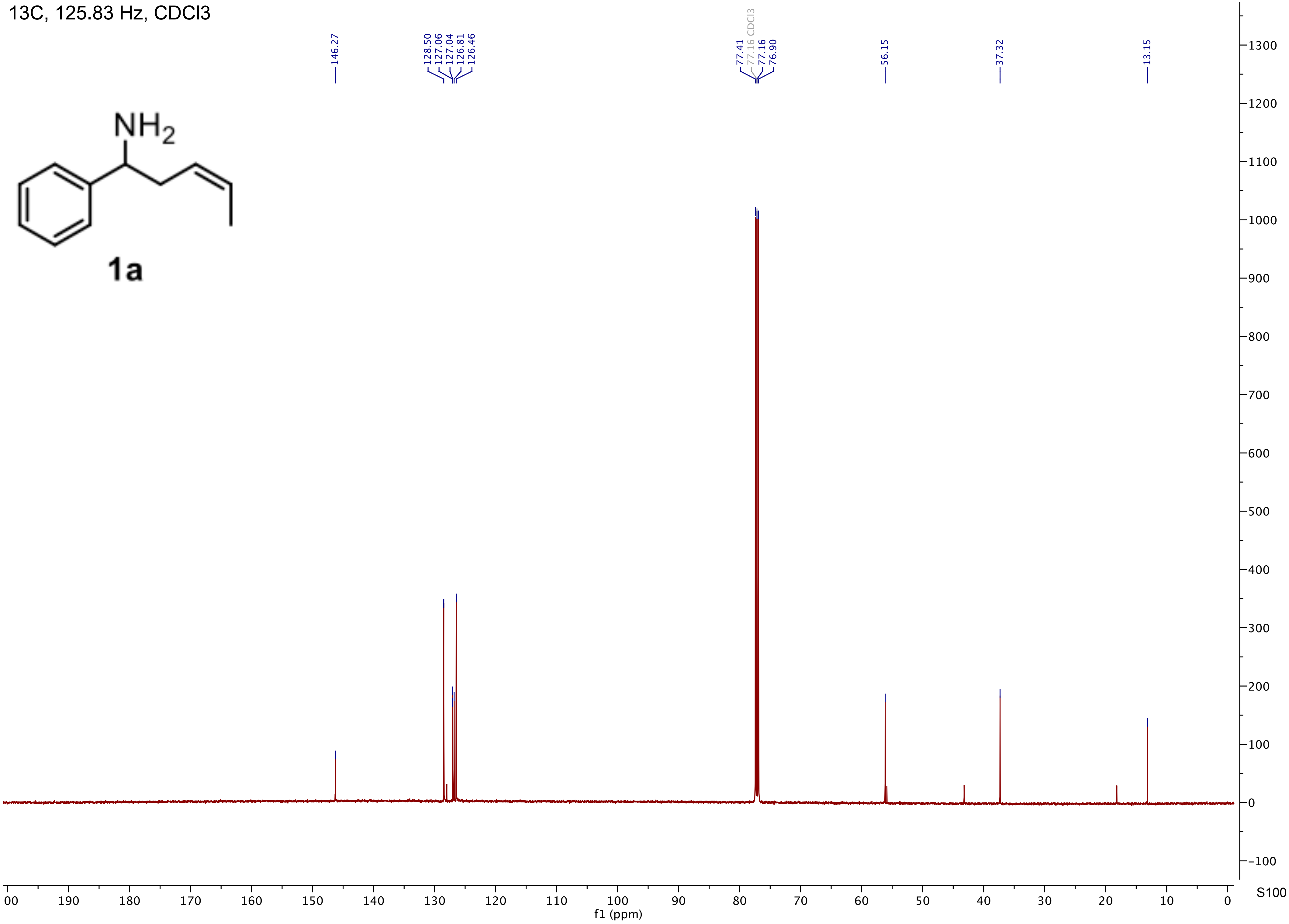

1a

1H, 500.35 Hz, CDCl3

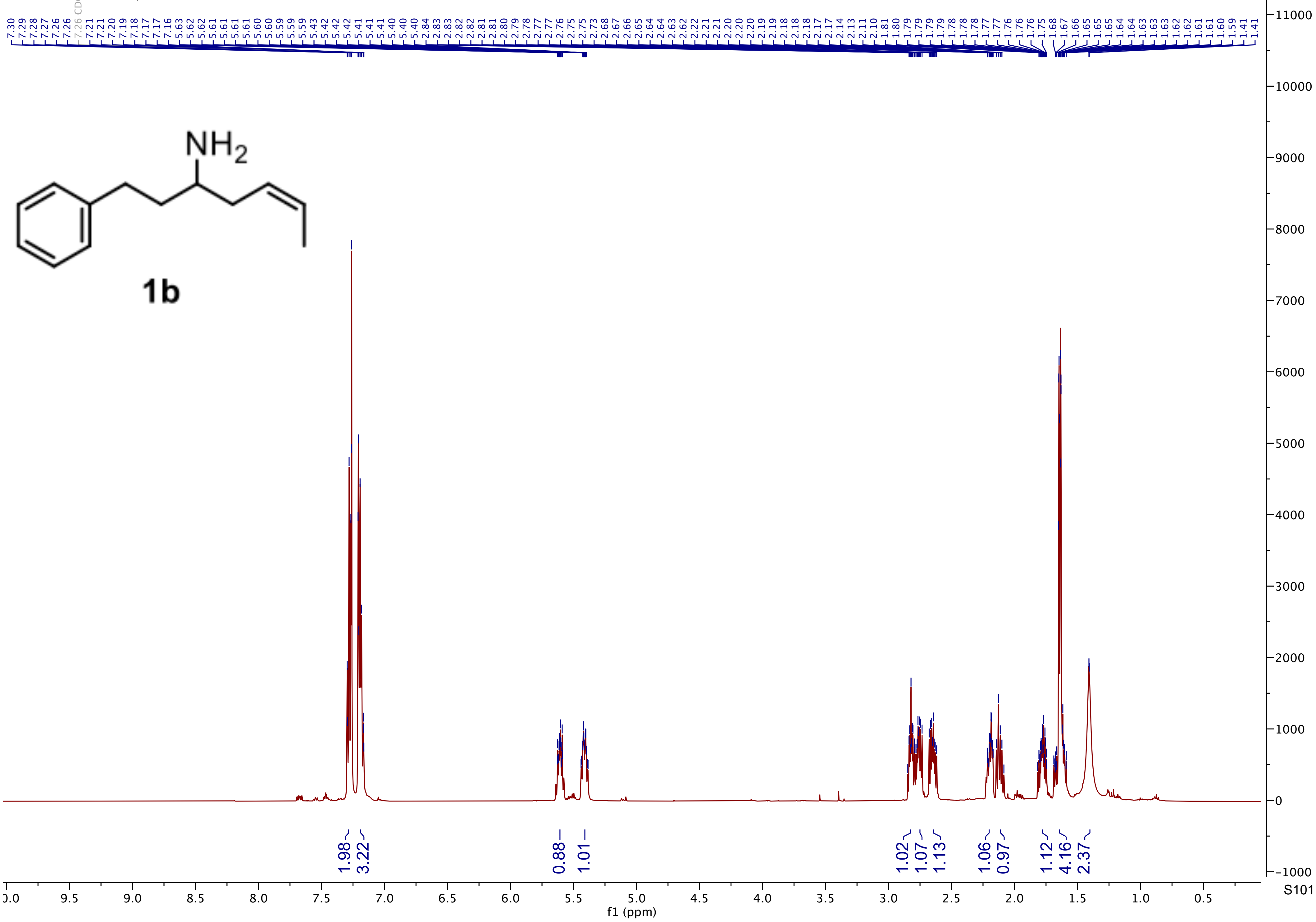

13C, 125.83 Hz, CDCl3

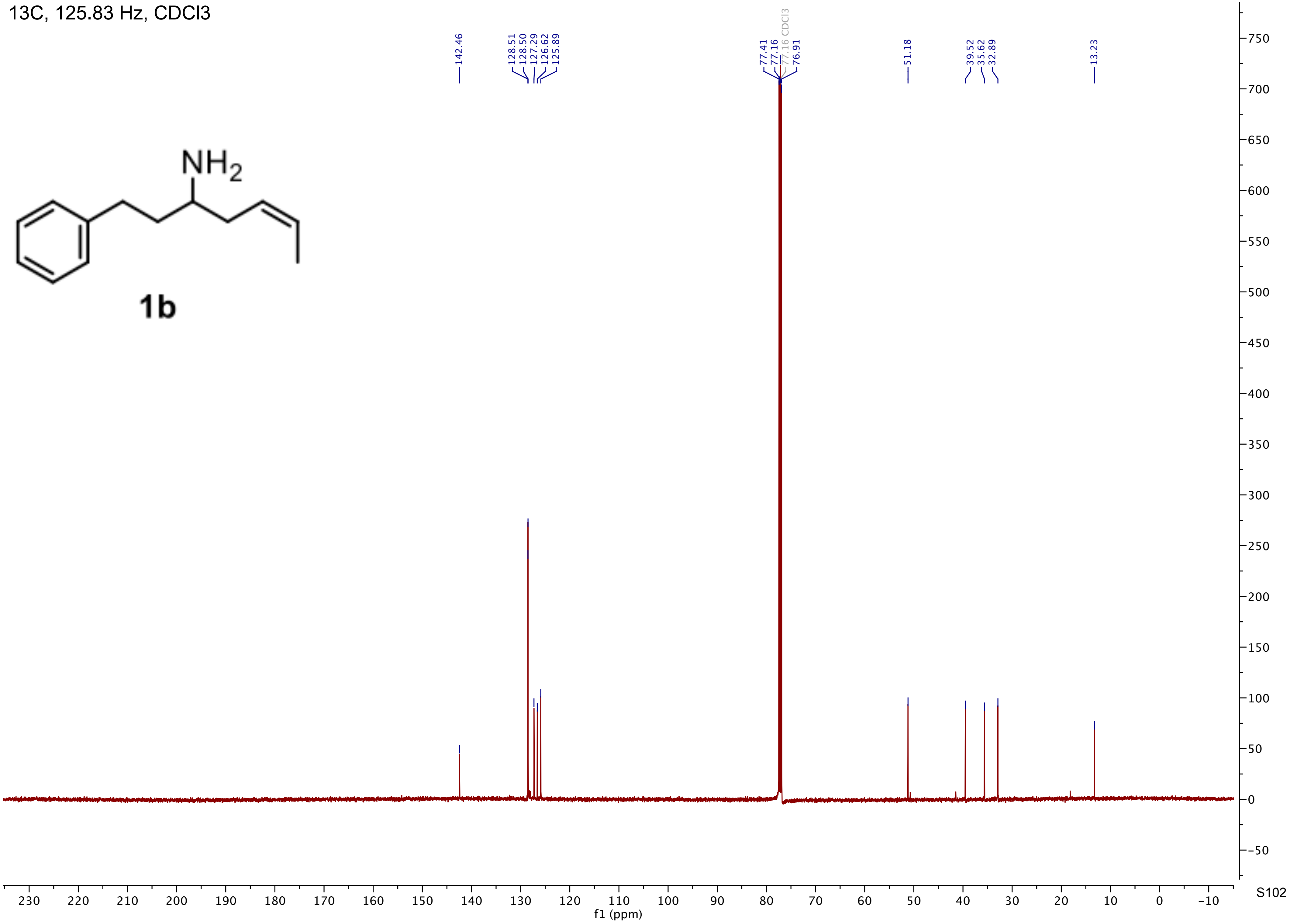

1H, 399.81 Hz, CDCl3

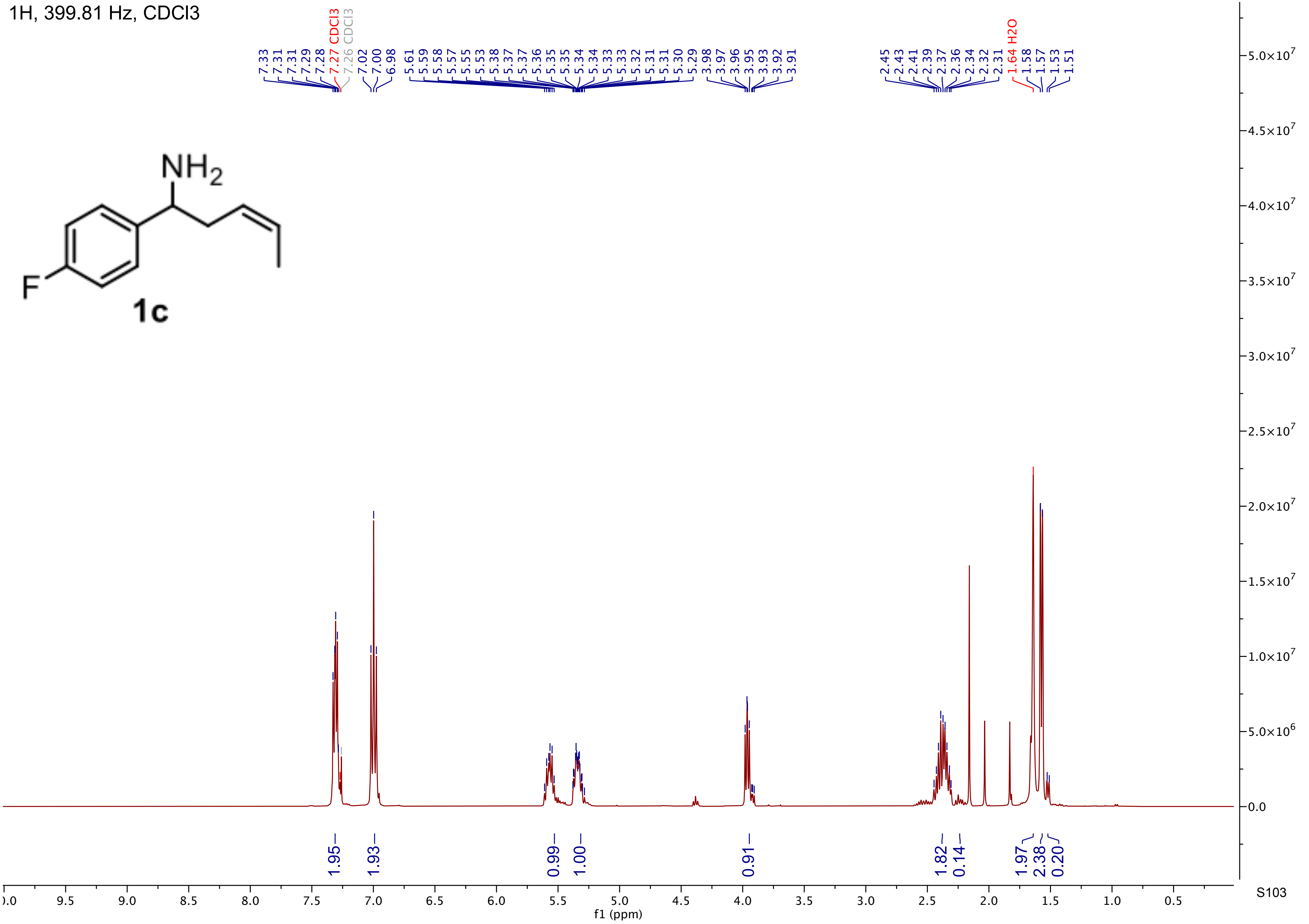

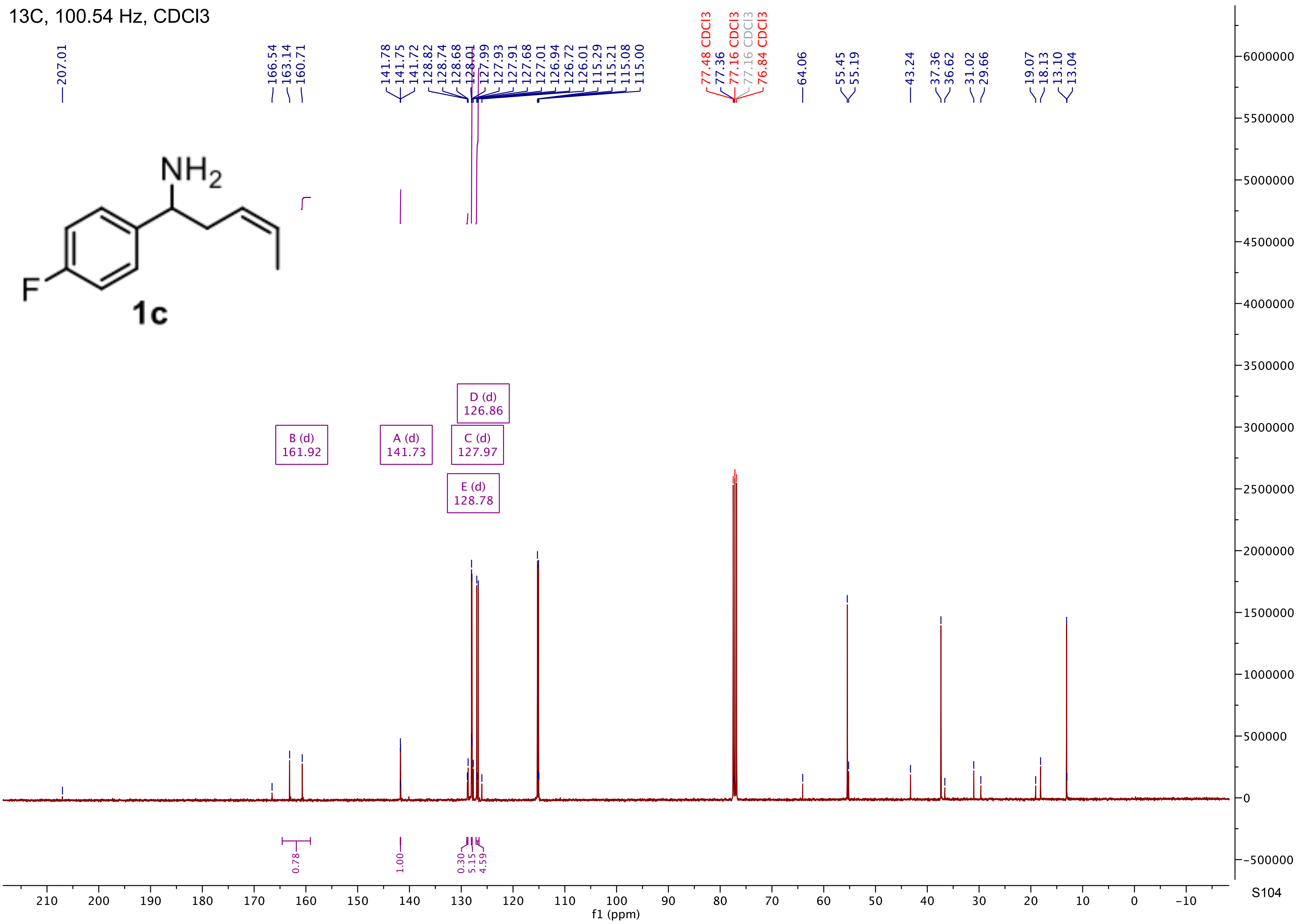
13C, 100.54 Hz, CDCl3
NH2
F
1c
207.01
166.54
163.14
160.71
141.78
141.75
141.72
128.82
128.74
128.68
128.01
127.99
127.93
127.91
127.68
127.01
126.94
126.72
126.01
115.29
115.21
115.08
115.00
77.48 CDCl3
77.36
77.16 CDCl3
77.16 CDCl3
76.84 CDCl3
64.06
55.45
55.19
43.24
37.36
36.62
31.02
29.66
19.07
18.13
13.10
13.04
D (d) 126.86
B (d) 161.92
A (d) 141.73
C (d) 127.97
E (d) 128.78
0.78
1.00
0.30
5.15
4.59
f1 (ppm)

19F, 376.30 Hz, cdcl3

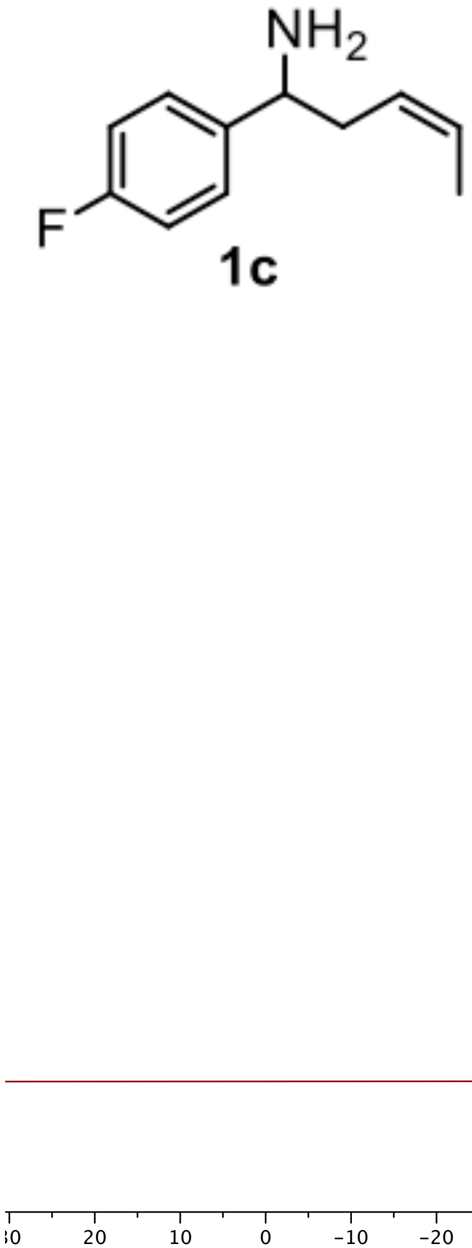

1c

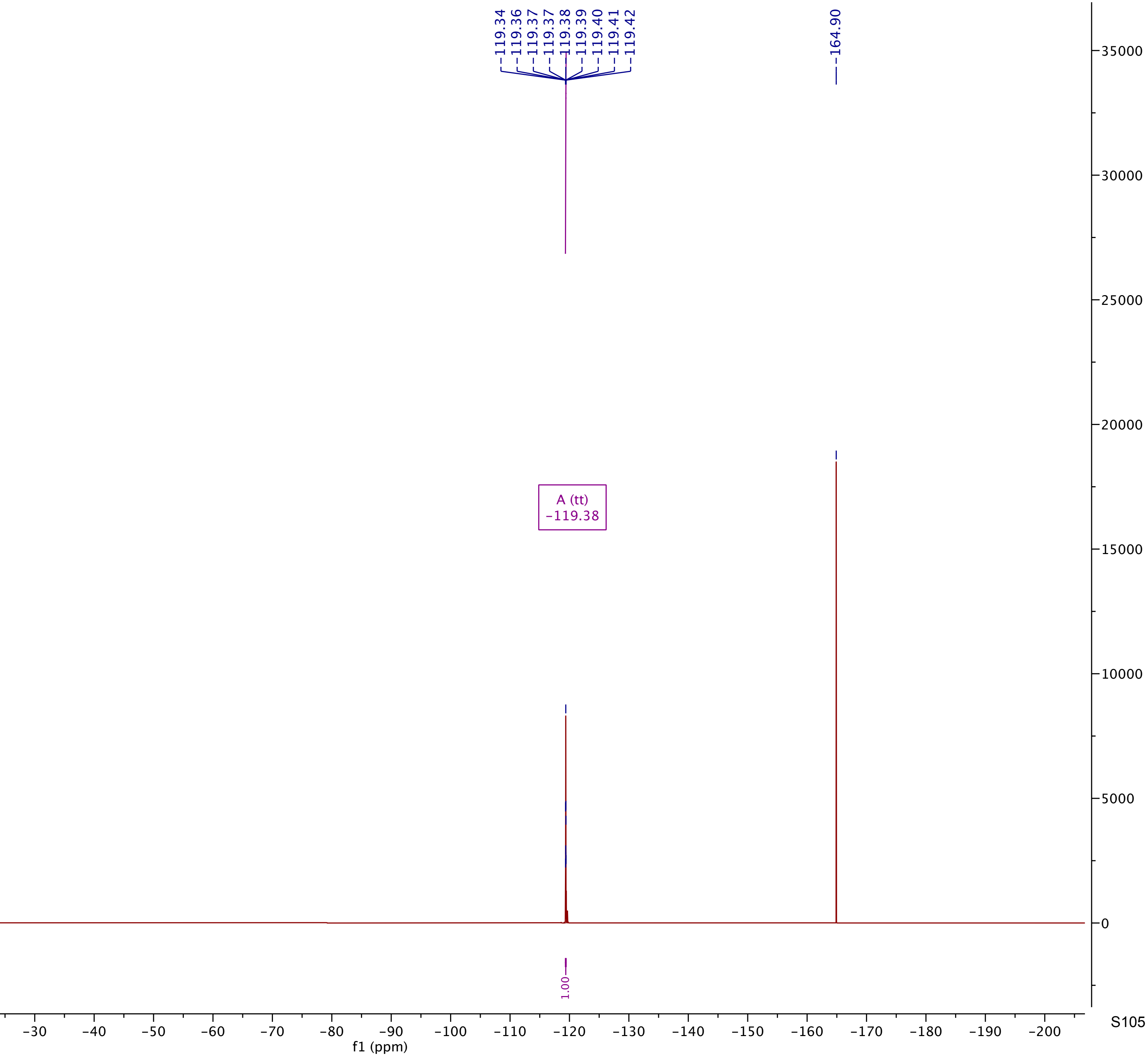

1H, 500.35 Hz, CDCl3

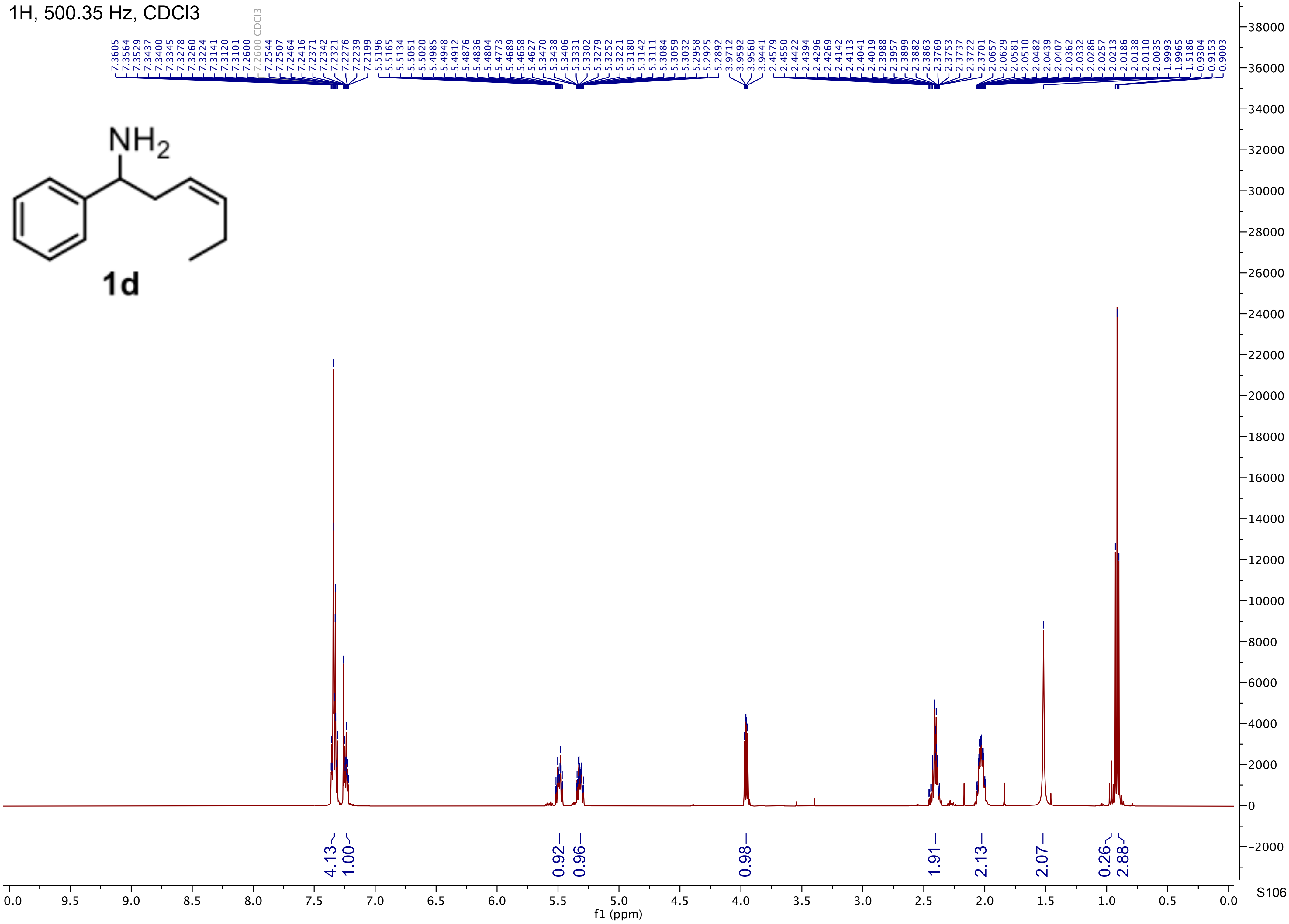

13C, 125.83 Hz, CDCl3

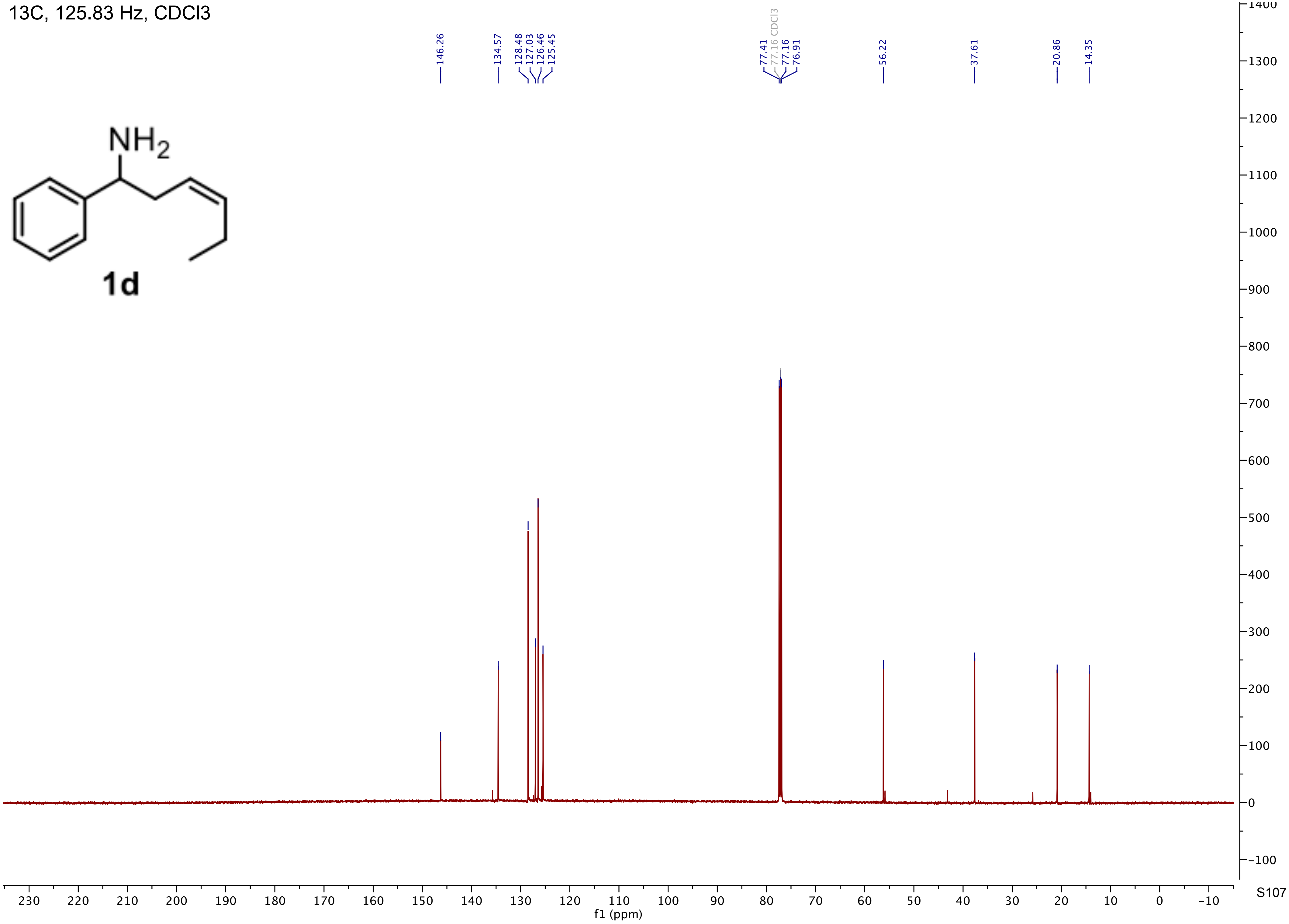

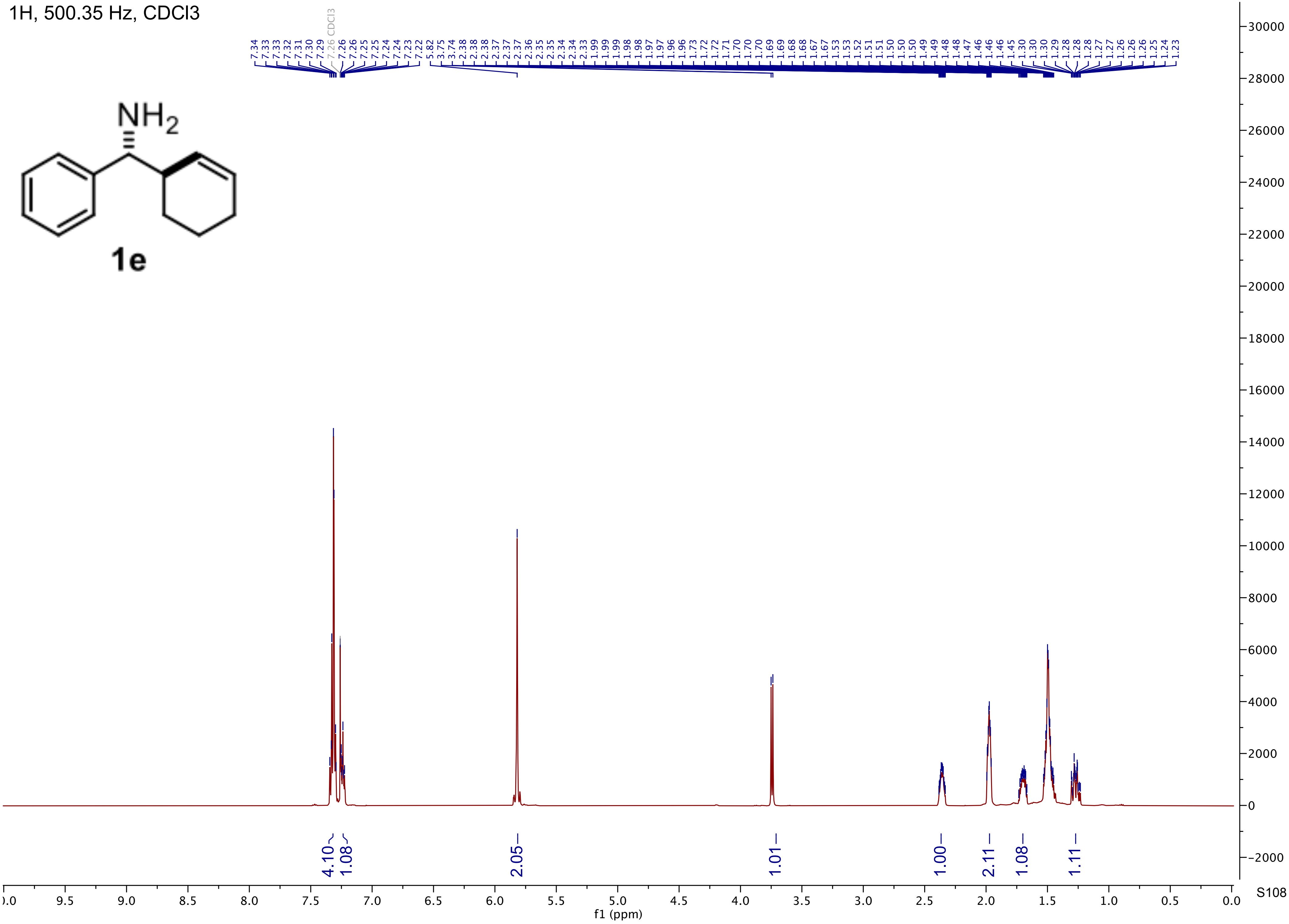
1H, 500.35 Hz, CDCl3
NH2
1e
4.10
1.08
2.05
1.01
1.00
2.11
1.08
1.11
f1 (ppm)

13C, 125.83 Hz, CDCl3

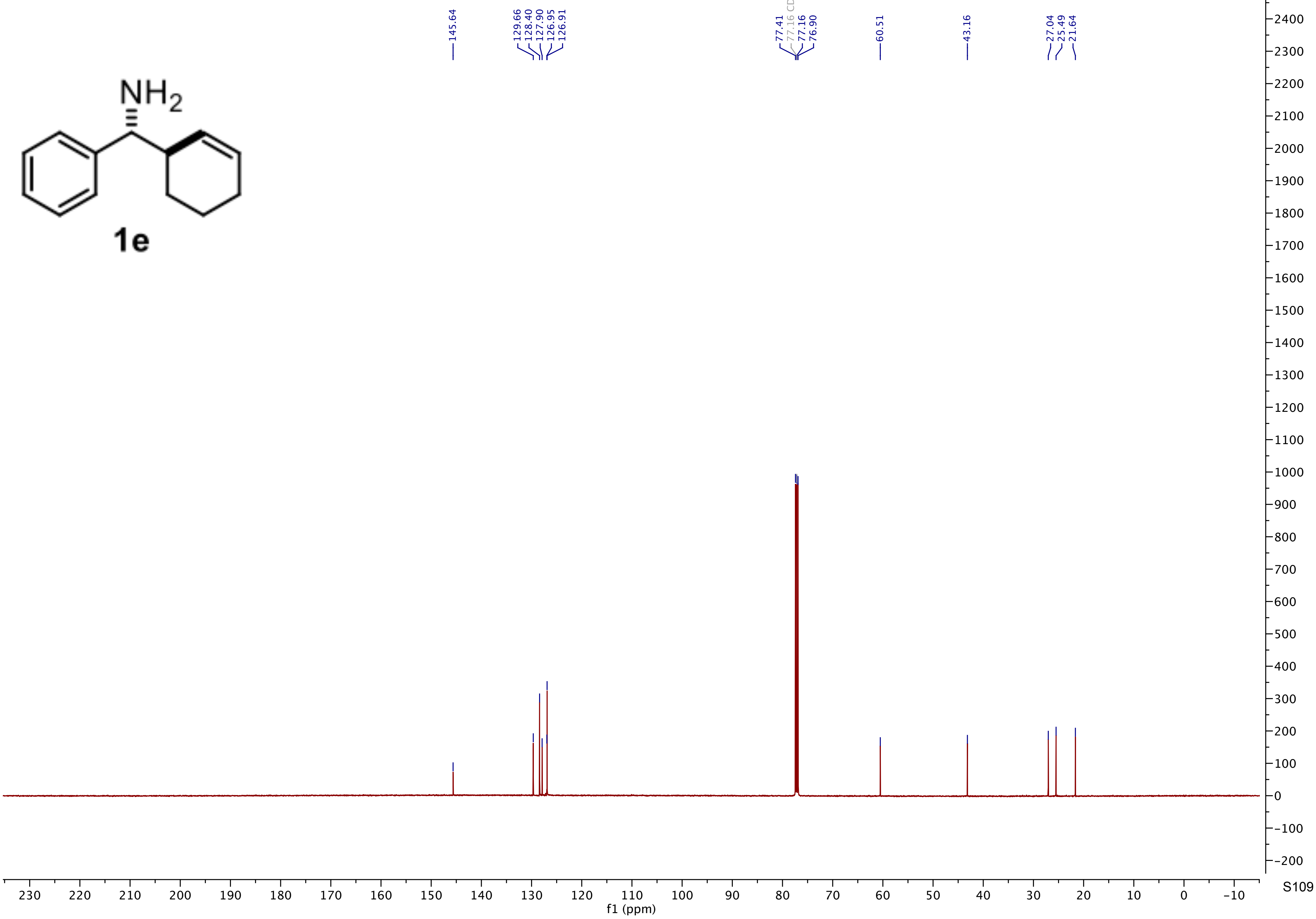

1H, 500.35 Hz, CDCl3

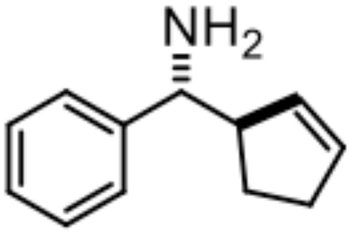

1f

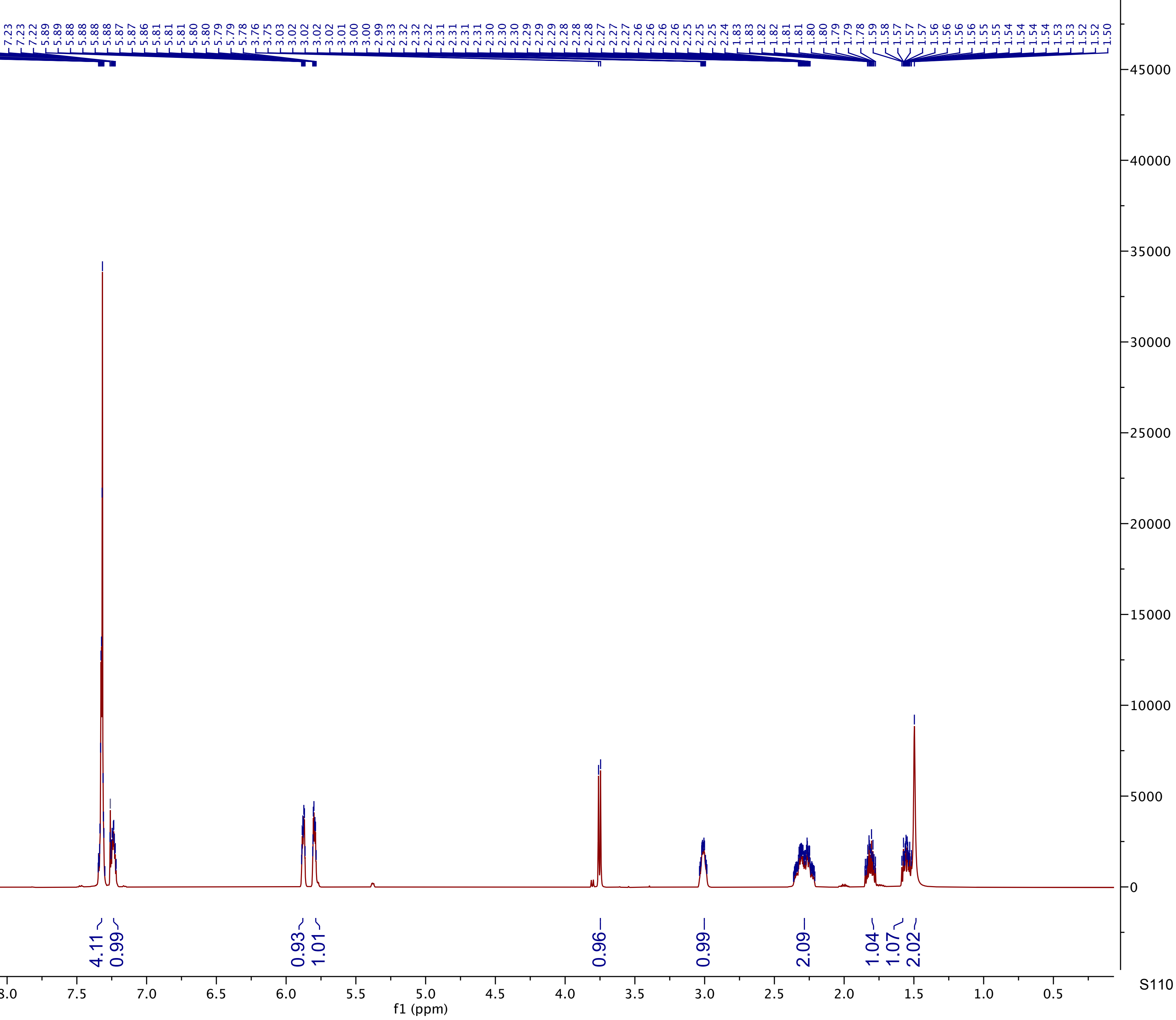

13C, 125.83 Hz, CDCl3

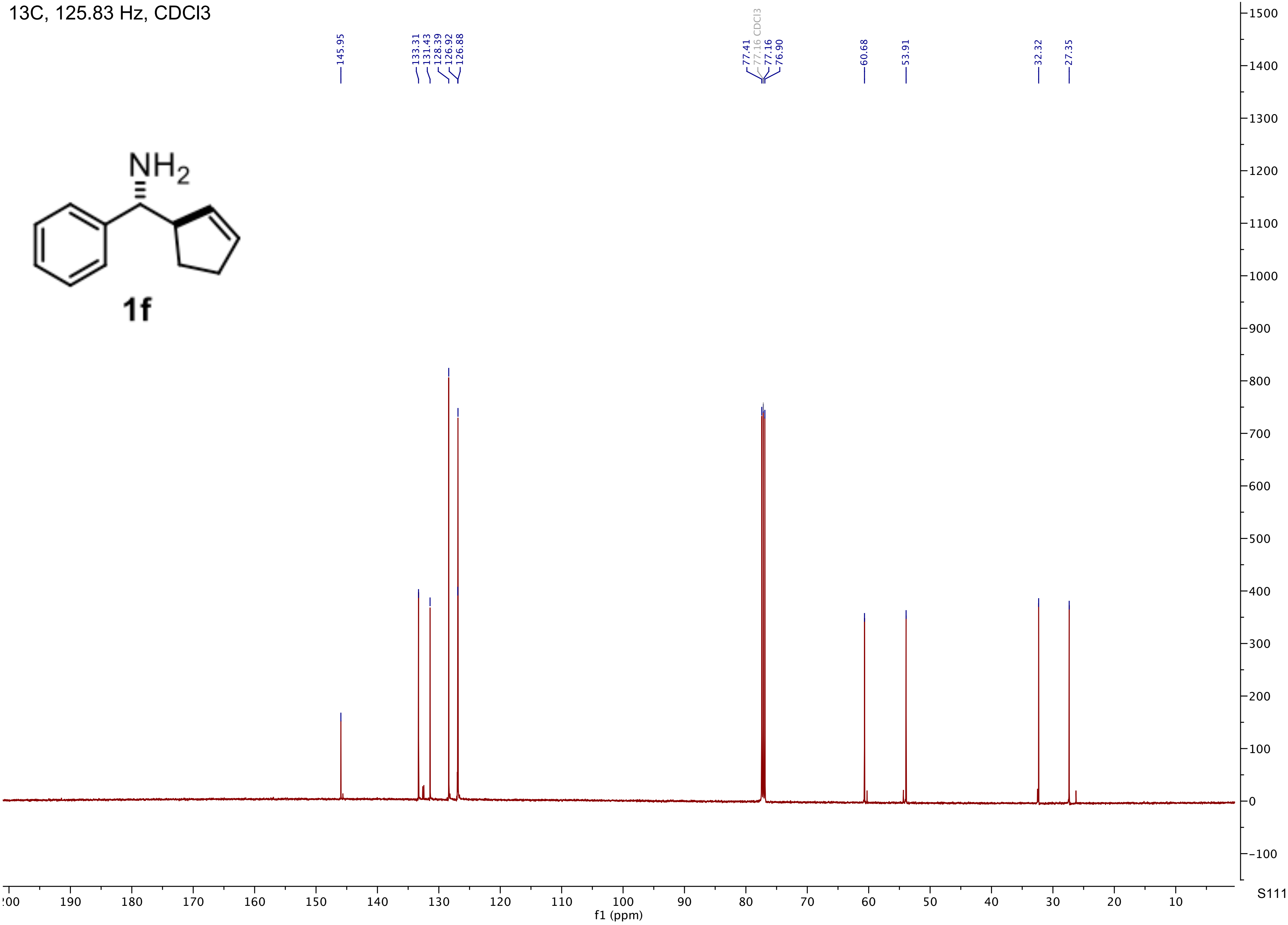

1H, 500.35 Hz, CDCl3

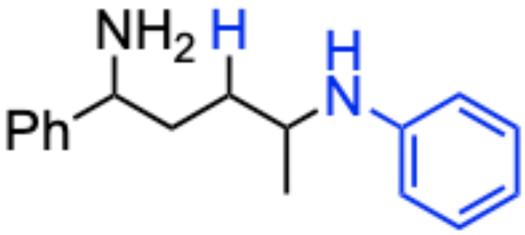

3

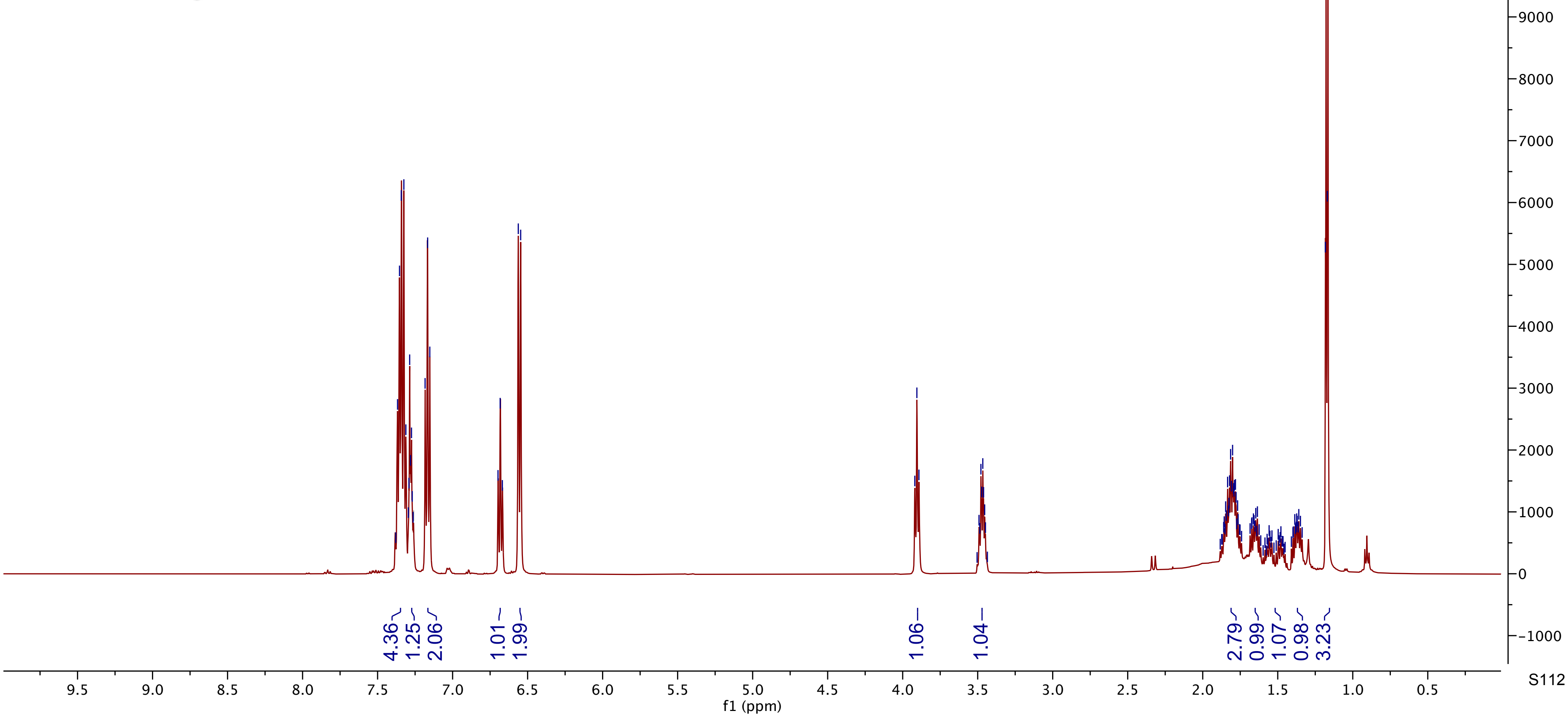

13C, 125.83 Hz, CDCl3

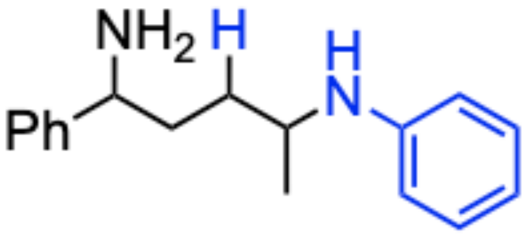

3

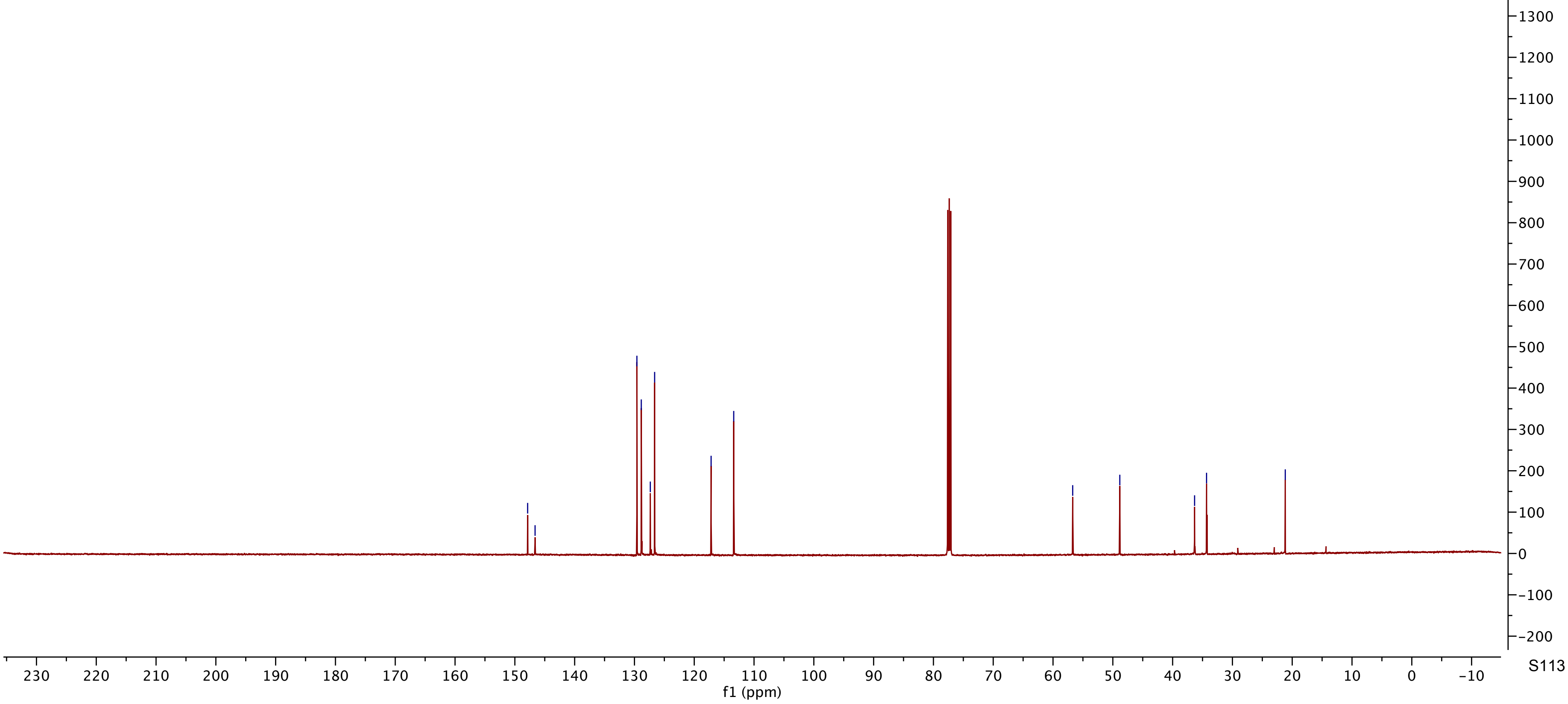

1H, 500.35 Hz, CDCl3

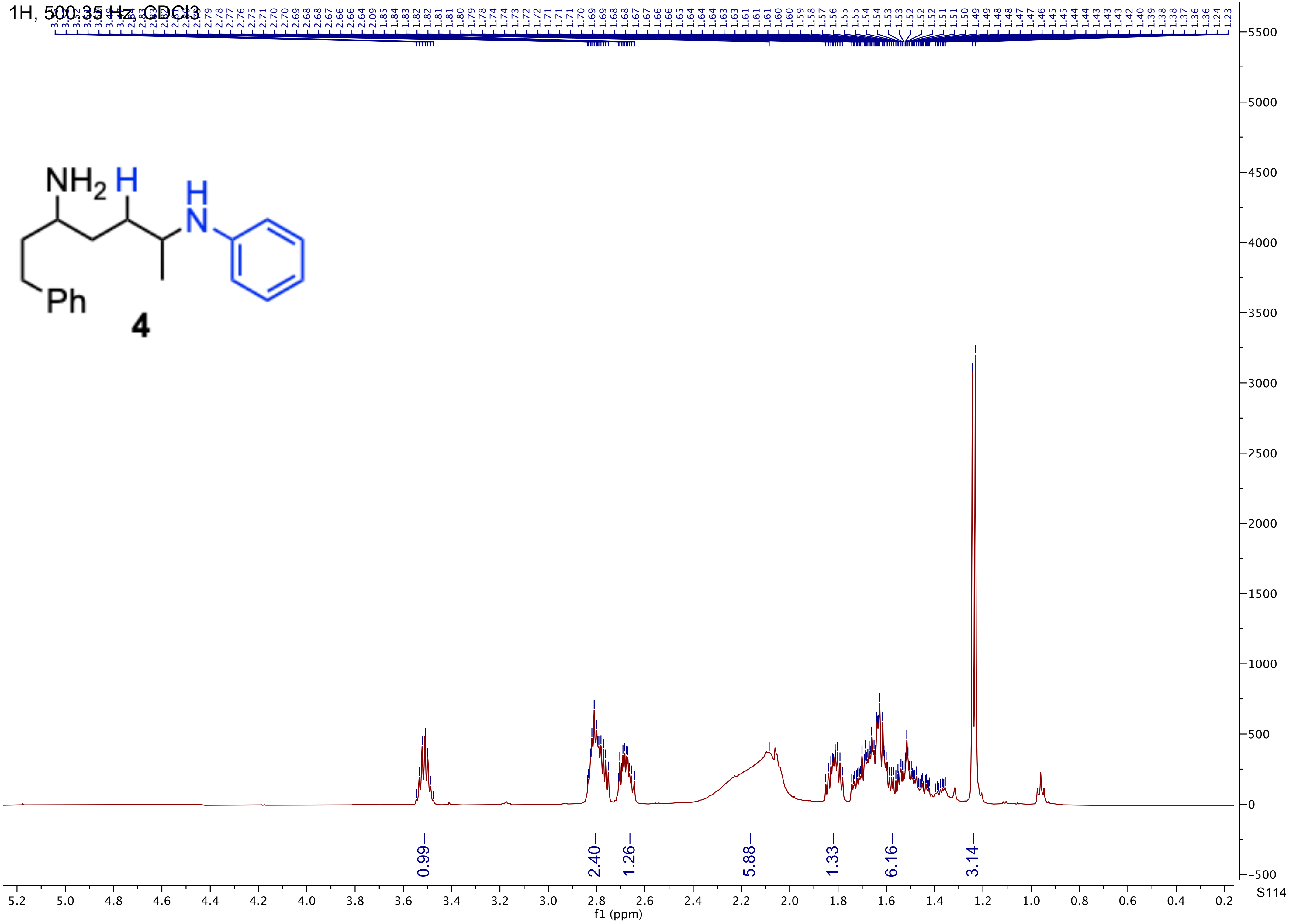

13C, 125.83 Hz, CDCl3

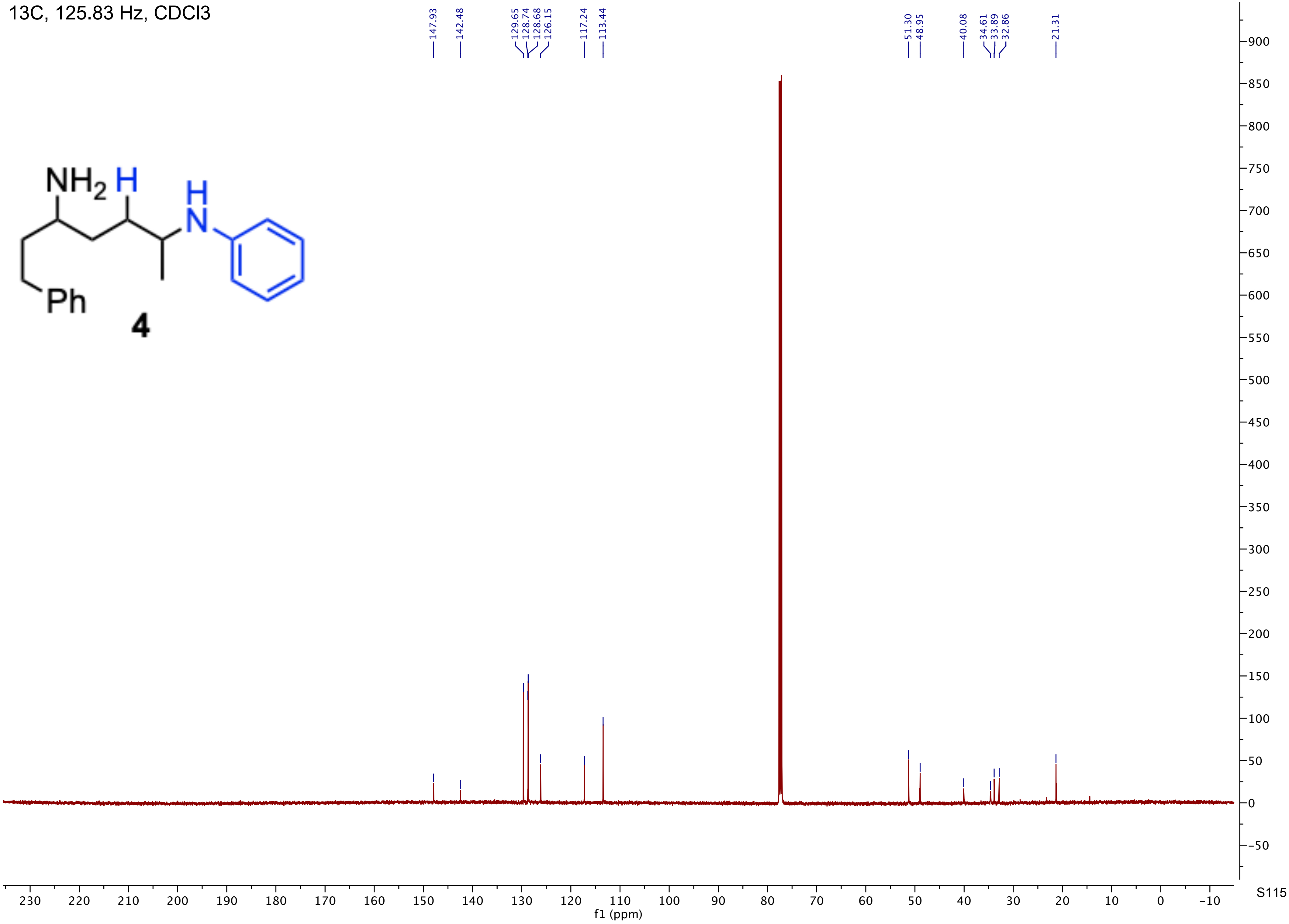

4

1H, 500.35 Hz, CDCl3

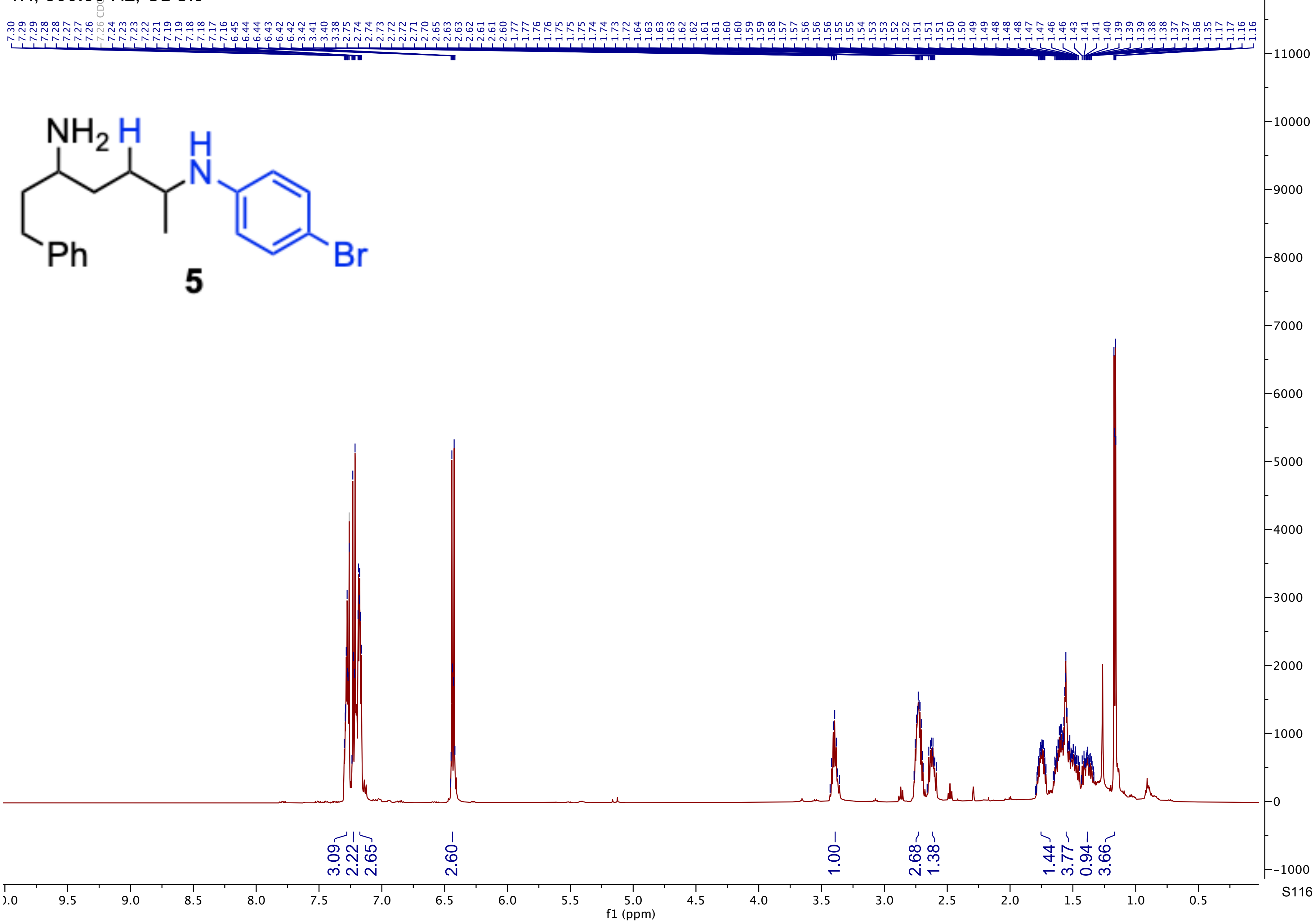

13C, 125.83 Hz, CDCl3

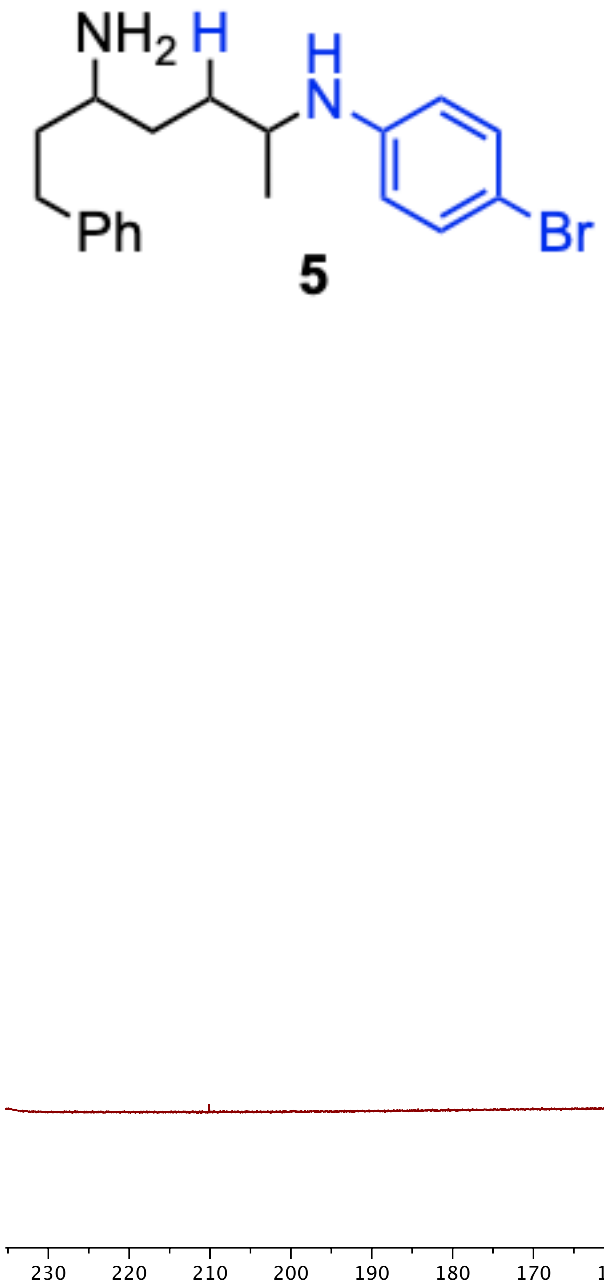

5

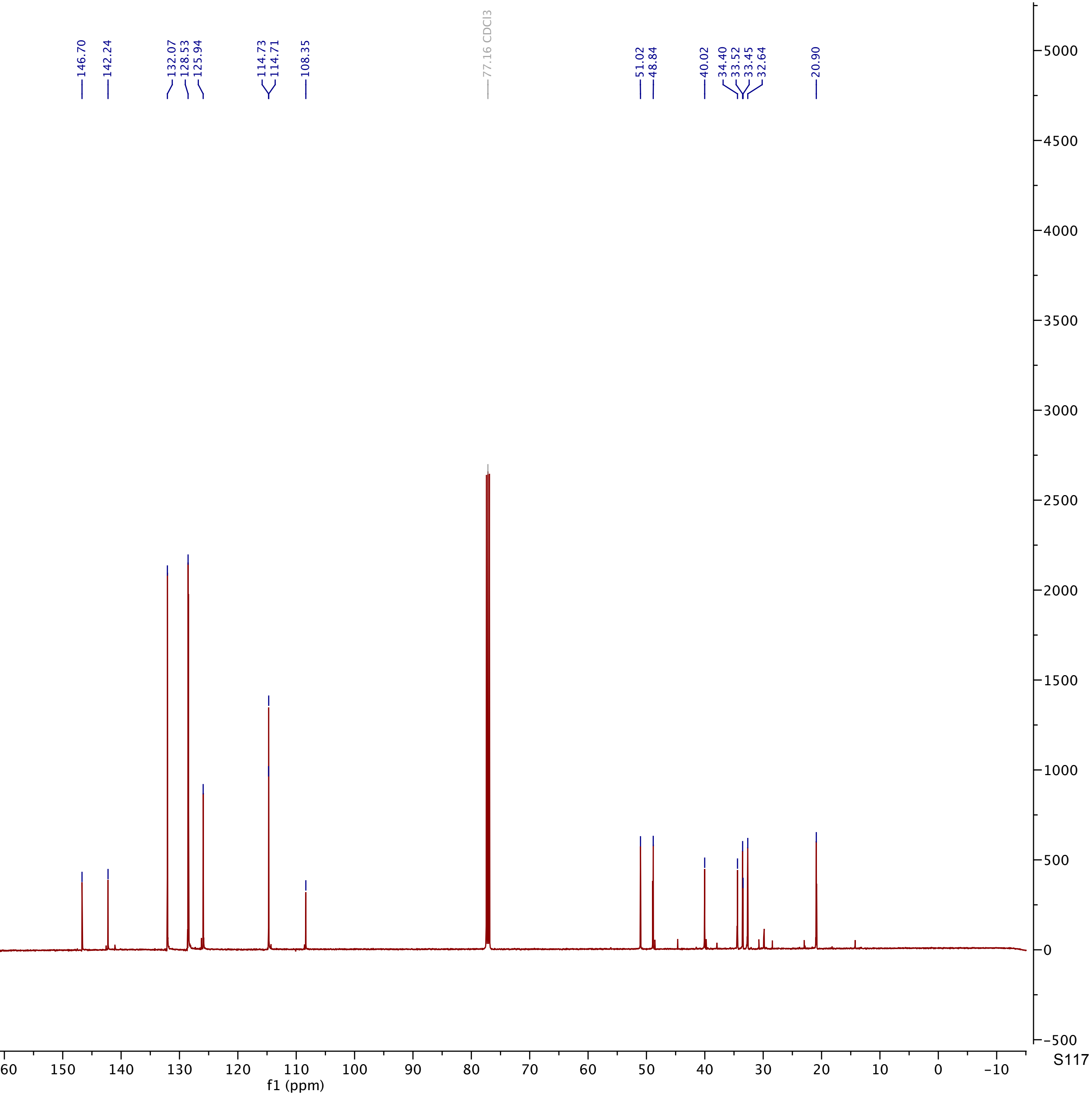

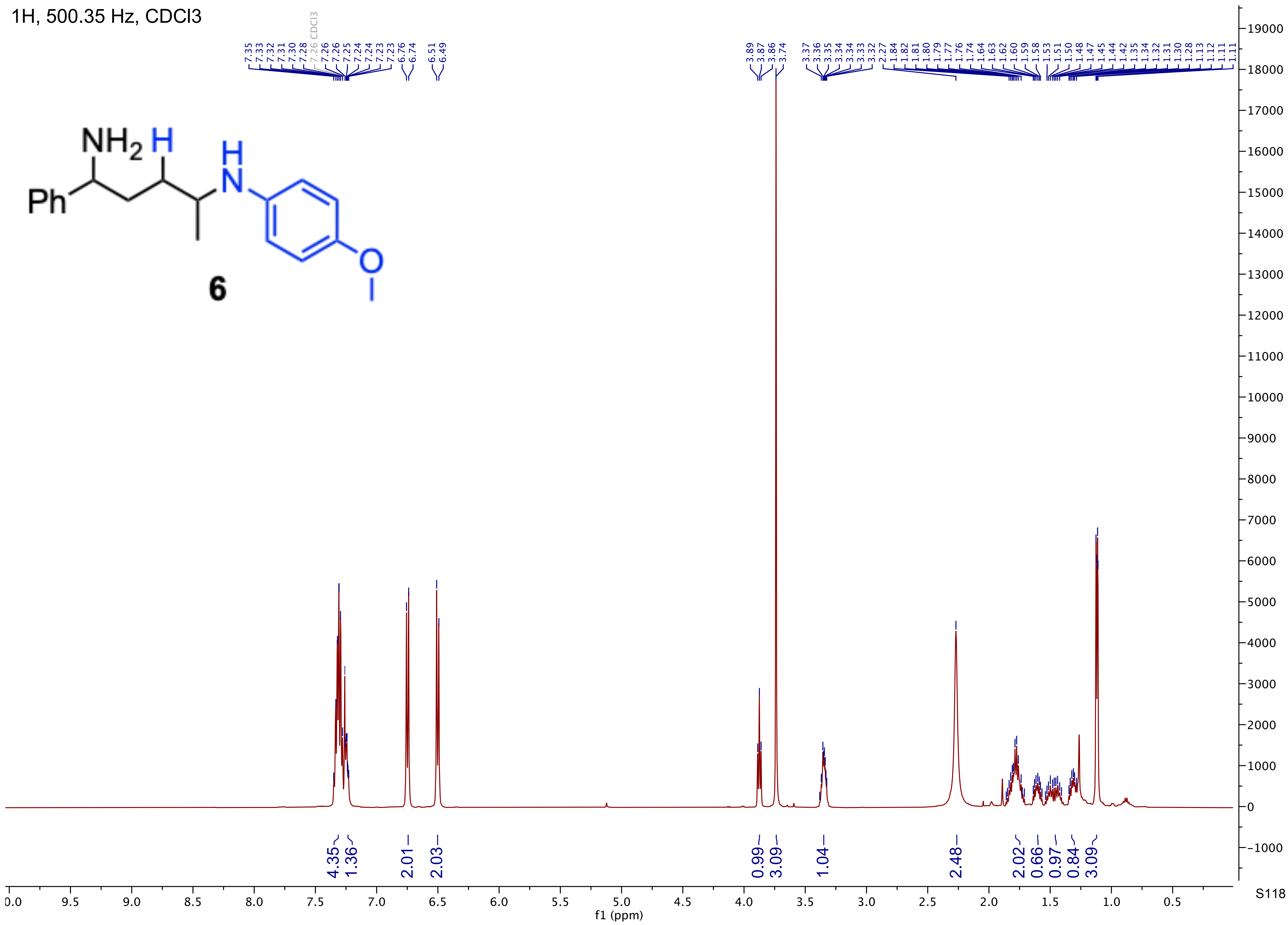
1H, 500.35 Hz, CDCl3
6
7.35 7.33 7.32 7.31 7.30 7.28 7.26 CDCl3 7.26 7.26 7.25 7.25 7.24 7.24 7.23 7.23 6.76 6.74 6.51 6.49
3.89 3.87 3.86 3.74 3.37 3.36 3.35 3.34 3.34 3.33 3.32 2.27 1.84 1.82 1.81 1.80 1.79 1.77 1.76 1.74 1.64 1.63 1.62 1.60 1.59 1.58 1.53 1.51 1.50 1.48 1.47 1.45 1.44 1.42 1.35 1.34 1.32 1.31 1.30 1.28 1.13 1.12 1.11 1.11
4.35 1.36 2.01 2.03 0.99 3.09 1.04 2.48 2.02 0.66 0.97 0.84 3.09
f1 (ppm)

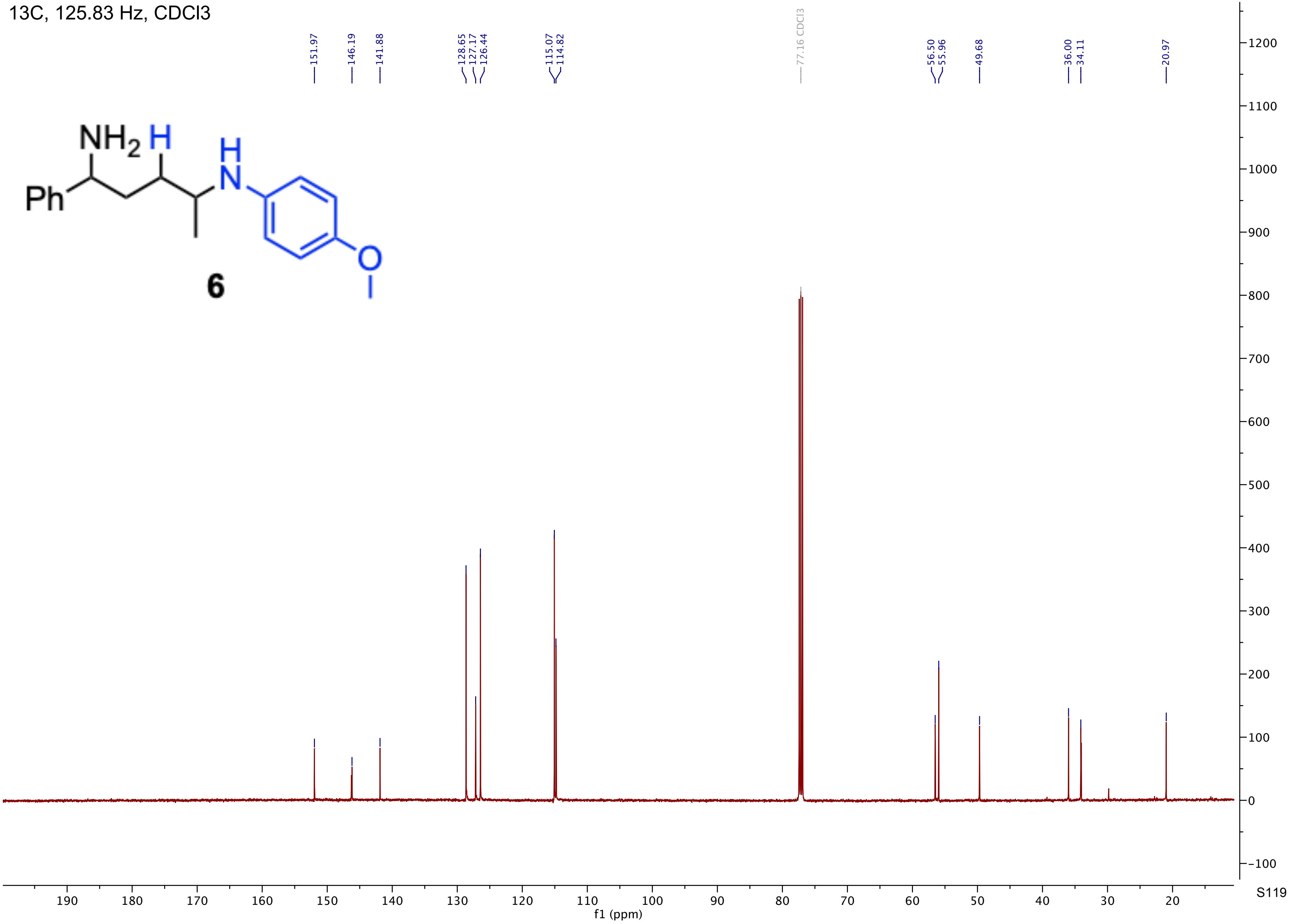
13C, 125.83 Hz, CDCl3
151.97
146.19
141.88
128.65
127.17
126.44
115.07
114.82
77.16 CDCl3
56.50
55.96
49.68
36.00
34.11
20.97
NH2
H
H
N
Ph
O
6
1200
1100
1000
900
800
700
600
500
400
300
200
100
0
−100
190
180
170
160
150
140
130
120
110
100
90
80
70
60
50
40
30
20
f1 (ppm)

1H, 500.35 Hz, CDCl3

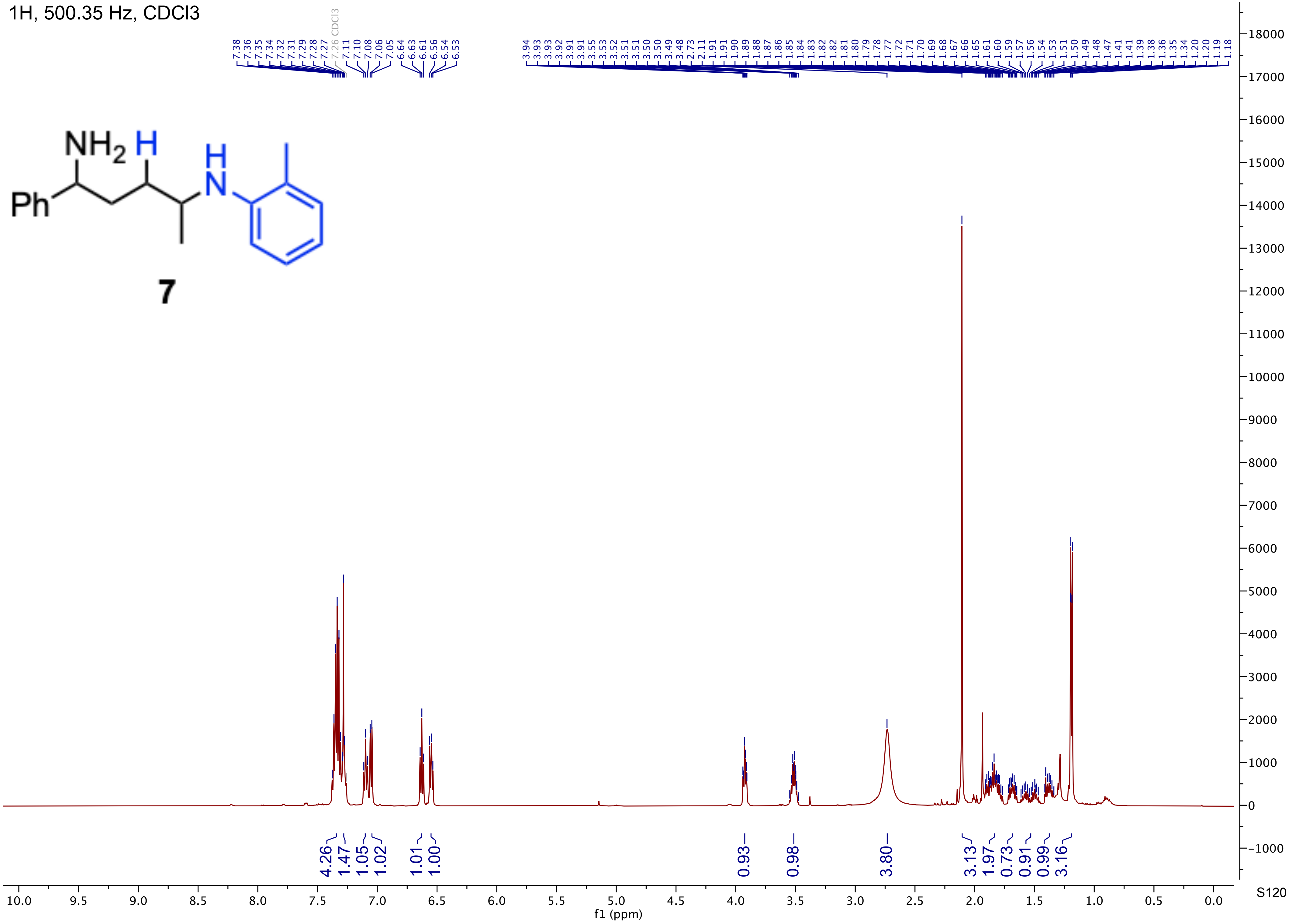

7

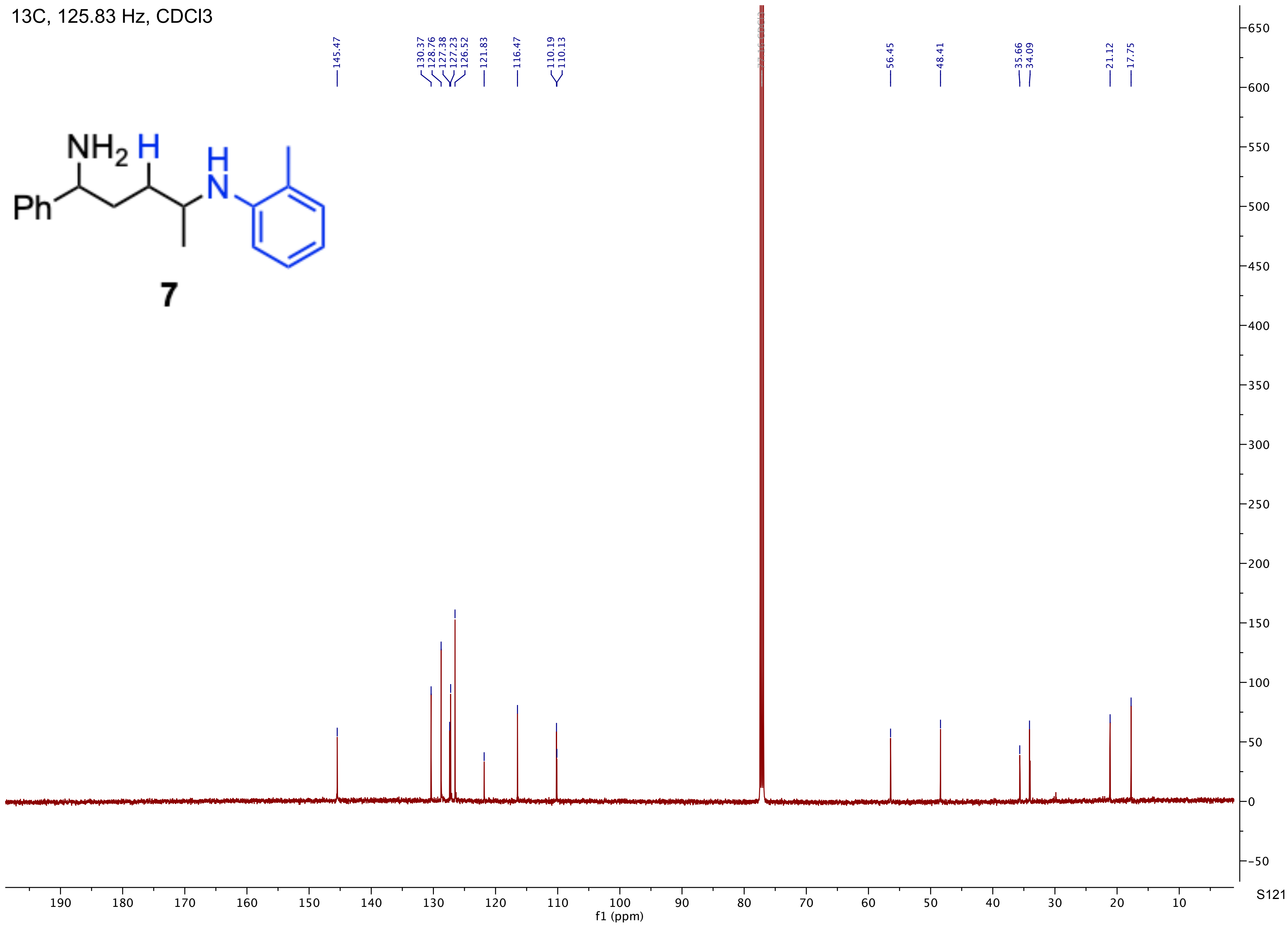
13C, 125.83 Hz, CDCl3
145.47
130.37
128.76
127.38
127.23
126.52
121.83
116.47
110.19
110.13
56.45
48.41
35.66
34.09
21.12
17.75
NH2
H
Ph
7
f1 (ppm)

1H, 399.95 Hz, cdcl3

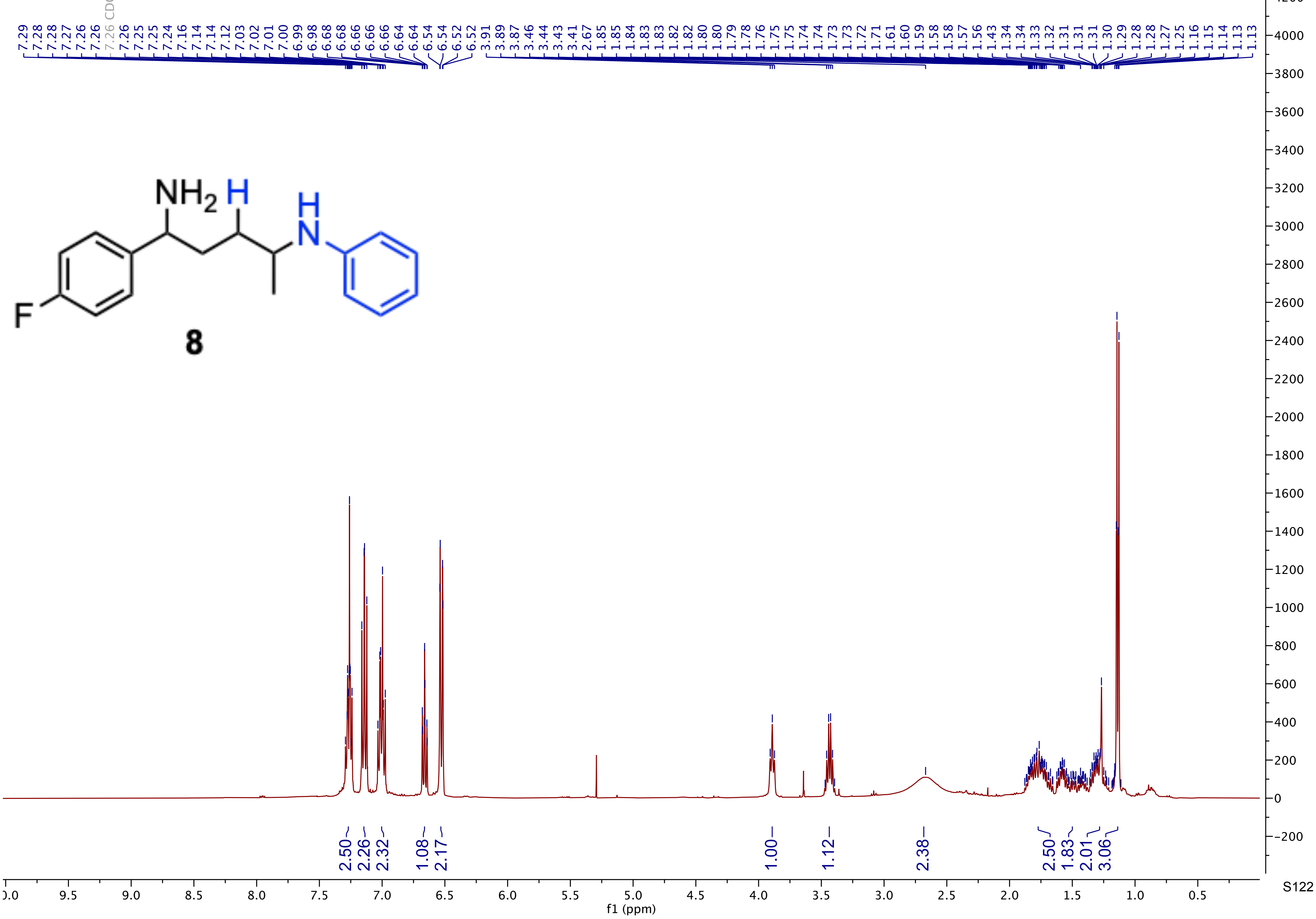

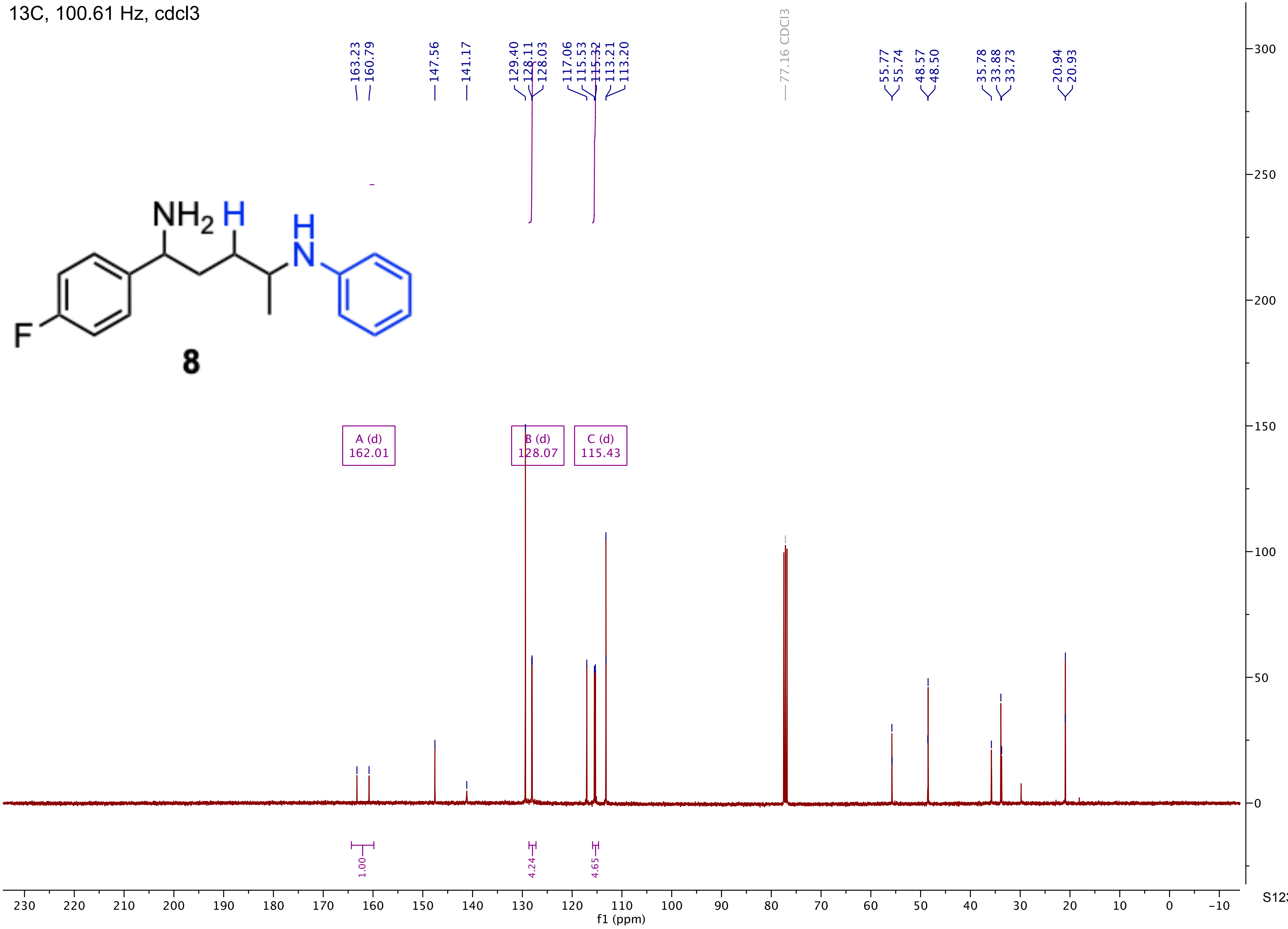
13C, 100.61 Hz, cdcl3
163.23
160.79
147.56
141.17
129.40
128.11
128.03
117.06
115.53
115.32
113.21
113.20
77.16 CDCl3
55.77
55.74
48.57
48.50
35.78
33.88
33.73
20.94
20.93
NH2 H
H
N
F
8
A (d)
162.01
B (d)
128.07
C (d)
115.43
1.00
4.24
4.65
f1 (ppm)

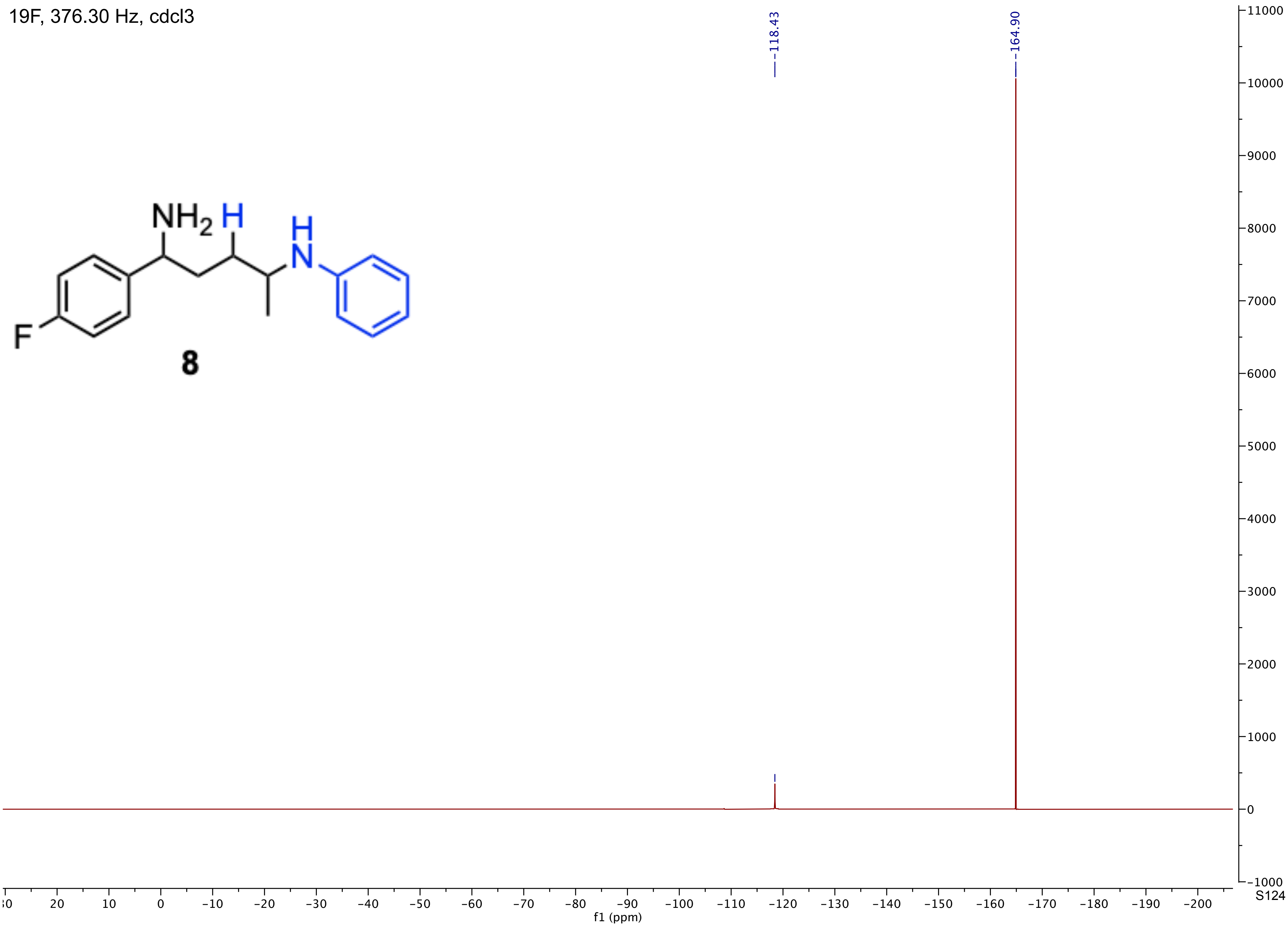
19F, 376.30 Hz, cdcl3
-118.43
-164.90
NH2
H
H
N
F
8
f1 (ppm)
11000
10000
9000
8000
7000
6000
5000
4000
3000
2000
1000
0
-1000
20
10
0
-10
-20
-30
-40
-50
-60
-70
-80
-90
-100
-110
-120
-130
-140
-150
-160
-170
-180
-190
-200

1H, 500.35 Hz, CDCl3

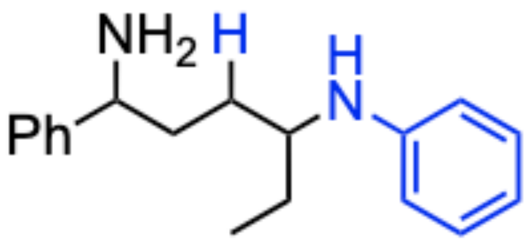

9

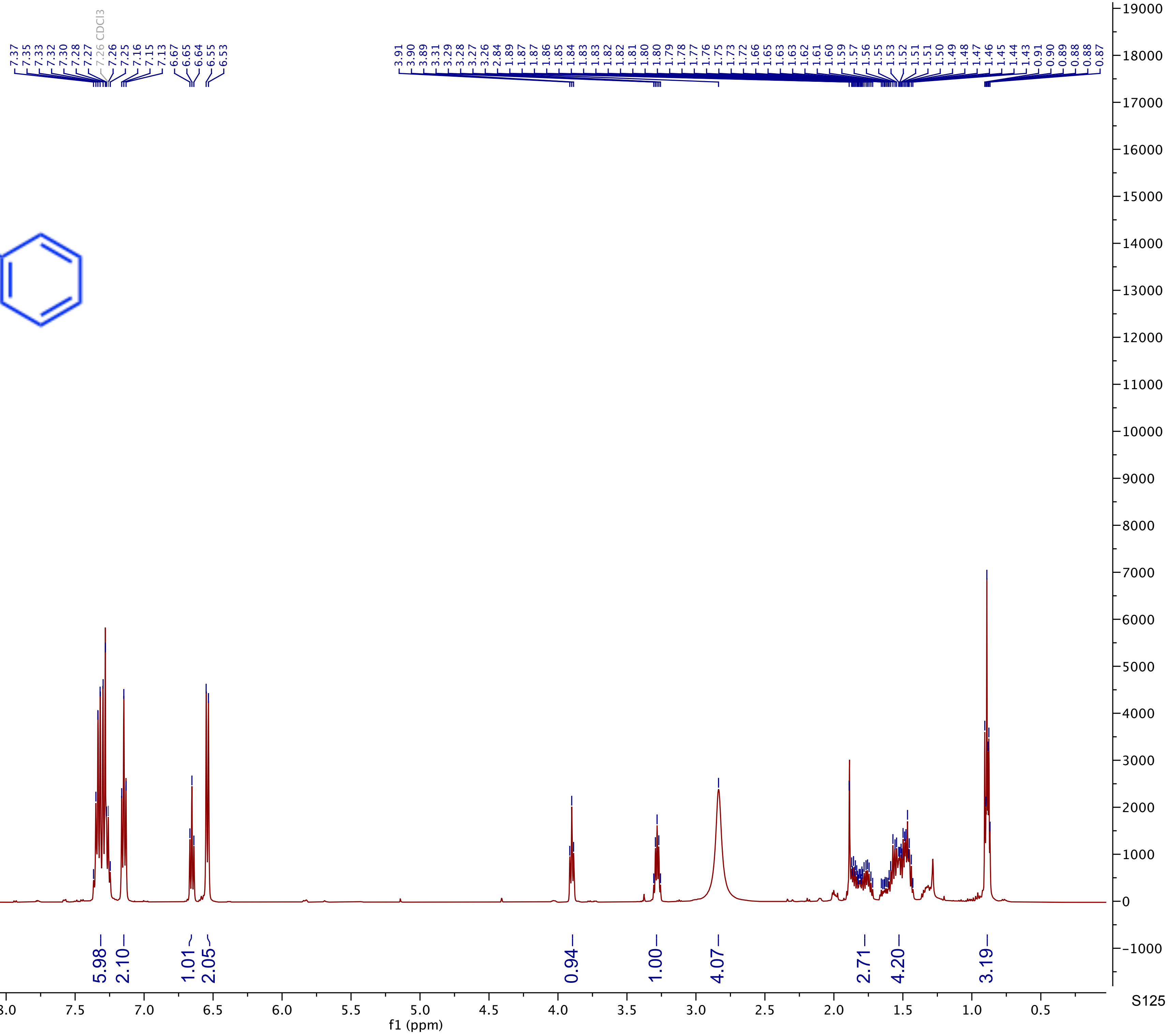

13C, 125.83 Hz, CDCl3

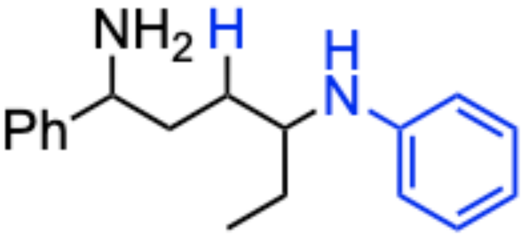

9

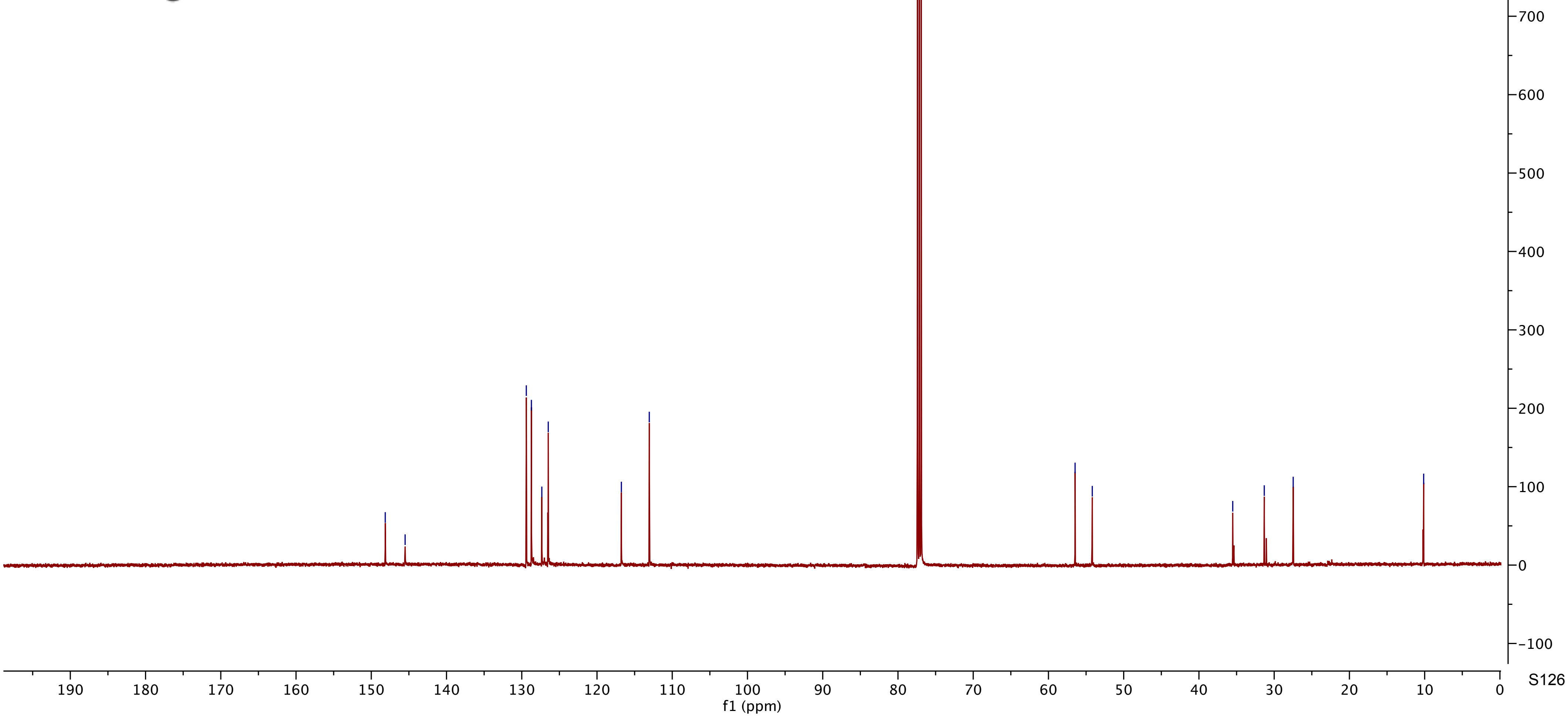

1H, 500.35 Hz, CDCl3

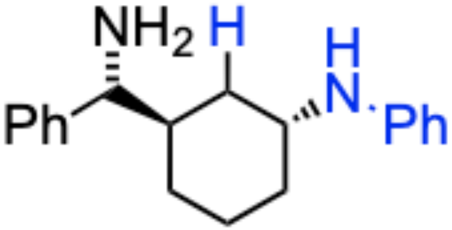

10

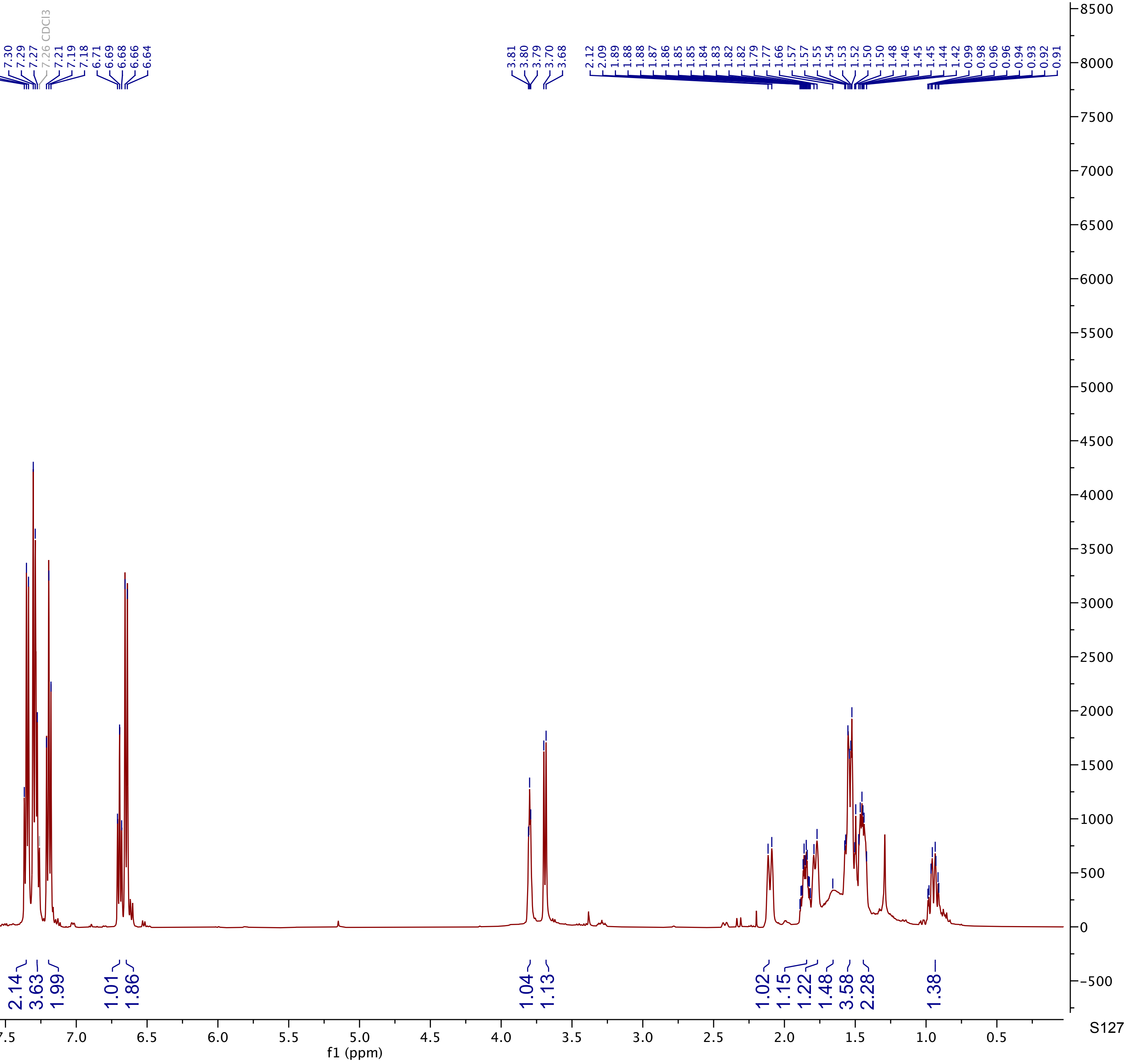

13C, 125.83 Hz, CDCl3

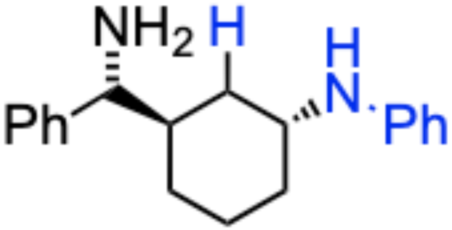

10

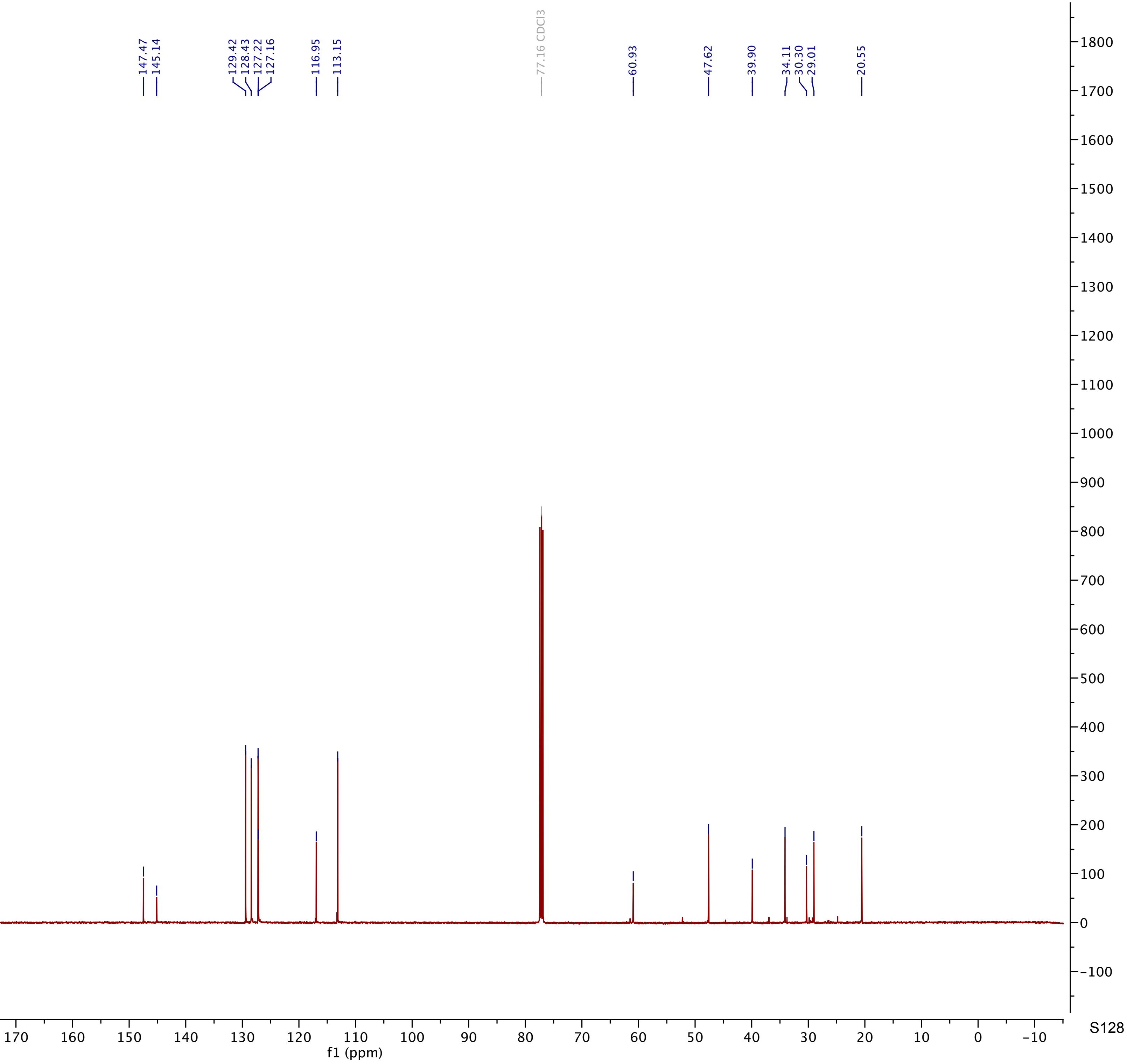

1H, 500.35 Hz, CDCl3

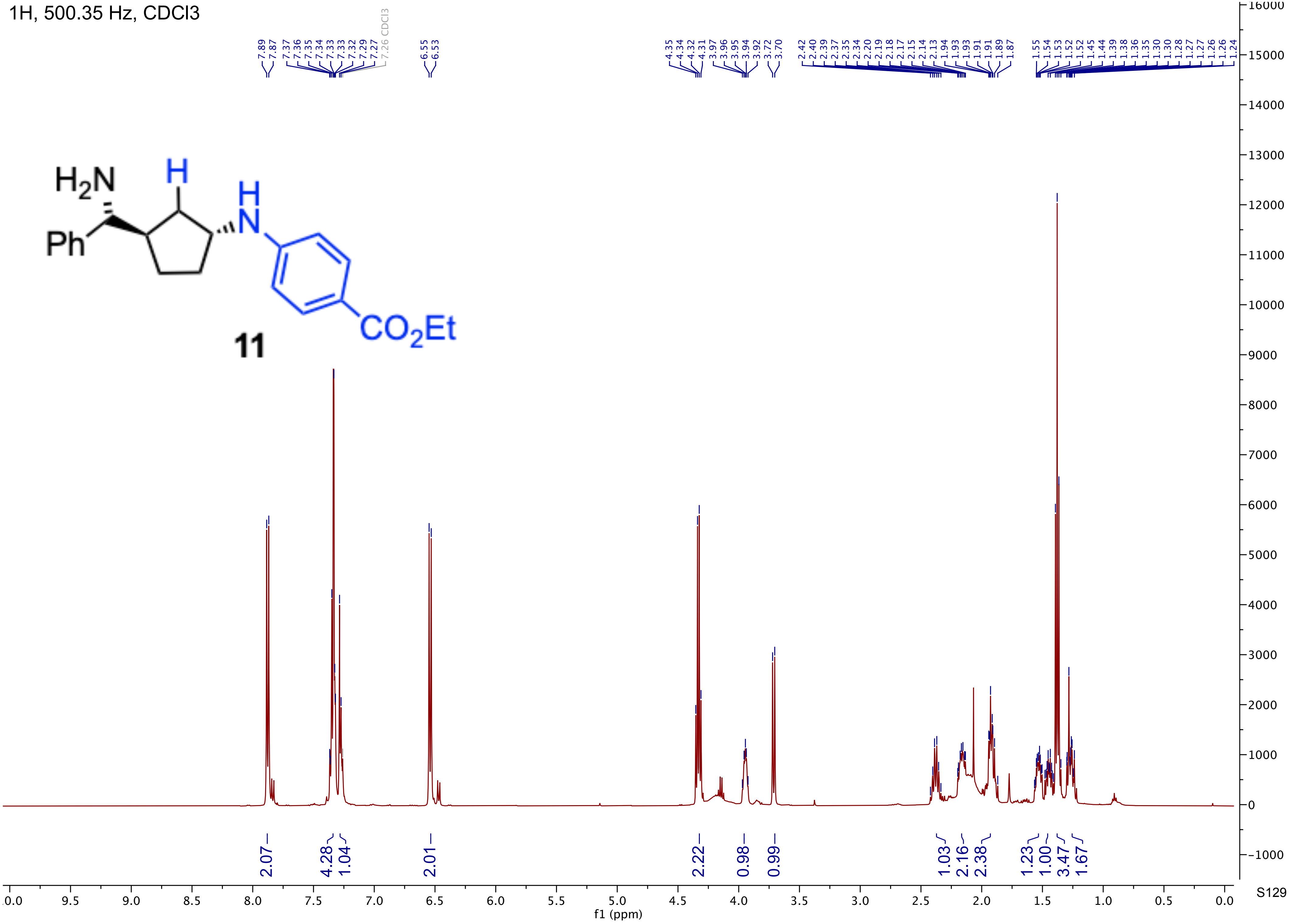

13C, 125.83 Hz, CDCl3

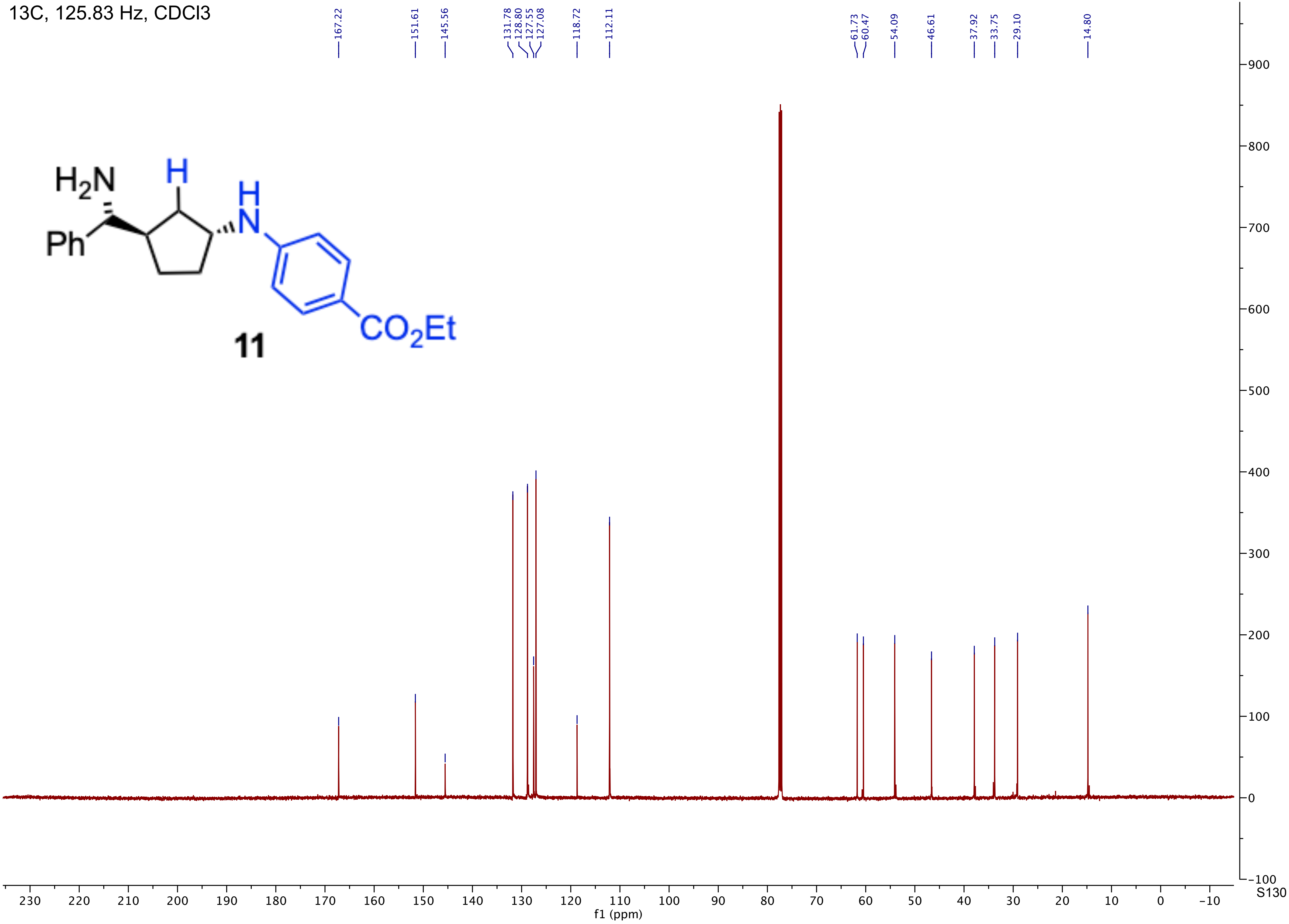

1H, 500.35 Hz, CDCl3

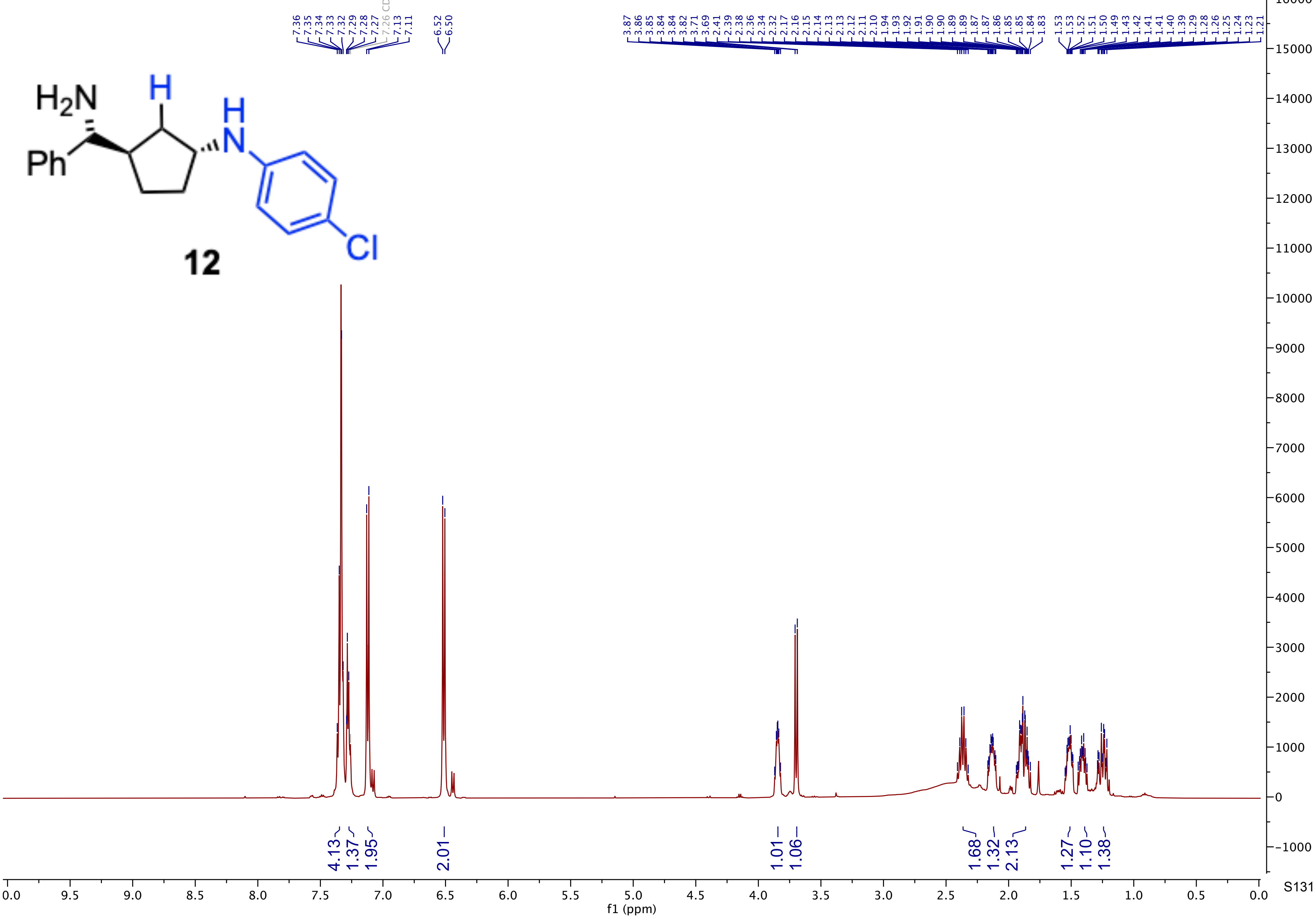

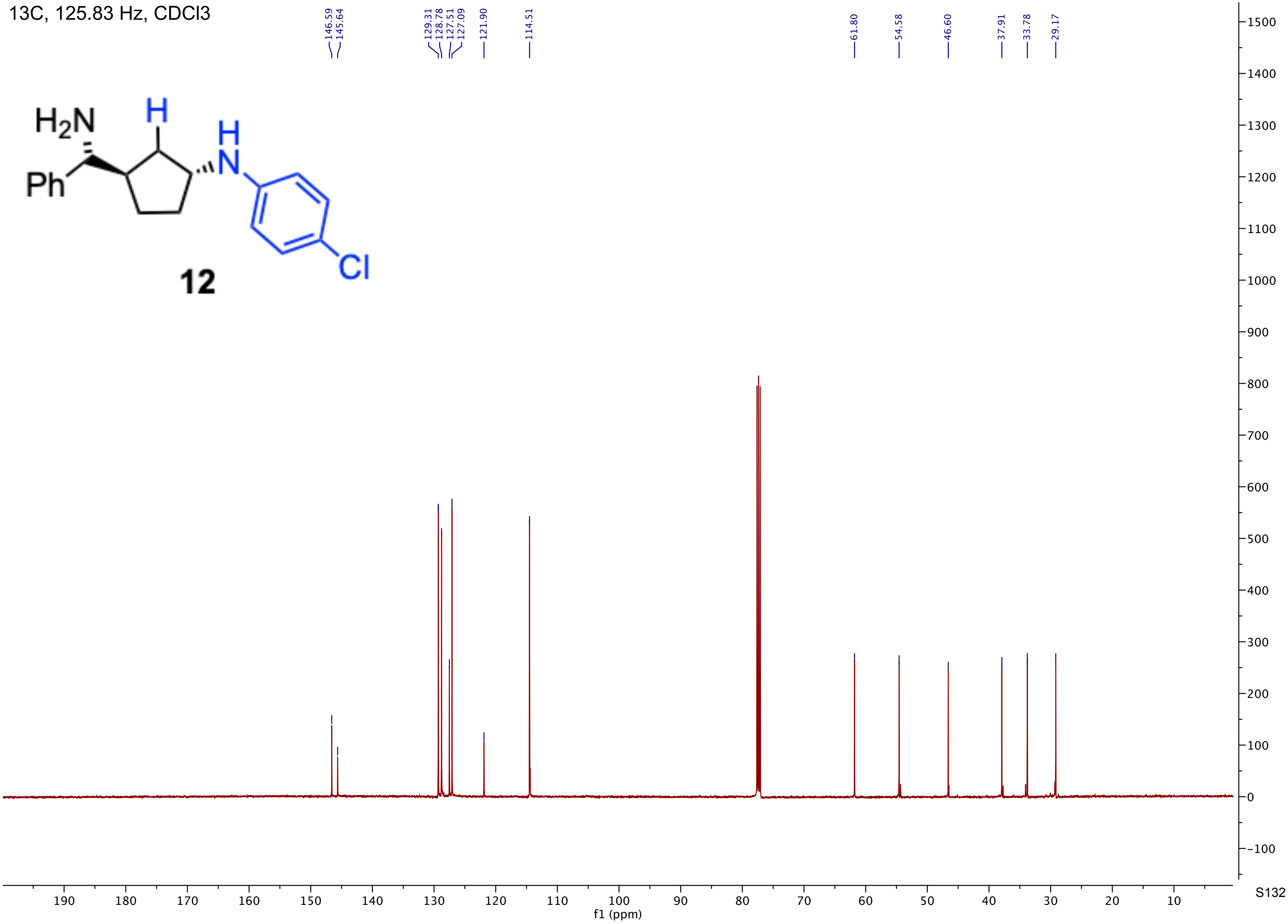
13C, 125.83 Hz, CDCl3
146.59
145.64
129.31
128.78
127.51
127.09
121.90
114.51
61.80
54.58
46.60
37.91
33.78
29.17
H2N
H
H
N
Ph
Cl
12
1500
1400
1300
1200
1100
1000
900
800
700
600
500
400
300
200
100
0
−100
190
180
170
160
150
140
130
120
110
100
90
80
70
60
50
40
30
20
10
f1 (ppm)

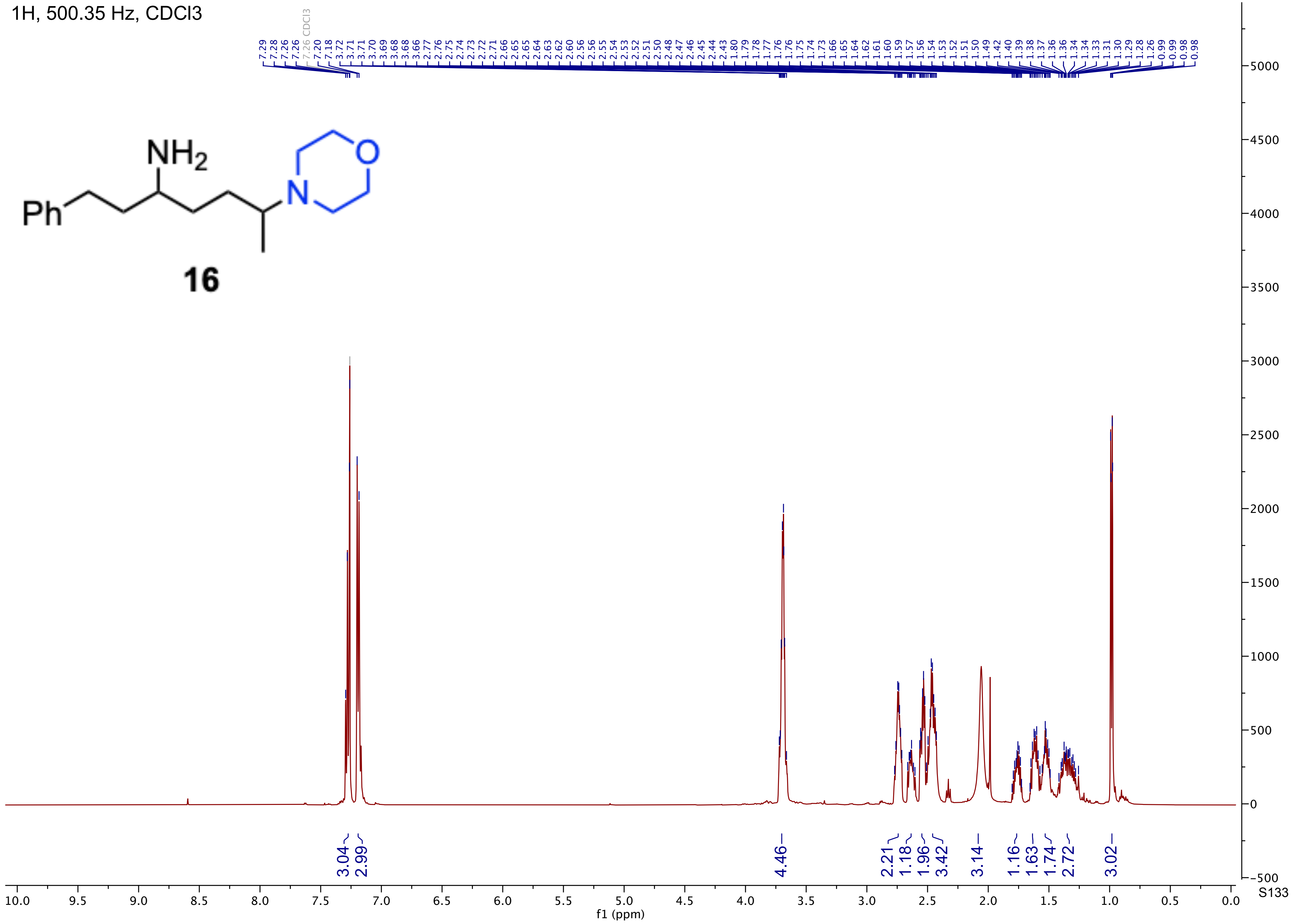
1H, 500.35 Hz, CDCl3
7.29 7.28 7.26 7.26 7.26 CDCl3 7.20 7.18 3.72 3.71 3.71 3.70 3.69 3.68 3.66 2.77 2.76 2.75 2.74 2.73 2.72 2.71 2.66 2.65 2.64 2.63 2.62 2.60 2.56 2.55 2.54 2.53 2.52 2.51 2.50 2.48 2.47 2.46 2.45 2.44 2.43 1.80 1.79 1.78 1.77 1.76 1.76 1.75 1.74 1.73 1.66 1.65 1.64 1.62 1.61 1.60 1.59 1.57 1.56 1.54 1.53 1.52 1.51 1.50 1.49 1.42 1.40 1.39 1.38 1.37 1.36 1.36 1.34 1.34 1.33 1.31 1.30 1.29 1.28 1.26 0.99 0.98 0.98
NH2
O
N
Ph
16
3.04 2.99 4.46 2.21 1.18 1.96 3.42 3.14 1.16 1.63 1.74 2.72 3.02
f1 (ppm)

13C, 125.83 Hz, CDCl3

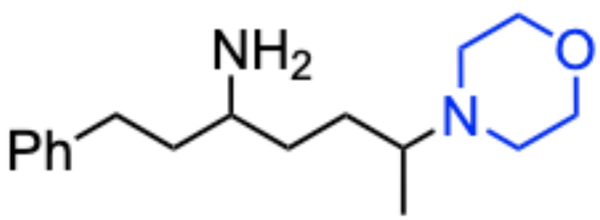

16

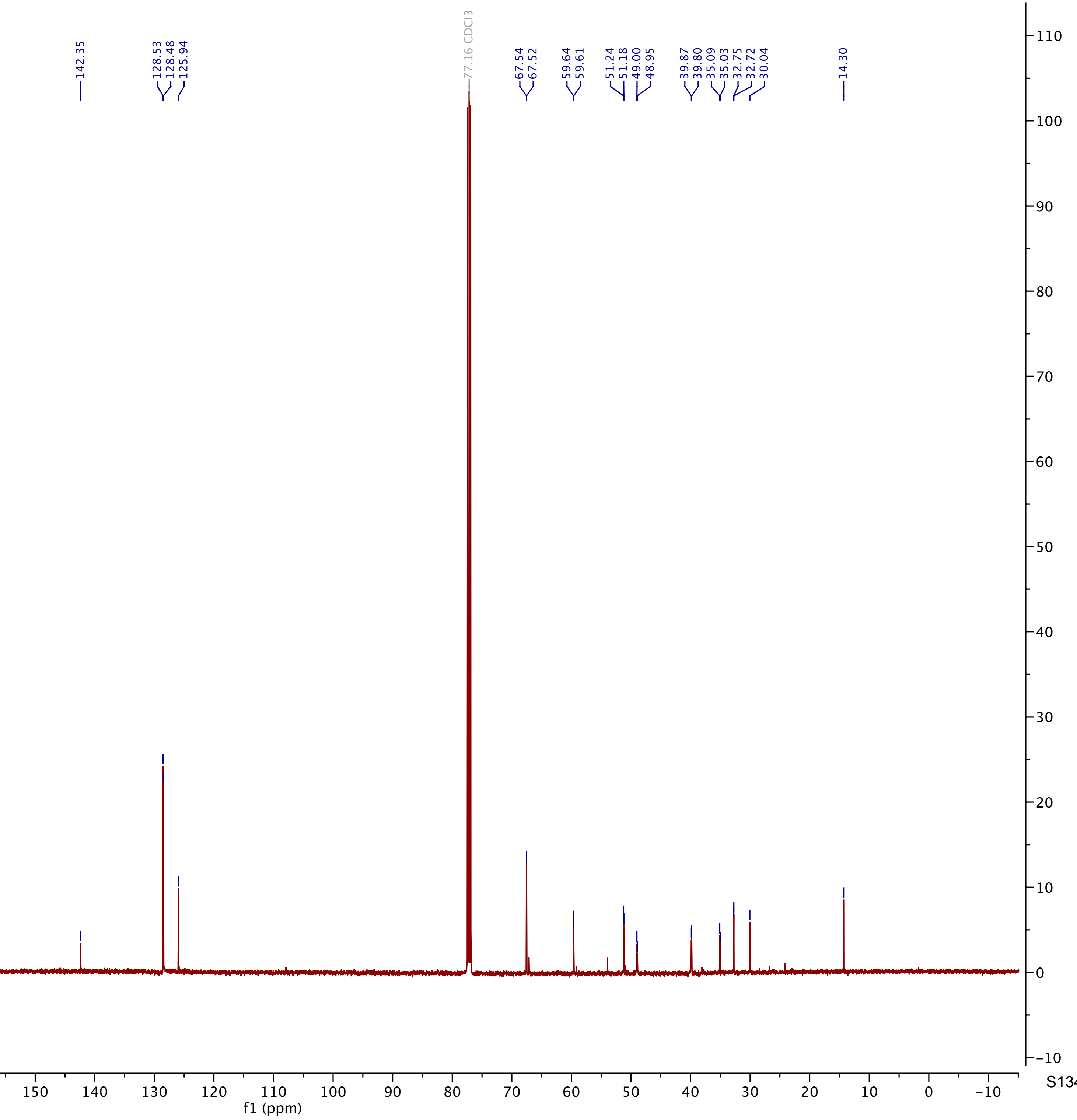

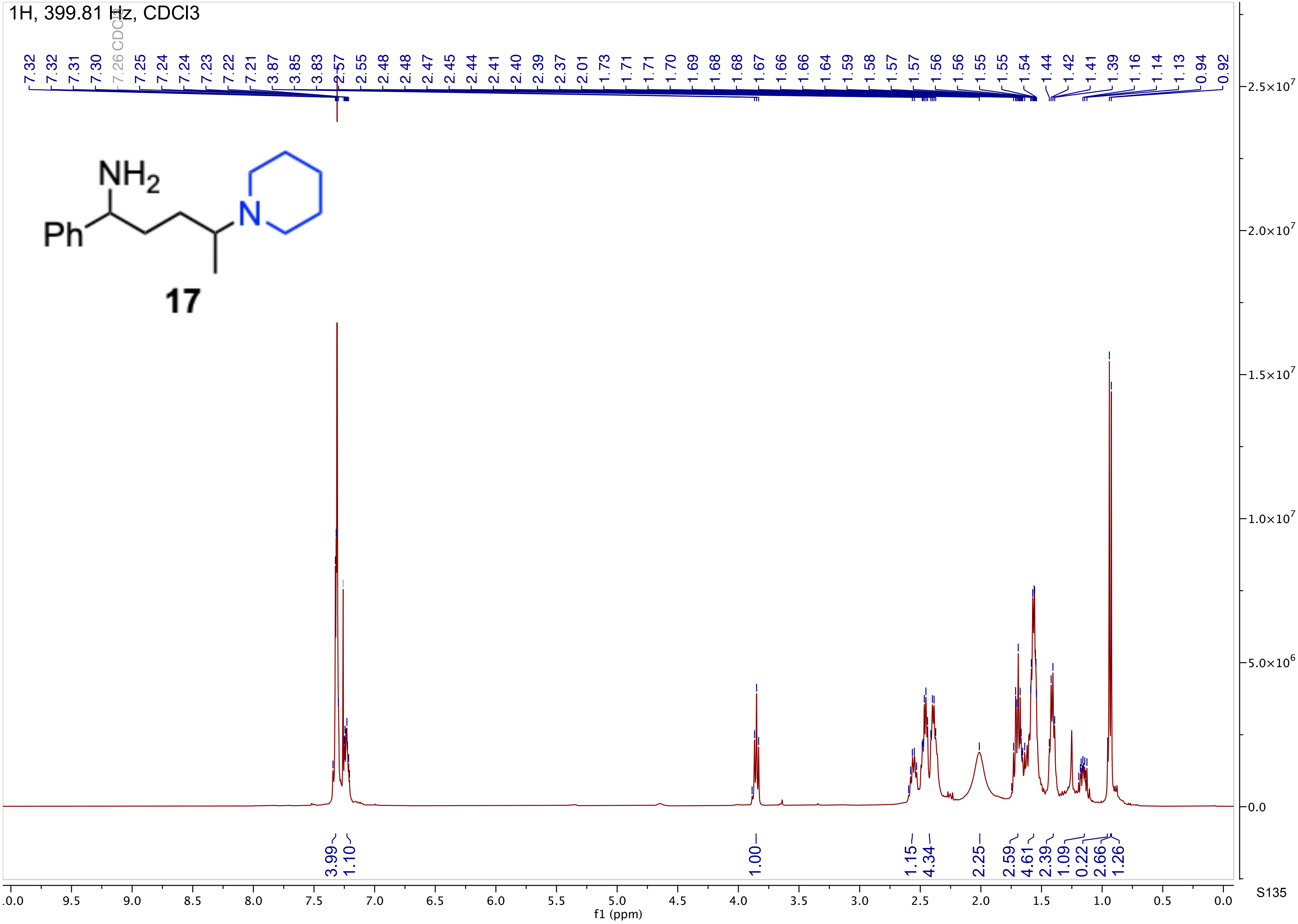
1H, 399.81 Hz, CDCl3
7.32 7.32 7.31 7.30 7.26 CDCl3 7.25 7.24 7.24 7.23 7.22 7.21 3.87 3.85 3.83 2.57 2.55 2.48 2.48 2.47 2.45 2.44 2.41 2.40 2.39 2.37 2.01 1.73 1.71 1.71 1.70 1.69 1.68 1.68 1.67 1.66 1.66 1.64 1.59 1.58 1.57 1.57 1.56 1.56 1.55 1.55 1.54 1.44 1.42 1.41 1.39 1.16 1.14 1.13 0.94 0.92
NH2
Ph
N
17
3.99 1.10 1.00 1.15 4.34 2.25 2.59 4.61 2.39 1.09 0.22 2.66 1.26
f1 (ppm)

13C, 100.54 Hz, CDCl3

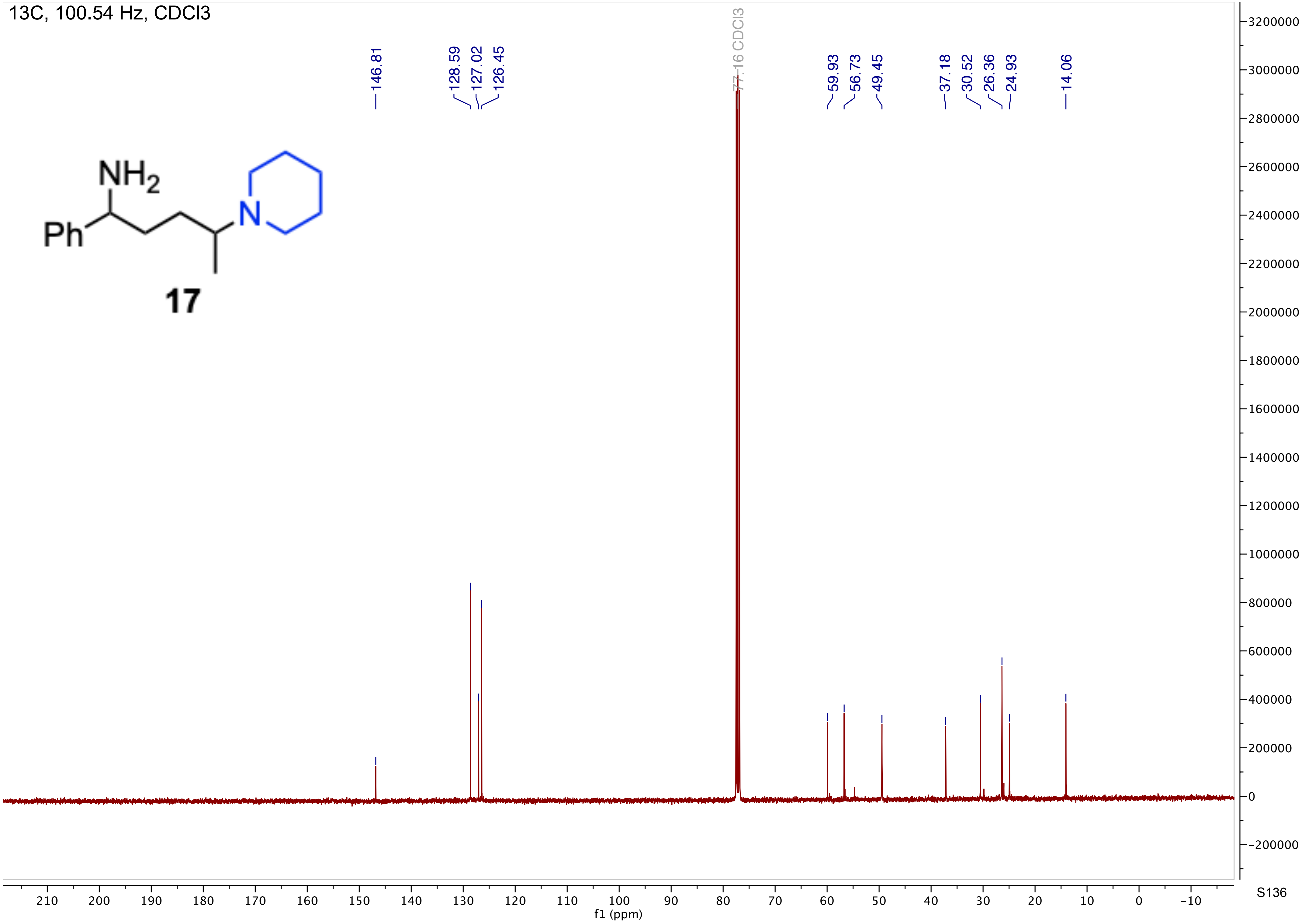

1H, 500.11 Hz, CDCl3

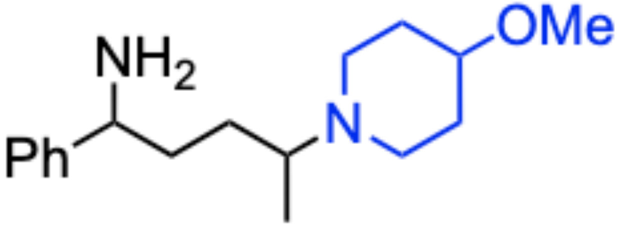

18

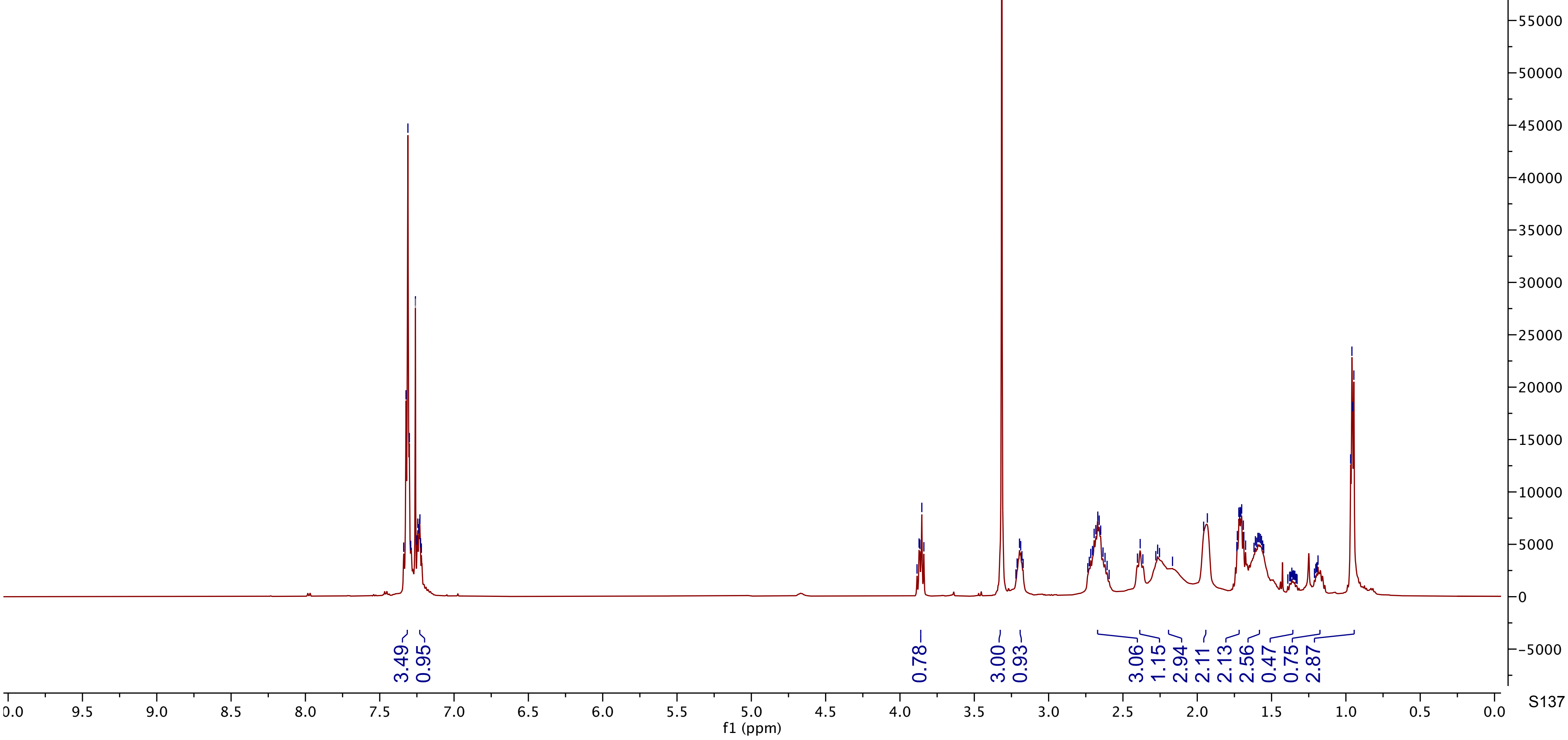

13C, 100.54 Hz, CDCl3

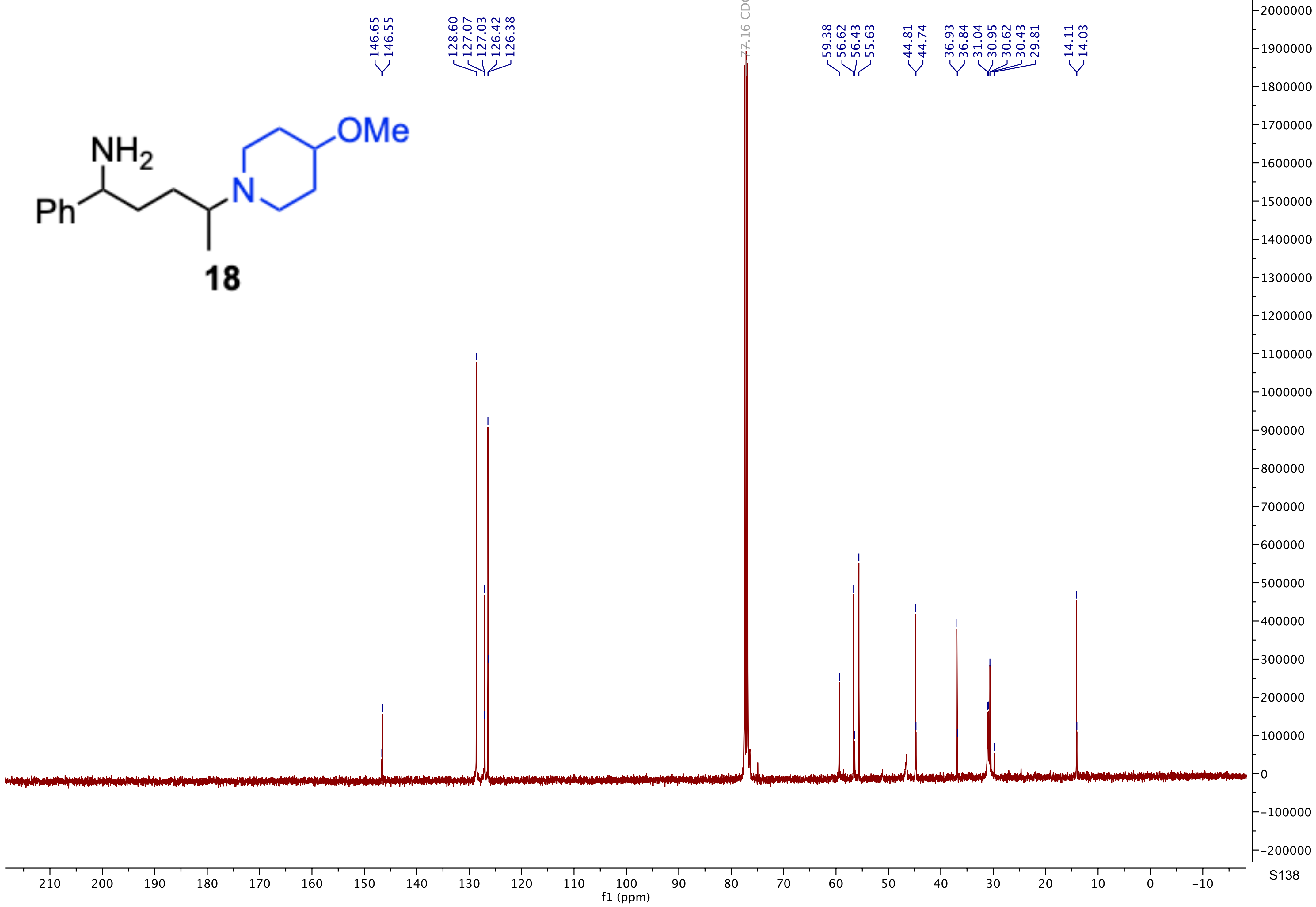

1H, 500.18 Hz, CDCl3

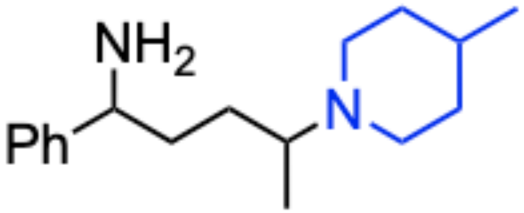

19

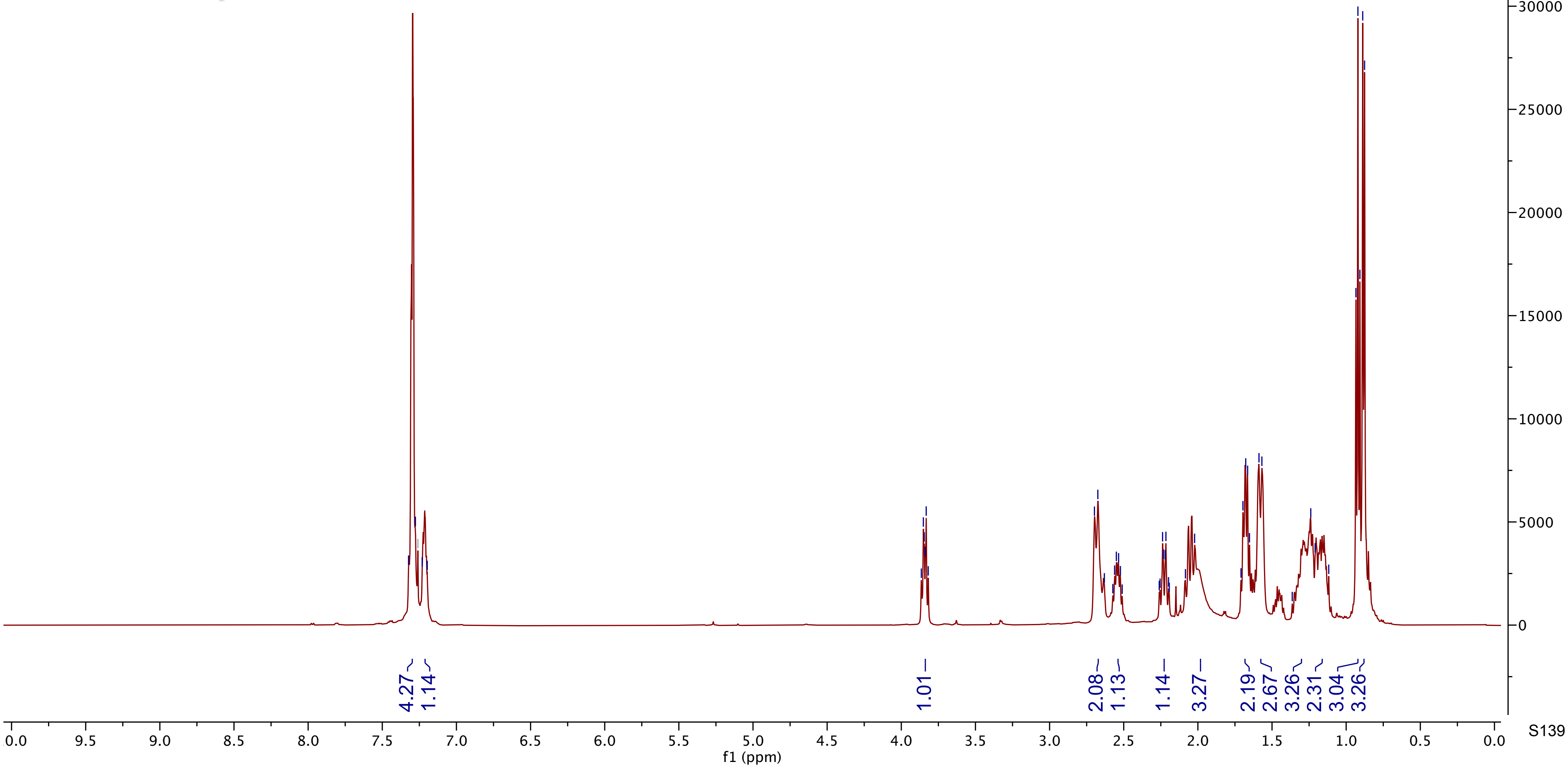

13C, 125.78 Hz, CDCl3

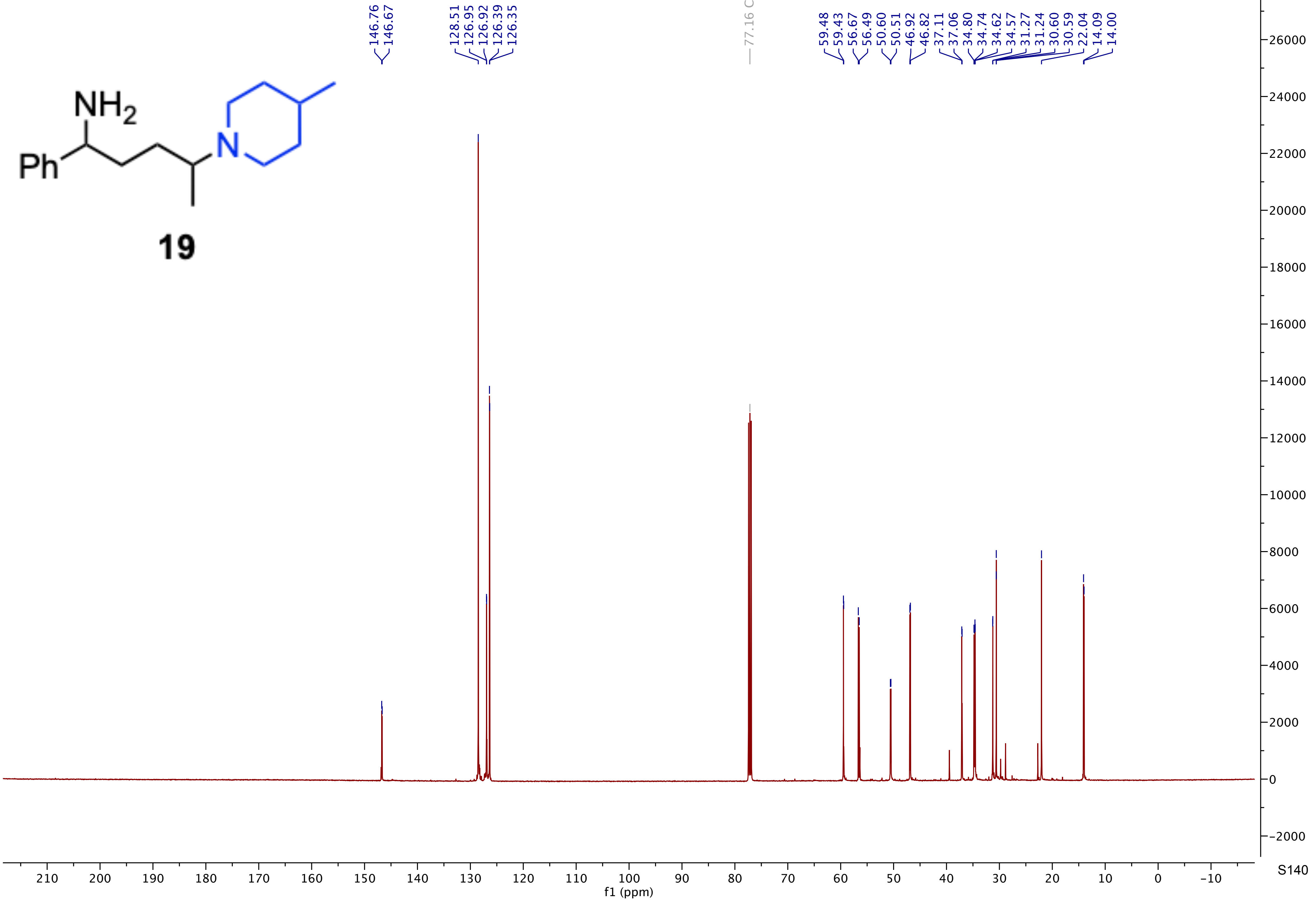

19

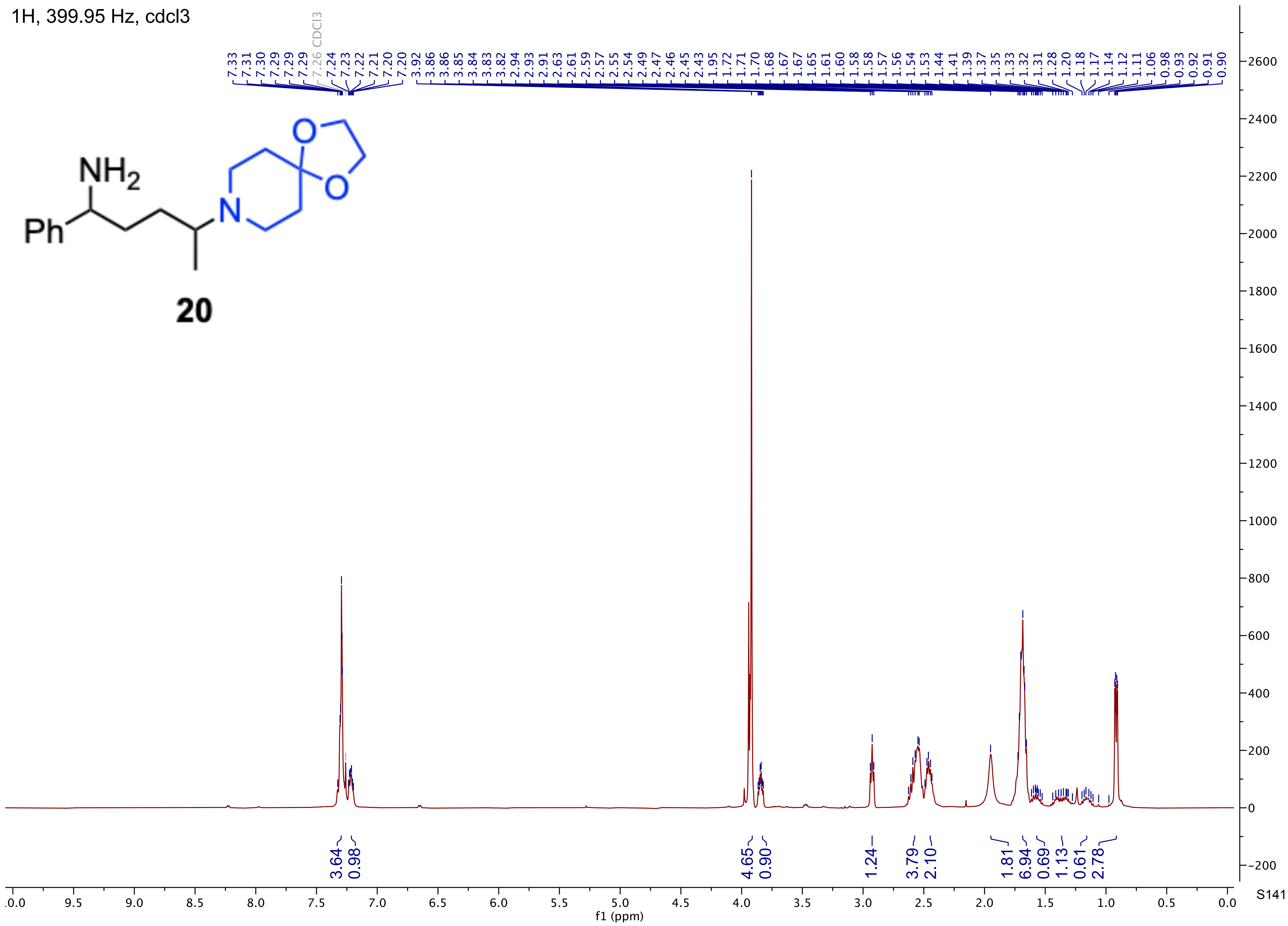
1H, 399.95 Hz, cdcl3
7.33 7.31 7.30 7.29 7.29 7.29 7.26 CDCl3 7.24 7.23 7.22 7.21 7.20 7.20
3.92 3.86 3.85 3.84 3.83 3.82 2.94 2.93 2.91 2.63 2.61 2.59 2.57 2.55 2.54 2.49 2.47 2.46 2.45 2.43 1.95 1.72 1.71 1.70 1.68 1.67 1.67 1.65 1.61 1.60 1.58 1.58 1.57 1.56 1.54 1.53 1.44 1.41 1.39 1.37 1.35 1.33 1.32 1.31 1.28 1.20 1.18 1.17 1.14 1.12 1.11 1.06 0.98 0.93 0.92 0.91 0.90
NH2
Ph
N
O
O
20
3.64 0.98 4.65 0.90 1.24 3.79 2.10 1.81 6.94 0.69 1.13 0.61 2.78
f1 (ppm)

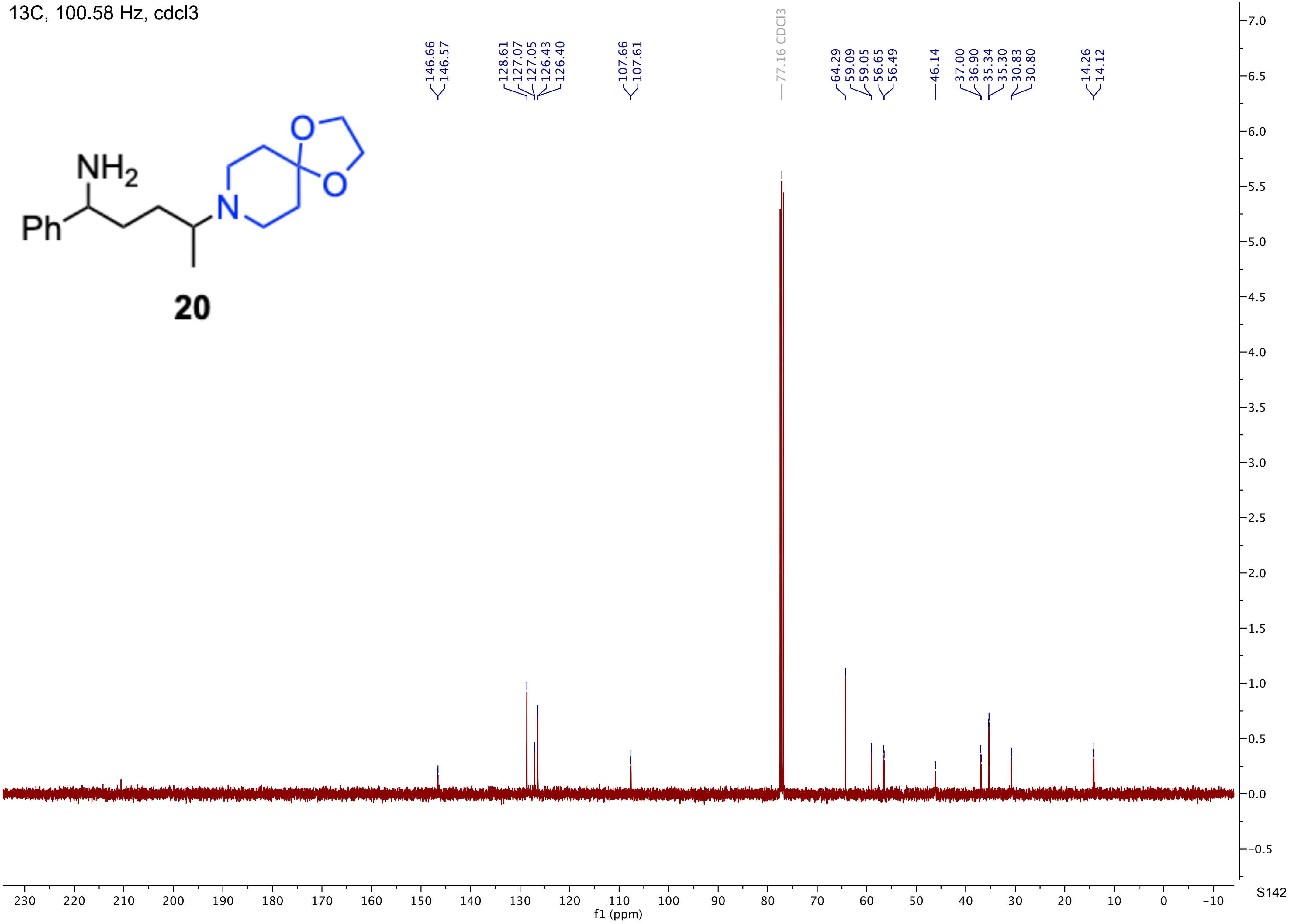
13C, 100.58 Hz, cdcl3
146.66
146.57
128.61
127.07
127.05
126.43
126.40
107.66
107.61
77.16 CDCl3
64.29
59.09
59.05
56.65
56.49
46.14
37.00
36.90
35.34
35.30
30.83
30.80
14.26
14.12
NH2
Ph
N
O
O
20
f1 (ppm)

1H, 500.18 Hz, CDCl3

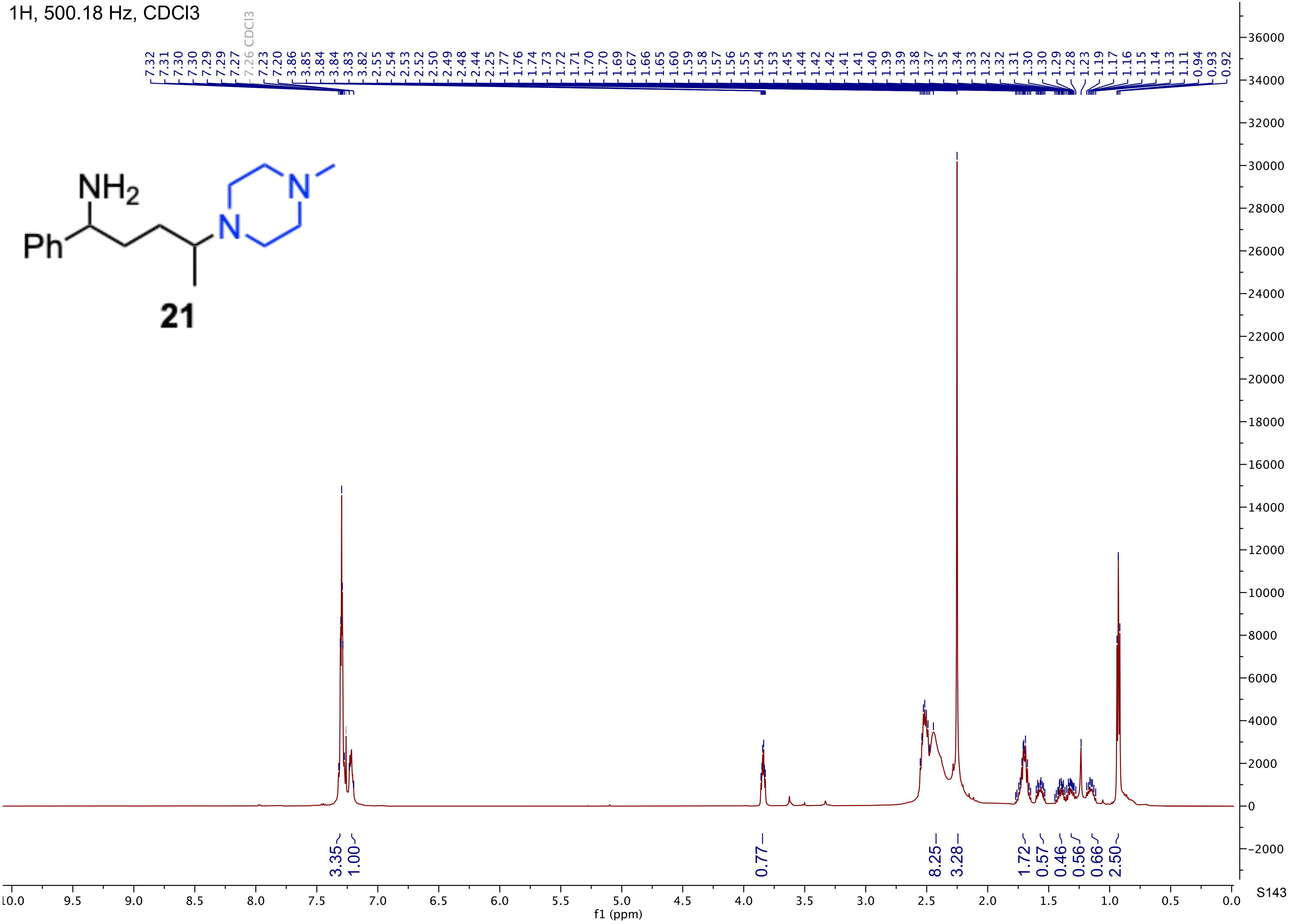

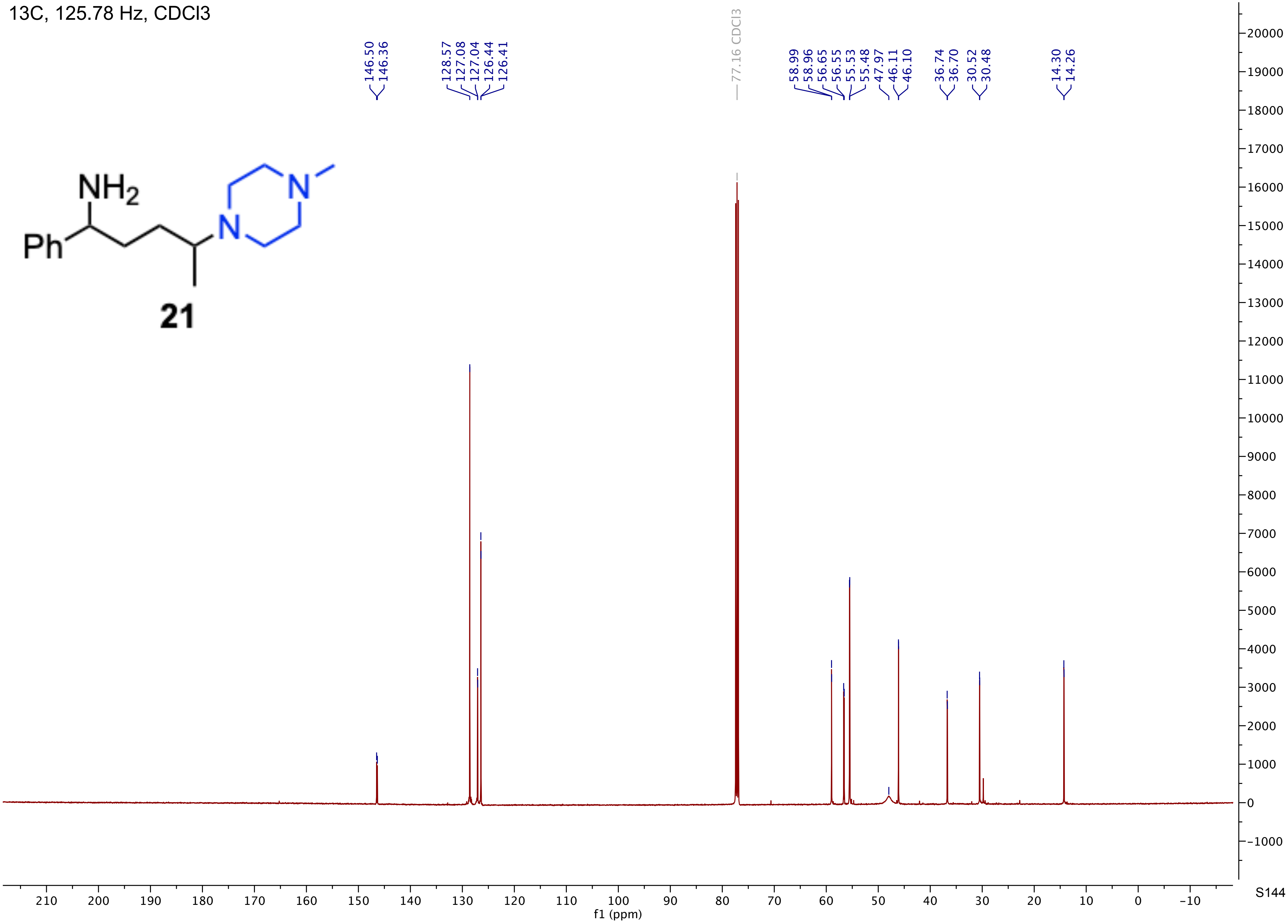
13C, 125.78 Hz, CDCl3
146.50
146.36
128.57
127.08
127.04
126.44
126.41
77.16 CDCl3
58.99
58.96
56.65
56.55
55.53
55.48
47.97
46.11
46.10
36.74
36.70
30.52
30.48
14.30
14.26
NH2
Ph
N
N
21
f1 (ppm)
210 200 190 180 170 160 150 140 130 120 110 100 90 80 70 60 50 40 30 20 10 0 −10
20000 19000 18000 17000 16000 15000 14000 13000 12000 11000 10000 9000 8000 7000 6000 5000 4000 3000 2000 1000 0 −1000

1H, 500.35 Hz, CDCl3

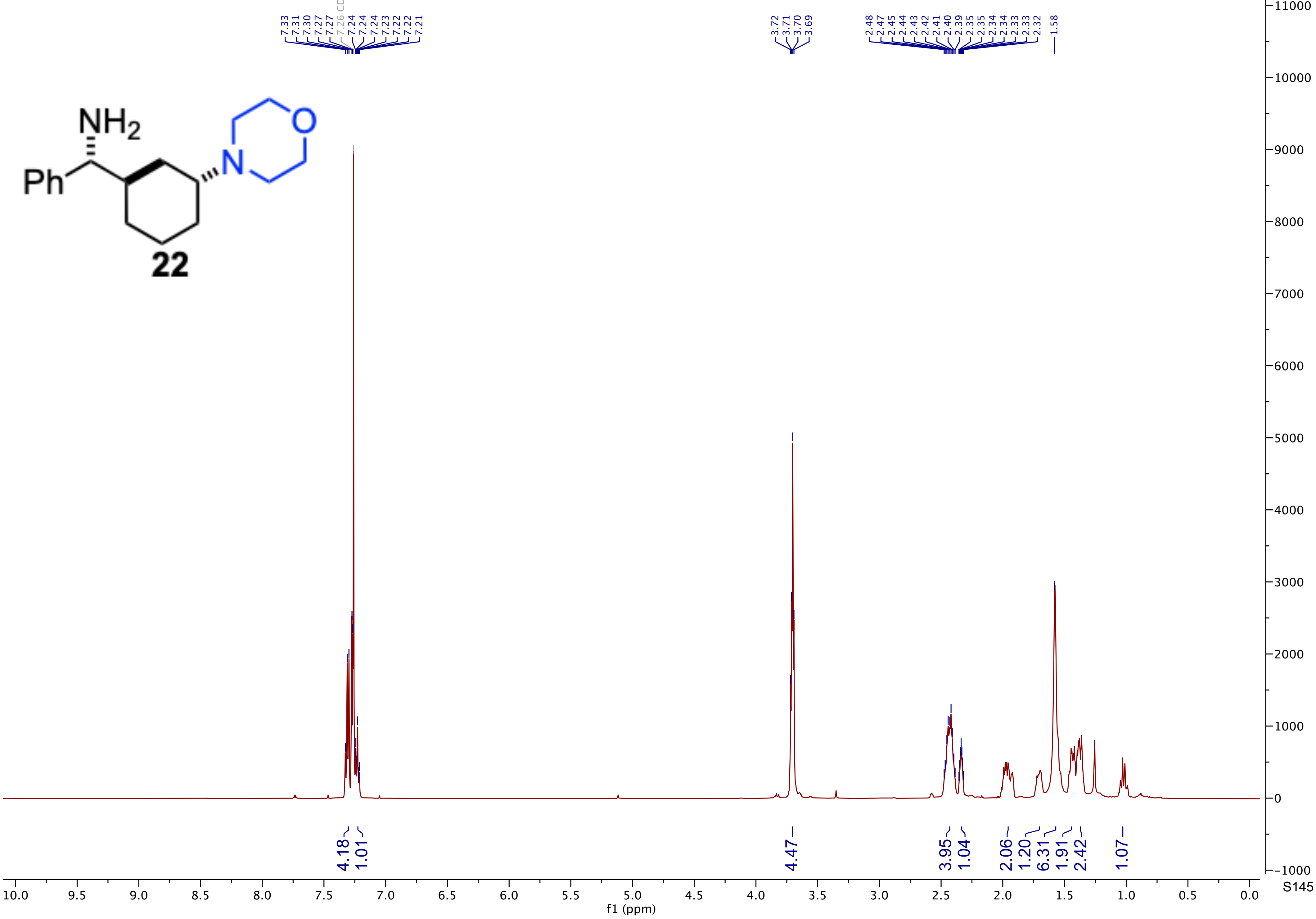

13C, 125.83 Hz, CDCl3

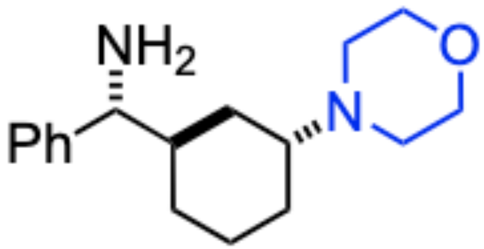

22

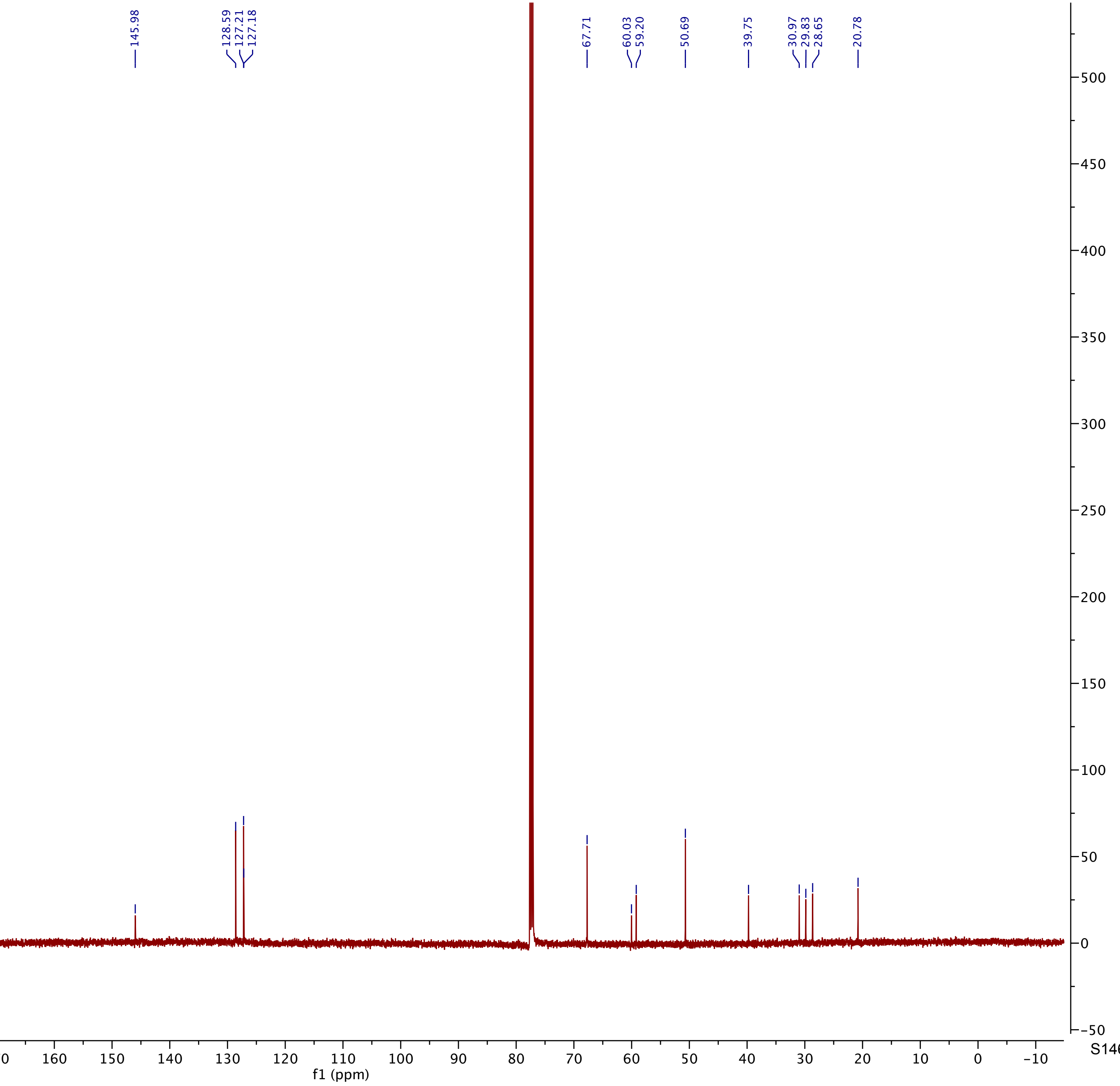

Supplement: CC-060-D3CC05594A-s001 [file CC-060-D3CC05594A-s001.pdf]
